# 4‐Phosphoryl Pyrazolones for Highly Selective Lithium Separation from Alkali Metal Ions

**DOI:** 10.1002/chem.202103640

**Published:** 2021-11-05

**Authors:** Jianfeng Zhang, Marco Wenzel, Johannes Steup, Gerrit Schaper, Felix Hennersdorf, Hao Du, Shili Zheng, Leonard F. Lindoy, Jan J. Weigand

**Affiliations:** ^1^ Faculty of Chemistry and Food Chemistry Technische Universität Dresden 01062 Dresden Germany; ^2^ National Engineering Laboratory for Hydrometallurgical Cleaner Production Technology Key Laboratory of Green Process and Engineering Institute of Process Engineering Chinese Academy of Sciences Beijing 100190 China; ^3^ School of Chemistry, F11 University of Sydney NSW 2006 Sydney Australia

**Keywords:** 4-phosphoryl pyrazolone ligand, Li ions, liquid-liquid/solid-liquid extraction, trinuclear Li complex, trioctylphosphine oxide

## Abstract

Effective receptors for the separation of Li^+^ from a mixture with other alkali metal ions under mild conditions remains an important challenge that could benefit from new approaches. In this study, it is demonstrated that the 4‐phosphoryl pyrazolones, H**L** ^2^‐H**L** ^4^, in the presence of the typical industrial organophosphorus co‐ligands tributylphosphine oxide (TBPO), tributylphosphate (TBP) and trioctylphosphine oxide (TOPO), are able to selectively recognise and extract lithium ions from aqueous solution. Structural investigations in solution as well as in the solid state reveal the existence of a series of multinuclear Li^+^ complexes that include dimers (TBPO, TBP) as well as rarely observed trimers (TOPO) and represent the first clear evidence for the synergistic role of the co‐ligands in the extraction process. Our findings are supported by detailed NMR, MS and extraction studies. Liquid‐liquid extraction in the presence of TOPO revealed an unprecedented high Li^+^ extraction efficiency (78 %) for H**L** ^4^ compared to the use of the industrially employed acylpyrazolone H**L** ^1^ (15 %) and benzoyl‐1,1,1‐trifluoroacetone (52 %) extractants. In addition, a high selectivity for Li^+^ over Na^+^, K^+^ and Cs^+^ under mild conditions (pH ∼8.2) confirms that H**L** ^2^‐H**L** ^4^ represent a new class of ligands that are very effective extractants for use in lithium separation.

## Graphical abstract

**4‐phosphoryl pyrazolone** as new type of selective lithium receptors were synthesized. The bi‐ and trinuclear lithium complexes relying on co‐ligands (TBPO, TBP and TOPO) are reported for the first time. The unexpected aggregation conversion (from tri‐ to binuclear) in different TOPO equiv. was monitored by ^31^P, ^1^H and ^7^Li NMR. High extraction power and selectivity for Li^+^ at pH ∼8.2 show promising application in Li^+^ recognition and separation.

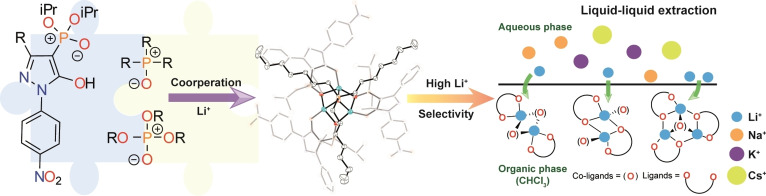

## Introduction

Lithium has attracted extensive commercial interest in recent decades since it is indispensable for the manufacture of lithium‐ion batteries (LIBs) and as a component of many other commercial goods that include glasses, ceramics, lubricants, and pharmaceuticals.^[1]^ In particular, LIBs are being extensively used to achieve a reduced dependency of private and other transport systems on fossil fuels to curb global warming by reducing CO_2_ emission, resulting in a rapid growth in the consumption of the earth's lithium resources.^[2]^ Some predictions forecast that the global lithium demand will not be able to be fulfilled by 2023 without recycling^[3]^ which is particularly problematic at present since the global recovery rate of lithium currently does not exceed 1 %.^[4]^ In order to achieve carbon‐neutral and sustainable development goals, exploring efficient strategies for the recognition and recovery of lithium from diverse sources, such as from spent LIBs, brackish brines and seawater, will clearly help to increase the available lithium resources and contribute to alleviating environmental pressures.

**Scheme 1:**
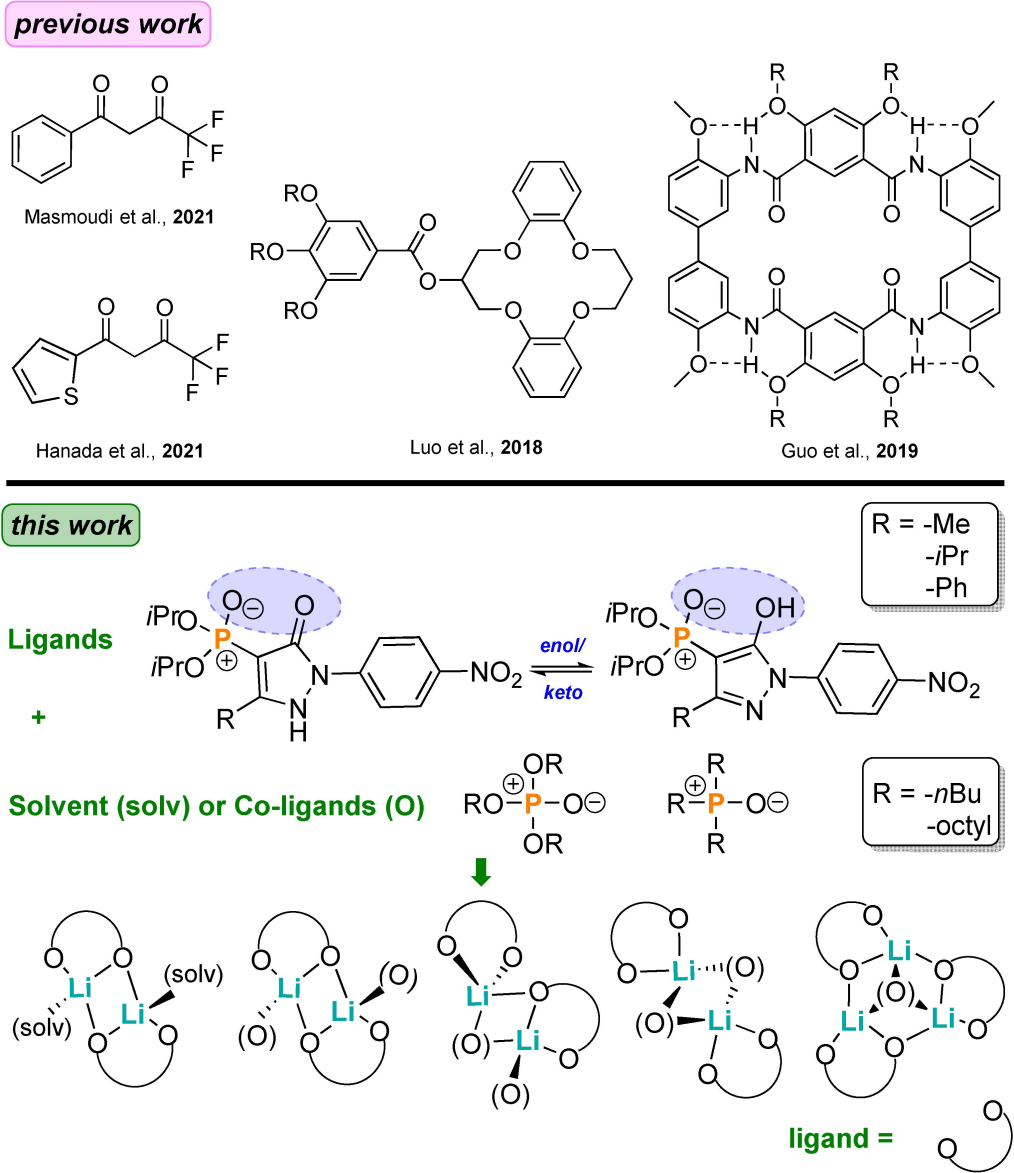
*Previous work*: a selection of ligands employed for the recognition and isolation of Li^+^; *this work*: the 4‐phosphoryl pyrazolone ligands and their observed binding motifs with Li^+^ in the presence of solvent or organophosphorus co‐ligands.

Over recent years, various techniques have been employed for lithium separation, these include liquid‐liquid extraction (LLE),^[5]^ solid‐liquid extraction (SLE),^[6]^ as well as adsorption,^[7]^ membrane^[8]^ and electrochemical processes.^[9]^ Due to its generally large processing capacity, high selectivity, and extraction efficiency, LLE has often been used and is considered to be the most promising process, especially for the separation of lithium from a mixture of other monovalent ions (such as sodium, potassium and cesium ions).^[10]^ However, the selective binding of lithium by specific organic receptors remains challenging and so far only a small variety of such ligand systems have been developed and applied in LLE and SLE (Scheme 1).[^5a^, ^5b^, ^5c^, ^5d^, ^6^, ^11^] Organophosphorus compounds such as di‐(2‐ethylhexyl)phosphoric acid (D2EHPA) and β‐diketones like benzoyl‐1,1,1‐trifluoroacetone (HBTA), 4,4,4‐trifluoro‐1‐(2‐thienyl)‐1,3‐butanedione (HTTA) or α‐acetyl‐m‐dodecylacetophenone (LIX54) have typically been employed for the extraction of Li^+^; however these ligand systems require the presence of a neutral, industrial co‐ligand(s) such as trioctylphosphine oxide (TOPO), mixtures of trialkylphosphine oxides (Cyanex923) or tributyl phosphate (TBP) and the synergistic mechanism of these co‐ligands is still unclear at the molecular level.[^5d^, ^12^] Due to its high hydration energy, Li^+^ tends to be less readily extracted than other alkali metal ions.[^6b^, ^13^] Furthermore, very basic conditions (pH>11) are generally required for extraction from aqueous solution.[^5b^, ^12c^] Macrocyclic receptors, particular crown‐4 derivatives are also selective for lithium ions and have been widely investigated by SLE (Scheme 1).[^5a^, ^6^, ^11b^, ^11d^, ^14^] For these, cavity size plays a vital role in lithium ion recognition. A range of ion‐pair receptors have also been developed in recent decades for metal salt recognition.^[15]^ While certain ditopic receptors have been demonstrated to be capable of extracting lithium salts selectively under SLE conditions, both the loading capacity together with the lithium selectivity tends to be less than ideal largely due to the previously‐mentioned high Li^+^ hydration energy; this hinders their use as successful LLE process reagents.[^5a^, ^6b^, ^11b^] Consequently, the development of more effective ligands that can be employed in SLE or LLE processes for Li^+^ under mild conditions, while challenging, remains an important goal.

Acylpyrazolones, derived from the β‐diketone family, are widely used for the coordination of various metal ions due to their generally strong chelating ability.^[16]^ Despite a considerable number of reports covering the coordination of such receptors for d‐ and f‐block elements,^[17]^ investigations of the coordination of s‐block elements (and especially alkali metals) are rare.^[18]^ We found 4‐phosphoryl pyrazolones can be promising alternatives to acylpyrazolones since they benefit from potentially higher functionality due to the presence of the conveniently adjustable (and versatile) phosphoryl moiety. Recently, we reported the successful employment of a 4‐phosphoryl pyrazolone for coordination of a series of f‐block elements.^[19]^ Based on our findings, we anticipated that this ligand class would make very suitable and selective lithium‐ion receptors.

**Figure 1:**
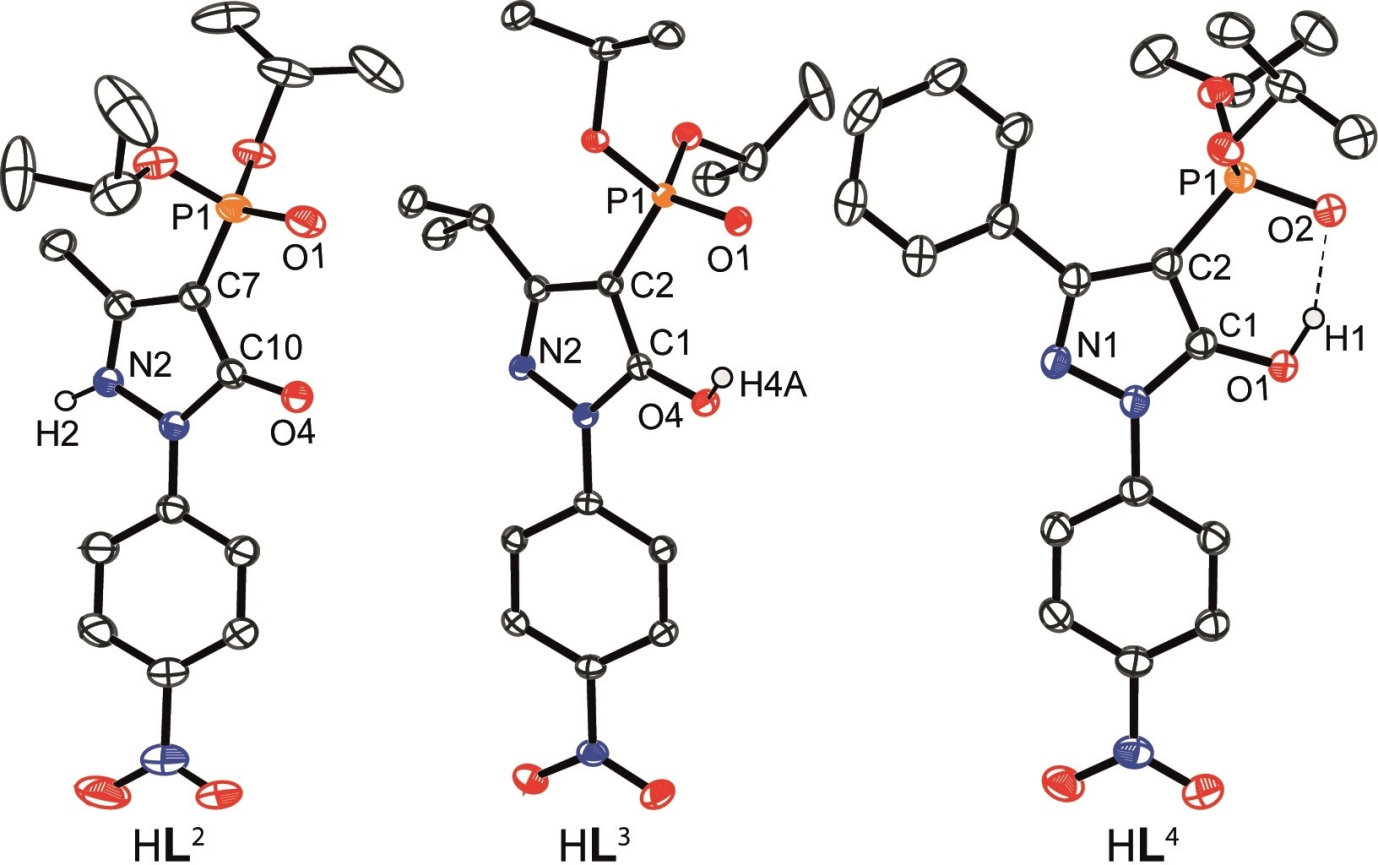
Molecular structures of H**L** ^2^–H**L** ^4^ (all carbon hydrogen atoms are omitted for clarity and ellipsoids are drawn at 50 % probability level).

Herein, we report the synthesis of a series of new 4‐phosphoryl pyrazolone ligands with an adaptable bite size between the chelating O‐donor atoms, which is able to be controlled by variation of the steric demand of the substituents (Figure 1). We studied the coordination behavior of these new ligands towards Li^+^ in the presence of different neutral organophosphorus co‐ligands and also compared the behavior of 1‐(5‐hydroxy‐3‐methyl‐1‐(4‐nitrophenyl)‐1H‐pyrazol‐4‐yl)ethan‐1‐one (H**L** ^1^) towards this ion. X‐ray single crystal structure analyses of the corresponding solid complexes were performed in order to provide a first insight into the coordination modes of the ligands and co‐ligands towards the lithium cation. Detailed NMR studies were also employed to examine complex formation in solution. Further, a series of LLE and SLE experiments were also used to probe the extraction ability as well as the composition of the extracted species. Overall, our results show both a very high extraction efficiency towards Li^+^ as well as high selectivity over Na^+^, K^+^, and Cs^+^ making this ligand class very promising reagents for use in lithium separation processes.

## Results and Discussion

**Scheme 2:**
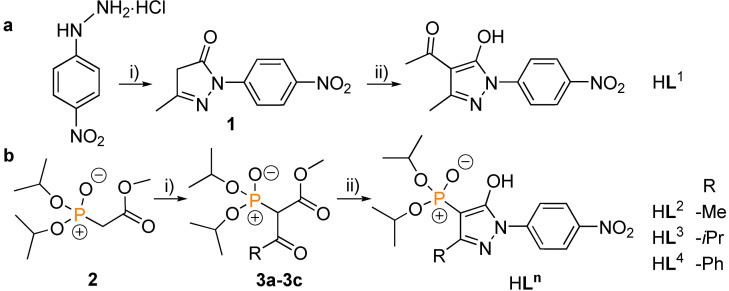
Synthesis of H**L** ^*n*^: a): i) 1.3 equiv. ethyl‐3‐oxobutanoate, AcOH, reflux, 3 h, add Et_2_O then stir 1 h at 0 °C; ii) 2.5 equiv. Ca(OH)_2_, dry 1,4‐dioxane, reflux 0.5 h, add 1.5 equiv. acetyl chloride at 0 °C then reflux 1.5 h. b): i) 1.0 equiv. MgCl_2_, 3.5 equiv. Et_3_N, 1.9 equiv. acetyl/isobutyryl/benzoyl chloride, dry CH_2_Cl_2_, r.t., 3 h; ii) when R=Me, 1.1 equiv. *p*‐NO_2_‐PhNHNH_2_ ⋅ HCl, H_2_O, reflux 3 h, adding 2.0 equiv. K_2_CO_3_, reflux 4 h, then r.t. overnight; when R=*i*Pr, 1.2 equiv. *p*‐NO_2_‐PhNHNH_2_ ⋅ HCl, AcOH, EtOH and H_2_O mixture, reflux 11 h; when R=Ph, 2.5 equiv. *p*‐NO_2_‐PhNHNH_2_ ⋅ HCl, AcOH, EtOH and H_2_O mixture, reflux 50 h.

The acylpyrazolone H**L** ^1^ was synthesized in 85 % yield according to a reported two‐step procedure (Scheme 2a)^[20]^ and the new 4‐phosphoryl pyrazolone ligands H**L** ^2^–H**L** ^4^ were obtained by our recently reported method starting from the phosphorylated acetate **2** ^[19]^ followed by conversion to the β‐dicarbonyl‐functionalized phosphonates **3a**–**3c** (Scheme 2b).^[21]^ Phosphonates **3a**–**3c** were obtained as yellow oily liquids in good to excellent yields (68 %–96 %). They were condensed with 4‐nitrophenylhydrazine hydrochloride using an adoption of reported procedures^[22]^ to yield yellow to brown ligands (H**L** ^2^−H**L** ^4^) in reasonable yields of 31 %–61 %. All ligands were fully characterized^[23]^ including their solid‐state structures. Suitable single crystals for X‐ray analysis were obtained by either slow evaporation of a MeOH‐Et_2_O ligand solution (2 : 1, v/v; H**L** ^2^) or by recrystallization from hot iso‐hexane (H**L** ^3,4^). From the respective structures, the bite size between the chelating O‐donor atoms of the phosphoryl and azole moieties appears of special importance for achieving selective lithium‐ion coordination. Interestingly, H**L** ^2^ crystallized in its *keto*‐form, whereas H**L** ^3,4^ crystallized in their *enol*‐forms (Figure 1). The *keto*/*enol* isomerization of the ligands is reflected in the C−O bond length of the azole unit; for H**L**;^3,4^ this bond is ∼0.09 Å longer than for H**L** ^2^, with a value of 1.233(2) Å which is typical for the *keto*‐form of pyrazolones (Table 1).^[24]^ Disregarding *keto/enol* considerations, the distance between the O‐donor atoms available for chelation decreases significantly from 3.0748(17) Å (H**L** ^2^) to 2.5614(12) Å (H**L** ^4^), indicating a more acute bite angle for H**L** ^4^ which is in line with the increase in the steric demand of the substituent on the pyrazolone moiety of the respective ligands. Compared to classical β‐diketones in their *enol*‐form, such as benzoyl‐1,1,1‐trifluoroacetone (HBTA) (O⋅⋅⋅O: 2.5236(13) Å)^[25]^ or acylpyrazolones, such as 4‐acyl‐5‐methyl‐2‐phenylpyrazolones (O⋅⋅⋅O: acyl=4‐propionyl 2.5327(14) Å; acyl=4‐butanoyl 2.583(5) Å; and acyl=4‐cinnamoyl 2.558(2) Å),^[26]^ a higher flexibility of the O⋅⋅⋅O distance (bite size) in the present ligands is evident which, as indicated above, was anticipated to affect the complexation (and extraction performance) of the respective ligands. In order to derive reliable distances between the chelating O‐donor atoms, we deprotonated ligands H**L** ^2–4^ with tetrabutylammonium hydroxide ([TBA][OH]) to form the corresponding ammonium salts [TBA]**L** ^2–4^ (Figure S61).^[23]^ For these alkyl‐substituted [TBA]**L** ^2,3^ salts, the O⋅⋅⋅O distance is increased to 3.2233(12) and 3.232(2) Å, respectively, which is significantly larger than occurs for the [TBA]**L** ^4^ salt (3.0816(12) Å). This appears to reflect the steric demand and orientation of the respective substituents (Me, *i*Pr, Ph) leading to a progressively more acute bite size for H**L** ^4^ (Table 1).

**Table 1:** Selected bond lengths and O⋅⋅⋅O distances [Å] for the pairs of chelating O‐donor atoms in H**L** ^2^, H**L** ^3^, H**L** ^4^.

Entry	H**L** ^2^/[TBA]**L** ^2^	H**L** ^3^/[TBA]**L** ^3^	H**L** ^4^/[TBA]**L** ^4^
C−O^[a]^	1.233(2)/ 1.2470(15)	1.3233(13)/1.239(3)	1.3269(14)/1.2427(14)
O_P_⋅⋅⋅O_C_^[b]^	3.0748(17)/3.2233(12)	3.0109(11)/3.232(2)	2.5614(12)/ 3.0816 (12)

[a] C−O bond lengths in the pyrazol‐5‐olate unit; [b] separation distance between the chelating O‐donor atoms.

**Figure 2:**
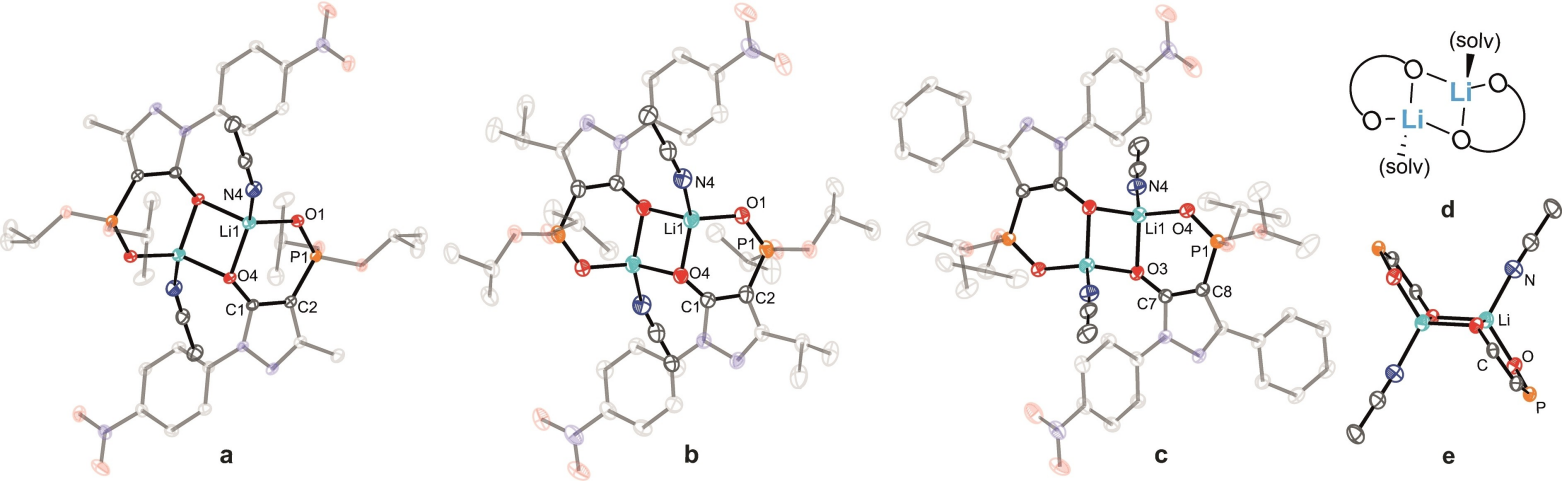
Molecular structures of a) [Li_2_(**L** ^2^)_2_(CH_3_CN)_2_] (**4**), b) [Li_2_(**L** ^3^)_2_(CH_3_CN)_2_] (**5**), c) [Li_2_(**L** ^4^)_2_(CH_3_CN)_2_] (**6**), d) schematic illustration of their coordination mode, e) side view of the lithium coordination of the core in **6** (hydrogen atoms are omitted for clarity and ellipsoids are drawn at 50 % probability level).

Lithium complexes are known to adopt a wide range of structural motifs, both in solution and in the solid state,^[27]^ with the structures often influenced by solvation effects.[^27a^, ^28^] Single crystals suitable for X‐ray investigation of the acetonitrile‐solvated dimer complexes [Li_2_(**L** ^2^)_2_(CH_3_CN)_2_] (**4**), [Li_2_(**L** ^3^)_2_(CH_3_CN)_2_] (**5**), and [Li_2_(**L** ^4^)_2_(CH_3_CN)_2_] (**6**) were obtained by slowly diffusing dry Et_2_O into a solution of the respective crude Li^+^ complexes dissolved in CH_3_CN at −30 °C in the glove‐box.^[23]^ The structures obtained are depicted in Figure 2. In all three structures, each Li^+^ is coordinated by three oxygen atoms from two ligands and a nitrogen donor from CH_3_CN to give each lithium center a distorted tetrahedral geometry. One oxygen donor from each pyrazolone unit bridges both Li^+^ centers to yield a planar (LiO)_2_ four‐membered ring. Each six‐membered (Li⋅⋅⋅O−P−C−C−O) chelate ring is slightly twisted and forms part of a step‐ladder type arrangement (Figure 2e). The Li⋅⋅⋅O interactions involving the phosphoryl units are slightly shorter (1.870(4)–1.905(4) Å) than those to the pyrazolone oxygen atoms (1.935(2)–1.986(4) Å) (Table 2). All Li⋅⋅⋅O contacts are in the typical range observed for other binuclear Li^+^ complexes.[^27b^, ^29^] In contrast, [Li(**L** ^1^)(EtOH)_2_] (**7**) is mononuclear when crystallized from EtOH, with two solvent molecules completing the tetrahedral coordination geometry of the lithium center (Figure S62).

**Table 2:** Summary of relevant Li⋅⋅⋅Li, Li⋅⋅⋅O(N)‐donor atom contacts and O⋅⋅⋅O distances (in Å) for the binuclear and trinuclear complexes **4**–**13**.

Entry	Li⋅⋅⋅O_P_^[a]^	Li⋅⋅⋅O_C_^[b]^	Li⋅⋅⋅O(N)_Co (solv)_^[c]^	Li⋅⋅⋅Li	O_P_⋅⋅⋅O_C_
**4**	1.898(2)	1.935(2) 1.969(2)^[i]^	2.073(2)	2.710(4)	3.0025(13)
**5**	1.905(4)	1.951(4) 1.986(4)^[i]^	2.092(4)	2.739(7)	3.023(2)
**6**	1.870(4)	1.948(3) 1.938(3)^[i]^	2.038(4)	2.562(6)	2.8934(18)
**8**	–	1.915(2) 1.936(2)^[d]^	1.938(2) 1.940(2)^[ii]^	2.707(4)	2.8552(13)
**9**	1.907(3) 1.909(3)	1.925(3) 1.967(3)	1.925(3) 1.903(3) 1.933(3)	2.704(4)	2.9666(17) 2.9602(17)
**10**	1.954(11)	1.902(10)	1.955(9) 1.979(9)^[i]^	2.723(17)	2.906(6)
**11**	1.917(4)	1.938(4) 1.956(4)^[iii]^	1.904(4)	2.645(8)	2.964(2)
**12**	1.995(4) 1.958(3)^[iv]^	1.852(4)	1.978(4)	2.661(6)	2.9722(18)
**13**	1.979(2) 1.941(2)^[vi]^	1.838(2)	1.990(2)	2.654(4)	2.9319(13)

oxygen atom of the [a] phosphoryl motif; [b] the pyrazolone unit; [c] co‐ligand (or nitrogen atom); [d] acyl group. Symmetry operation [i] 1−*x*,1−*y*,1−*z*; [ii] 1−*x*, −*y*,1−*z*; [iii] −*x*,1−*y*,1−*z*; [iv] 1+*y*−*x*,1−*x*,+*z*; [vi] +*y*−*x*,1−*x*,+*z*.

**Figure 3:**
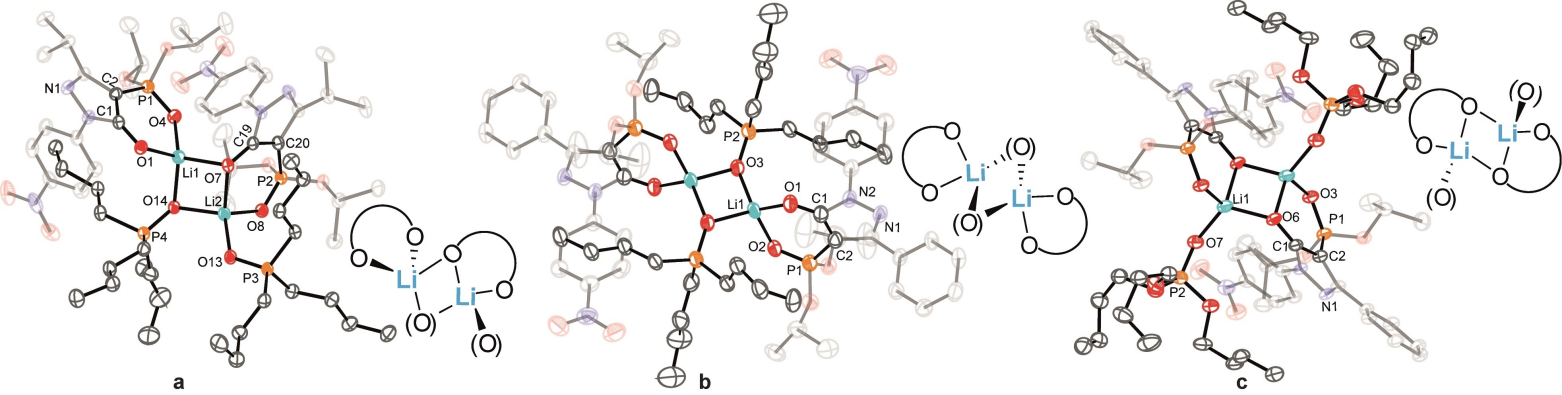
Molecular structures of a) [Li_2_(**L** ^3^)_2_(TBPO)_2_] (**9**), b) [Li_2_(**L** ^4^)_2_(TBPO)_2_] ⋅ 2CH_2_Cl_2_ (**10**), c) [Li_2_(**L** ^4^)_2_(TBP)_2_] (**11**) including a schematic drawing of their coordination mode (hydrogen atoms and CH_2_Cl_2_ solvent are omitted for clarity and ellipsoids are drawn at 50 % probability level).

**Figure 4:**
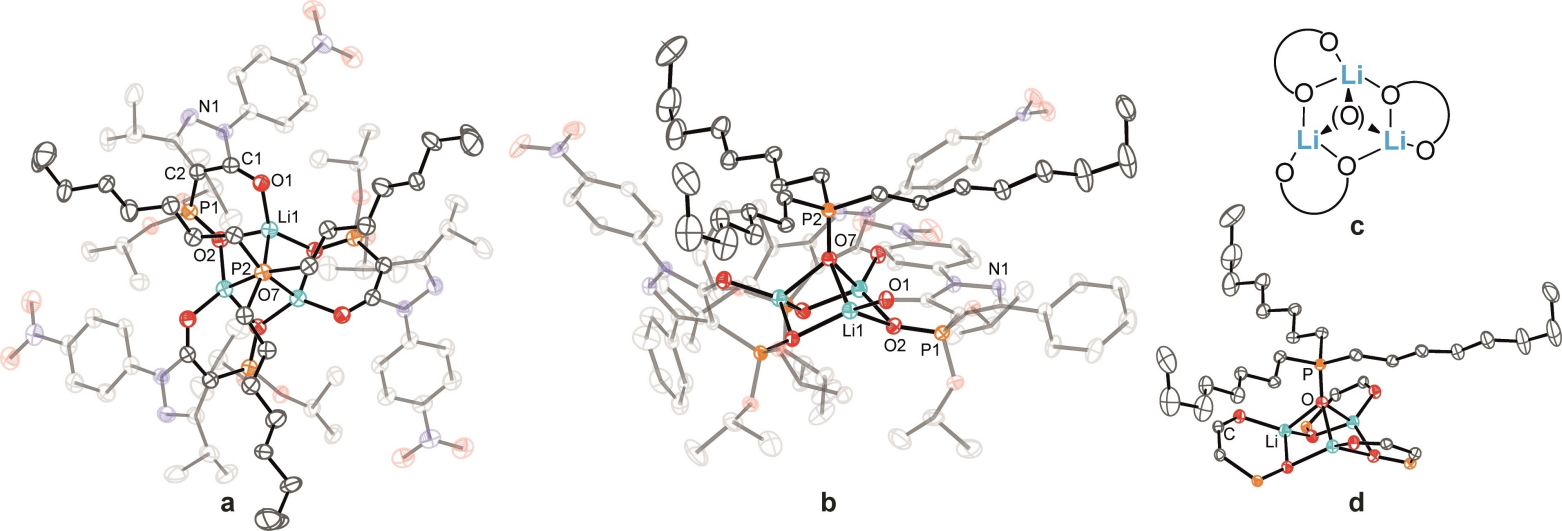
Molecular structures of a) [Li_3_(**L** ^3^)_3_(TOPO)] ⋅ 0.65 C_5_H_12_ (**12**), b) [Li_3_(**L** ^4^)_3_(TOPO)] ⋅ 0.67 C_5_H_12_ (**13**), c) schematic representation of the coordination mode of the trinuclear complex, d) view of the lithium coordination in the core of **13** (hydrogen atoms and pentane solvent are omitted for clarity and ellipsoids are drawn at 50 % probability level).

Motivated by the isolation of the above solvent‐coordinated dimeric structures, we investigated the prospect of replacing the coordinated solvents with the neutral organophosphorus co‐ligands, tributylphosphine oxide (TBPO), tributyl phosphate (TBP) and trioctylphosphine oxide (TOPO) ‐ reagents that are extensively employed in separation science to obtain the molecular level understanding of their roles.[^5c^, ^5d^, ^11a^, ^11c^, ^12^] Complexes incorporating TBPO and TBP co‐ligands were obtained from the reaction of H**L** ^1,3,4^ and the respective co‐ligand with LiOH ⋅ H_2_O in CH_2_Cl_2_.^[23]^ Single crystals of [Li_2_(**L** ^1^)_2_(TBPO)_2_] (**8**), [Li_2_(**L** ^3^)_2_(TBPO)_2_] (**9**), [Li_2_(**L** ^4^)_2_(TBPO)_2_] ⋅ 2CH_2_Cl_2_ (**10**) and [Li_2_(**L** ^4^)_2_(TBP)_2_] (**11**) suitable for X‐ray analyses were obtained by slow diffusion of pentane into a saturated CH_2_Cl_2_ solution of the respective crude products (Figures 3 and S63).^[23]^ In all cases, a 2 : 2 : 2 (Li^+^ : [**L** ^n^]^−^: TBPO/TBP) stoichiometry was obtained. The TBPO‐containing complexes **8** (Figure S63) and **9** (Figure 3b) both adopt a coordination mode with bridging TBPO oxygen atoms between the two Li cations. Complex **9** is non‐symmetric, incorporating one bridging TBPO co‐ligand and one non‐bridging TBPO co‐ligand, each coordinated via their O‐donor to Li2 (Figure 3a). This arrangement corresponds to a ‘transition structure’ with respect to the symmetric complex **11** (Figure 3c) in which both TBP co‐ligands now coordinate to the Li ions, similar to the situation occurring in complexes **4**–**6**. The Li^+^ centers in all complexes display distorted tetrahedral LiO_4_ coordination, with a four‐membered [LiO]_2_‐core present in each complex. Slightly shorter Li⋅⋅⋅O contacts involving the phosphoryl units (Li⋅⋅⋅O_P_; 1.907(3), 1.909(3) Å) compared to the pyrazolone oxygen atoms (Li⋅⋅⋅O_C_, 1.925(3), 1.967(3) Å) are observed in **9** (Table 2). The situation slightly changes for the symmetric complex **10** in which a shorter Li⋅⋅⋅O_C_ contact (1.902(10) Å) compared to the Li⋅⋅⋅O_P_ contact (1.954(11) Å) is observed. The Li⋅⋅⋅Li distances in complexes **8**–**10** range from 2.704(4) to 2.723(17) Å and are thus comparable; however, this distance is significantly shorter in **11** at 2.645(8) Å. For complex **11** (Figure 3c) marginally longer Li⋅⋅⋅O_P_ (∼0.05 Å) and Li⋅⋅⋅O_C_ (∼0.01 Å) contacts were observed compared to the CH_3_CN‐containing complex counterparts (Table 2). The bite sizes in **9**–**11**, reflected by the O_P_⋅⋅⋅O_C_ distances, range from 2.906(6) to 2.9666(17) Å and are significantly shortened compared to the respective deprotonated ligands in the salts [TBA]**L** ^2–4^ (Table 1). Reaction of H**L** ^3,4^ with LiOH ⋅ H_2_O in CH_2_Cl_2_ in the presence of TOPO resulted in a completely different coordination mode to those present in **4**–**6**. After recrystallization of the crude product,^[23]^ single crystals were obtained with composition [Li_3_(**L** ^3^)_3_(TOPO)] ⋅ 0.65 C_5_H_12_ (**12**) and [Li_3_(**L** ^4^)_3_(TOPO)] ⋅ 0.67 C_5_H_12_ (**13**) whose X‐ray structures are presented in Figure 4. Both complexes contain a trinuclear complex core with strongly distorted tetrahedral coordinated Li‐centers, with each O_4_‐donor set composed of three deprotonated H**L** ^3,4^ chelating ligands and one oxygen from a TOPO co‐ligand (Figure 4). The three Li centers are capped via μ_3_‐coordination of the TOPO O‐donor atom. The Li⋅⋅⋅O contacts are 1.978(4) Å (**12**) and 1.990(2) Å (**13**), respectively, resulting in a trigonal‐pyramidal [Li_3_O] arrangement which is, to the best of our knowledge, an unprecedented structural motif for trinuclear lithium complexes.^[27a]^ Compared to the TBPO‐containing complexes **9** and **10**, the Li⋅⋅⋅O_C_ contacts are shortened by approximately 0.06 Å, and Li⋅⋅⋅O_P_ interactions are in part elongated up to 1.995(4) Å (Table 2) as a result of the bifurcated coordination to two Li cations in the trinuclear complexes. The observed Li⋅⋅⋅Li distances are 2.661(6) Å (**12**) and 2.654(4) Å (**13**), similar to those in our dimers but are significantly shorter than those in related cyclic trinuclear complexes for which values in a range from 2.8221(5) Å to 3.3372(6) Å have been reported.^[30]^ We note that our Li^+^ complexes incorporating neutral organophosphorus co‐ligands collectively represent a completely new complex category that we suggest offers a promising starting point for the further design (and understanding) of new lithium separation processes and applications.

**Figure 5:**
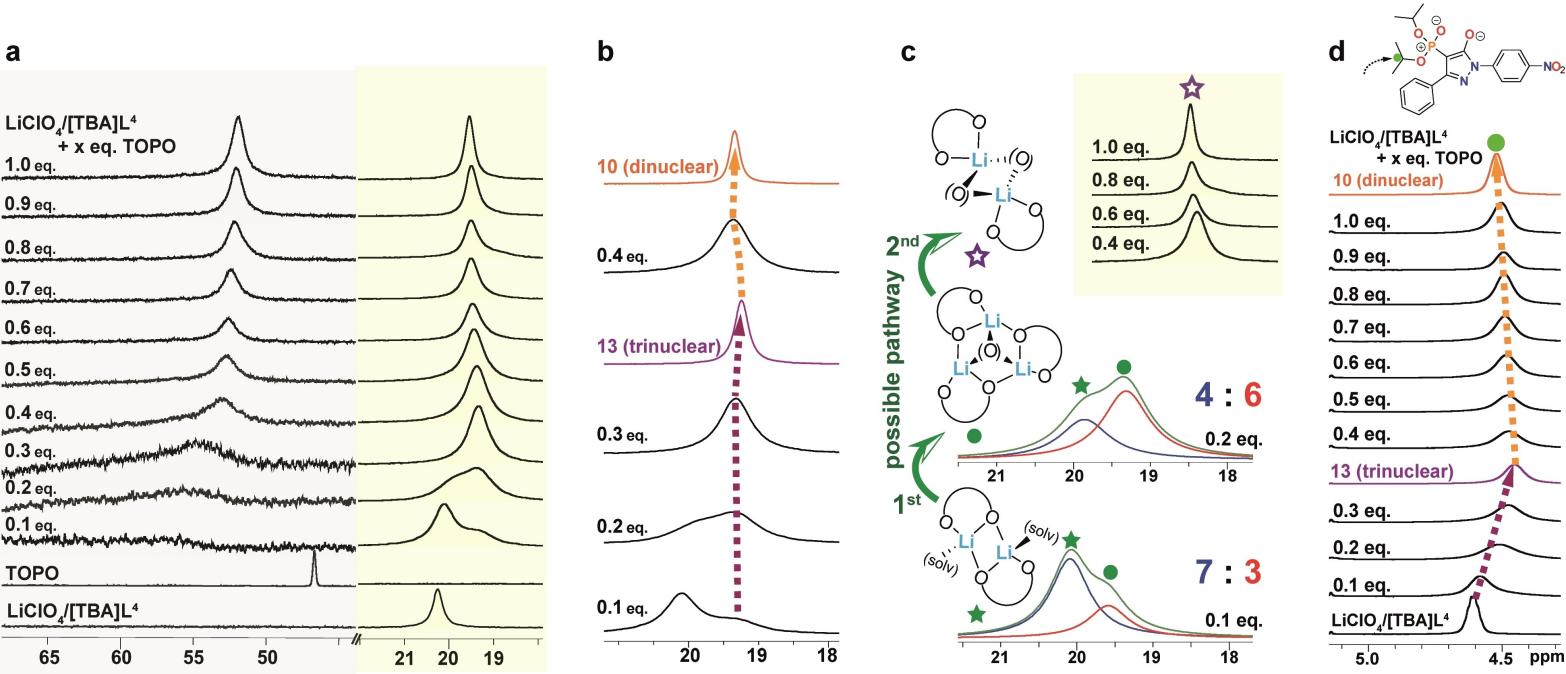
a) Stacked (cut‐out) of ^31^P NMR spectra corresponding to the step‐wise titration of LiClO_4_/[TBA]**L** ^4^ solution with TOPO; b) comparison of ^31^P NMR spectra of up to 0.4 equiv. TOPO titration progress to **13** (purple) then **10** (orange); c) deconvolution analysis for 0.1 equiv. and 0.2 equiv. of added TOPO with the proposed rearrangement pathways (blue and red: fitted lines, green: ‘envelope’ line); d) stacked cut‐out of the ^1^H NMR spectra of the step‐wise titration (methine proton of the *i*Pr groups) compared to **13** and **10** (CD_2_Cl_2_, 300 K).

**Figure 6:**
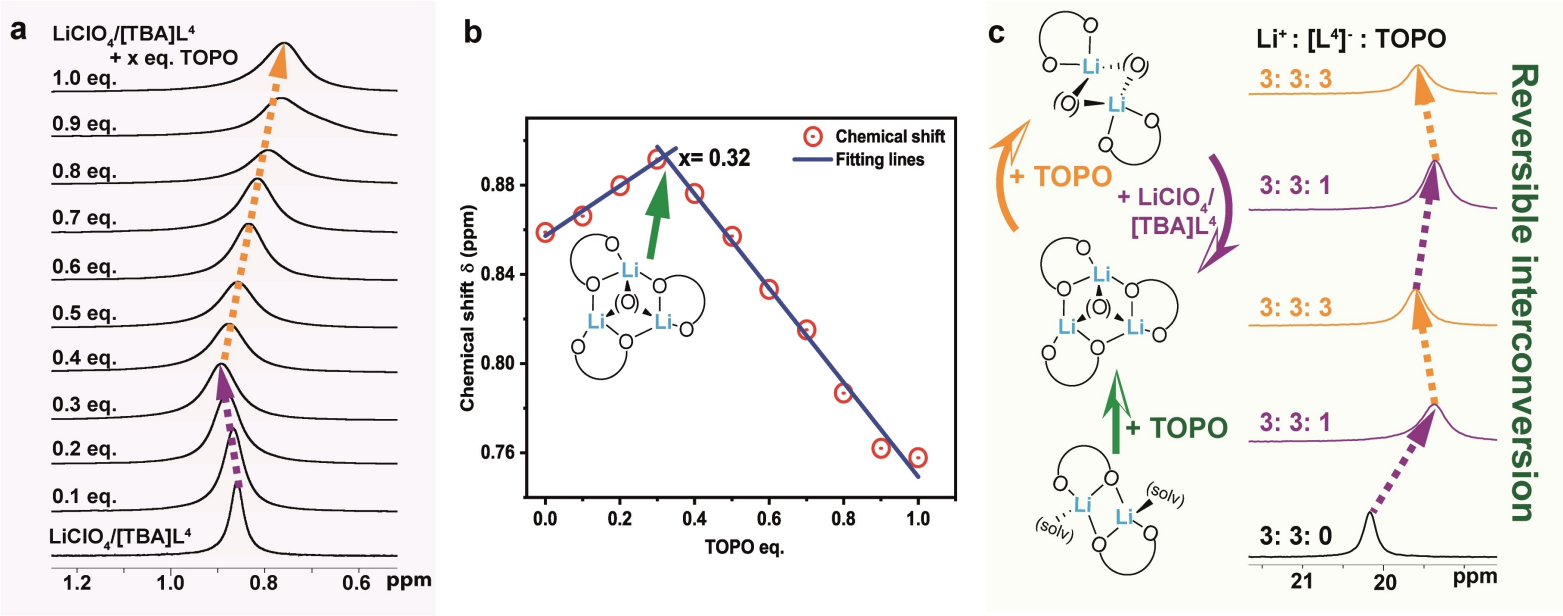
a) Stacked cut‐out of the ^7^Li NMR spectra for the step‐wise titration; b) ^7^Li NMR chemical shift ‐ [TOPO] : [LiClO_4_/[TBA]**L** ^4^] plot (CD_2_Cl_2_, 300 K); c) cut‐out of ^31^P NMR spectra of different mole ratios of LiClO_4_/[TBA]**L** ^4^ : TOPO.

Since the bite‐size of ligand [**L** ^4^]^−^ in [TBA]**L** ^4^ is the smallest, we investigated the speciation chemistry of this salt following its reaction with LiClO_4_ and co‐ligand TOPO through extensive NMR (^31^P, ^1^H and ^7^Li) studies. The latter included several concentration‐dependent procedures such as the continuous variation,^[31]^ mole ratio^[32]^ and slope ratio^[33]^ methods in order to shed light on the lithium complex composition and its dynamic behavior in solution. The continuous variation method turned out to be not suitable for this system due to the required large excess of TOPO and the resulting generation of a number of species (Figure S64).^[23]^ The mole ratio method proved much more suitable and thus was used instead. In a typical experiment, we gradually increased the concentration of TOPO in steps of 0.1 equiv. in a 7.2 mM solution of LiClO_4_/[TBA]**L** ^4^ in CD_2_Cl_2_ at 300 K (Table S2) and recorded the corresponding ^31^P, ^1^H and ^7^Li NMR spectra (Figure 5a–d; the ^31^P and ^7^Li chemical shifts are summarized in Table S3).^[23]^ For reference, Figure 5a (bottom) shows the respective singlet resonances of the mixture of LiClO_4_/[TBA]**L** ^4^ (*δ*=20.2 ppm) and co‐ligand TOPO (*δ*=46.7 ppm) dissolved in CD_2_Cl_2_. We assume that the 1 : 1 LiClO_4_/[TBA]**L** ^4^ mixture consists of the dimeric Li‐complex [Li_2_(**L** ^4^)_2_(solv)_2_], similar to the investigated complexes **4**–**6**. Upon addition of TOPO, the resonances attributed to coordinated TOPO, that are expected to be observed between *δ*=50–60 ppm, are extremely broad until the proportion of the ligand reaches 0.4 equiv., indicating a dynamic exchange process. Interestingly, the resonance that is attributed to the coordinated ligand [**L** ^4^]^−^ (*δ*=20.2 ppm) upon addition of 0.1 equiv. TOPO is slightly displaced by a high‐field shifted shoulder indicating the formation of a new complex species. The latter becomes the dominant species when the proportion of the co‐ligand reaches ∼0.3 equiv. (*δ*=19.3 ppm; *ν*_1/2_=112 Hz). With increasing concentration of TOPO, this resonance shifts back slightly to lower‐field with a much sharper resonance at *δ*=19.5 ppm (*ν*_1/2_=51 Hz). The same sharpening is observed for the resonance attributed to the co‐ligand (*δ*=52.1 ppm; *ν*_1/2_=154 Hz) until 1.0 equiv. is reached. The change in resonance in the ^31^P NMR spectra for [**L** ^4^]^−^ upon addition of TOPO is consistent with the occurrence of different species in solution. A deconvolution analysis of these phosphorus signals was performed using the deconvolution function of the commercially available topspin software designed primarily to analyze high‐resolution small molecule NMR data (Figure 5c).[^23^, ^34^] The observed changes of these overlapping resonances were analyzed for the 0.1 equiv. and 0.2 equiv. TOPO cases and showed that for 0.1 equiv. TOPO an integration ratio of 7 : 3 (Table S4) was present. This indicates that the new resonance involves 0.3 equiv. ligand as well as 0.1 equiv. TOPO. Upon the addition of 0.2 equiv. TOPO, this ratio increased to 4 : 6 (Table S5), consistent with the involvement of 0.6 equiv. of ligand and 0.2 equiv. of co‐ligand TOPO. As already discussed, for a proportion of the co‐ligand of ∼0.3 equiv. the corresponding singlet resonance occurs between *δ*=19.1–19.3 ppm which is consistent with the Li^+^ : [**L** ^4^]^−^ : [TOPO]=3 : 3 : 1 complex, [Li_3_(**L** ^4^)_3_(TOPO)] (**13**; *δ*=19.2 ppm), occurring to represent the first rearrangement step from [Li_2_(**L** ^4^)_2_(solv)_2_] as illustrated in Figure 5c. For comparison, a cut‐out of the ^31^P NMR spectra corresponding to 0.1 to 0.4 equiv. TOPO as well as for the trinuclear complex **13** in purple and, for reference, [Li_2_(**L** ^4^)_2_(TBPO)_2_] (**10**) in orange are also shown (Figure 5b). The latter complex differs only in the alkyl chain‐length of the TBPO (butyl) versus TOPO (octyl) and thus, the chemical shifts for both dimers are comparable. Therefore, we assume that with further increase in the TOPO concentration, the 3 : 3 : 1 complex [Li_3_(**L** ^4^)_3_(TOPO)] (**13**) converts to the dimeric complex [Li_2_(**L** ^4^)_2_(TOPO)_2_] (as indicated in the second rearrangement step shown in Figure 5c). Additional evidence, but less indicative, for the assumed stepwise rearrangement from [Li_2_(**L** ^4^)_2_(solv)_2_]→[Li_3_(**L** ^4^)_3_(TOPO)] (**13**)→[Li_2_(**L** ^4^)_2_(TOPO)_2_] is observed in the ^1^H NMR (methine proton resonance of the *i*Pr groups) shown in Figure 5d, with the full spectra presented in Figure S65 as well as by the ^7^Li NMR spectra (Figure 6a). The respective resonances are significantly broadened preventing a similar deconvolution analysis to that employed for the ^31^P NMR spectra; however, the same shift trends are observed and are comparable to those of the trinuclear complex **13** (referenced to [Li_2_(**L** ^4^)_2_(TBPO)_2_] (**10**)) (Figure 5d).

A significant “turning” of the resonances when the proportion of the co‐ligand reaches values >0.3 equiv. is nicely shown in Figure 6a. The conversion of [Li_3_(**L** ^4^)_3_(TOPO)] (**13**)→[Li_2_(**L** ^4^)_2_(TOPO)_2_] as depicted in Figure 5c is further demonstrated by a *δ*‐[TOPO] : [LiClO_4_/[TBA]**L** ^4^] plot of the ^7^Li NMR spectra which gives rise to two straight lines with an intersection at 0.32 equiv., consistent with the expected Li^+^ : [**L** ^4^]^−^ : [TOPO] ratio of 3 : 3 : 1 for the composition of complex **13** (Figure 6b). In addition, the interconversion between these complexes is reversible as shown by the respective ^31^P NMR spectra following the alternating stoichiometric additions of LiClO_4_/[TBA]**L** ^4^ and TOPO and, in particular, the observed resonance shifts that are attributed to the presence of the binuclear (*δ*=19.5 ppm; 2 : 2 : 2) and trinuclear (*δ* =19.3 ppm; 3 : 3 : 1) species in solution (Figure 6c).

**Figure 7:**
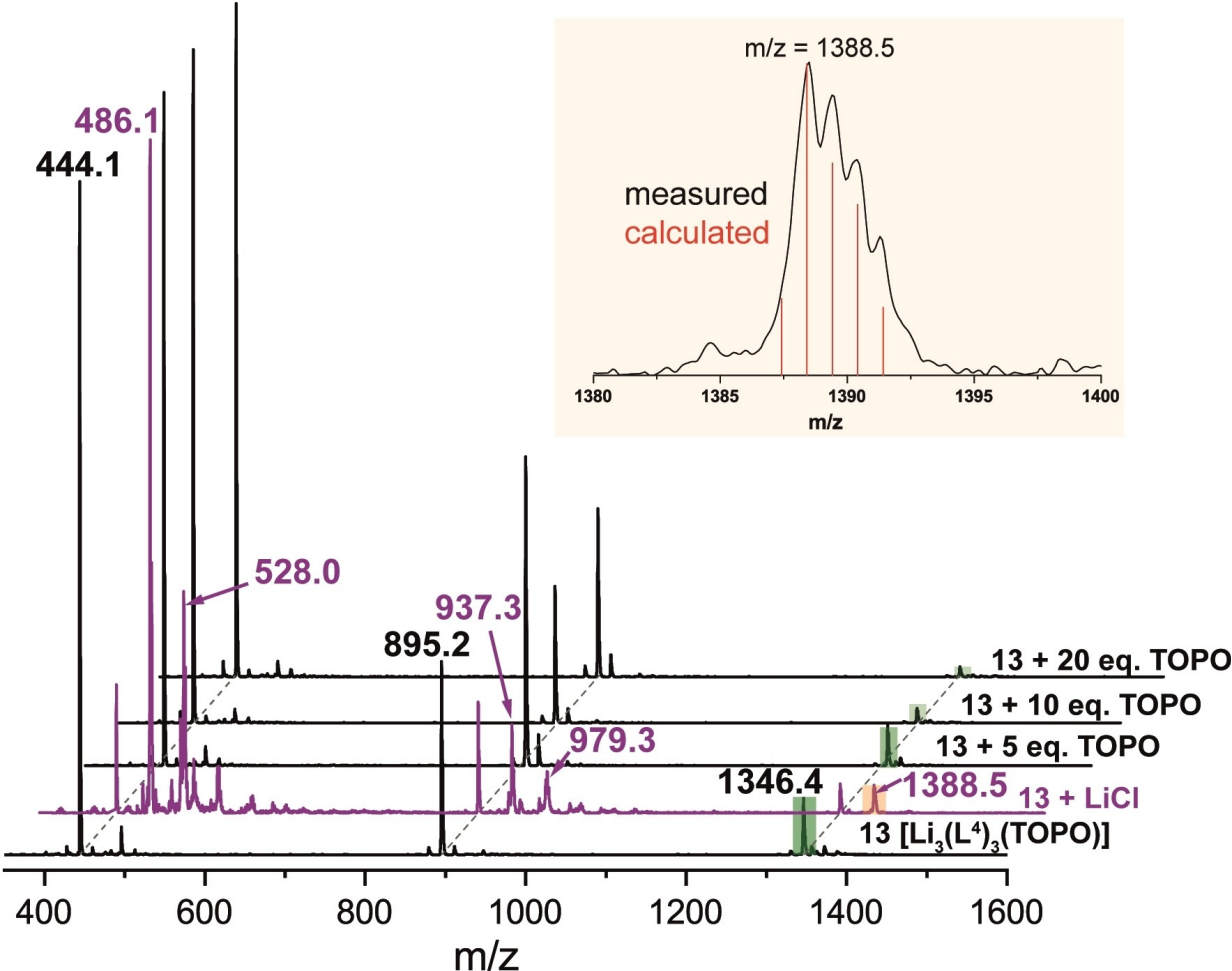
ESI‐MS (negative‐ion mode) of **13** complex in CH_3_OH; on addition of excess LiCl (purple); on addition of extra 5–20 equiv. TOPO; Top:peak at *m*/*z*=1388.5 with isotope pattern superimposed.

The speciation behavior of the Li^+^ complexes in solution was further investigated by obtaining the electrospray ionization mass spectra (ESI‐MS)^[35]^ of **12** and **13** dissolved in both neat methanol and in LiCl/methanol solution (Figures 7 and S66).^[23]^ The attributed peaks were assigned according to their simulated isotope patterns, as well as from recorded daughter experiments (Table 3, Figures S67–S70). For both complexes comparable peaks were observed, which can be attributed to the presence of similar complex species (including trinuclear species).

**Table 3:** Summary of observed peaks and their assignments for **12** and **13** in the absence and presence of excess LiCl.

Observed peaks				Assignment^[a]^
**12**	**12**+LiCl	**13**	**13**+LiCl	
410.2	410.2	444.1	444.1	[**L** ^*n*^]^−^
–	452.1	–	486.1	[Li ⋅ **L** ^*n*^ ⋅ Cl]^−^
–	494.1	–	528.0	[Li_2_ ⋅ **L** ^*n*^ ⋅ Cl_2_]^−^
827.2	827.2	895.2	895.2	[Li ⋅ **L** ^*n*^_2_]^−^
–	869.3	–	937.3	[Li_2_ ⋅ **L** ^*n*^_2_ ⋅ Cl]^−^
–	911.3	–	979.3	[Li_3_ ⋅ **L** ^*n*^_2_ ⋅ Cl_2_]^−^
1244.4	1244.4	1346.4	1346.4	[Li_2_ ⋅ **L** ^*n*^_3_]^−^
–	1286.5	–	1388.5	[Li_3_ ⋅ **L** ^*n*^_3_ ⋅ Cl]^−^

[a] *n*=3 in **12** and *n*=4 in **13**.

In subsequent experiments, the ESI‐MS spectra of **13** in the presence of a varying excess of TOPO were recorded (Figure 7).^[23]^ With increasing TOPO concentration (5–20 equiv.), a significant decrease in the relative intensity of the *m*/*z*=1346.4 peak, attributed to the species [Li_2_ ⋅ **L** ^4^_3_]^−^, most likely originated form the trinuclear species [Li_3_ ⋅ **L** ^4^_3_], was observed, whereas the relative intensity of the peak at *m*/*z*=895.2, attributed to [Li ⋅ **L** ^4^_2_]^−^, remains the dominant peak. This observation indicates that an excess of TOPO facilitates the conversion of the trinuclear species into other species, which is in line with the proposed behavior observed in the NMR experiments.

**Figure 8:**
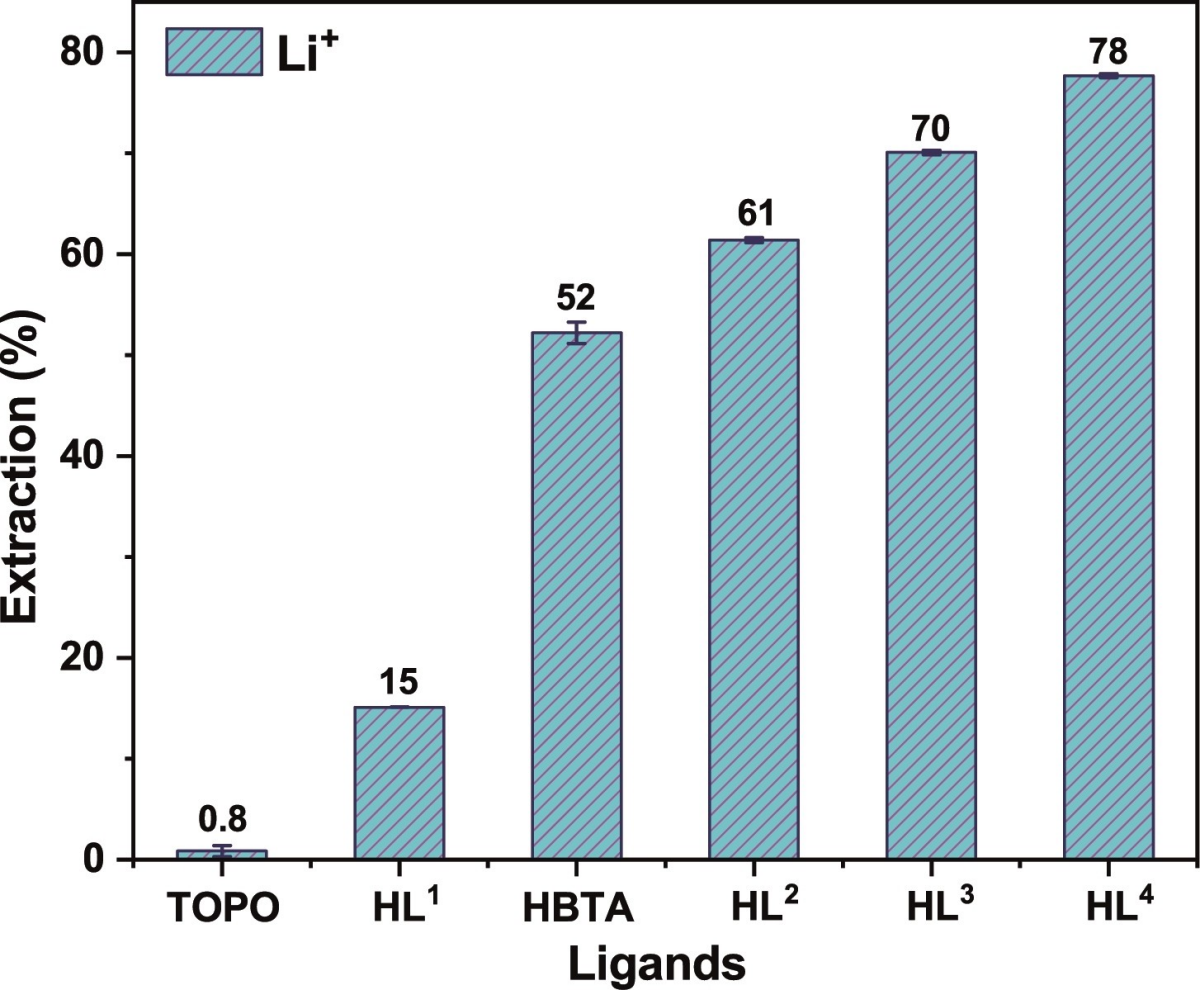
Percent extraction of Li^+^ with different ligands in the presence of TOPO. Conditions: [LiCl]=0.01 M, [NH_4_Cl]=0.1 M, pH=8.2 (Tris/HCl buffer), [H**L**]=0.1 M, [TOPO]=0.2 M in CHCl_3_, 298 K, 1 h.

To investigate the ability of H**L** ^2–4^ to extract Li^+^, LLE experiments were performed in the presence and absence of TOPO (Figures 8 and S71) at pH 8.2. For comparison, the acylpyrazolone ligand H**L** ^1^ as well as the widely employed HBTA reagent were also included in these studies. The Li^+^ extraction for all ligands in the absence of TOPO was essentially negligible (<5%, Figure S71). In contrast, in the presence of TOPO, Li^+^ extraction of 78 % was observed for H**L** ^4^ followed by 70 % for H**L** ^3^ and 61 % for H**L** ^2^. These three 4‐phosphoryl pyrazolone ligands clearly outperform the acylpyrazolone ligand H**L** ^1^ (15 % extraction) and HBTA (52 % extraction) under similar conditions.

As mentioned above, the slope ratio method is also an important strategy for use in solution studies. Herein, slope analyses of the respective extraction data using log*D*‐log*c*_**L**(org)_ diagrams were performed (Figure S72) in order to investigate the composition of the extracted species corresponding to H**L** ^3^, H**L** ^4^ and HBTA in the organic phases.^[33]^ For this, extraction experiments that involved variation of the ligand and co‐ligand in excess TOPO were performed using aqueous solutions of 0.01 M LiCl (see Eqs. (S3)–(S5) in Section S9).^[23]^ For HBTA a determined slope of approximately 1 in the log*D*_Li_‐log[H**L**]_(org)_ diagram and of approximately 2 in the log*D*_Li_‐log[TOPO]_(org)_ diagram are in accordance with the formation of a 1 : 1 : 2 (Li^+^ : [BTA]^−^ : TOPO) complex. Furthermore, this result is consistent with the reported results for the similar extractant HTTA, also employed for the separation of lithium.^[12b]^ In contrast, slopes of approximately 1 were obtained for H**L** ^3^ and H**L** ^4^ in both diagrams and indicate extraction with the formation of complexes of 1 : 1 : 1 (Li^+^ : [**L** ^*n*^]^−^ : TOPO) stoichiometry, presumably corresponding to an actual composition of 2 : 2 : 2 (Li^+^ : [**L** ^*n*^]^−^ : TOPO), comparable to that observed in the solid state for the complexes incorporating co‐ligands TBPO and TBP. These results are in line with those obtained from the NMR mole ratio method and the ESI‐MS results, which further reinforce that the binuclear motif is the dominant species in the presence of a high excess of TOPO under the conditions employed for the LLE.

In order to probe if a single complex is formed in the organic phase under ‘saturation’ conditions (corresponding to a constant co‐ligand concentration) in the presence of excess metal, a second set of LLE experiments was performed.^[36]^ These studies can reveal the composition of the extracted species when the co‐ligands’ presence is in shortfall under LLE conditions. The experimental data are shown in Figures S73 and S74 in Section S10 and the results are summarized in Table 4. When TOPO was employed as co‐ligand, from the extraction data an approximate [TOPO] : [metal+ligand] ratio of 1 : 3 was determined, indicating that both ligands can extract Li^+^ by the formation of a complex with 0.33 equiv. TOPO. This result is consistent with the stoichiometry present in the corresponding X‐ray structure (3 : 3 : 1 (Li^+^ : [**L** ^*n*^]^−^ : TOPO)) and that obtained by the NMR mole ratio method in the presence of a shortfall of TOPO. In contrast, the studies employing TBPO as co‐ligand reveal inflection points at approximately 1 (Table 4), in keeping with a stoichiometry of the extracted species of 1 : 1 : 1 (Li^+^ : [**L** ^*n*^]^−^ : TBPO), most likely of type [Li_2_(**L** ^*n*^)_2_(TBPO)_2_], which is consistent with the ratio observed in the corresponding X‐ray structures. For HBTA, an inflection at 1.88 was obtained (Figure S73c, Table 4), indicating a stoichiometry for the extracted species of 1 : 1 : 2 (Li^+^ : [BTA]^−^ : TOPO), which parallels the result of the extraction studies as well as for the reported behavior of HTTA in Li^+^ extraction.^[12b]^

**Table 4:** Maximum co‐ligand : Li^+^/ligand ratios for the organic phases obtained from loading experiments involving a fixed concentration of ligand and Li^+^ and varying co‐ligand concentration (see text).

System	H**L** ^3^	H**L** ^4^	HBTA
TOPO (eq.) Li^+^ : [**L** ^n^]^−^ : TOPO	0.34 3 : 3 : 1	0.36 3 : 3 : 1	1.88 1 : 1 : 2
TBPO (eq.) Li^+^ : [**L** ^n^]^−^ : TBPO	0.96 2 : 2 : 2	0.97 2 : 2 : 2	–

**Figure 9:**
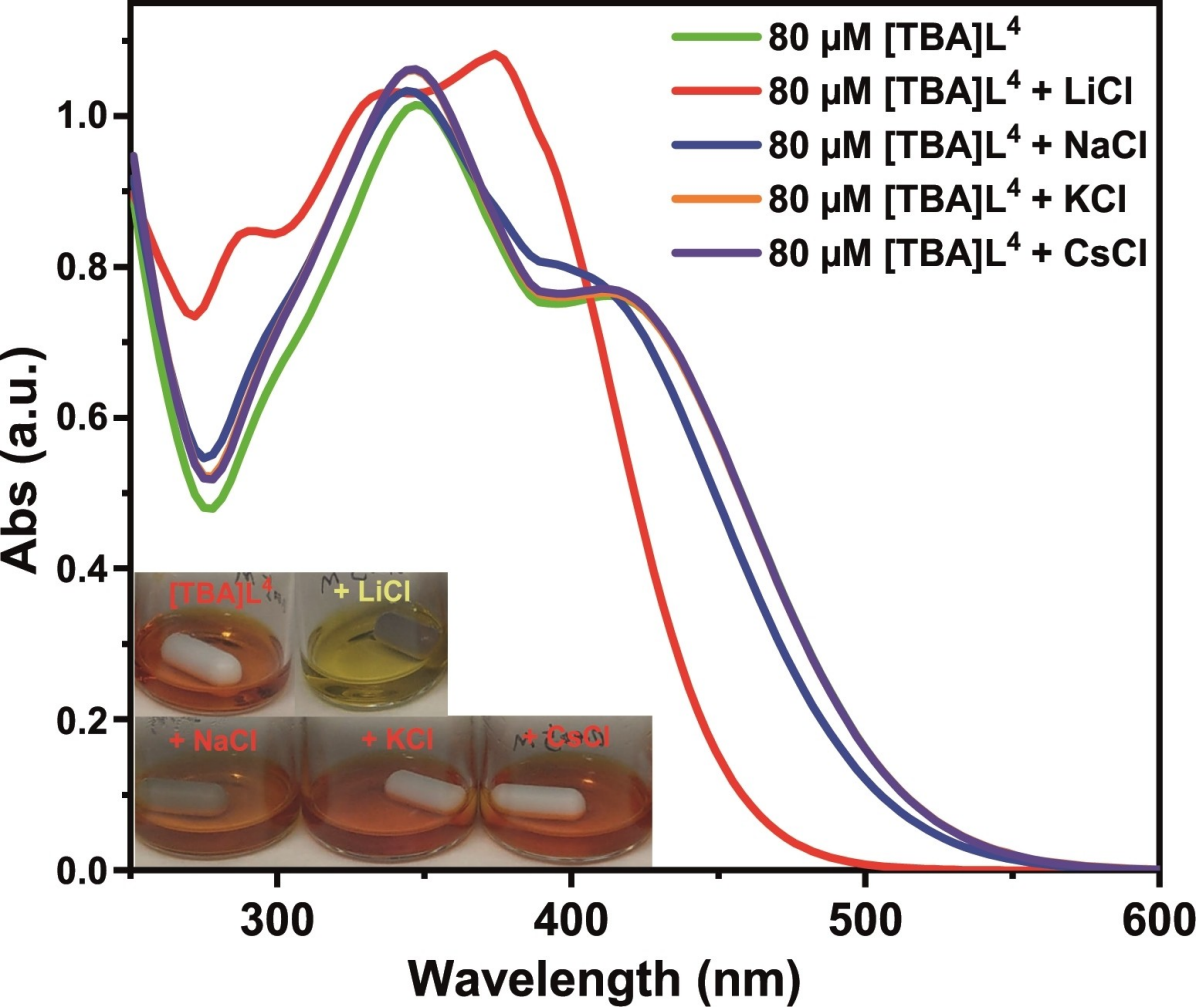
UV‐vis absorption spectra of [TBA]**L** ^4^ (80 μM) in the presence of equimolar quantities of LiCl, NaCl, KCl, CsCl in MeCN.

**Figure 10:**
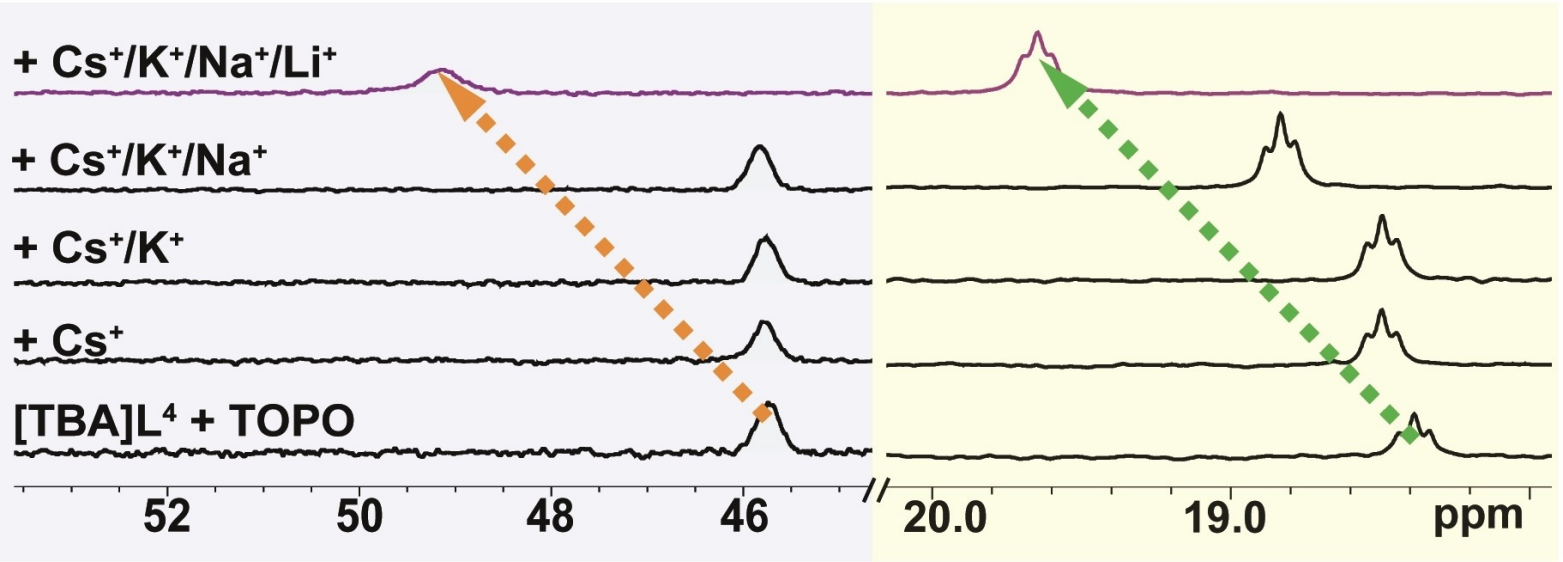
Partial ^31^P NMR spectra (CD_3_CN, 300 K) of an equimolar mixture of [TBA]**L** ^4^ and TOPO (3 mM each) following the step‐wise addition of 3 mM CsCl, KCl, NaCl and LiCl. TOPO (gray shaded, left), [**L** ^4^]^−^ (light yellow shaded, right).

UV‐vis and ^31^P NMR studies were performed in the presence of [TBA]**L** ^4^ in order to probe the potential selectivity of [**L** ^4^]^−^ for Li^+^ over Na^+^, K^+^ and Cs^+^. [TBA]**L** ^4^ was used to avoid the presence (and detection) of simple deprotonation processes during complex formation. For the UV‐vis studies, 5 mM [TBA]**L** ^4^ was mixed with equimolar quantities of LiCl, NaCl, KCl, and CsCl in MeCN for 4 h. Images of the resulting solutions and the corresponding UV‐vis spectra are depicted in Figure 9. Notably, only the LiCl‐containing system exhibited a color change (from red to pale yellow) while for the NaCl, KCl and CsCl systems no color change was evident. These naked eye observations correlate well with the respective spectra, with the spectra both in the absence and presence of NaCl, KCl or CsCl being very similar while that of [TBA]**L** ^4^ plus LiCl differs. In this latter case the spectrum shows the absence of the prominent shoulder at 413 nm observed in the spectrum of [TBA]**L** ^4^. There is also a bathochromic shift of 27 nm from the absorption maximum for [TBA]**L** ^4^ at 347 nm to 374 nm in the LiCl case. In contrast, only minor spectral changes are evident for the NaCl, KCl and CsCl systems, in accordance with the preferred recognition of Li^+^ by the [**L** ^4^]^−^ anion. The above selective Li^+^ binding was further investigated by NMR studies using an equimolar mixture of [TBA]**L** ^4^ and TOPO (3 mM each) in CD_3_CN. This solution was subsequently treated with an equimolar amount of CsCl, with the procedure repeated with KCl, NaCl and LiCl substituted for CsCl. After each addition, the reaction mixture was stirred for 18 h in an inert atmosphere. The ^31^P NMR spectra in CD_3_CN of the respective mixtures are shown in Figure 10. Upon the addition of CsCl and KCl a slight downfield shift for the [**L** ^4^]^−^ resonance was observed (Figure 10), whereas practically no shifts of the ^31^P resonance of TOPO were evident. After the addition of NaCl, the ^31^P resonance of [**L** ^4^]^−^ shifts by approximately 0.4 ppm. However, a significantly increased downfield shift of the ^31^P resonance of [**L** ^4^]^−^ (1.3 ppm) was observed when LiCl was added, which points to a clear selectivity for Li^+^ over the other alkali metal ions. Greater differences occur for the ^31^P resonance of TOPO. On addition of LiCl to the solution, a downfield shift of 3.4 ppm was observed, and the peak broadens. This observation is in accord with the phosphine oxide moiety of TOPO also participating in the coordination of the Li^+^. The NMR selectivity studies under LLE conditions also confirm a clear selectivity for Li^+^ (Figure S75).

**Figure 11:**
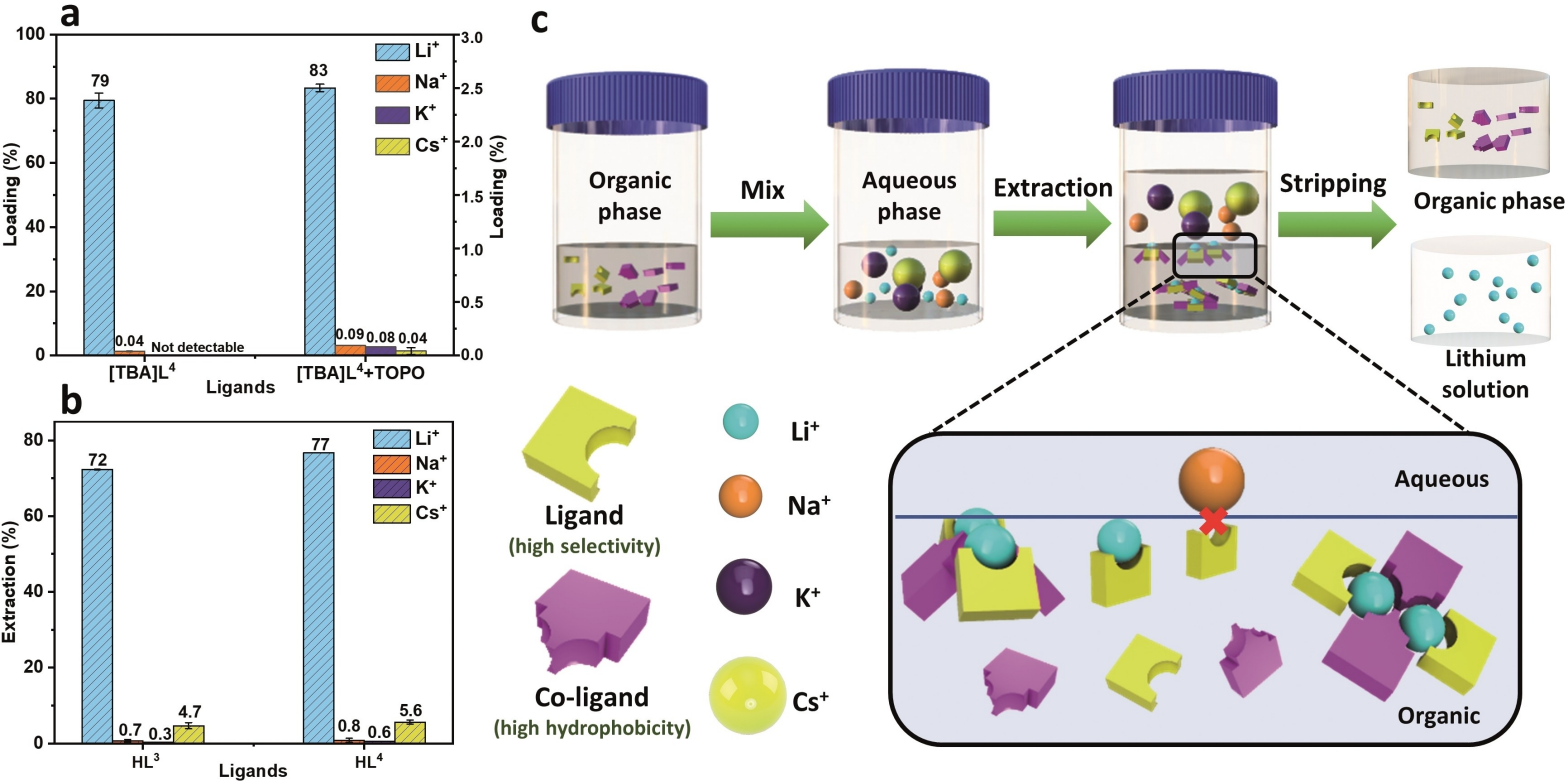
a) SLE loading of 5 mM [TBA]**L** ^4^ in the presence and absence of 5 mM TOPO in CHCl_3_ for the indicated alkali metal ions using a solid mixture of LiCl, NaCl, KCl, CsCl with each salt in 50 times excess (250 mM); b) Percent extraction of Li^+^, Na^+^, K^+^ and Cs^+^ competitively extracted with H**L** ^3^ and H**L** ^4^ in the presence of TOPO. [LiCl]=0.01 M, [NaCl]=0.1 M, [KCl]=0.1 M, [CsCl]=0.1 M, [NH_4_Cl]=0.1 M, pH=8.3 (Tris/HCl buffer), [H**L**]=0.1 M, [TOPO]=0.2 M in CHCl_3_, 298 K, 1 h; c) Schematic illustration of the extraction process with the selective Li^+^ capture by the cooperation of ligand and co‐ligand.

In an extension of the above studies, competitive SLE experiments in the presence of 50 times excess of the alkali chloride salts were carried out employing [TBA]**L** ^4^ and a mixture of [TBA]**L** ^4^ and TOPO in CHCl_3_.^[23]^ The loadings for all four metal ions in both experiments are depicted in Figure 11a. A loading of 79 % for Li^+^ was obtained while Na^+^, K^+^, and Cs^+^ are effectively not loaded (all <0.1 %). When TOPO is present, the loading percentage for Li^+^ was observed to marginally increase to 83 % (but still lies within experimental error of 79 %) while the loading of Na^+^, K^+^ and Cs^+^ remains negligible (<0.1 %). These results again demonstrate the high selectivity of [TBA]**L** ^4^ for Li^+^ over the other alkali metal ions under the SLE conditions employed. Moreover, at best, only a marginal synergistic effect is observed in the presence of the co‐ligand TOPO. To investigate whether the 4‐phosphoryl pyrazolone ligands are also capable of extracting Li^+^ under competitive conditions, LLE experiments in the presence of 10‐fold excess of Na^+^, K^+^ and Cs^+^ were carried out using H**L** ^3^ and H**L** ^4^ (Figure 11b). The results show close to undifferentiated (high) Li^+^ extraction for both ligands (72 % for H**L** ^3^ and 77 % for H**L** ^4^), whereas any extraction of the competing metal ions is very limited. For Cs^+^ an extraction of 5 % by H**L** ^3^ and 6 % by H**L** ^4^ was observed, while Na^+^ and K^+^ were extracted in trace amounts (<1%). These results demonstrate the impressive high affinity and selectivity of these ligand systems towards Li^+^, which are clearly illustrated from the extraction process under LLE conditions given in Figure 11c. The bite size between two chelating O‐donor of these ligands may provide a suitable fit for the coordination with Li^+^ which make them remarkably selective for the capture of this ion. The formation of complexes involving the co‐ligand TOPO results in the capability to extract Li^+^ into the organic phase. It should be noted that there are only a few synthetic receptors known that can extract Li^+^ from water – a situation arising from the previously mentioned high enthalpy of hydration of this ion,[^5a^, ^6b^, ^11b^] and the often need for very basic conditions (pH >11).[^5b^, ^12c^]

## Conclusion

Despite a wealth of previous reports on reagents for lithium ion coordination and extraction in recent decades, the development of more effective reagents for use under mild conditions has remained challenging. This study demonstrates that the present 4‐phosphoryl pyrazolones represent a new class of ligands that are excellent reagents for the recognition and extraction of lithium ions in the presence of excess sodium, potassium and cesium ions. Single crystal X‐ray analyses confirm the formation of binuclear Li^+^ complexes in the solid state in which co‐coordination of MeCN, TBPO, or TBP occurs to yield a 2 : 2 : 2 (Li^+^ : [L^*n*^]^−^ : solvent/co‐ligand) stoichiometry in each case. In contrast, in the presence of TOPO, unusual trinuclear complexes are formed in which the three metal centers are bridged by the O‐donor atom of TOPO to yield a tetrahedral [Li_3_O]‐core. Detailed NMR and MS studies reveal the presence of these polynuclear species in solution. Their dynamic aggregation and reversible conversion in two steps were clearly monitored by multinuclear NMR spectroscopy. Detailed slope analysis and loading experiments under LLE conditions further confirmed the corresponding active species dependence of co‐ligands. It is worth noting that the overall explorations provide a good example of the use of solid and solution studies for understanding the coordination mechanism in mixed‐ligand systems. We propose that the present study provides a powerful basis, not only for the development of further simple lithium‐selective receptor systems that display high extraction efficiency at moderately basic pH values, but also for related d‐ and f‐block metal complexation/extraction studies ‐ including for achieving rare earth separation. The ready functionalization of the present (new) class of N,O,P‐donor phosphoryl pyrazolone ligands will clearly also facilitate the successful exploitation of these opportunities. Studies of the above type are planned for the near future.

## Experimental Section

General experimental procedures for the synthesis of all compounds, characterization, liquid‐liquid extraction, solid‐liquid extraction and X‐ray crystallography are described in the Supporting Information.

Deposition Number 2101594 (for H**L** ^2^), 2101595 (for H**L** ^3^), 2101600 (for H**L** ^4^), 2101598 (for [TBA]**L** ^2^), 2101601 (for [TBA]**L** ^3^), 2101604 (for [TBA]**L** ^4^), 2101602 (for **4**), 2101589 (for **5**), 2101590 (for **6**), 2101591 (for **7**), 2101592 (for **8**), 2101603 (for **9**), 2101599 (for **10**), 2101593 (for **11**), 2101596 (for **12**), 2101597 (for **13**) contain the supplementary crystallographic data for this paper. These data are provided free of charge by the joint Cambridge Crystallographic Data Centre and Fachinformationszentrum Karlsruhe Access Structures service.

## Conflict of interest

The authors declare no conflict of interest.

1

## Supporting information

As a service to our authors and readers, this journal provides supporting information supplied by the authors. Such materials are peer reviewed and may be re‐organized for online delivery, but are not copy‐edited or typeset. Technical support issues arising from supporting information (other than missing files) should be addressed to the authors.

Supporting Information

## Data Availability

The data that support the findings of this study are available in the supplementary material of this article.

## References

[1a] J. B. Goodenough , K.-S. Park , J. Am. Chem. Soc. 2013, 135, 1167–1176; 23294028 10.1021/ja3091438

[1b] I. W. Donald , B. L. Metcalfe , L. A. Gerrard , J. Am. Ceram. Soc. 2008, 91, 715–720;

[1c] V. P. Parikh , A. Ahmadi , M. H. Parekh , F. Sadeghi , V. G. Pol , Environ. Sci. Technol. 2019, 53, 3757–3763; 30821445 10.1021/acs.est.8b07016

[1d] N. J. Birch , Chem. Rev. 1999, 99, 2659–2682; 11749496 10.1021/cr9804240

[1e] K. D. Mjos , C. Orvig , Chem. Rev. 2014, 114, 4540–4563. 24456146 10.1021/cr400460s

[2a] L. Oliveira , M. Messagie , S. Rangaraju , J. Sanfelix , M. Hernandez Rivas , J. Van Mierlo , J. Cleaner Prod. 2015, 108, 354–362;

[2b] H. Bae , Y. Kim , Mater. Adv. 2021, 2, 3234–3250.

[3] A. Sonoc , J. Jeswiet , V. K. Soo , Procedia CIRP 2015, 29, 752–757.

[4] B. Swain , Sep. Purif. Technol. 2017, 172, 388–403.

[5a] Q. He , N. J. Williams , J. H. Oh , V. M. Lynch , S. K. Kim , B. A. Moyer , J. L. Sessler , Angew. Chem. Int. Ed. 2018, 57, 11924–11928; 10.1002/anie.201805127 29800493

[5b] A. Masmoudi , G. Zante , D. Trébouet , R. Barillon , M. Boltoeva , Sep. Purif. Technol. 2021, 255, 117653;

[5c] T. Hanada , M. Goto , ACS Sustainable Chem. Eng. 2021, 9, 2152–2160;

[5d] L. C. Zhang , L. J. Li , H. M. Rui , D. Shi , X. W. Peng , L. M. Ji , X. X. Song , J. Hazard. Mater. 2020, 398, 122840; 32516726 10.1016/j.jhazmat.2020.122840

[5e] W. Chen , X. W. Li , L. L. Chen , G. L. Zhou , Q. Q. Lu , Y. Huang , Y. H. Chao , W. S. Zhu , Chem. Eng. J. 2021, 420, 127648;

[5f] L. Cui , K. Jiang , J. F. Wang , K. Dong , X. P. Zhang , F. Q. Cheng , AIChE J. 2019, 65, e16606.

[6a] Q. He , Z. Zhang , J. T. Brewster , V. M. Lynch , S. K. Kim , J. L. Sessler , J. Am. Chem. Soc. 2016, 138, 9779–9782; 27442768 10.1021/jacs.6b05713

[6b] H. Gohil , S. Chatterjee , S. Yadav , E. Suresh , A. R. Paital , Inorg. Chem. 2019, 58, 7209–7219. 31091090 10.1021/acs.inorgchem.9b00135

[7] S. Wang , S. Zheng , Z. Wang , W. Cui , H. Zhang , L. Yang , Y. Zhang , P. Li , Chem. Eng. J. 2018, 332, 160–168.

[8a] Y. Guo , Y. L. Ying , Y. Y. Mao , X. S. Peng , B. L. Chen , Angew. Chem. Int. Ed. 2016, 55, 15120–15124; 10.1002/anie.201607329 27805300

[8b] A. Razmjou , M. Asadnia , E. Hosseini , A. H. Korayem , V. Chen , Nat. Commun. 2019, 10, 5793; 31857585 10.1038/s41467-019-13648-7 PMC6923379

[8c] J. Lu , H. C. Zhang , J. Hou , X. Y. Li , X. Y. Hu , Y. X. Hu , C. D. Easton , Q. Y. Li , C. H. Sun , A. W. Thornton , M. R. Hill , X. W. Zhang , G. P. Jiang , J. Z. Liu , A. J. Hill , B. D. Freeman , L. Jiang , H. T. Wang , Nat. Mater. 2020, 19, 767–774; 32152561 10.1038/s41563-020-0634-7

[8d] J. Hou , H. C. Zhang , A. W. Thornton , A. J. Hill , H. T. Wang , K. Konstas , Adv. Funct. Mater. 2021, 10.1002/adfm.202105991.

[9a] S. Zavahir , T. Elmakki , M. Gulied , Z. Ahmad , L. Al-Sulaiti , H. K. Shon , Y. Chen , H. Park , B. Batchelor , D. S. Han , Desalination 2021, 500, 114883;

[9b] S. X. Yang , F. Zhang , H. P. Ding , P. He , H. S. Zhou , Joule 2018, 2, 1648–1651;

[9c] P. Srimuk , X. Su , J. Yoon , D. Aurbach , V. Presser , Nat. Rev. Mater. 2020, 5, 517–538.

[10a] A. Kumar , H. Fukuda , T. A. Hatton , V. J. H. Lienhard , ACS Energy Lett. 2019, 4, 1471–1474;

[10b] Y. C. Bai , N. Muralidharan , Y. K. Sun , S. Passerini , M. S. Whittingham , I. Belharouak , Mater. Today 2020, 41, 304–315.

[11a] B. Swain , J. Chem. Technol. Biotechnol. 2016, 91, 2549–2562;

[11b] J. M. Mahoney , A. M. Beatty , B. D. Smith , Inorg. Chem. 2004, 43, 7617–7621; 15554626 10.1021/ic049066b

[11c] G. Liu , Z. Zhao , A. Ghahreman , Hydrometallurgy 2019, 187, 81–100;

[11d] A. M. Li , H. J. Zhai , J. L. Li , Q. He , Chem. Lett. 2020, 49, 1125–1135.

[12a] L. Zhang , L. Li , D. Shi , X. Peng , F. Song , F. Nie , W. Han , Hydrometallurgy 2018, 175, 35–42;

[12b] T. V. Healy , J. Inorg. Nucl. Chem. 1968, 30, 1025–1036;

[12c] J. Wang , S. Yang , R. Bai , Y. Chen , S. Zhang , Ind. Eng. Chem. Res. 2019, 58, 1363–1372;

[12d] G. R. Harvianto , S. H. Kim , C. S. Ju , Rare Met. 2016, 35, 948–953;

[12e] Y. F. Song , Z. W. Zhao , L. H. He , Sep. Purif. Technol. 2020, 249, 8;

[12f] A. D. Sharma , N. D. Patil , A. W. Patwardhan , R. K. Moorthy , P. K. Ghosh , Sep. Sci. Technol. 2016, 51, 2242–2254;

[12g] L. C. Zhang , L. M. Ji , L. J. Li , D. Shi , T. S. Xu , X. W. Peng , X. X. Song , Hydrometallurgy 2021, 204, 105718.

[13a] H. Mukai , S. Miyazaki , S. Umetani , S. Kihara , M. Matsui , Anal. Chim. Acta 1989, 220, 111–117;

[13b] Y. Marcus , J. Chem. Soc. Faraday Trans. 1991, 87, 2995–2999;

[13c] J. Mahler , I. Persson , Inorg. Chem. 2012, 51, 425–438. 22168370 10.1021/ic2018693 PMC3250073

[14a] Y. Luo , N. Marets , T. Kato , Chem. Sci. 2018, 9, 608–616; 29629125 10.1039/c7sc03652c PMC5868304

[14b] X. Guo , Y. Yang , Z. Peng , Y. Cai , W. Feng , L. Yuan , Org. Chem. Front. 2019, 6, 2654–2661.

[15a] Z. Z. Lai , T. Zhao , J. L. Sessler , Q. He , Coord. Chem. Rev. 2020, 425, 14;

[15b] Q. He , G. I. Vargas-Zuniga , S. H. Kim , S. K. Kim , J. L. Sessler , Chem. Rev. 2019, 119, 9753–9835; 31081334 10.1021/acs.chemrev.8b00734

[15c] A. J. McConnell , P. D. Beer , Angew. Chem. Int. Ed. 2012, 51, 5052–5061; 10.1002/anie.201107244 22419667

[15d] K. Marsalek , V. Sindelar , Org. Lett. 2020, 22, 1633–1637; 32023070 10.1021/acs.orglett.0c00216

[15e] K. I. Hong , H. Kim , Y. Kim , M. G. Choi , W. D. Jang , Chem. Commun. 2020, 56, 10541–10544; 10.1039/d0cc04809g 32780033

[15f] J. V. Gavette , J. Lara , L. L. Reling , M. M. Haley , D. W. Johnson , Chem. Sci. 2013, 4, 585–590. 23505609 10.1039/C2SC21501B PMC3595051

[16] F. Marchetti , R. Pettinari , C. Pettinari , Coord. Chem. Rev. 2015, 303, 1–31.

[17a] F. Marchetti , R. Pettinari , C. Di Nicola , C. Pettinari , J. Palmucci , R. Scopelliti , T. Riedel , B. Therrien , A. Galindo , P. J. Dyson , Dalton Trans. 2018, 47, 868–878; 29255821 10.1039/c7dt04249c

[17b] F. Marchetti , J. Palmucci , C. Pettinari , R. Pettinari , S. Scuri , I. Grappasonni , M. Cocchioni , M. Amati , F. Lelj , A. Crispini , Inorg. Chem. 2016, 55, 5453–5466; 27177324 10.1021/acs.inorgchem.6b00495

[17c] J. Xu , K. N. Raymond , Angew. Chem. Int. Ed. 2006, 45, 6480–6485; 10.1002/anie.200602060 16952184

[17d] M. A. Petrova , ACS Sustainable Chem. Eng. 2016, 4, 2366–2375.

[18a] H. Bukowsky , E. Uhlemann , K. Gloe , P. Muhl , Anal. Chim. Acta 1992, 257, 105–108;

[18b] S. Umetani , K. Maeda , S. Kihara , M. Matsui , Talanta 1987, 34, 779–782. 18964405 10.1016/0039-9140(87)80095-4

[19] J. Zhang , M. Wenzel , K. Schnaars , F. Hennersdorf , K. Schwedtmann , J. März , A. Rossberg , P. Kaden , F. Kraus , T. Stumpf , J. J. Weigand , Dalton Trans. 2021, 50, 3550–3558. 33605972 10.1039/d1dt00365h

[20a] M. K. Purohit , S. K. Chakka , I. Scovell , A. Neschadim , A. M. Bello , N. Salum , Y. Katsman , M. C. Bareau , D. R. Branch , L. P. Kotra , Bioorg. Med. Chem. 2014, 22, 2739–2752; 24685704 10.1016/j.bmc.2014.03.016

[20b] B. S. Jensen , Acta Chem. Scand. 1959, 13, 1668–1670;

[20c] V. B. Kurteva , M. A. Petrova , J. Chem. Educ. 2014, 92, 382–384.

[21] B. Corbel , I. L′Hostis-Kervella , J.-P. Haelters , Synth. Commun. 1996, 26, 2561–2568.

[22a] J. Modranka , R. Jakubowski , M. Rozalski , U. Krajewska , A. Janecka , K. Gach , D. Pomorska , T. Janecki , Eur. J. Med. Chem. 2015, 92, 565–574; 25602933 10.1016/j.ejmech.2015.01.029

[22b] V. Zsoldos-Mády , O. Ozohanics , A. Csámpai , V. Kudar , D. Frigyes , P. Sohár , J. Organomet. Chem. 2009, 694, 4185–4195.

[23] For further details see Supporting Information.

[24] L. A. Malaspina , A. H. White , D. Wege , M. B. Tolmie , B. W. Skelton , S. Grabowsky , Struct. Chem. 2017, 28, 1343–1357.

[25] P. A. Stabnikov , L. G. Bulusheva , N. I. Alferova , A. I. Smolentsev , I. A. Korol'kov , N. V. Pervukhina , I. A. Baidina , J. Struct. Chem. 2012, 53, 740–747.

[26a] W. Holzer , K. Mereiter , B. Plagens , Heterocycles 1999, 50, 799–818;

[26b] A. B. Uzoukwu , S. S. Aljuaid , P. B. Hitchcock , J. D. Smith , Polyhedron 1993, 12, 2719–2724.

[27a] H. J. Reich , Chem. Rev. 2013, 113, 7130–7178; 23941648 10.1021/cr400187u

[27b] K. J. Jin , D. B. Collum , J. Am. Chem. Soc. 2015, 137, 14446–14455. 26554898 10.1021/jacs.5b09524 PMC4762874

[28] U. Olsher , R. M. Izatt , J. S. Bradshaw , N. K. Dalley , Chem. Rev. 1991, 91, 137–164.

[29] G. Hilmersson , P. I. Arvidsson , O. Davidsson , M. Hakansson , J. Am. Chem. Soc. 1998, 120, 8143–8149.

[30a] S. Harder , J. Boersma , L. Brandsma , J. A. Kanters , W. Bauer , P. V. Schleyer , Organometallics 1989, 8, 1696–1700;

[30b] C. Strohmann , V. H. Gessner , Angew. Chem. Int. Ed. 2007, 46, 8281–8283; 10.1002/anie.200702116 17899584

[30c] V. H. Gessner , C. Strohmann , Organometallics 2010, 29, 1858–1861.

[31a] K. Hirose , J. Inclusion Phenom. Macrocyclic Chem. 2001, 39, 193–209;

[31b] J. S. Renny , L. L. Tomasevich , E. H. Tallmadge , D. B. Collum , Angew. Chem. Int. Ed. 2013, 52, 11998–12013; 10.1002/anie.201304157 PMC4028694 24166797

[32a] H. Ju , Y. Tsuruoka , M. Hayano , E. Lee , K.-M. Park , M. Ikeda , J.-i. Ishi-i , S. Kuwahara , Y. Habata , Angew. Chem. Int. Ed. 2021, 60, 650–654; 10.1002/anie.202010436 32959445

[32b] H. Ju , T. Abe , Y. Takahashi , Y. Tsuruoka , A. Otsuka , E. Lee , M. Ikeda , S. Kuwahara , Y. Habata , Inorg. Chem. 2021, 60, 1738–1745. 33444007 10.1021/acs.inorgchem.0c03222

[33] H. Stephan , M. Kubeil , K. Gloe , K. Gloe , Analytical Methods in Supramolecular Chemistry (second edition) *(Ed.*: C. A. Schalley *)*, Wiley-VCH, Weinheim, 2012, p. 2105–2127.

[34a] Q. Zhao , W.-H. Chen , S.-J. Huang , Y.-C. Wu , H.-K. Lee , S.-B. Liu , J. Phys. Chem. B 2002, 106, 4462–4469;

[34b] Y. Seo , K. Cho , Y. Jung , R. Ryoo , ACS Catal. 2013, 3, 713–720.

[35] J. S. McIndoe , K. L. Vikse , J. Mass Spectrom. 2019, 54, 466–479. 30980780 10.1002/jms.4359

[36] Y. Marcus , A. S. Kertes , Ion Exchange and Solvent Extraction of Metal Complexes, Wiley, London, 1969, p. 1480–1487.

