# Supplementary material for: 4‐Phosphoryl Pyrazolones for Highly Selective Lithium Separation from Alkali Metal Ions
Source: Chemistry. 2021 Nov 5;28(1):e202103640. doi: 10.1002/chem.202103640 (PMC9298229; doi:10.1002/chem.202103640)
Supplement: Supplementary file 1 — Supporting Information [file CHEM-28-0-s001.pdf]

Chemistry–A European Journal

Supporting Information

4-Phosphoryl Pyrazolones for Highly Selective Lithium Separation from Alkali Metal Ions

Jianfeng Zhang, Marco Wenzel, Johannes Steup, Gerrit Schaper, Felix Hennersdorf, Hao Du, Shili Zheng, Leonard F. Lindoy, and Jan J. Weigand*

Table of Contents

Table of Contents 2

S1. General remarks. 3

S2. Synthesis of acylpyrazolone (HL^1) 4

S2.1 Preparation of 5-methyl-2-(4-nitrophenyl)-2,4-dihydro-3H-pyrazol-3-one (1) 4

S2.2. Preparation of 1-(5-hydroxy-3-methyl-1-(4-nitrophenyl)-1H-pyrazol-4-yl)ethan-1-one (HL^1) 4

S3. Synthesis of 4-phosphoryl pyrazolones 6

S3.1 Preparation of methyl 2-(diisopropoxyphosphoryl)acetate (2) 6

S3.2. General Procedure for the Synthesis of (3) 6

S3.2.1. Preparation of methyl 2-(diisopropoxyphosphoryl)-3-oxobutanoate (3a) 7

S3.2.2. Preparation of methyl 2-(diisopropoxyphosphoryl)-4-methyl-3-oxopentanoate (3b) 7

S3.2.3. Preparation of methyl 2-(diisopropoxyphosphoryl)-3-oxo-3-phenylpropanoate (3c) 8

S3.3. Preparation of diisopropyl (5-hydroxy-3-methyl-1-(4-nitrophenyl)-1H-pyrazol-4-yl)phosphonate (HL^2) 9

S3.4. Preparation of diisopropyl (5-hydroxy-3-isopropyl-1-(4-nitrophenyl)-1H-pyrazol-4-yl) phosphonate (HL^3) 11

S3.5. Preparation of diisopropyl(5-hydroxy-1-(4-nitrophenyl)-3-phenyl-1H-pyrazol-4-yl)phosphonate (HL^4) 13

S3.6. General Procedure for the Synthesis of [TBA]L^n salts 15

S3.6.1 Preparation of [TBA]L^2 15

S3.6.2 Preparation of [TBA]L^3 18

S3.6.3 Preparation of [TBA]L^4 20

S4. Synthesis of lithium complexes 22

S4.1. General procedure for the synthesis of acetonitrile-solvated lithium complexes 22

S4.1.1. Preparation of $[Li_2(L^2)_2(CH_3CN)_2]$ (4) 22

S4.1.2. Preparation of $[Li_2(L^3)_2(CH_3CN)_2]$ (5) 25

S4.1.3. Preparation of $[Li_2(L^4)_2(CH_3CN)_2]$ (6) 28

S4.2. Preparation of $[Li(L^1)(EtOH)_2]$ (7) 31

S4.3. General procedure for the synthesis of TBPO-coordinated lithium complexes 33

S4.3.1. Preparation of $[Li_2(L^1)_2(TBPO)_2]$ (8) 33

S4.3.2. Preparation of $[Li_2(L^3)_2(TBPO)_2]$ (9) 36

S4.3.3 Preparation of [$Li_2(L^4)_2(TBPO)_2$]·$2CH_2Cl_2$ (10) 39

S4.4. Preparation of [$Li_2(L^4)_2(TBP)_2$] (11) 42

S4.5. Preparation of [$Li_3(L^3)_3(TOPO)$]·$0.65C_5H_{12}$ (12) 45

S4.6. Preparation of [$Li_3(L^4)_3(TOPO)$]·$0.67C_5H_{12}$ (13) 48

S5. Crystal structures 51

S6. NMR studies 52

S6.1 Method of Continuous Variation 52

S6.2 Mole Ratio Method 52

S7. Mass Spectral studies 55

S8. n-octanol/water distribution 57

S9. Li^+ single element extraction under LLE conditions 58

S10. Slope analysis and loading experiments 58

S11. Selectivity studies 61

S11.1 Selectivity studies by NMR under LLE conditions 61

S11.2 Selectivity studies under SLE conditions 61

S12. Crystallographic details. 62

S12.1 Structure solution and refinement 62

References 66

S1. General remarks.

General Considerations: all manipulations were performed with HPLC grade, analytical grade or technical grade reagents and solvents, which were used without further purification. The starting materials used were purchased from *SIGMA-ALDRICH, FLUKA, MERCK, VWR, TCI, ABCR CHEMICALS, ACROS, CARL ROTH or WAKO*.
Manipulations under dry and oxygen-free conditions were performed in a Glovebox MB Unilab or using Schlenk techniques under an atmosphere of purified nitrogen. Dry, oxygen-free solvents (CH_2Cl_2, CH_3CN (distilled from CaH_2), *n*-hexane, *n*-pentane, Et_2O, 1,4-dioxane (distilled from potassium)) were employed. Anhydrous deuterated acetonitrile (CD_3CN), dichloromethane (CD_2Cl_2), chloroform ($CDCl_3$) and methanol (CD_3OD) were purchased from Sigma-Aldrich, Deutero or Eurisotop. All distilled and deuterated solvents were stored over molecular sieves (4 Å: CH_2Cl_2, CD_2Cl_2, C_6D_6, *n*-hexane, Et_2O; 3 Å: CH_3CN, CD_3CN). All glassware was oven-dried at 160 °C prior to use.
NMR spectra were measured on a Bruker AVANCE III HD Nanobay, 400 MHz UltraSield (^1H (400.13 MHz), ^{13}C (100.61 MHz), ^{31}P (161.98 MHz,) ^7Li (155.51 MHz)) or on a Bruker AVANCE III HDX, 500 MHz Ascend (^1H (500.13 MHz), ^{13}C (125.75 MHz), ^{31}P

(202.45 MHz), ^{7}Li (194.37 MHz)). All ^{13}C NMR spectra were exclusively recorded with composite pulse decoupling. Reported numbers assigning atoms in the ^{13}C spectra were indirectly deduced from the cross-peaks in 2D correlation experiments (HMBC, HSQC). Chemical shifts were referenced to the respective solvent to δ = 7.26 ppm (^{1}H), 77.16 ppm (^{13}C) for $CDCl_3$, δ = 5.32 ppm (^{1}H), 53.84 ppm (^{13}C) for CD_2Cl_2, δ = 1.94 ppm (^{1}H), 118.26 ppm (^{13}C) for CD_3CN, δ = 3.31 ppm (^{1}H), 49.00 ppm (^{13}C) for CD_3OD)[1] and $\delta_{H3PO4(85\%)}$ = 0.00 ppm (^{31}P, externally). Chemical shifts (*δ*) are reported in ppm. Coupling constants (J) are reported in Hz. Melting points were recorded on an electrothermal melting point apparatus (Büchi Switzerland, Melting point M-560) in sealed capillaries (under nitrogen atmosphere for air or moisture sensitive compounds) and are uncorrected. Infrared (IR) and Raman spectra were recorded at ambient temperature using a Bruker Vertex 70 instrument equipped with a RAM II module (Nd-YAG laser, 1064 nm). The Raman intensities are reported in percent relative to the most intense peak and are given in parenthesis. An ATR unit (diamond) was used for recording IR spectra. The intensities are reported relative to the most intense peak and are given in parenthesis using the following abbreviations: vw = very weak, w = weak, m = medium, s = strong, vs = very strong. Elemental analyses were performed on a Vario MICRO cube Elemental Analyzer by Elementar Analysatorsysteme GmbH in CHNS modus. For the mass spectrometry experiments a waters ACQUITY UPLC H-Class system in combination with an ACQUITY TQ Detector V4.1 SCN849 SCN896 was used. MassLynx V4.1 SCN849 SCN896 served as the evaluation software. The required ionization was provided by the electrospray method (ESI). The diluent for the samples was an acetonitrile water mixture containing both solvents in a 70:30 (acetonitrile: water) ratio and additionally 0.1% of formic acid. UV/Vis data were recorded on a PerkinElmer LAMBDA 2 double beam spectrometer at room temperature against blank solvent using quartz cuvettes with 10 mm path lengths.

S2. Synthesis of acylpyrazolone (HL^{1})

S2.1 Preparation of 5-methyl-2-(4-nitrophenyl)-2,4-dihydro-3H-pyrazol-3-one (1)

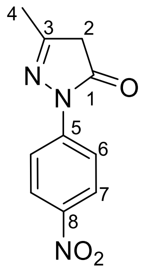

The general procedure for the synthesis of **1** is reported in the literature.[2] 4-nitrophenyl hydrazine hydrochloride (15.31 g, 79.13 mmol, 1.0 eq.) were dissolved in 100 ml acetic acid and ethyl-3-oxobutanoate (13.01 ml, 102.87 mmol, 1.3 eq.) were added to this solution under stirring. The mixture was refluxed 3 h then cooled to 0 °C and 200 ml Et_2O were added. The reaction mixture was stirred for 1 h at 0 °C and filtered to give a yellow-brown powder. The crude product was recrystallized from methanol and purified by column chromatography (CH_2Cl_2 : CH_3OH = 200 : 1, Rf = 0.53).

Yield: 3.14 g (17%); **^{1}H NMR** ($CDCl_3$, 300K, in ppm): δ = 2.24 (3H, s, H4), 3.50 (2H, s, H2), 8.11-8.15 (2H, m, H6), 8.24-8.28 (2H, m, H7); **^{13}C{^{1}H} NMR** ($CDCl_3$, 300K, in ppm): 17.3 (1C, s, C4), 43.2 (1C, s, C2), 118.0 (2C, s, C6), 125.0 (2C, s, C7), 143.3 (1C, s, C5), 144.1 (1C, s, C8), 157.7 (1C, s, C3), 171.0 (1C, s, C1).

S2.2. Preparation of 1-(5-hydroxy-3-methyl-1-(4-nitrophenyl)-1H-pyrazol-4-yl)ethan-1-one (HL^{1})

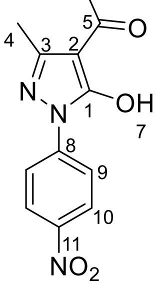

The synthesis of H**L**^{1} was based on the procedure reported by Jensen and Kurteva and was conducted under a N_2 atmosphere.[3] **1** (5-methyl-2-(4-nitrophenyl)-2,4-dihydro-3H-pyrazol-3-one, 3.08 g, 13.21 mmol, 1.0 eq.) were suspended in 70 ml dry 1,4-dioxane and gently heated until completely dissolved. $Ca(OH)_2$ (2.50 g, 33.74 mmol, 2.5 eq.) were added and the mixture was refluxed for 0.5 h. The reaction mixture was cooled using an ice bath and acetyl chloride (1.45 ml, 19.78 mmol, 1.5 eq.) were added dropwise. The resulting solution was refluxed for 1.5 h, cooled to room temperature and 100 ml 10% (v/v) HCl (10 ml, 37% HCl) were added. The mixture was stirred overnight at room temperature. The precipitate was filtered off and washed with H_2O and ethanol to give a light yellow powder in 85% yield.

Yield: 3.02 g (85%); **mp.**: 204 °C; **Raman** (100 mW, 298K, in cm^{-1}): 3094 (13), 2930 (20), 1630 (9), 1592 (100), 1544 (11), 1521 (7), 1440 (10), 1407 (7), 1377 (13), 1333 (93), 1299 (34), 1198 (12), 1179 (22), 1116 (58), 1045 (14), 1010 (12), 864 (27), 738 (12), 695 (10), 641 (11), 572 (6), 231 (7), 196 (6); **IR** (ATR, 298 K, in cm^{-1}): 3119 (vw), 3079 (vw), 2928 (vw), 1933 (vw), 1699 (vw), 1650 (m),

1628 (m), 1591 (s), 1543 (w), 1517 (vs), 1499 (s), 1430 (w), 1405 (w), 1378 (w), 1336 (vs), 1312 (vs), 1200 (s), 1127 (vw), 1114 (w), 1072 (w), 1044 (w), 1009 (w), 969 (w), 901 (w), 862 (vw), 848 (vs), 829 (s), 761 (vw), 750 (s), 737 (w), 686 (m), 641 (w), 571 (w), 530 (vw), 516 (w), 498 (m), 466 (m); **^{1}H NMR** ($CDCl_3$, 300K, in ppm): δ = 2.47 (3H, s, H4), 2.49 (3H, s, H6), 8.15-8.18 (2H, m, H9), 8.29-8.32 (2H, m, H10); **^{13}C{^{1}H} NMR** ($CDCl_3$, 300K, in ppm): δ = 16.0 (1C, s, C4), 24.7 (1C, s, C6), 105.2 (1C, s, C2), 119.4 (2C, s, C9), 125.0 (2C, s, C10), 142.8 (1C, s, C8), 145.0 (1C, s, C11), 149.1 (1C, s, C3), 164.2 (1C, s, C1), 190.8 (1C, s, C5); **Elemental analysis** for $C_{12}H_{11}N_3O_4$, calculated: C 55.17, H 4.24, N 16.09; found: C 55.17, H 4.13, N 16.04; **ESI-MS** (m/z, [Da/e]): 259.8 $[M-H]^-$.

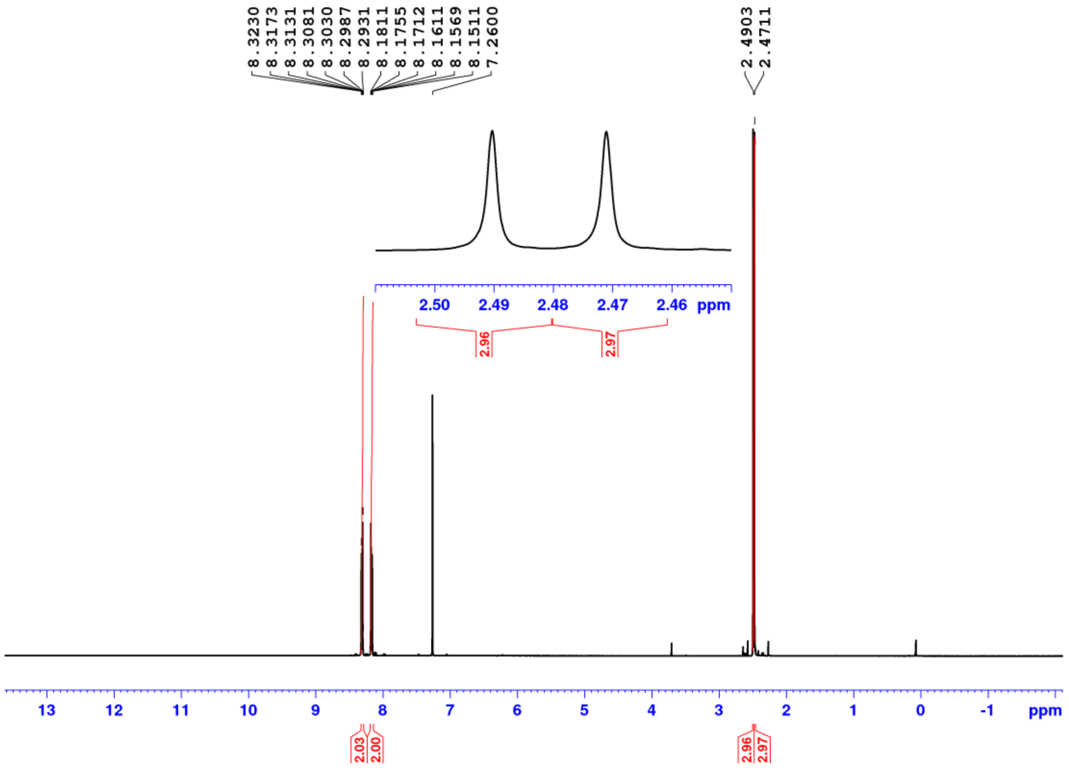

Figure S1. ^{1}H NMR of H**L**^{1} ($CDCl_3$, 300 K)

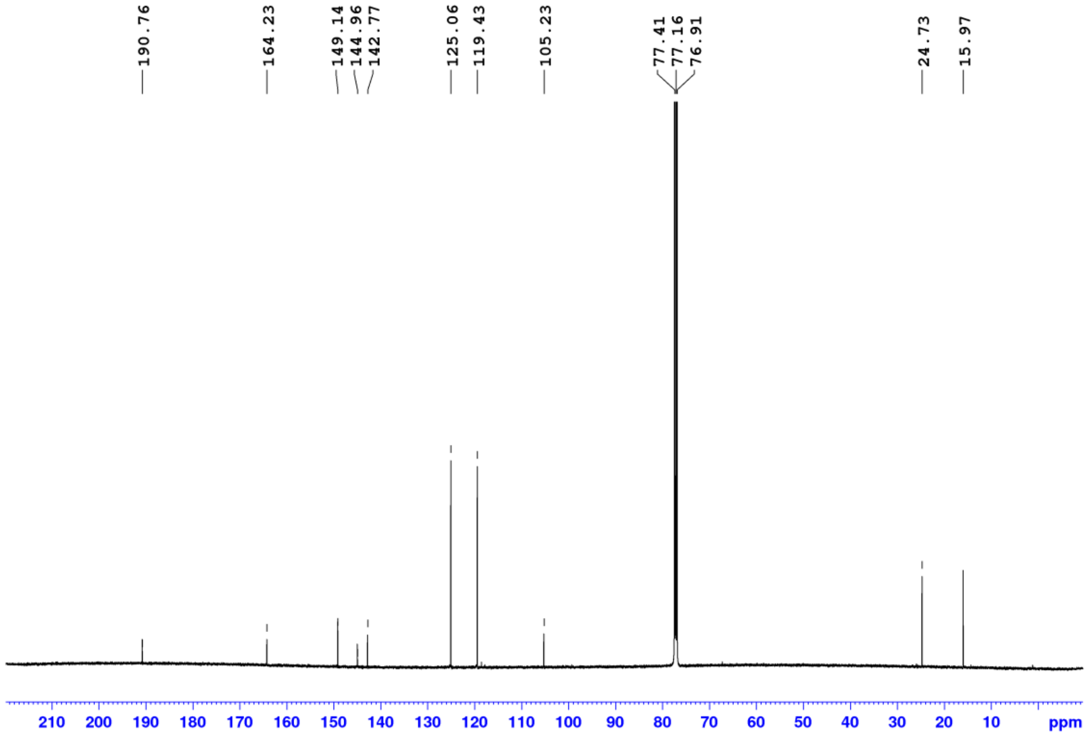

Figure S2. $^{13}C\{^{1}H\}$ NMR of HL^{1} ($CDCl_3$, 300 K)

S3. Synthesis of 4-phosphoryl pyrazolones

S3.1 Preparation of methyl 2-(diisopropoxyphosphoryl)acetate (2)

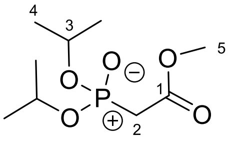

Methyl 2-(diisopropoxyphosphoryl)acetate **2** was obtained by the procedure presented in a recent report by our group.[4] Triisopropyl phosphite (54.83 g, 0.25 mol, 1.0 eq.) was heated to 120 °C and 25.62 ml of methyl bromoacetate (97%, 0.26 mol, 1.05 eq.) were slowly added over 2 h. while keeping the temperature at 120 °C. Simultaneously, the generated 2-bromopropane was distilled from the reaction mixture at about 35 - 50 °C inner temperature in the distillation apparatus. After the addition of methyl bromoacetate was completed, the amount of evolving 2-bromopropane decreased and the reaction mixture was kept at 120 - 130 °C for an additional 2 h. After cooling, residual volatiles were removed under reduced pressure to yield 61.91 g of colourless oil containing the crude product **2** in 94% purity (determined by integration of the ^{31}P NMR spectrum). The product can be used without further purification (or after distillation in 99% purity).

Yield: 61.91 g (98%); **^{1}H NMR** ($CDCl_3$, 300K, in ppm): δ = 1.27 (12H, d, $^{3}J_{HH}$ = 6.2 Hz, H4), 2.86 (2H, d, $^{2}J_{HP}$ = 21.8 Hz, H2), 3.66 (3H, s, H5), 4.68 (2H, d sept, $^{3}J_{HP}$ = 7.6 Hz, $^{3}J_{HH}$ = 6.2 Hz, H3); **$^{13}C\{^{1}H\}$ NMR** ($CDCl_3$, 300K, in ppm): δ = 23.8 (2C, d, $^{3}J_{CP}$ = 5 Hz, C4a), 24.0 (2C, d, $^{3}J_{CP}$ = 4 Hz, C4b), 35.3 (1C, d, $^{1}J_{CP}$ = 135 Hz, C2), 52.3 (1C, s, C5), 71.5 (2C, d, $^{2}J_{CP}$ = 7 Hz, C3), 166.4 (1C, d, $^{2}J_{CP}$ = 6 Hz, C1); **$^{31}P\{^{1}H\}$ NMR** ($CDCl_3$, 300K, in ppm): δ = 17.3 (s); **^{31}P NMR** ($CDCl_3$, 300K, in ppm): δ = 17.3 (tt, $^{2}J_{PH}$ = 21.5 Hz, $^{3}J_{PH}$ = 12.1 Hz).

S3.2. General Procedure for the Synthesis of (3)

The general procedure for the synthesis of **3** corresponds to that given in the literature.[5] All experiments were carried out under a dried N_2 atmosphere, except the acidification step using 1M HCl. 1.0 eq. of **2** (methyl 2-(diisopropoxyphosphoryl)acetate) was dissolved in dried dichloromethane (CH_2Cl_2) and the solution was added to a suspension of anhydrous $MgCl_2$ (1.0 eq.) in the same solvent. Then 2.0 eq. of triethylamine (Et_3N) were added and the mixture was stirred at room temperature for 1 h. The reaction mixture was placed in an ice-water bath and 1.1 eq. of the corresponding acyl chloride was added slowly. Defined equivalents of Et_3N and acyl chloride were

added step-wise in intervals of 0.5 - 1 h to ensure the completeness of reaction. The resulting red-orange suspension was quenched using 1 M HCl (2.0 eq) and extracted 3 times with CH_2Cl_2. The combined organic phases were dried over Na_2SO_4, filtered, and the solvent was evaporated under reduced pressure. The crude product was purified by column chromatography to yield a yellow oil containing a mixture of the different keto-enol-tautomers of the target molecules.

S3.2.1. Preparation of methyl 2-(diisopropoxyphosphoryl)-3-oxobutanoate (3a)

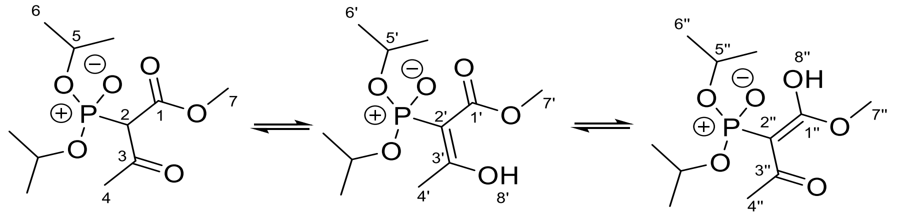

Derivative **3a** was prepared according to the general procedure. **2** (94%, 12.77 g, 50.39 mmol) in 20 ml CH_2Cl_2; $MgCl_2$ (4.90 g, 50.95 mmol) in 30 ml CH_2Cl_2; Et_3N step 1: 14.00 ml, 100.49 mmol; step 2: 7.00 ml, 50.25 mmol; step 3: 3.50 ml, 25.12 mmol; Acetyl chloride step 1: 4.28 ml, 58.78 mmol; step 2: 2.14 ml, 29.39 mmol; step 3: 1.07 ml, 14.70 mmol. The yellow oily crude product was purified by column chromatography (iso-hexane : EtOAc = 4 : 1, Rf = 0.60) to give 9.87 g (70%) of **3a** in 99% purity.

Yield: 9.87 g (70%); **Raman** (255 mW, 298 K, in cm^{-1}): 2983 (49), 2939 (100), 2927 (100), 2875 (31), 2738 (9), 1712 (10), 1587 (6), 1454 (16), 1355 (7), 887 (7), 734 (7), 129 (6), 69 (25); **IR** (ATR, 298 K, in cm^{-1}): 2982 (vw), 2937 (vw), 1744 (vw), 1709 (m), 1591 (w), 1466 (vw), 1437 (w), 1412 (w), 1385 (w), 1375 (m), 1329 (w), 1242 (s), 1180 (w), 1144 (w), 1103 (m), 1080 (m), 982 (vs), 899 (w), 887 (w), 851 (vw), 812 (vw), 781 (w), 771 (w), 737 (vw), 625 (w), 598 (w), 534 (m), 449 (w), 422 (w), 413 (w); **^{1}H NMR** ($CDCl_3$, 300K, in ppm): δ = 1.22-1.26 (6H, m, H6a/H6´a/H6´´a), 1.31-1.34 (6H, m, H6b/H6´b/H6´´b), 2.39 (3H, s, H4), 2.43 (3H, d, $^{4}J_{HP}$ = 0.7 Hz, H4´), 2.51 (3H, d, $^{4}J_{HP}$ = 0.8 Hz, H4´´), 3.69 (3H, s, H7´), 3.76 (3H, s, H7), 3.79 (3H, s, H7´´), 4.18 (1H, d, $^{2}J_{HP}$ = 23.8 Hz, H2), 4.57 (2H, d sept, $^{3}J_{HP}$ = 8.4 Hz, $^{3}J_{HH}$ = 6.2 Hz, H5´), 4.60 (2H, m, H5´´), 4.81 (2H, m, H5), 13.81 (1H, s, H8´), 14.55 (1H, s, H8´´); **^{13}C{^{1}H} NMR** ($CDCl_3$, 300K, in ppm): δ = 22.6 (1C, s, C4´´), 23.0 (1C, s, C4´a), 23.1 (1C, s, C4´b), 23.6-24.2 (4C, m, C6/C6´/C6´´), 30.0 (1C, s, C4), 51.1 (1C, s, C7´), 52.1 (1C, s, C7´´), 52.9 (1C, s, C7), 62.7 (1C, d, $^{1}J_{CP}$ = 126 Hz, C2), 70.4 (2C, d, $^{2}J_{CP}$ = 5 Hz, C5´´), 71.9 (2C, d, $^{2}J_{CP}$ = 5 Hz, C5´), 72.8 (1C, d, $^{2}J_{CP}$ = 7 Hz, C5a), 72.9 (1C, d, $^{2}J_{CP}$ = 7 Hz, C5b), 89.5 (1C, d, $^{1}J_{CP}$ = 179 Hz, C2´), 92.0 (1C, d, $^{1}J_{CP}$ = 206 Hz, C2´´), 164.9 (1C, d, $^{2}J_{CP}$ = 6 Hz, C1), 166.8 (1C, d, $^{2}J_{CP}$ = 9 Hz, C1´), 173.8 (1C, d, $^{2}J_{CP}$ = 9 Hz, C1´´), 187.9 (1C, d, $^{2}J_{CP}$ = 6 Hz, C3´), 189.6 (1C, d, $^{2}J_{CP}$ = 22 Hz, C3´´), 196.6 (1C, d, $^{2}J_{CP}$ = 5 Hz, C3); **^{31}P{^{1}H} NMR** ($CDCl_3$, 300K, in ppm): δ = 11.2 (s, P), 14.5 (s, P´´), 23.0 (s, P´); **^{31}P NMR** ($CDCl_3$, 300K, in ppm): δ = 11.2 (td, $^{2}J_{PH}$ = 23.7 Hz, $^{3}J_{PH}$ = 7.6 Hz, P), 14.5 (t, $^{3}J_{PH}$ = 8.3 Hz, P´´), 23.0 (t, $^{3}J_{PH}$ = 8.3 Hz, P´).

S3.2.2. Preparation of methyl 2-(diisopropoxyphosphoryl)-4-methyl-3-oxopentanoate (3b)

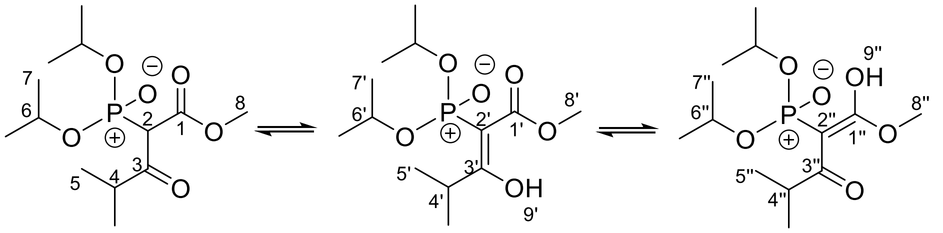

Derivative **3b** was prepared according to the general procedure. **2** (99%, 20.20 g, 83.95 mmol) in 40 ml CH_2Cl_2; $MgCl_2$ (99%, 8.07 g, 83.90 mmol) in 50 ml CH_2Cl_2; Et_3N step 1: 23.30 ml, 167.25 mmol; step 2: 11.65 ml, 83.63 mmol; step 3: 5.83 ml, 41.85 mmol; isobutyryl chloride step 1: 9.70 ml, 90.73 mmol; step 2: 4.90 ml, 45.83 mmol; step 3: 2.45 ml, 22.92 mmol. The yellow oily crude product was purified by column chromatography (iso-hexane : EtOAc = 4 : 1, Rf = 0.36) to give 17.84 g (68%) of **3b** in 99% purity.

Yield: 17.84 g (68%); **Raman** (100 mW, 298 K, in cm^{-1}): 2982 (58), 2942 (100), 2924 (94), 2875 (50), 2843 (8), 2763 (5), 2735 (8), 1708 (15), 1582 (13), 1452 (33), 1387 (5), 1336 (14), 1179 (5), 1144 (9), 1103 (18), 920 (11), 888 (10), 837 (9), 792 (8), 769 (8), 738 (14), 426 (8); **IR** (ATR, 298 K, in cm^{-1}): 2979 (w), 2936 (vw), 2876 (vw), 1746 (vw), 1707 (m), 1578 (w), 1469 (vw), 1436 (w), 1386 (w), 1375 (w), 1322 (w), 1260 (w), 1231 (m), 1180 (w), 1145 (w), 1101 (w), 1078 (m), 983 (vs), 937 (w), 899 (w), 887 (w), 836 (vw), 785 (w),

769 (w), 735 (vw), 659 (w), 583 (m), 539 (w); **^{1}H NMR** ($CDCl_3$, 300K, in ppm): δ = 1.07-1.16 (18H, m, H5/H5´/H5´´), 1.24-1.33 (18H, m, H7/H7´/H7´´), 2.53 (1H, sept, $^{3}J_{HH}$ = 7.0 Hz, H4´´), 2.99 (1H, sept, $^{3}J_{HH}$ = 6.8 Hz, H4), 3.68 (3H, s, H8´), 3.73 (3H, s, H8), 3.78 (3H, s, H8´´), 3.81 (1H, sept, $^{3}J_{HH}$ = 6.6 Hz, H4´, E/Z), 4.43 (1H, d, $^{2}J_{HP}$ = 22.8 Hz, H2), 4.56 (2H, d sept, $^{3}J_{HP}$ = 8.4 Hz, $^{3}J_{HH}$ = 6.2 Hz, H6´), 4.71 (2H, d sept, $^{3}J_{HP}$ = 8.8 Hz, $^{3}J_{HH}$ = 6.2 Hz, H6´´), 4.79 (1H, d sept, $^{3}J_{HP}$ = 7.4 Hz, $^{3}J_{HH}$ = 5.9 Hz, H6a), 4.83 (1H, d sept, $^{3}J_{HP}$ = 7.2 Hz, $^{3}J_{HH}$ = 6.0 Hz, H6b), 13.80 (1H, s, H9´), 14.74 (1H, s, H9´´); **^{13}C{^{1}H} NMR** ($CDCl_3$, 300K, in ppm): δ = 17.8-20.1 (2C, m, C5/C5´/C5´´), 23.6-24.3 (4C, m, C7/C7´/C7´´), 32.4 (1C, s, C4´a), 32.5 (1C, s, C4´b), 33.8 (1C, s, C4´´), 41.3 (1C, s, C4), 51.1 (1C, s, C8´), 52.1 (1C, s, C8´´), 52.9 (1C, s, C8), 59.6 (1C, d, $^{1}J_{CP}$ = 130 Hz, C2), 71.5 (2C, d, $^{2}J_{CP}$ = 7 Hz, C6´´), 71.9 (2C, d, $^{2}J_{CP}$ = 5 Hz, C6´), 72.6 (1C, d, $^{2}J_{CP}$ = 7 Hz, C6a), 72.9 (1C, d, $^{2}J_{CP}$ = 7 Hz, C6b), 87.9 (1C, d, $^{1}J_{CP}$ = 176 Hz, C2´), 90.2 (1C, d, $^{1}J_{CP}$ = 207 Hz, C2´´), 164.8 (1C, d, $^{2}J_{CP}$ = 5 Hz, C1), 166.6 (1C, d, $^{2}J_{CP}$ = 9 Hz, C1´), 174.2 (1C, d, $^{2}J_{CP}$ = 10 Hz, C1´´), 180.5 (1C, s, C3´´), 194.4 (1C, d, $^{2}J_{CP}$ = 4 Hz, C3´), 202.4 (1C, d, $^{2}J_{CP}$ = 6 Hz, C3); **^{31}P{^{1}H} NMR** ($CDCl_3$, 300K, in ppm): δ = 11.5 (s, P), 14.5 (s, P´´), 23.7 (s, P´); **^{31}P NMR** ($CDCl_3$, 300K, in ppm): δ = 11.5 (td, $^{2}J_{PH}$ = 22.6 Hz, $^{3}J_{PH}$ = 7.3 Hz, P), 14.5 (t, $^{3}J_{PH}$ = 8.1 Hz, P´´), 23.7 (t, $^{3}J_{PH}$ = 8.1 Hz, P´).

S3.2.3. Preparation of methyl 2-(diisopropoxyphosphoryl)-3-oxo-3-phenylpropanoate (3c)

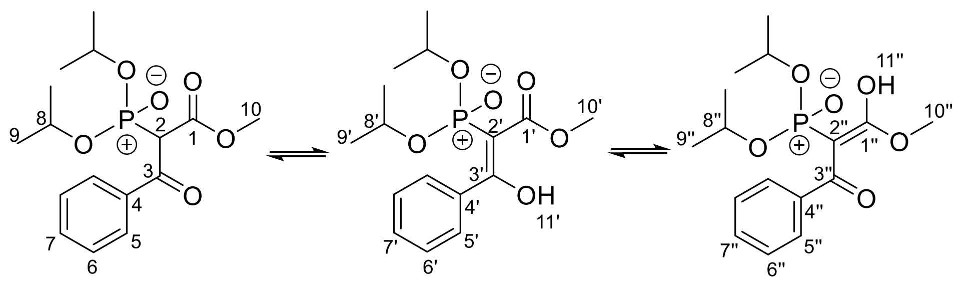

Derivative **3c** was prepared according to the general procedure. **2** (99%, 17.17 g, 71.36 mmol) in 25 ml CH_2Cl_2; $MgCl_2$ (99%, 6.90 g, 71.74 mmol) in 50 ml CH_2Cl_2; Et_3N (99.5%) step 1: 19.80 ml, 142.13 mmol; step 2: 9.90 ml, 71.06 mmol; step 3: 4.95 ml, 35.53 mmol; Benzoyl chloride (99%) step 1: 9.07 ml, 77.29 mmol; step 2: 4.54 ml, 38.69 mmol; step 3: 2.27 ml, 19.34 mmol. The yellow oily crude product was purified by column chromatography (iso-hexane : acetone = 3: 1, Rf = 0.38) to give 23.23 g (96%) of **3c** in 92% purity.

Yield: 23.23 g (96%); **Raman** (100 mW, 298 K, in cm^{-1}): 3070 (37), 2983 (24), 2940 (38), 2923 (31), 2875 (12), 2840 (5), 2739 (5), 1746 (27), 1687 (6), 1626 (48), 1601 (100), 1579 (15), 1494 (5), 1452 (17), 1355 (5), 1289 (12), 1235 (19), 1182 (9), 1162 (12), 1144 (5), 1107 (5), 1032 (10), 1002 (81), 935 (5), 890 (5), 848 (6), 766 (7), 618 (14), 405 (5); **IR** (ATR, 298 K, in cm^{-1}): 3064 (vw), 2980 (vw), 2936 (vw), 1731 (m), 1686 (vw), 1626 (w), 1599 (vw), 1578 (vw), 1493 (vw), 1450 (w), 1434 (vw), 1386 (vw), 1375 (vw), 1235 (vs), 1178 (w), 1142 (w), 1102 (m), 1079 (m), 1059 (s), 975 (vs), 935 (m), 888 (w), 847 (vw), 791 (w), 767 (m), 704 (s), 638 (vw), 617 (vw), 568 (w), 532 (m), 422 (vw); **^{1}H NMR** ($CDCl_3$, 300K, in ppm): δ = 1.16-1.33 (6H, m, H9/H9´/H9´´), 3.67 (3H, s, H10´/H10´´), 3.70 (3H, s, H10´/H10´´), 3.74 (3H, s, H10), 4.68 (2H, d sept, $^{3}J_{HP}$ = 7.2 Hz, $^{3}J_{HH}$ = 6.1 Hz, H8´/H8´´), 4.79 (2H, d sept, $^{3}J_{HP}$ = 7.4 Hz, $^{3}J_{HH}$ = 6.3 Hz, H8´/H8´´), 4.84-4.91 (2H, m, H8), 5.16 (1H, d, $^{2}J_{HP}$ = 22.4 Hz, H2), 7.32-7.61 (3H, m, H6/H6´/H6´´and H7/H7´/H7´´), 7.96 (2H, d, $^{3}J_{HH}$ = 7.5 Hz, H5), 8.02-8.04 (2H, m, H5´/H5´´), 8.13-8.15 (2H, m, H5´/H5´´), 13.33 (1H, s, H11´/H11´´); **^{13}C{^{1}H} NMR** ($CDCl_3$, 300K, in ppm): δ = 23.5-24.7 (4C, m, C9/C9´/C9´´), 52.7 (1C, s, C10´/C10´´), 52.8 (1C, s, C10´/C10´´), 53.1 (1C, s, C10), 56.5 (1C, d, $^{1}J_{CP}$ = 133 Hz, C2), 71.9 (2C, d, $^{2}J_{CP}$ = 6 Hz, C8´/C8´´), 72.2 (2C, d, $^{2}J_{CP}$ = 6 Hz, C8´/C8´´), 72.6 (1C, d, $^{2}J_{CP}$ = 7 Hz, C8a), 72.9 (1C, d, $^{2}J_{CP}$ = 7 Hz, C8b), 118.3 (1C, d, $^{1}J_{CP}$ = 189 Hz, C2´/C2´´), 118.5 (1C, d, $^{1}J_{CP}$ = 178 Hz, C2´/C2´´), 127.3-133.9 (3C, s, C6/C6´/C6´´ and C7/C7´/C7´´), 129.1 (2C, s, C5), 130.2 (2C, s, C5´/C5´´), 130.4 (2C, s, C5´/C5´´), 136.3 (1C, d, $^{3}J_{CP}$ = 2 Hz, C4), 136.8 (1C, d, $^{3}J_{CP}$ = 2 Hz, C4´/C4´´), 160.6 (1C, d, $^{3}J_{CP}$ = 3 Hz, C4´/C4´´), 163.5 (1C, d, $^{2}J_{CP}$ = 2 Hz, C3´/C3´´), 163.7 (1C, d, $^{2}J_{CP}$ = 2 Hz, C3´/C3´´), 164.9 (1C, d, $^{2}J_{CP}$ = 5 Hz, C1), 165.0 (1C, d, $^{2}J_{CP}$ = 9 Hz, C1´/C1´´), 165.7 (1C, d, $^{2}J_{CP}$ = 9 Hz, C1´/C1´´), 189.0 (1C, d, $^{2}J_{CP}$ = 6 Hz, C3); **^{31}P{^{1}H} NMR** ($CDCl_3$, 300K, in ppm): δ = 6.2 (s, P´/P´´), 11.4 (s, P), 21.4 (s, P´/P´´); **^{31}P NMR** ($CDCl_3$, 300K, in ppm): δ = 6.2 (t, $^{3}J_{PH}$ = 7.7 Hz, P´/P´´), 11.4 (td, $^{2}J_{PH}$ = 22.4 Hz, $^{3}J_{PH}$ = 7.5 Hz, P), 21.4 (t, $^{3}J_{PH}$ = 8.0 Hz, P´/P´´).

S3.3. Preparation of diisopropyl (5-hydroxy-3-methyl-1-(4-nitrophenyl)-1H-pyrazol-4-yl)phosphonate (H**L**^{2})

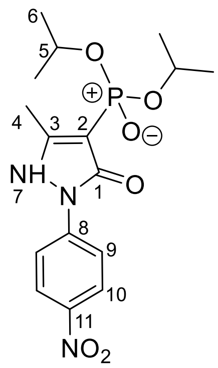

The ligand H**L**^{2} was synthesized following our recently reported method.[4] To a suspension of **3a** (9.86 g, 99%, 34.83 mmol, 1.0 eq.) in 130 ml H_2O, 4-nitrophenylhydrazine hydrochloride (7.41 g, 38.31 mmol, 1.1 eq.) was added and the mixture was refluxed for 3 h. After cooling to room temperature, potassium carbonate (9.63 g, 69.61 mmol, 2.0 eq.) were slowly added and the reaction mixture was refluxed for 4 h then stirred overnight at room temperature and washed with Et_2O (4 × 50 ml). The combined aqueous phase was acidified with 0.5 M HCl to pH = 2 and the yellow oil suspension was extracted with EtOAc (3 × 50 ml). The combined organic phase was dried over Na_2SO_4, filtered and the solution was evaporated under reduced pressure. H**L**^{2} was obtained as a yellow-brown solid in 61% yield with 95% purity and was used without further purification.

Yield: 8.52 g (61%); **mp.**: 148 °C; **Raman** (100 mW, 298K, in cm^{-1}): 2934 (6), 1598 (74), 1402 (6), 1367 (12), 1337 (100), 1184 (7), 1113 (33), 1051 (8), 1012 (6), 862 (10); **IR** (ATR, 298 K, in cm^{-1}): 3123 (vw), 2981 (vw), 2935 (vw), 1656 (m), 1607 (w), 1595 (w), 1549 (w), 1514 (s), 1500 (m), 1431 (vw), 1399 (vw), 1385 (w), 1372 (w), 1330 (s), 1317 (vs), 1303 (m), 1269 (w), 1217 (m), 1181 (w), 1142 (vw), 1108 (m), 1073 (vw), 1051 (vw), 996 (s), 974 (vs), 901 (w), 887 (w), 860 (m), 849 (m), 823 (w), 778 (s), 763 (w), 749 (s), 731 (vw), 684 (w), 666 (vw), 634 (w), 622 (w), 574 (vs), 532 (w), 513 (vw), 484 (vw), 436 (vw), 420 (vw), 417 (vw); **^{1}H NMR** ($CDCl_3$, 300K, in ppm): δ = 1.29 (6H, d, $^{3}J_{HH}$ = 6.2 Hz, H6a), 1.41 (6H, d, $^{3}J_{HH}$ = 6.1 Hz, H6b), 2.28 (3H, s, H4), 4.64 (2H, d sept, $^{3}J_{PH}$ = 7.6 Hz, $^{3}J_{HH}$ = 6.3 Hz, H5), 8.07-8.11 (2H, m, H9), 8.28-8.32 (2H, m, H10); **^{13}C{^{1}H} NMR** ($CDCl_3$, 300K, in ppm): δ = 14.1 (1C, s, C4), 23.9 (2C, d, $^{3}J_{CP}$ = 5 Hz, C6a), 24.1 (2C, d, $^{3}J_{CP}$ = 4 Hz, C6b), 72.0 (2C, d, $^{2}J_{CP}$ = 5 Hz, C5), 86.7 (1C, d, $^{1}J_{CP}$ = 217 Hz, C2), 120.3 (2C, s, C9), 125.0 (2C, s, C10), 143.1 (1C, s, C8), 145.2 (1C, s, C11), 151.0 (1C, d, $^{2}J_{CP}$ = 9 Hz, C3), 160.4 (1C, d, $^{2}J_{CP}$ = 24 Hz, C1); **^{31}P{^{1}H} NMR** ($CDCl_3$, 300K, in ppm): δ = 14.4 (s); **^{31}P NMR** ($CDCl_3$, 300K, in ppm): δ = 14.4 (t, $^{3}J_{PH}$ = 8.2 Hz); **Elemental analysis** for $C_{16}H_{22}N_3O_6P$, calculated: C 50.13, H 5.78, N 10.96; found: C 50.14, H 5.53, N 11.02; **ESI-MS** (m/z, [Da/e]): 382.2 [M-H]$^{-}$ (ESI^{-}), 384.4 [M+H]$^{+}$ (ESI^{+}).

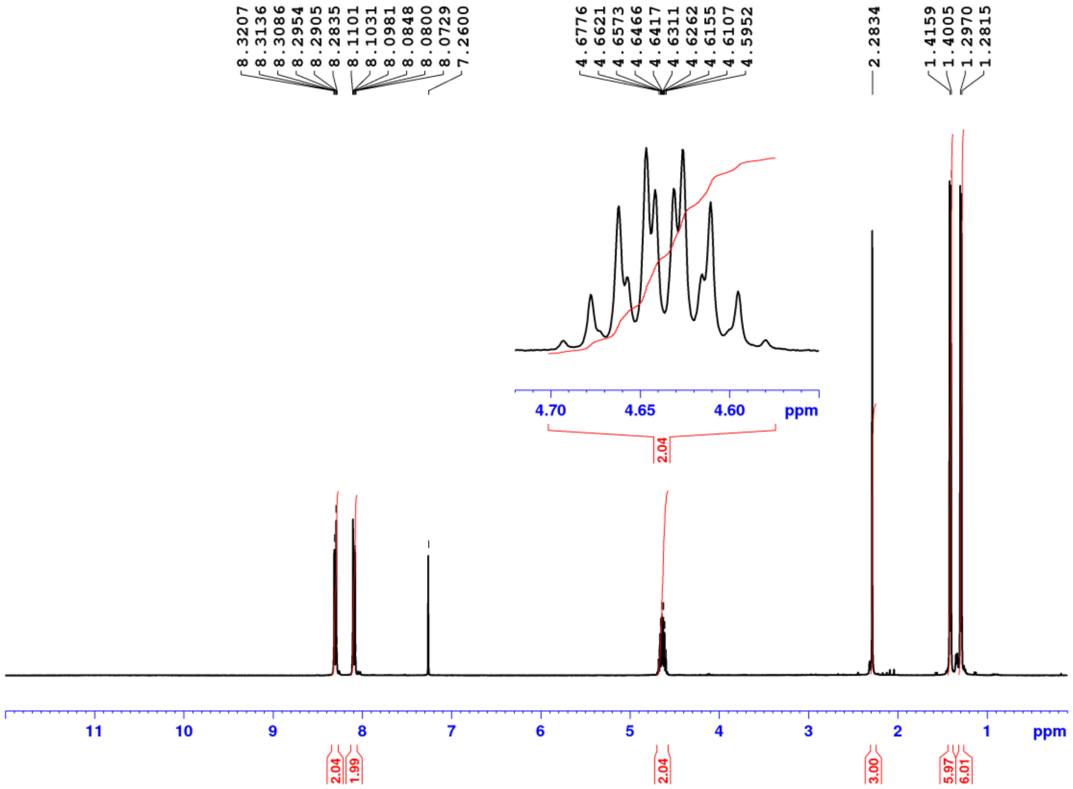

Figure S3. ^{1}H NMR of H**L**^{2} ($CDCl_3$, 300 K)

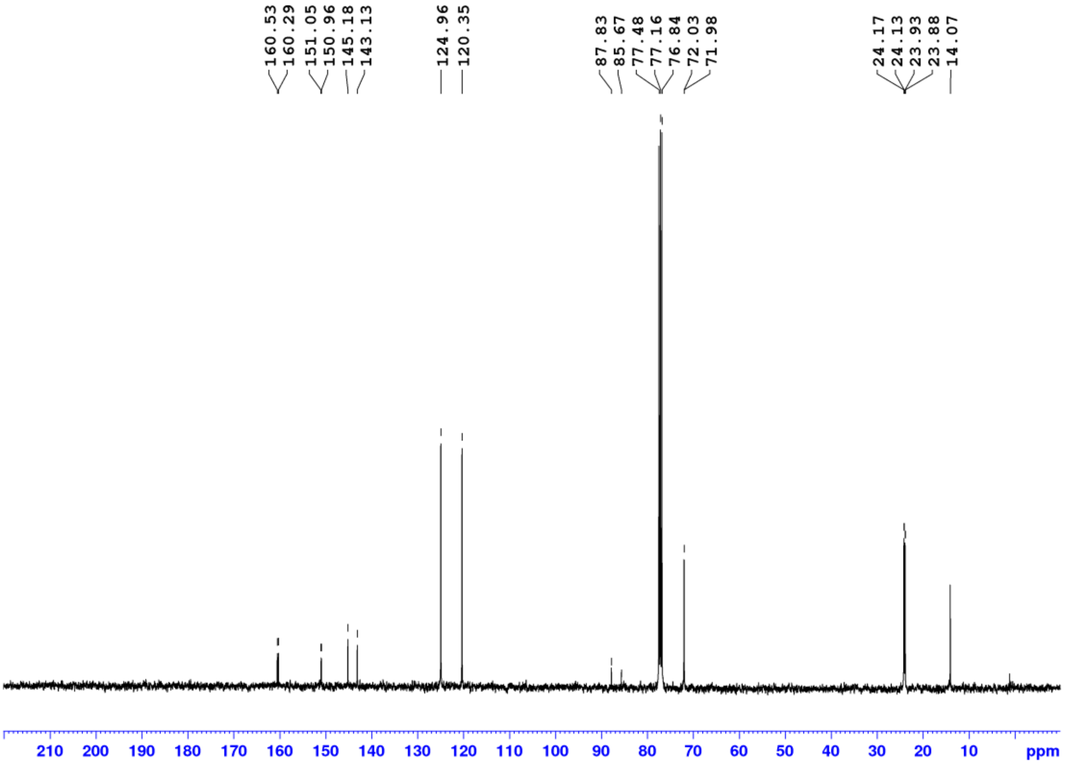

Figure S4. $^{13}C\{^{1}H\}$ NMR of H**L**^{2} ($CDCl_3$, 300 K)

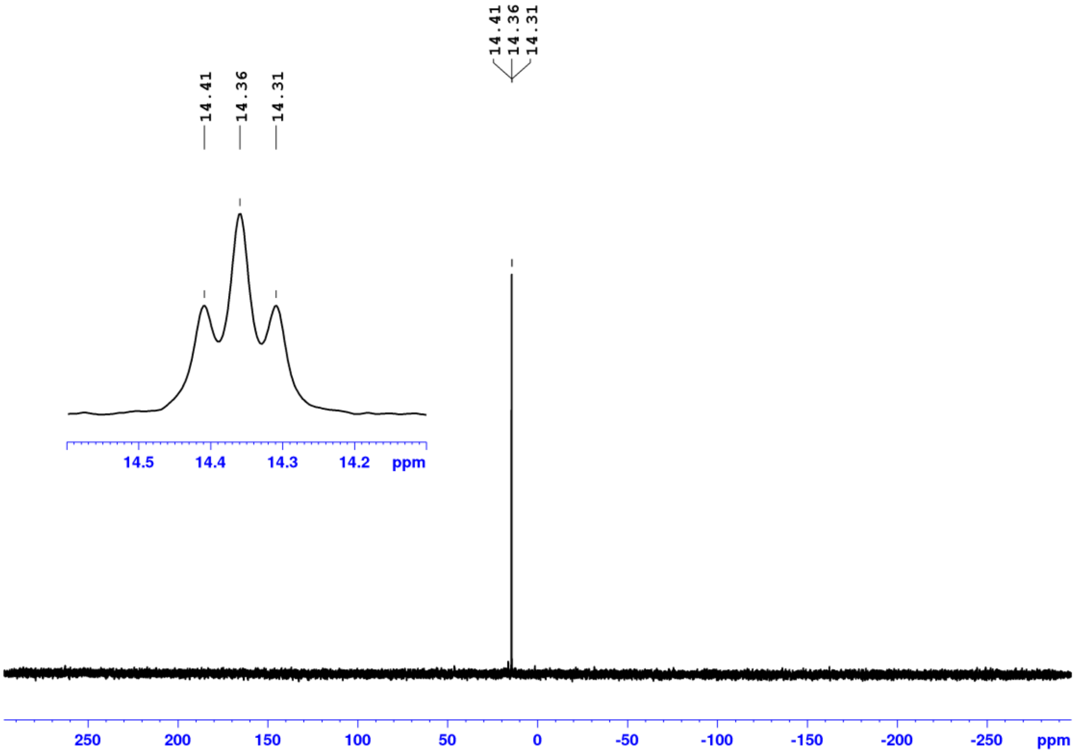

Figure S5. ^{31}P NMR of H**L**^{2} ($CDCl_3$, 300 K)

S3.4. Preparation of diisopropyl (5-hydroxy-3-isopropyl-1-(4-nitrophenyl)-1H-pyrazol-4-yl) phosphonate (HL^3)

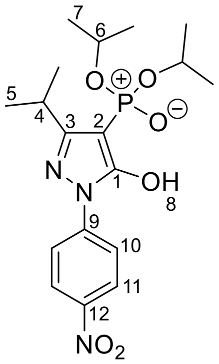

The synthesis of H**L**^{3} was based on procedures reported in the literature with some modifications.[6] **3b** (7.51 g, 99%, 24.12 mmol, 1.0 eq.) was dissolved in a mixture of AcOH (15 ml), EtOH (15 ml) and H_2O (100 ml) and 4-nitrophenylhydrazine hydrochloride (5.59 g, 28.89 mmol, 1.2 eq.) were added. The reaction mixture was refluxed for 11 h under a N_2 atmosphere. The resulting mixture was extracted with EtOAc (3 × 50 ml), washed with brine, dried over Na_2SO_4 and the solvent was evaporated under reduced pressure. The crude product was recrystallized from hot iso-hexane (~ 70 °C) and purified by column chromatography (iso-hexane : acetone = 2 : 1, Rf = 0.26). To remove remaining impurities the product obtained was dissolved in $CHCl_3$ and washed with 5 mM aqueous HCl solution (A/O = 1:1). The solvent was removed under reduce pressure to give 3.04 g of H**L**^{3} as yellow powder in 99% purity.

Yield: 3.04 g (31%); **mp.**: 111 °C; **Raman** (100 mW, 298K, in cm^{-1}): 2940 (5), 1595 (70), 1522 (13), 1424 (6), 1405 (13), 1344 (100), 1115 (28), 1095 (9), 887 (6); **IR** (ATR, 298 K, in cm^{-1}): 3187 (vw), 3120 (vw), 3087 (vw), 2983 (vw), 2967 (w), 2931 (vw), 2870 (vw), 1608 (w), 1594 (m), 1558 (m), 1516 (vs), 1493 (w), 1448 (w), 1423 (vw), 1407 (vw), 1383 (w), 1374 (vw), 1337 (s), 1281 (w), 1244 (vw), 1191 (s), 1169 (m), 1153 (m), 1115 (w), 1106 (w), 1092 (m), 1067 (m), 1032 (w), 983 (vs), 940 (m), 896 (w), 884 (w), 856 (s), 832 (vw), 802 (vw), 777 (m), 751 (m), 720 (w), 685 (w), 679 (w), 660 (m), 625 (w), 570 (s), 526 (w), 502 (w), 484 (w), 447 (w); **^{1}H NMR** ($CDCl_3$, 300K, in ppm): δ = 1.30 (6H, d, $^3J_{HH}$ = 6.2 Hz, H7a), 1.32 (6H, d, $^3J_{HH}$ = 6.9 Hz, H5), 1.40 (6H, d, $^3J_{HH}$ = 6.2 Hz, H7b), 2.96 (1H, sept, $^3J_{HH}$ = 6.9 Hz, H4), 4.68 (2H, d sept, $^3J_{HP}$ = 7.5 Hz, $^3J_{HH}$ = 6.3 Hz, H6), 8.09-8.12 (2H, m, H10), 8.26-8.30 (2H, m, H11); **^{13}C{^{1}H} NMR** ($CDCl_3$, 300K, in ppm): δ = 21.6 (2C, s, C5), 24.0 (2C, d, $^3J_{CP}$ = 5 Hz, C7a), 24.1 (2C, d, $^3J_{CP}$ = 4 Hz, C7b), 28.9 (1C, s, C4), 72.1 (2C, d, $^2J_{CP}$ = 6 Hz, C6), 85.5 (1C, d, $^1J_{CP}$ = 220 Hz, C2), 120.4 (2C, s, C10), 124.8 (2C, s, C11), 143.4 (1C, d, $^4J_{CP}$ = 1 Hz, C9), 145.0 (1C, s, C12), 160.1 (1C, d, $^2J_{CP}$ = 10 Hz, C3), 160.3 (1C, d, $^2J_{CP}$ = 25 Hz, C1); **^{31}P{^{1}H} NMR** ($CDCl_3$, 300K, in ppm): δ = 15.1 (s); **^{31}P NMR** ($CDCl_3$, 300K, in ppm): δ = 15.1 (t, $^3J_{PH}$ = 7.9 Hz); **Elemental analysis** for $C_{18}H_{26}N_3O_6P$, calculated: C 52.55, H 6.37, N 10.21; found: C 52.54, H 6.30, N 10.20; **ESI-MS** (m/z, [Da/e]): 410.1 $[M-H]^-$ (ESI^-), 412.3 $[M+H]^+$ (ESI^+).

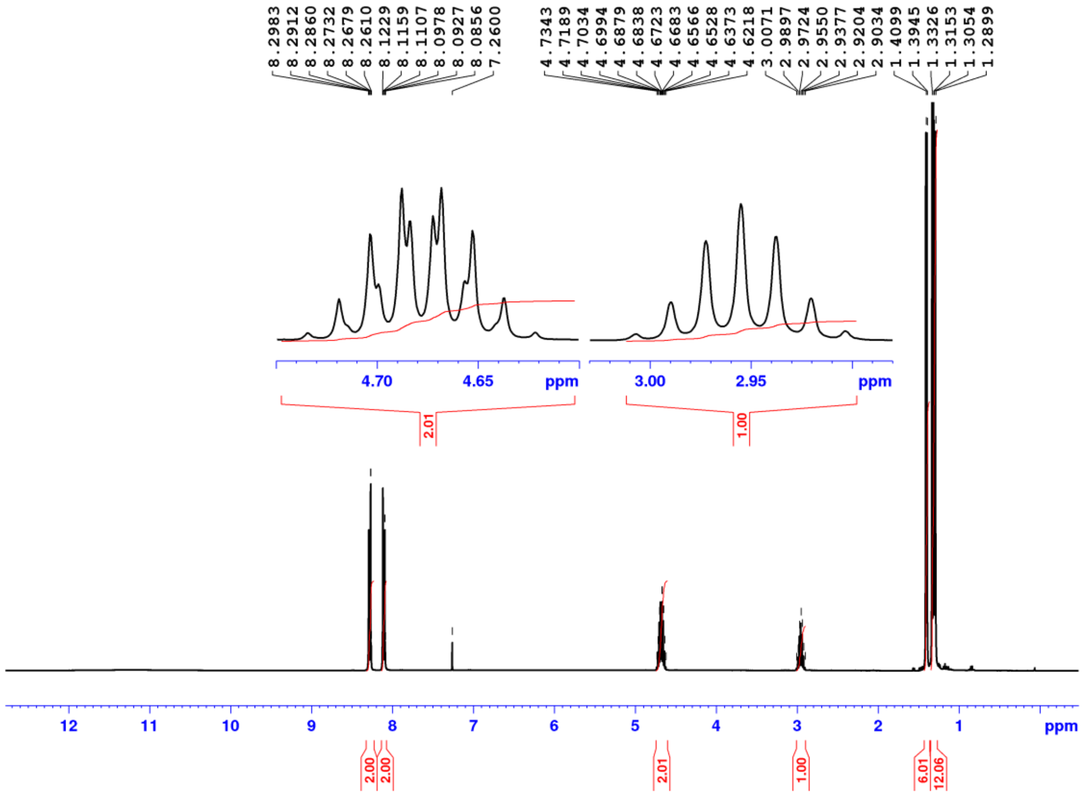

Figure S6. ^{1}H NMR of H**L**^{3} ($CDCl_3$, 300 K)

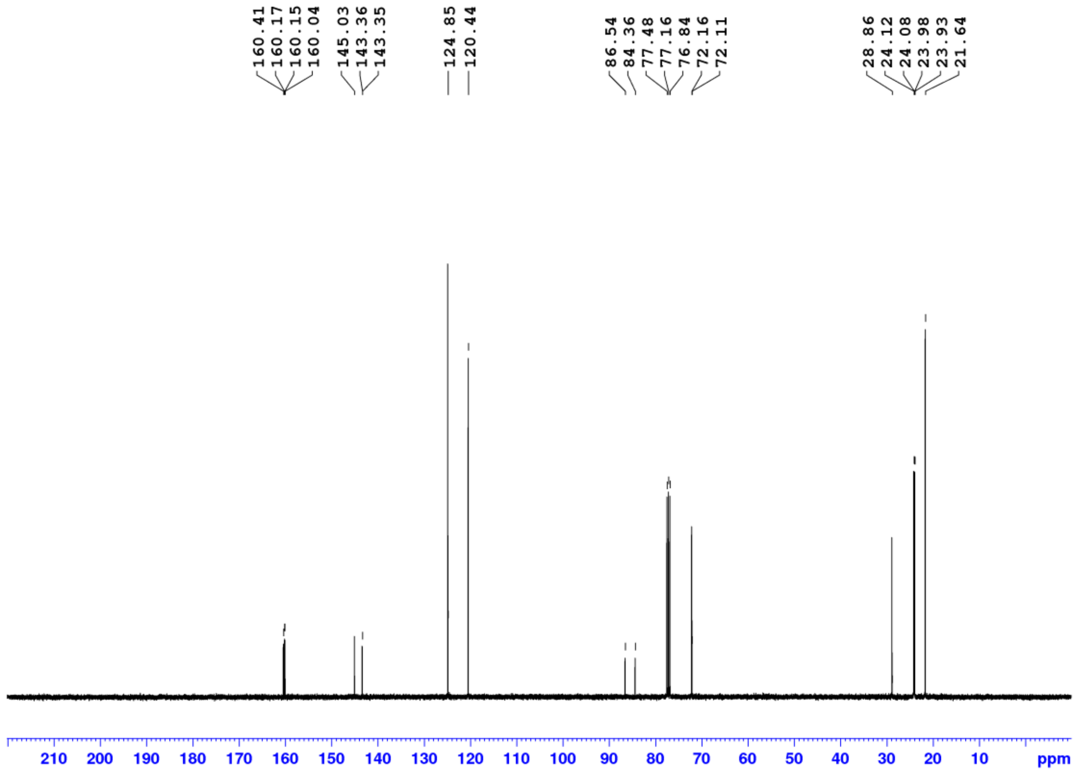

Figure S7. $^{13}C\{^{1}H\}$ NMR of H**L**^{3} ($CDCl_3$, 300 K)

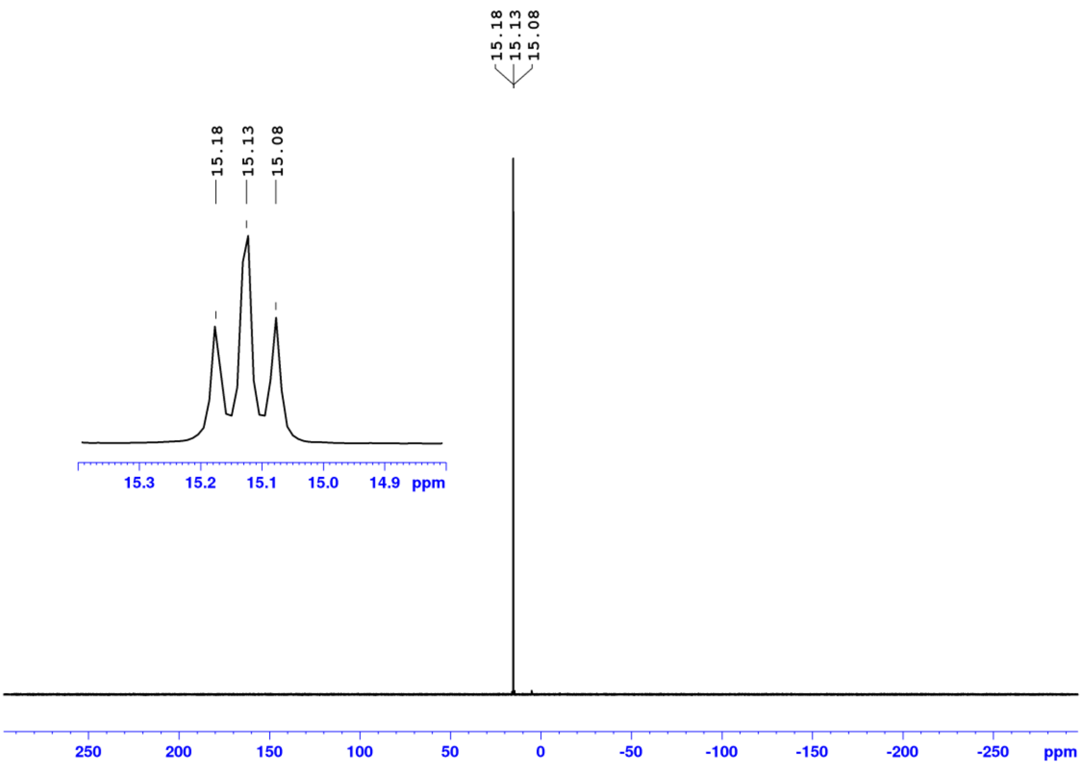

Figure S8. ^{31}P NMR of H**L**^{3} ($CDCl_3$, 300 K)

S3.5. Preparation of diisopropyl(5-hydroxy-1-(4-nitrophenyl)-3-phenyl-1H-pyrazol-4-yl)phosphonate (HL[4])

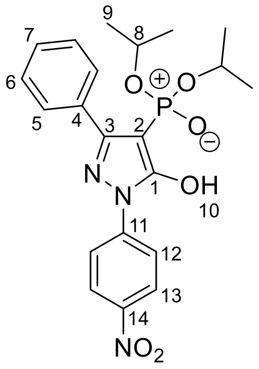

Compound **3c** (17.05 g, 92%, 45.82 mmol, 1.0 eq.) was suspended in a mixture of AcOH (42 ml), EtOH (21 ml) and H_2O (110 ml) and 4-nitrophenylhydrazine hydrochloride (10.64 g, 55.00 mmol, 1.2 eq.) were added. The reaction mixture was refluxed for 8.5 h under a N_2 atmosphere. The reaction was monitored by TLC and brought to completeness by sequential addition of an excess of 0.60 eq. followed by 0.45 eq. and 0.25 eq. of 4-nitrophenylhydrazine hydrochloride. After refluxing for 50 h, the resulting mixture was extracted with EtOAc (3 × 100 ml), washed with brine and dried over Na_2SO_4. The crude product was recrystallized from hot iso-hexane (~ 70 °C) and purified by column chromatography (iso-hexane : acetone = 3 : 2, Rf = 0.23). To remove remaining impurities, the product was dissolved in $CHCl_3$ and washed with 5 mM aqueous HCl solution (A/O = 1:1). The solvent was removed under reduced pressure to give 8.33 g of H**L**[4] as a yellow powder in 98% purity.

Yield: 8.33 g (40%); **mp.**: 139 °C; **Raman** (100 mW, 298K, in cm^{-1}): 1597 (40), 1514 (18), 1443 (17), 1384 (7), 1334 (100), 1111 (17), 1001 (5), 982 (5); **IR** (ATR, 298 K, in cm^{-1}): 3139 (vw), 2983 (vw), 2933 (vw), 2359 (vw), 1595 (w), 1539 (m), 1511 (s), 1491 (m), 1444 (w), 1419 (w), 1386 (w), 1373 (w), 1334 (s), 1264 (w), 1202 (vw), 1181 (w), 1151 (m), 1126 (w), 1104 (m), 1072 (w), 972 (vs), 902 (vw), 889 (w), 854 (s), 824 (vw), 773 (m), 748 (s), 721 (m), 691 (w), 670 (s), 611 (s), 598 (m), 546 (w), 530 (m), 492 (w), 465 (vw), 425 (w); **^{1}H NMR** ($CDCl_3$, 300K, in ppm): δ = 1.14 (6H, d, $^3J_{HH}$ = 6.2 Hz, H9a), 1.39 (6H, d, $^3J_{HH}$ = 6.2 Hz, H9b), 4.64 (2H, d sept, $^3J_{HP}$ = 7.7 Hz, $^3J_{HH}$ = 6.3 Hz, H8), 7.40-7.46 (3H, m, H7 and H6), 8.00-8.01 (2H, m, H5), 8.23-8.26 (2H, m, H12), 8.33-8.36 (2H, m, H13), 12.16 (1H, s(br), H10); **^{13}C{^{1}H} NMR** ($CDCl_3$, 300K, in ppm): δ = 23.7 (2C, d, $^3J_{CP}$ = 5 Hz, C9a), 24.1 (2C, d, $^3J_{CP}$ = 4 Hz, C9b), 72.6 (2C, d, $^2J_{CP}$ = 5 Hz, C8), 84.5 (1C, d, $^1J_{CP}$ = 213 Hz, C2), 120.7 (2C, s, C12), 125.0 (2C, s, C13), 127.7 (2C, s, C5), 128.6 (2C, s, C6), 129.5 (1C, s, C7), 131.9 (1C, d, $^3J_{CP}$ = 0.4 Hz, C4), 143.24 (1C, d, $^4J_{CP}$ = 1 Hz, C11), 145.4 (1C, s, C14), 152.4 (1C, d, $^2J_{CP}$ = 9 Hz, C3); 161.7 (1C, d, $^2J_{CP}$ = 23 Hz, C1); **^{31}P{^{1}H} NMR** ($CDCl_3$, 300K, in ppm): δ = 15.3 (s); **^{31}P NMR** ($CDCl_3$, 300K, in ppm): δ = 15.3 (t, $^3J_{PH}$ = 7.9 Hz); **Elemental analysis** for $C_{21}H_{24}N_3O_6P$, calculated: C 56.63, H 5.43, N 9.43; found: C 56.59, H 5.35, N 9.52; **ESI-MS** (m/z, [Da/e]): 444.1 $[M-H]^-$

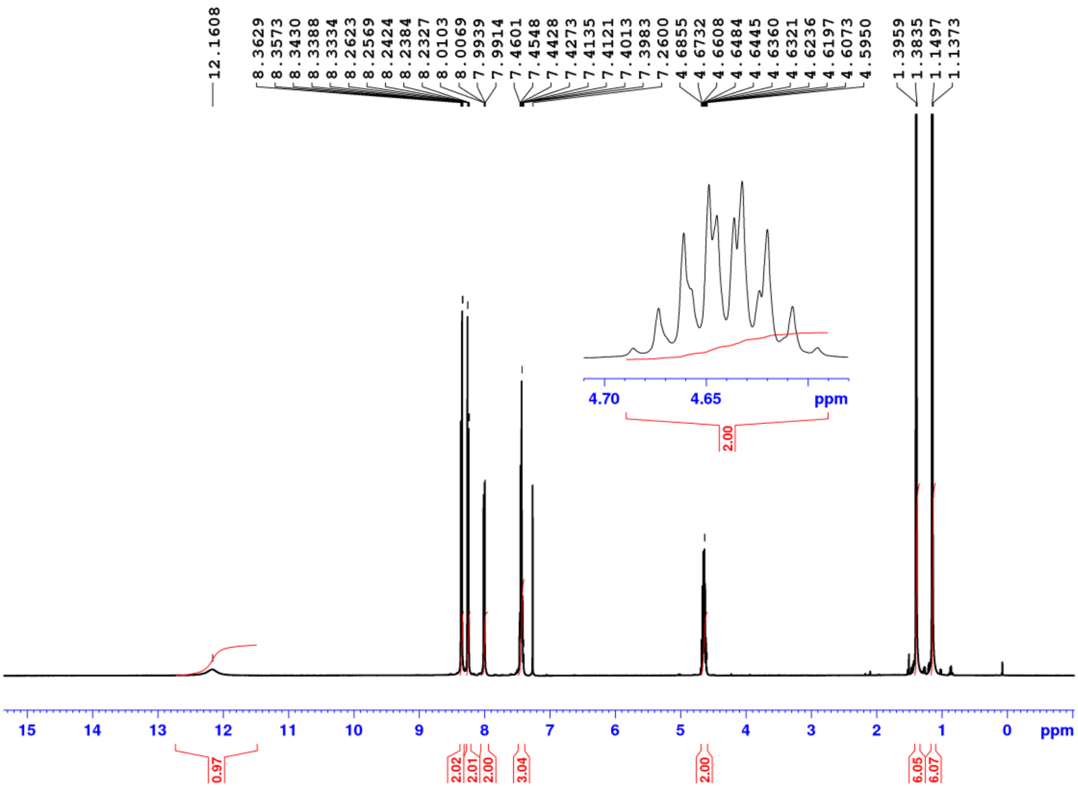

Figure S9. ^{1}H NMR of H**L**[4] ($CDCl_3$, 300 K)

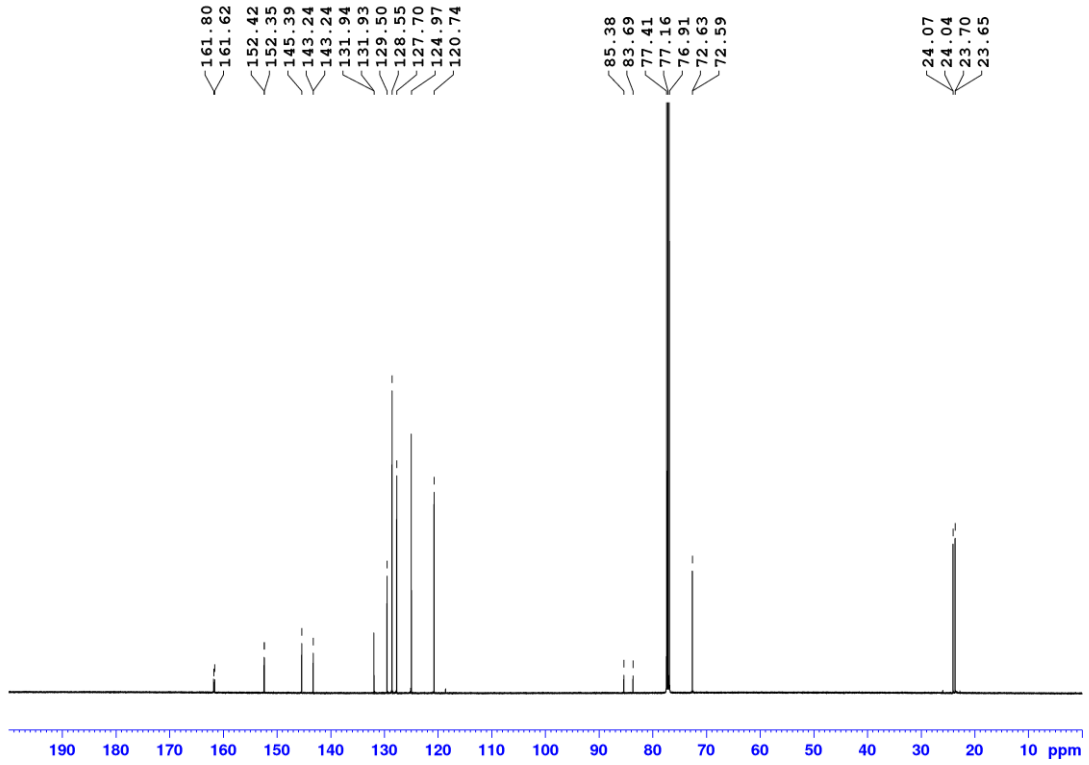

Figure S10. $^{13}C\{^{1}H\}$ NMR of H**L**[4] ($CDCl_3$, 300 K)

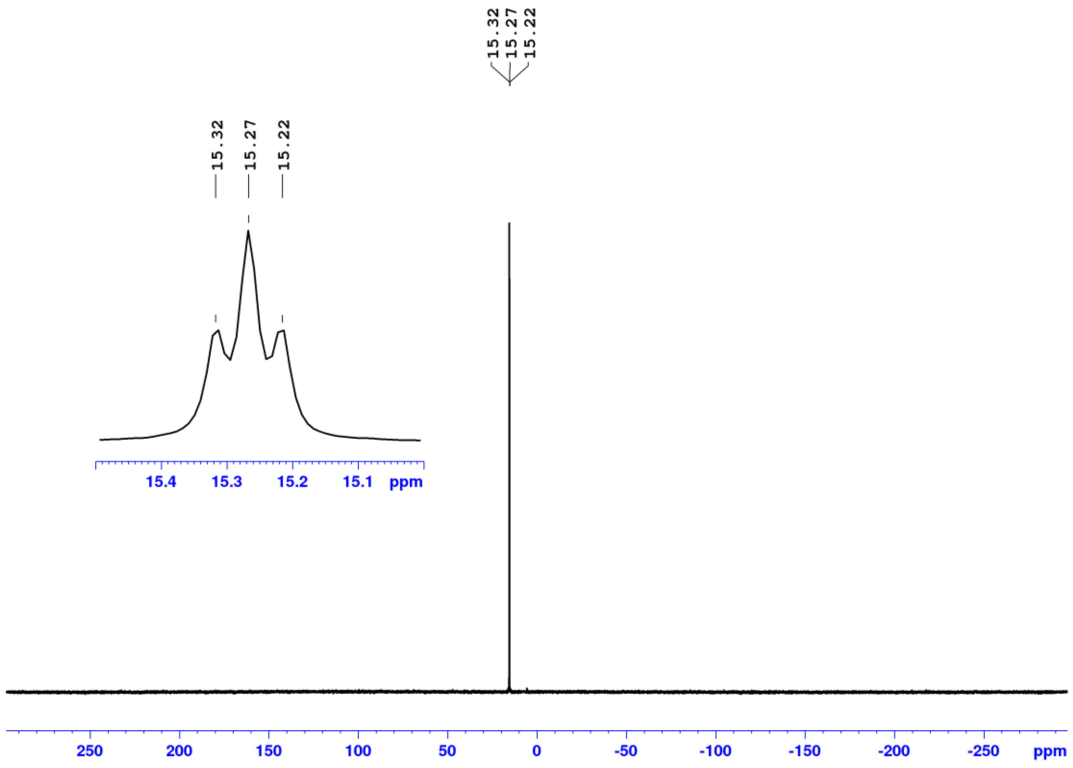

Figure S11. ^{31}P NMR of H**L**[4] ($CDCl_3$, 300 K)

S3.6. General Procedure for the Synthesis of [TBA]L^n salts

The H$\mathbf{L}^n$ ligands were dissolved in CH_2Cl_2 and tetrabutylammonium hydroxide ([TBA][OH]) (40% in CH_3OH, 1.05-1.3 eq.) was slowly added to each solution with stirring. The color of the resulting solutions changed to deep red immediately indicating that ligand deprotonation had occurred. Each reaction mixture was stirred for 0.5 - 1 h and the solvent was then removed under reduced pressure. The red solid obtained was dissolved in dry CH_2Cl_2 and the solvent was removed under reduced pressure to remove remaining water. This procedure was repeated once or twice using dry CH_2Cl_2 and once using dry CH_3CN. Single crystals suitable for X-ray diffraction analysis were obtained by diffusion of dry Et_2O into a dry CH_3CN solution (in presence of few drops of dry CH_2Cl_2) containing all the above (crude) product at -18 °C. The crystals that formed were collected by filtration, washed with dry Et_2O, and dried overnight *in vacuo.*

S3.6.1 Preparation of [TBA]L^2

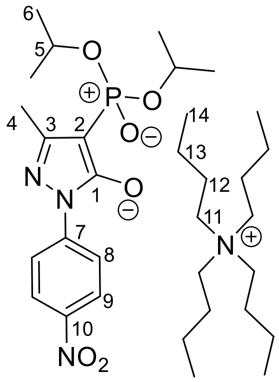

[TBA]$\mathbf{L}^2$ was prepared according to the general procedure. H$\mathbf{L}^2$ (0.62 g, 95%, 1.0 eq.) was dissolved in 12 ml of CH_2Cl_2 and tetrabutylammonium hydroxide ([TBA][OH]) (40% in CH_3OH, 1.29 g, 1.3 eq.) was slowly added with stirring. The drying process was repeated three times using dry CH_2Cl_2 (3 x 5 ml). Recrystallization of the crude material yielded red crystals suitable for X-ray analysis.

Yield: 0.51 g (53%); **mp**.: 143 °C; **Raman** (100 mW, 298K, in cm^{-1}): 1584 (11), 1521 (9), 1344 (5), 1305 (100), 1271 (18), 1122 (5), 1107 (21), 1055 (8), 1040 (9), 988 (9), 857 (6), 575 (6); **IR** (ATR, 298 K, in cm^{-1}): 2962 (w), 2924 (vw), 2874 (vw), 1616 (s), 1582 (m), 1520 (w), 1493 (m), 1481 (w), 1467 (w), 1418 (w), 1383 (w), 1362 (vw), 1345 (w), 1300 (vs), 1269 (vs), 1220 (s), 1178 (w), 1166 (w), 1139 (vw), 1105 (s), 1053 (w), 1039 (w), 984 (s), 967 (vs), 880 (m), 854 (s), 817 (w), 773 (m), 748 (s), 720 (w), 697 (w), 679 (w), 640 (vw), 630 (m), 621 (w), 573 (vs), 535 (m), 517 (w), 501 (w), 488 (w), 444 (vw); ^{1}**H NMR** (CD_3CN, 300K, in ppm): δ = 0.96 (12H, t, $^3J_{HH}$ = 7.4 Hz, H14), 1.22 (6H, d, $^3J_{HH}$ = 6.6 Hz, H6a), 1.23 (6H, d, $^3J_{HH}$ = 6.5 Hz, H6b), 1.34 (8H, sext, $^3J_{HH}$ = 7.4 Hz, H13), 1.55-1.62 (8H, m, H12), 2.16 (3H, d, $^4J_{HP}$ = 0.7 Hz, H4), 3.05-3.08 (8H, m, H11), 4.51 (2H, d sept, $^3J_{HP}$ = 8.7 Hz, $^3J_{HH}$ = 6.2 Hz, H5), 8.12-8.15 (2H, m, H8), 8.39-8.42 (2H, m, H9); ^{13}**C{^{1}H} NMR** (CD_3CN, 300K, in ppm): δ = 13.7 (4C, s, C14), 16.4 (1C, d, $^3J_{CP}$ = 1 Hz, C4), 20.3 (4C, m, C13), 24.3 (4C, s, C12), 24.4 (2C, d, $^3J_{CP}$ = 6 Hz, C6a), 24.5 (2C, d, $^3J_{CP}$ = 4 Hz, C6b), 59.3 (4C, m, C11), 68.9 (2C, d, $^2J_{CP}$ = 5 Hz, C5), 81.2 (1C, d, $^1J_{CP}$ = 223 Hz, C2), 116.8 (2C, s, C9), 125.5 (2C, s, C8), 141.5 (1C, s, C7), 148.2 (1C, s, C10), 155.9 (1C, d, $^2J_{CP}$ = 16 Hz, C3), 168.9 (1C, d, $^2J_{CP}$ = 23 Hz, C1); ^{31}**P{^{1}H} NMR** (CD_3CN, 300K, in ppm): δ = 19.5 (s); ^{31}**P NMR** (CD_3CN, 300K, in ppm): δ = 19.5 (t, $^3J_{PH}$ = 8.7 Hz); **Elemental analysis** for $C_{32}H_{57}N_4O_6P$, calculated: C 61.52, H 9.20, N 8.97; found: C 61.38, H 9.01, N 8.77; **ESI-MS** (m/z, [Da/e]): 382.1 $[M\text{-}TBA]^-$ (ESI^-); 242.4 [M-**L**]$^+$, 384.3 $[M\text{-}TBA+2H]^+$ (ESI^+).

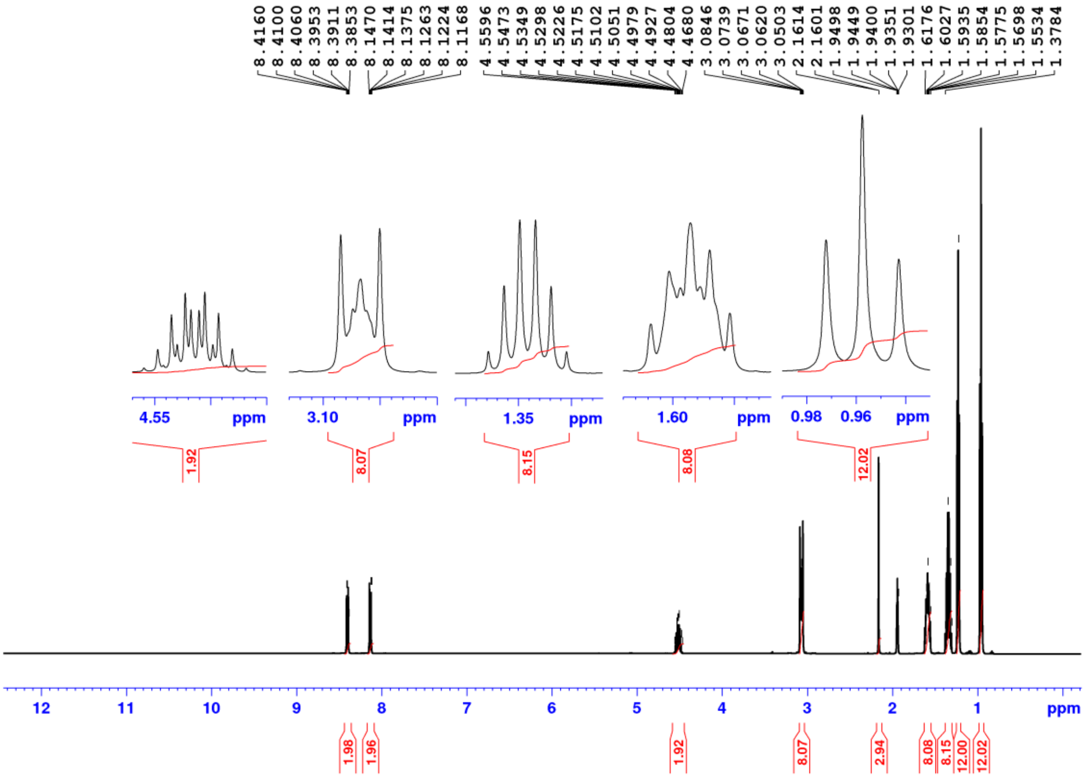

Figure S12. ^{1}H NMR of [TBA]**L**^{2} (CD_3CN, 300 K)

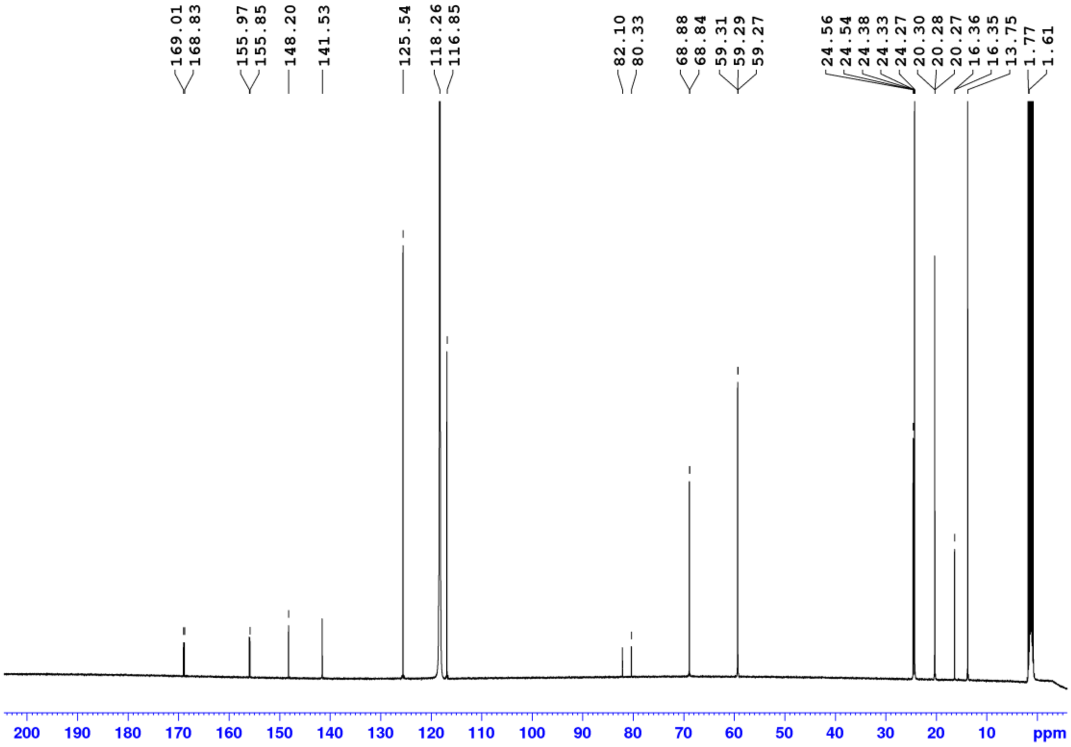

Figure S13. ^{13}C{^{1}H} NMR of [TBA]**L**^{2} (CD_3CN, 300 K)

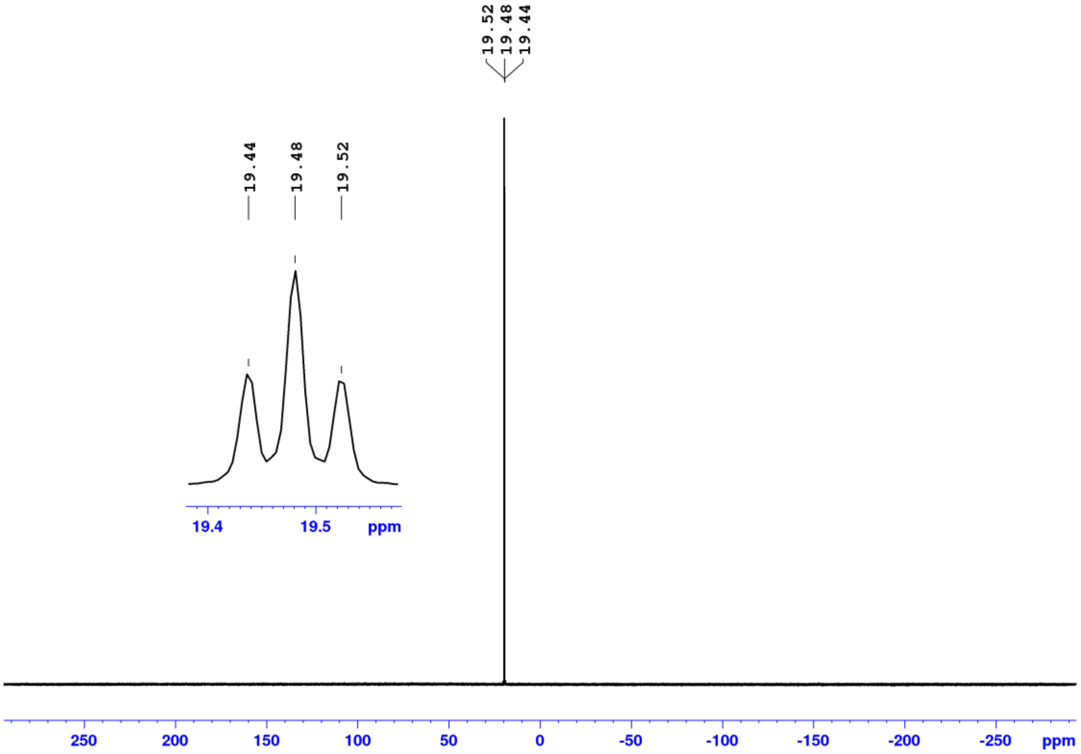

Figure S14. ^{31}P NMR of [TBA]$\mathbf{L}^2$ (CD_3CN, 300 K)

S3.6.2 Preparation of [TBA]L^3

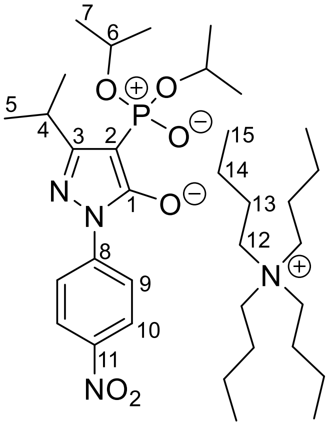

[TBA]**L**^{3} was prepared according to the general procedure. H**L**^{3} (0.17 g, 99%, 1.0 eq.) was dissolved in 3 ml CH_2Cl_2 and tetrabutylammonium hydroxide ([TBA][OH]) (40% in CH_3OH, 0.29 g, 1.05 eq.) was slowly added with stirring. The drying process was repeated twice employing dry CH_2Cl_2 (2 x 3 ml) and once employing dry CH_3CN (3 ml). Recrystallization of the crude material yielded orange crystals suitable for X-ray analysis.

Yield: 0.12 g (44%); **mp.**: 117 °C; **Raman** (100 mW, 298K, in cm^{-1}): 2927 (9), 2876 (6), 1584 (31), 1509 (15), 1346 (8), 1306 (100), 1260 (10), 1165 (5), 1129 (13), 1107 (28), 1049 (5), 987 (9), 874 (5); **IR** (ATR, 298 K, in cm^{-1}): 2963 (w), 2930 (w), 2873 (vw), 1612 (s), 1581 (m), 1493 (m), 1468 (w), 1423 (vw), 1379 (w), 1348 (vw), 1299 (vs), 1256 (s), 1218 (s), 1169 (w), 1139 (vw), 1102 (s), 1049 (vw), 969 (vs), 874 (s), 852 (s), 802 (vw), 745 (s), 664 (m), 626 (vw), 571 (vs), 535 (m), 504 (w), 491 (w), 452 (vw), 421 (vw); **^{1}H NMR** (CD_3CN, 300K, in ppm): δ = 0.96 (12H, t, $^3J_{HH}$ = 7.4 Hz, H15), 1.21-1.23 (18H, m, H5 and H7), 1.35 (8H, sext, $^3J_{HH}$ = 7.4 Hz, H14), 1.56-1.62 (8H, m, H13), 3.05-3.09 (8H, m, H12), 3.36 (1H, sept, $^3J_{HH}$ = 6.9 Hz, H4), 4.53 (2H, d sept, $^3J_{HP}$ = 8.8 Hz, $^3J_{HH}$ = 6.2 Hz, H6), 8.12-8.15 (2H, m, H10), 8.41-8.44 (2H, m, H9); **^{13}C{^{1}H} NMR** (CD_3CN, 300K, in ppm): δ = 13.7 (4C, s, C15), 20.3 (4C, m, C14), 22.4 (2C, s, C5), 24.3 (4C, s, C13), 24.4 (2C, d, $^3J_{CP}$ = 6 Hz, C7a), 24.5 (2C, d, $^3J_{CP}$ = 4 Hz, C7b), 28.4 (1C, d, $^3J_{CP}$ = 1 Hz, C4), 59.3 (4C, m, C12), 68.8 (2C, d, $^2J_{CP}$ = 5 Hz, C6), 79.6 (1C, d, $^1J_{CP}$ = 221 Hz, C2), 116.9 (2C, s, C9), 125.5 (2C, s, C10), 141.5 (1C, s, C11), 148.4 (1C, d, $^4J_{CP}$ = 1 Hz, C8), 164.9 (1C, d, $^2J_{CP}$ = 17 Hz, C3), 169.1 (1C, d, $^2J_{CP}$ = 22 Hz, C1); **^{31}P{^{1}H} NMR** (CD_3CN, 300K, in ppm): δ = 19.4 (s); **^{31}P NMR** (CD_3CN, 300K, in ppm): δ = 19.4 (t, $^3J_{PH}$ = 8.7 Hz); **Elemental analysis** for $C_{34}H_{61}N_4O_6P$, calculated: C 62.55, H 9.42, N 8.58; found: C 62.21, H 9.14, N 8.47; **ESI-MS** (m/z, [Da/e]): 410.1 [M-TBA]$^-$ (ESI$^-$), 242.4 [M-**L**]$^+$, 412.3 [M-TBA+2H]$^+$ (ESI$^+$).

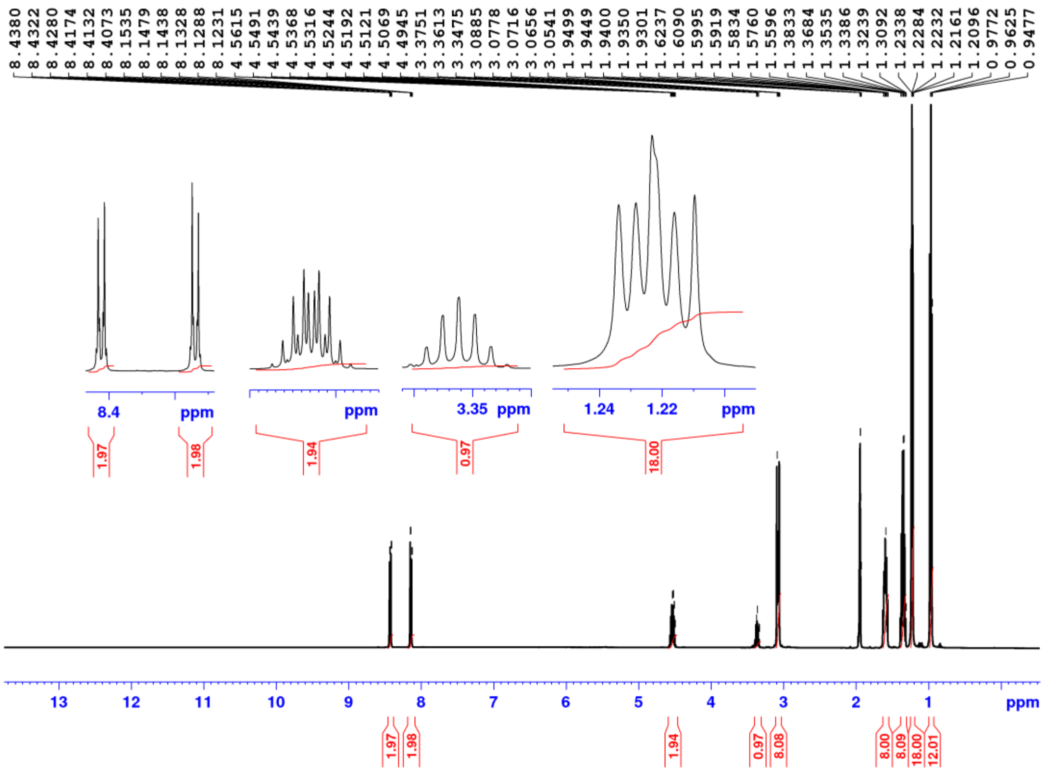

Figure S15. ^{1}H NMR of [TBA]**L**^{3} (CD_3CN, 300 K)

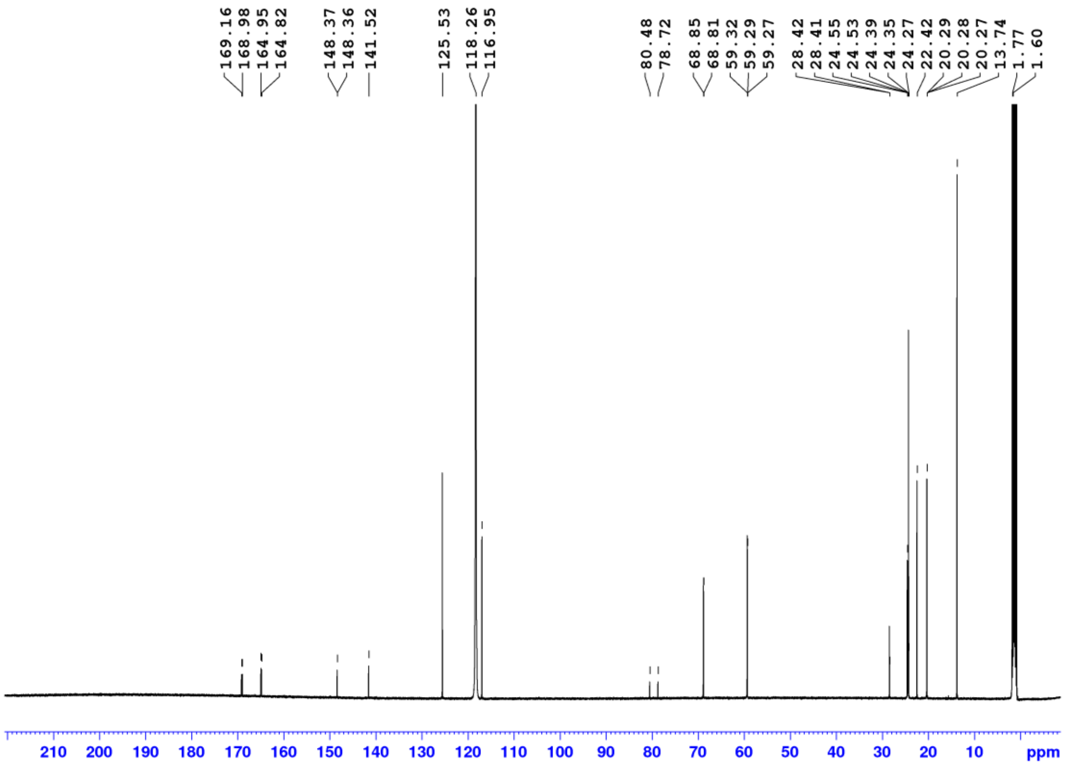

Figure S16. $^{13}C\{^{1}H\}$ NMR of [TBA]**L**^{3} (CD_3CN, 300 K)

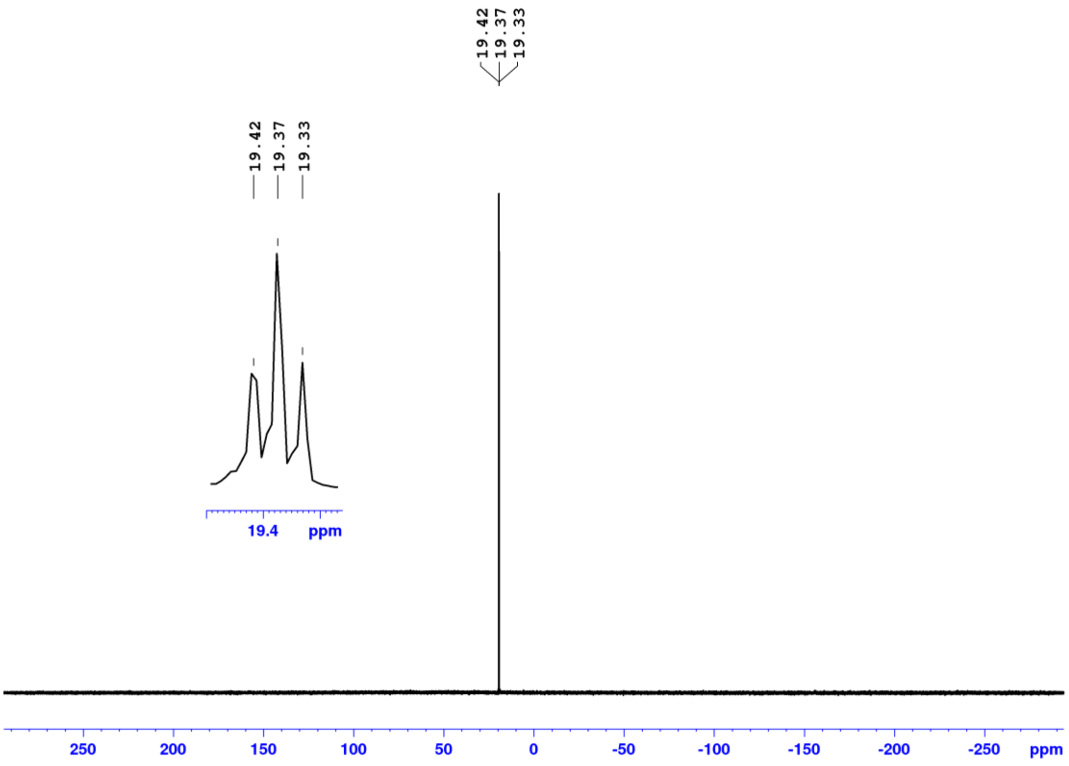

Figure S17. ^{31}P NMR of [TBA]**L**^{3} (CD_3CN, 300 K)

S3.6.3 Preparation of [TBA]L[4]

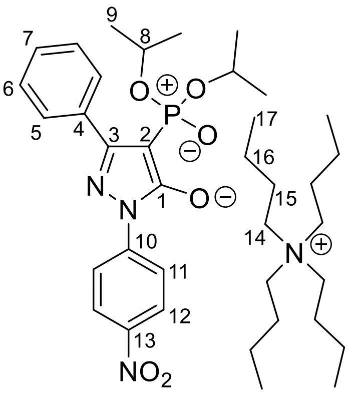

[TBA]**L**[4] was prepared according to the general procedure. H**L**[4] (0.49 g, 98%, 1.0 eq.) was dissolved in 6 ml of CH_2Cl_2 and tetrabutylammonium hydroxide ([TBA][OH]) (40% in CH_3OH, 0.93 g, 1.3 eq.) was slowly added with stirring. The drying process was repeated three times employing dry CH_2Cl_2 (3 x 5 ml). Recrystallization of the crude product yielded yellow crystals suitable for X-ray analysis.

Yield: 0.57 g (77%); **mp.**: 146 °C; **Raman** (100 mW, 298K, in cm^{-1}): 1604 (11), 1586 (23), 1507 (6), 1475 (12), 1436 (10), 1343 (21), 1313 (100), 1269 (5), 1156 (6), 1132 (15), 1111 (29), 1096 (7), 1045 (7), 1008 (6), 954 (18); **IR** (ATR, 298 K, in cm^{-1}): 2959 (w), 2931 (vw), 2873 (vw), 1624 (s), 1584 (m), 1506 (m), 1491 (m), 1474 (w), 1403 (vw), 1381 (vw), 1369 (vw), 1342 (vw), 1309 (vs), 1267 (m), 1220 (s), 1177 (w), 1144 (vw), 1108 (m), 1076 (vw), 1043 (vw), 1028 (vw), 995 (m), 968 (vs), 953 (vs), 933 (w), 880 (m), 852 (m), 801 (vw), 773 (s); **^{1}H NMR** (CD_3CN, 300K, in ppm): δ = 0.95 (12H, t, $^3J_{HH}$ = 7.4 Hz, H17), 1.16 (6H, d, $^3J_{HH}$ = 6.2 Hz, H9a), 1.21 (6H, d, $^3J_{HH}$ = 6.2 Hz, H9b), 1.33 (8H, sext, $^3J_{HH}$ = 7.4 Hz, H16), 1.54-1.60 (8H, m, H15), 3.03-3.07 (8H, m, H14), 4.51 (2H, d sept, $^3J_{HP}$ = 8.4 Hz, $^3J_{HH}$ = 6.1 Hz, H8), 7.28-7.35 (3H, m, H7 and H6), 7.78-7.80 (2H, m, H5), 8.16-8.19 (2H, m, H11), 8.50-8.53 (2H, m, H12); **^{13}C{^{1}H} NMR** (CD_3CN, 300K, in ppm): δ = 13.8 (4C, s, C17), 20.3 (4C, m, C16), 24.2 (2C, d, $^3J_{CP}$ = 6 Hz, C9a), 24.3 (2C, s, C15), 24.5 (2C, d, $^3J_{CP}$ = 4 Hz, C9b), 59.2-59.3 (4C, m, C14), 69.2 (2C, d, $^2J_{CP}$ = 5 Hz, C8), 81.0 (1C, d, $^1J_{CP}$ = 224 Hz, C2), 117.6 (2C, s, C12), 125.5 (2C, s, C11), 128.0 (2C, s, C6), 128.3 (1C, s, C7), 130.0 (2C, s, C5), 137.5 (1C, s, C4), 142.2 (1C, s, C10), 148.1 (1C, s, C13), 157.4 (1C, d, $^2J_{CP}$ = 15 Hz, C3), 169.2 (1C, d, $^2J_{CP}$ = 22 Hz, C1); **^{31}P{^{1}H} NMR** (CD_3CN, 300K, in ppm): δ = 18.4 (s); **^{31}P NMR** (CD_3CN, 300K, in ppm): δ = 18.4 (t, $^3J_{PH}$ = 8.3 Hz); **Elemental analysis** for $C_{37}H_{59}N_4O_6P$, calculated: C 64.70, H 8.66, N 8.16; found: C 64.28, H 8.41, N 8.11; **ESI-MS** (m/z, [Da/e]): 444.1 $[M\text{-}TBA]^-$ (ESI^-), 241.9 [M-**L**]$^+$ (ESI^+).

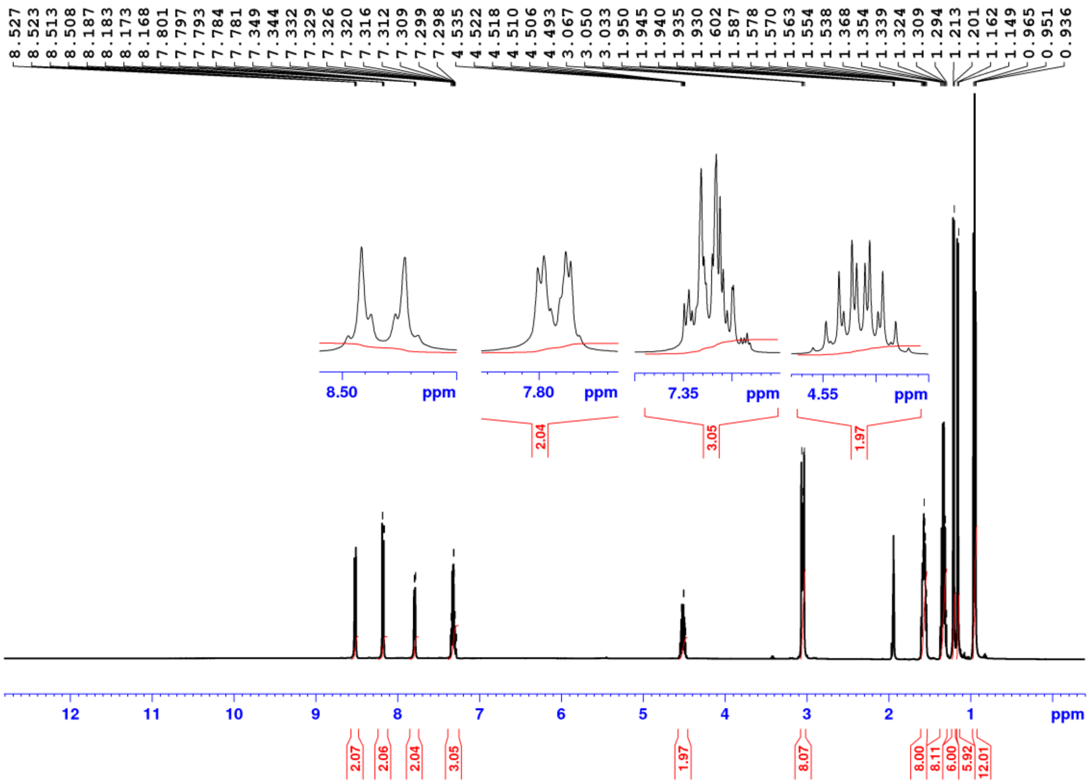

Figure S18. ^{1}H NMR of [TBA]**L**[4] (CD_3CN, 300 K)

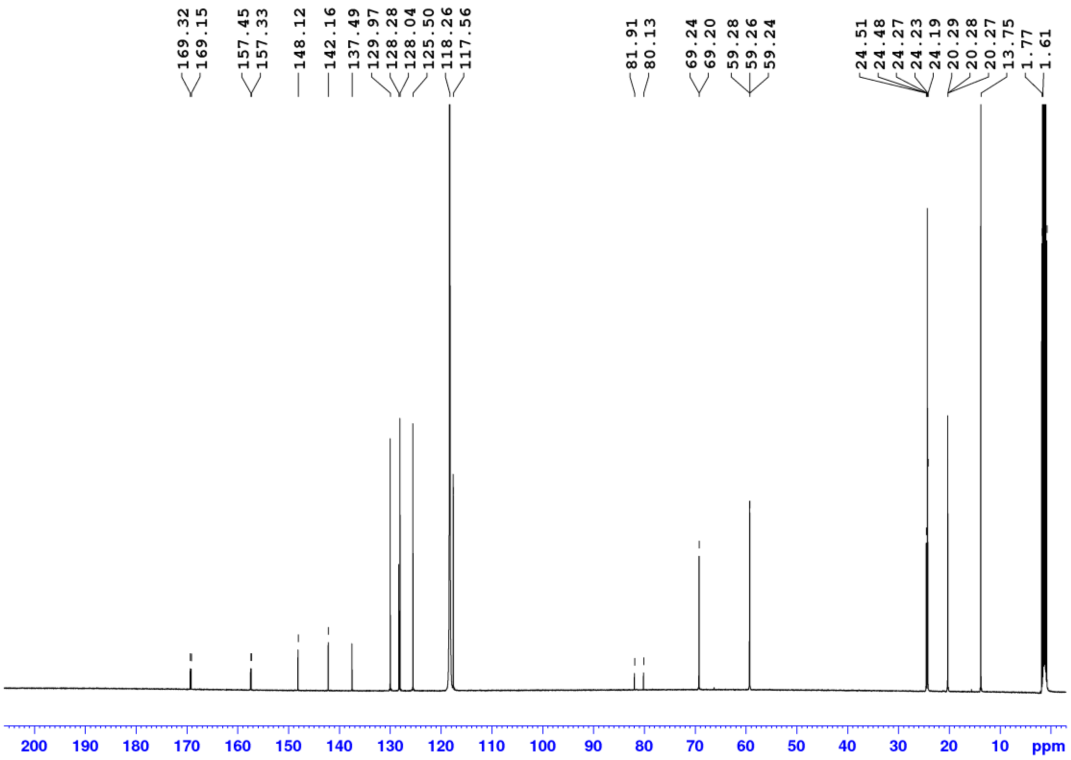

Figure S19. $^{13}C\{^{1}H\}$ NMR of [TBA]**L**[4] (CD_3CN, 300 K)

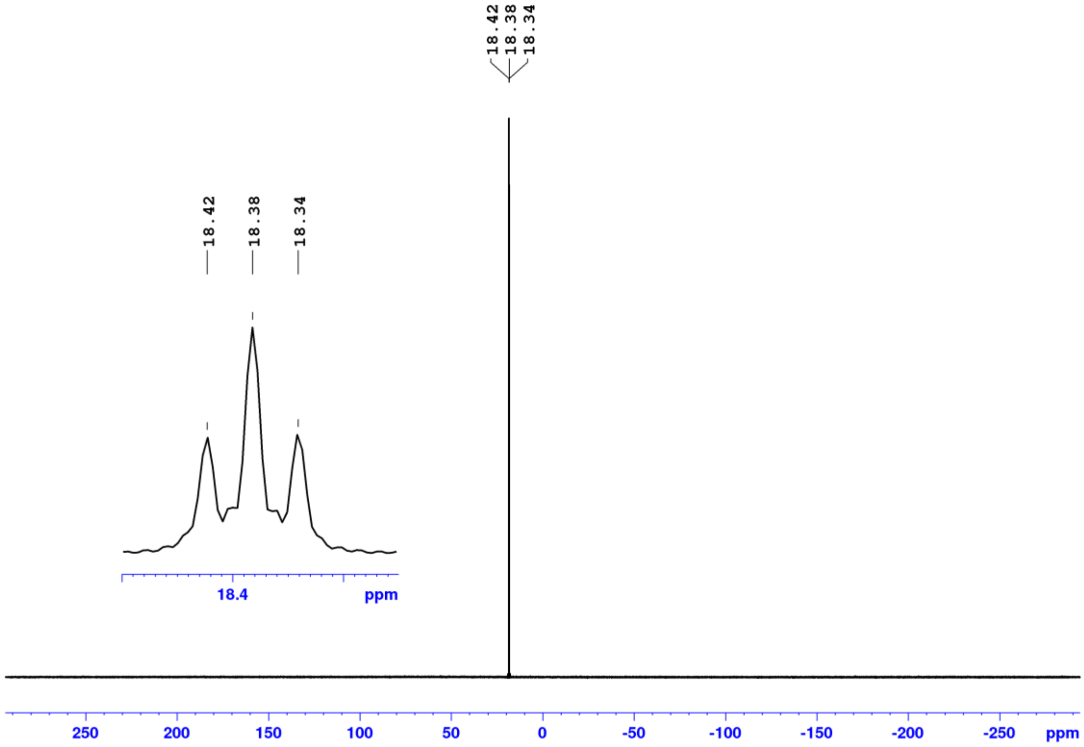

Figure S20. ^{31}P NMR of [TBA]**L**[4] (CD_3CN, 300 K)

S4. Synthesis of lithium complexes

S4.1. General procedure for the synthesis of acetonitrile-solvated lithium complexes

To prepare the [Li_2(**L**^{n})$_2$(CH_3CN)$_2$] complexes, H**L**^{n} were dissolved in CH_2Cl_2 and LiOH·H_2O (1.0 eq.) were added. The reaction mixture was stirred at 50 °C for 4 h. The cooled reaction mixture was then filtered and the solvent was removed under reduced pressure. To remove remaining water the yellow product was dissolved in dry CH_2Cl_2 and the solvent was removed under reduced pressure. The procedure was repeated once using dry CH_2Cl_2 and twice using dry CH_3CN and the solid obtained was dried *in vacuo.* Single crystals suitable for X-ray diffraction analysis were obtained by diffusion of dry Et_2O into a solution containing of all the crude product in dry CH_3CN or in a mixture of dry CH_3CN and CH_2Cl_2 at -30 °C in the glove-box.

S4.1.1. Preparation of [Li_2(L^2)$_2$(CH_3CN)$_2$] (4)

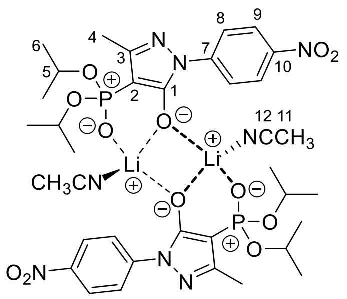

[Li_2(**L**^{2})$_2$(CH_3CN)$_2$] (**4**) was prepared according to the general procedure. H**L**^{2} (403.6 mg, 1.0 mmol, 1.0 eq.) was dissolved in 15 ml of CH_2Cl_2 and LiOH·H_2O (42.4 mg, 1.0 mmol, 1.0 eq.) were added. The drying process was repeated twice with dry CH_2Cl_2 (2 x 5 ml) and twice with dry CH_3CN (2 × 5 ml). Recrystallization of the crude material yielded yellow crystals suitable for X-ray analysis.

Yield: 123.4 mg (29%); **mp**.: 248 °C; **Raman** (100 mW, 298 K, in cm^{-1}): 2926 (6), 1601 (23), 1584 (16), 1326 (100), 1289 (9), 1109 (23), 1060 (5); **IR** (ATR, 298 K, in cm^{-1}): 3572 (vw), 3134 (vw), 2979 (vw), 2925 (vw), 1650 (vw), 1605 (s), 1563 (s), 1513 (s), 1468 (vw), 1422 (m), 1387 (w), 1372 (w), 1349 (w), 1323 (vs), 1303 (m), 1289 (s), 1210 (m), 1177 (w), 1140 (vw), 1109 (m), 1060 (vw), 1002 (s), 955 (vs), 887 (m), 851 (m), 836 (m), 778 (s), 750 (s), 681 (w), 637 (w), 626 (w), 590 (vs), 532 (w), 502 (vw), 460 (w), 422 (w); **^{1}H NMR** (CD_2Cl_2, 300K, in ppm): δ = 1.15 (12H, d, $^3J_{HH}$ = 6.1 Hz, H6a), 1.24 (12H, d, $^3J_{HH}$ = 5.8 Hz, H6b), 1.93 (6H, s, H11), 2.16 (6H, s, H4), 4.46-4.52 (4H, m, H5), 8.04 (8H, s, H8 and H9); **^{13}C{^{1}H} NMR** (CD_2Cl_2, 300K, in ppm): δ = 2.0 (2C, s, C11), 14.8 (2C, s, C4), 23.9 (4C, d, $^3J_{CP}$ = 4 Hz, C6a), 24.1 (4C, d, $^3J_{CP}$ = 4 Hz, C6b), 71.3 (4C, s, C5), 83.5 (2C, d, $^3J_{CP}$ = 228 Hz, C2), 117.2 (2C, s, C12), 120.0 (4C, s, C9), 124.9 (4C, s, C8), 143.8 (2C, s, C7), 145.4 (2C, s, C10), 152.2 (2C, d, $^2J_{CP}$ = 12 Hz, C3), 168.0 (2C, d, $^2J_{CP}$ = 25 Hz, C1); **^{31}P{^{1}H} NMR** (CD_2Cl_2, 300K, in ppm): δ = 19.3 (s); **^{31}P NMR** (CD_2Cl_2, 300K, in ppm): δ = 19.3 (s); **^{7}Li NMR** (CD_2Cl_2, 300K, in ppm): δ = 0.57 (s); **Elemental analysis**: Complex **4** readily lost the solvated CH_3CN and the results are consistent with $C_{39}H_{58.5}Li_2N_{6.5}O_{13.5}P_2$ (Li_2(**L**^{2})$_2$(0.5CH_3CN)(1.5Et_2O)): calculated: C 51.46, H 6.48, N 10.00; found: C 51.60, H 6.23, N 9.59; **ESI-MS** (m/z, [Da/e]): 382.1 [M-2Li-**L**-2CH_3CN]$^-$, 771.1 [M-Li-2CH_3CN]$^-$, 1160.4 [M-2CH_3CN+**L**]$^-$.

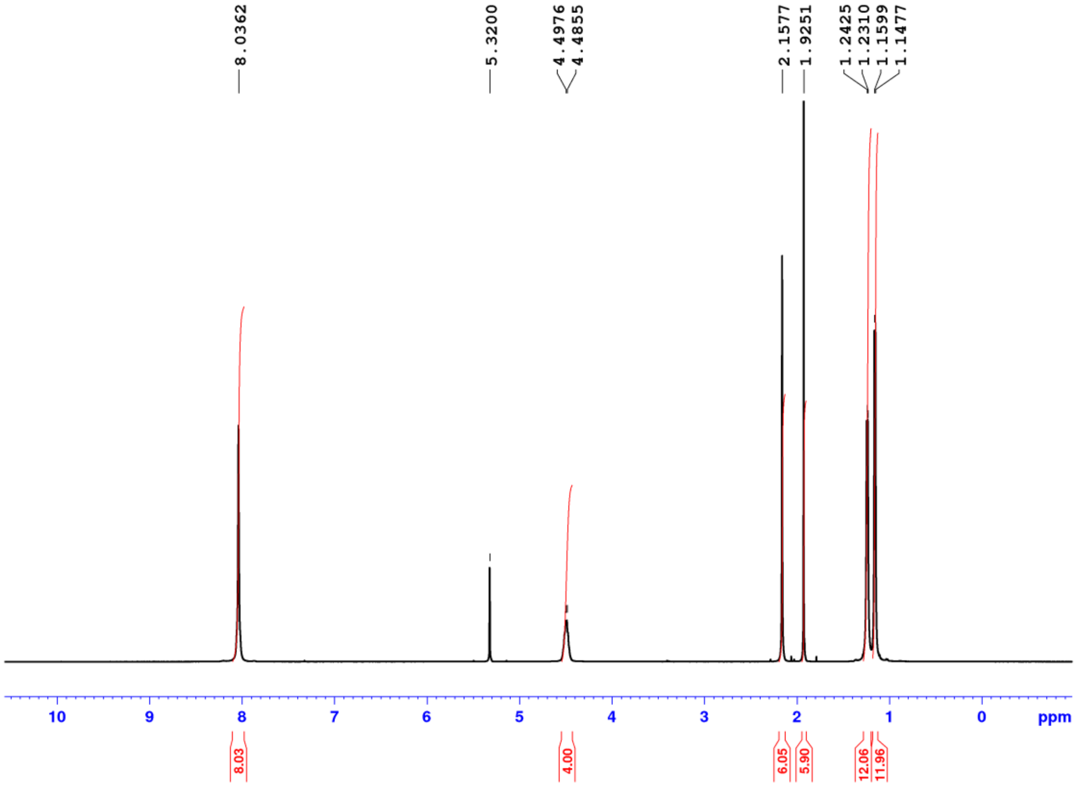

Figure S21. ^{1}H NMR of dissolved single crystals of $[Li_2(\mathbf{L}^2)_2(CH_3CN)_2]$ (CD_2Cl_2, 300 K)

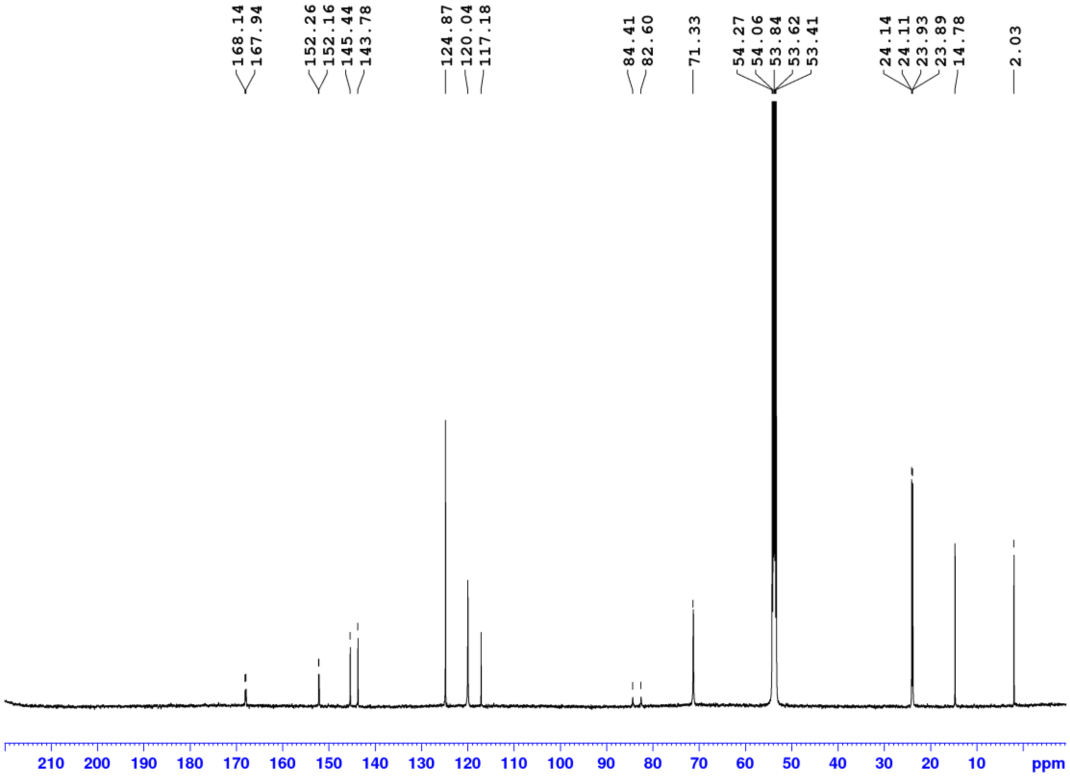

Figure S22. ^{13}C{^{1}H} NMR of the dissolved single crystals of $[Li_2(\mathbf{L}^2)_2(CH_3CN)_2]$ (CD_2Cl_2, 300 K)

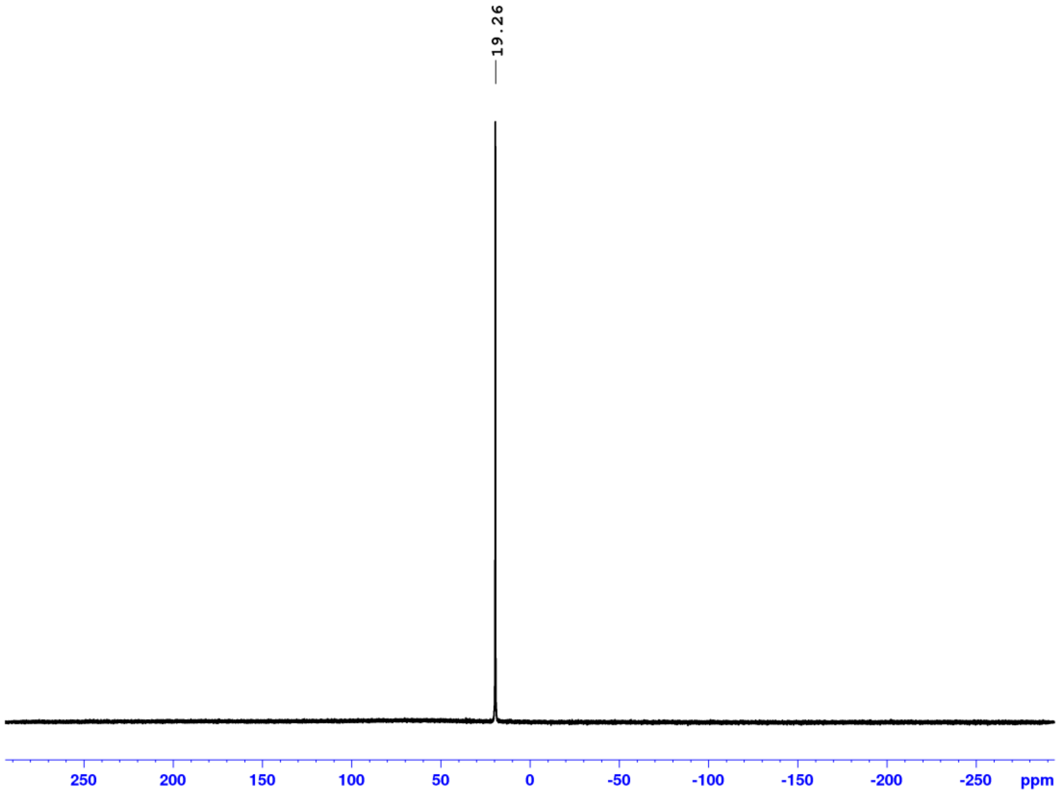

Figure S23. ^{31}P NMR of the dissolved single crystals of $[Li_2(\mathbf{L}^2)_2(CH_3CN)_2]$ (CD_2Cl_2, 300 K)

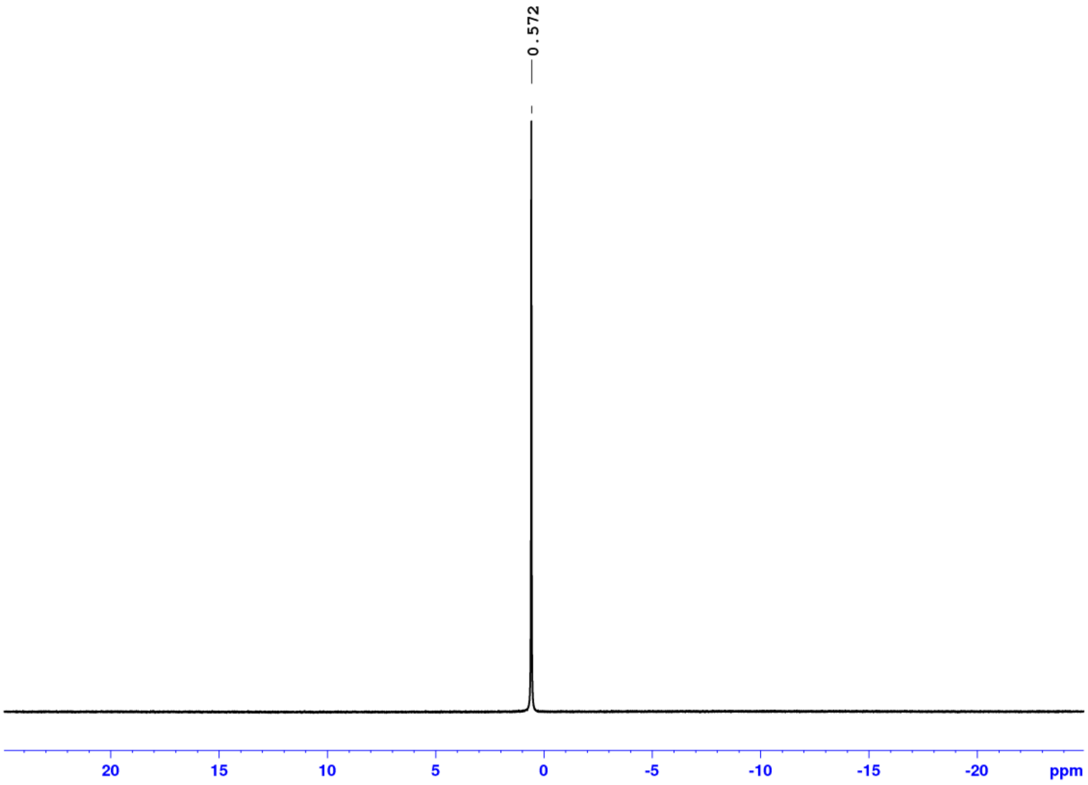

Figure S24. ^{7}Li NMR of the dissolved single crystals of $[Li_2(\mathbf{L}^2)_2(CH_3CN)_2]$ (CD_2Cl_2, 300 K)

S4.1.2. Preparation of [$Li_2(L^3)_2(CH_3CN)_2$] (5)

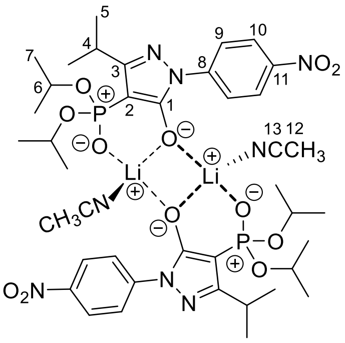

[Li_2(**L**^{3})$_2$(CH_3CN)$_2$] (**5**) was prepared according to the general procedure. H**L**^{3} (143.5 mg, 0.35 mmol, 1.0 eq.) was dissolved in 5 ml CH_2Cl_2 and LiOH·H_2O (15.1 mg, 0.35 mmol, 1.0 eq.) were added. The reaction mixture was stirred at 50 °C for 2 h before an additional 10 ml of CH_2Cl_2 were added. Upon heating for a further 2 h, all solids had dissolved. The drying process was repeated twice with CH_2Cl_2 (2 x 5 ml) and twice with dry CH_3CN (2 × 5 ml). Recrystallization of the crude material yielded yellow crystals suitable for X-ray analysis.

Yield: 50.7 mg (32%); **mp**.: 260 °C; **Raman** (100 mW, 298 K, in cm^{-1}): 2983 (5), 2928 (7), 1597 (28), 1580 (15), 1510 (6), 1326 (100), 1275 (11), 1167 (7), 1112 (27), 1007 (5); **IR** (ATR, 298 K, in cm^{-1}): 2976 (w), 2934 (vw), 2871 (vw), 2646 (vw), 2263 (vw), 1594 (m), 1556 (m), 1504 (s), 1474 (w), 1426 (w), 1374 (w), 1351 (w), 1324 (vs), 1269 (m), 1198 (m), 1174 (w), 1141 (w), 1109 (m), 1088 (m), 1079 (m), 1035 (w), 984 (vs), 934 (w), 893 (w), 879 (w), 853 (s), 824 (vw), 812 (vw), 762 (s), 750 (s), 691 (w), 668 (w), 591 (s), 533 (w), 507 (w), 474 (w), 454 (vw), 415 (vw); **^{1}H NMR** (CD_2Cl_2, 300K, in ppm): δ = 1.16 (12H, d, $^3J_{HH}$ = 4.6 Hz, H7a), 1.25-1.26 (24H, m, H5 and H7b), 1.91 (6H, s, H12), 2.95 (2H, sept, $^3J_{HH}$ = 6.4, H4), 4.53 (4H, s, H6), 8.02-8.04 (8H, m, H9 and H10); **^{13}C{^{1}H} NMR** (CD_2Cl_2, 300K, in ppm): 2.0 (2C, s, C12), 22.0 (4C, s, C5), 24.0 (4C, d, $^3J_{CP}$ = 5 Hz, C7a), 24.0 (4C, d, $^3J_{CP}$ = 5 Hz, C7b), 28.4 (2C, s, C4), 71.3 (4C, s, C6), 82.4 (2C, d, $^1J_{CP}$ = 230 Hz, C2), 117.2 (2C, s, C13), 120.3 (4C, s, C10), 124.8 (4C, s, C9), 143.7 (2C, s, C8), 145.7 (2C, s, C11), 161.2 (2C, d, $^2J_{CP}$ = 13 Hz, C3), 167.6 (2C, d, $^2J_{CP}$ = 26 Hz, C1); **^{31}P{^{1}H} NMR** (CD_2Cl_2, 300K, in ppm): δ = 19.6 (s); **^{31}P NMR** (CD_2Cl_2, 300K, in ppm): δ = 19.6 (s); **^{7}Li NMR** (CD_2Cl_2, 300K, in ppm): δ = 0.57 (s); **Elemental analysis** for $C_{40}H_{56}Li_2N_8O_{12}P_2$ (Li_2(**L**^{3})$_2$(CH_3CN)$_2$): calculated: C 52.41, H 6.16, N 12.22; found: C 52.09, H 5.98, N 12.01; **ESI-MS** (m/z, [Da/e]): 410.1 [M-2Li-**L**-2CH_3CN]$^-$, 827.2 [M-Li-2CH_3CN]$^-$, 1244.3 [M-2CH_3CN+**L**]$^-$ (ESI$^-$); 412.2 [M-2Li-**L**-2CH_3CN+2H]$^+$ (ESI$^+$).

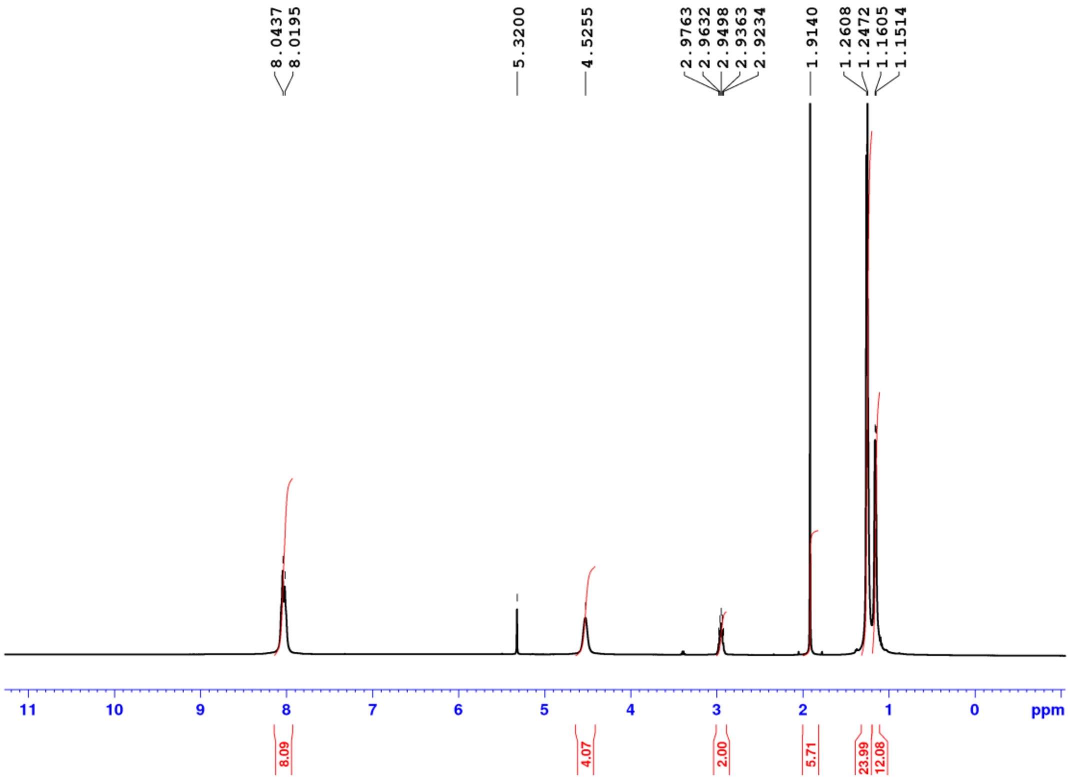

Figure S25. ^{1}H NMR of [Li_2(**L**^{3})$_2$(CH_3CN)$_2$] (CD_2Cl_2, 300 K)

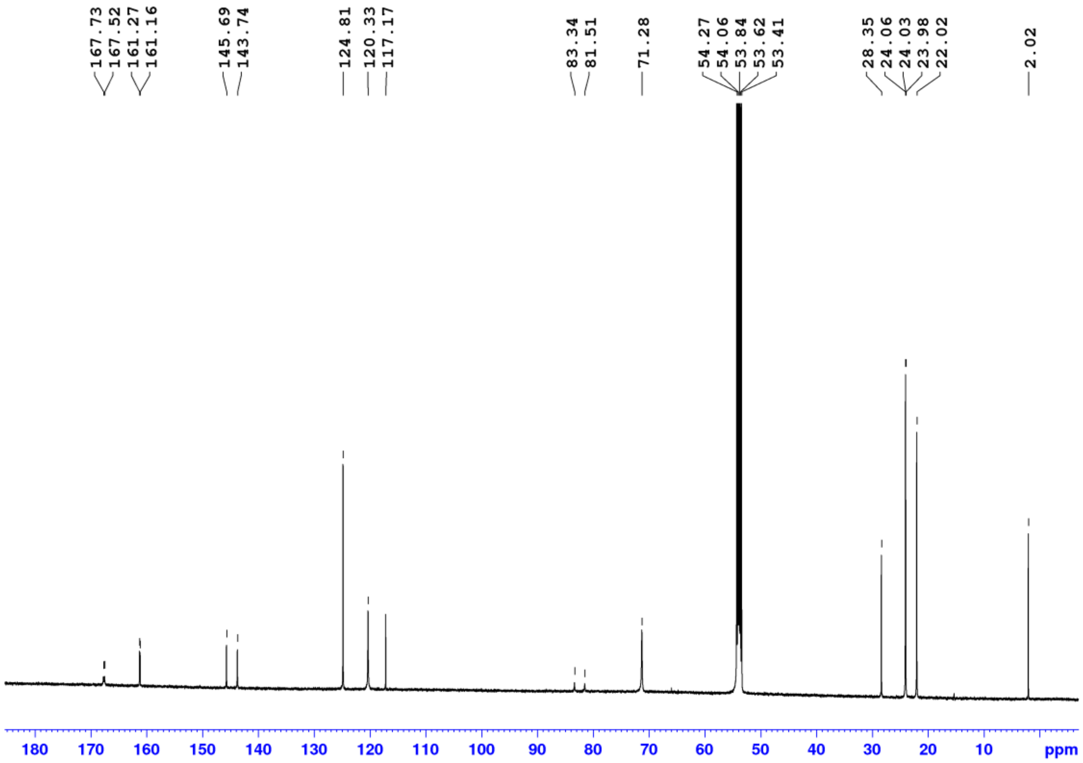

Figure S26. $^{13}C\{^{1}H\}$ NMR of $[Li_2(\mathbf{L}^3)_2(CH_3CN)_2]$ (CD_2Cl_2, 300 K)

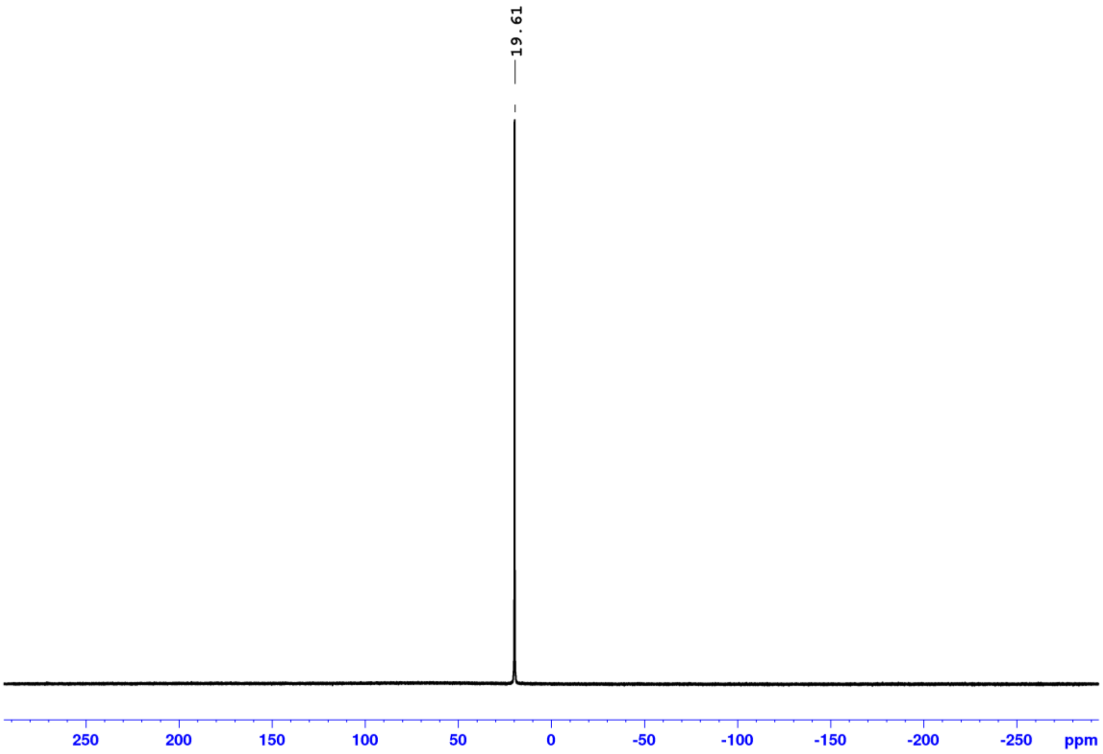

Figure S27. ^{31}P NMR of $[Li_2(\mathbf{L}^3)_2(CH_3CN)_2]$ (CD_2Cl_2, 300 K)

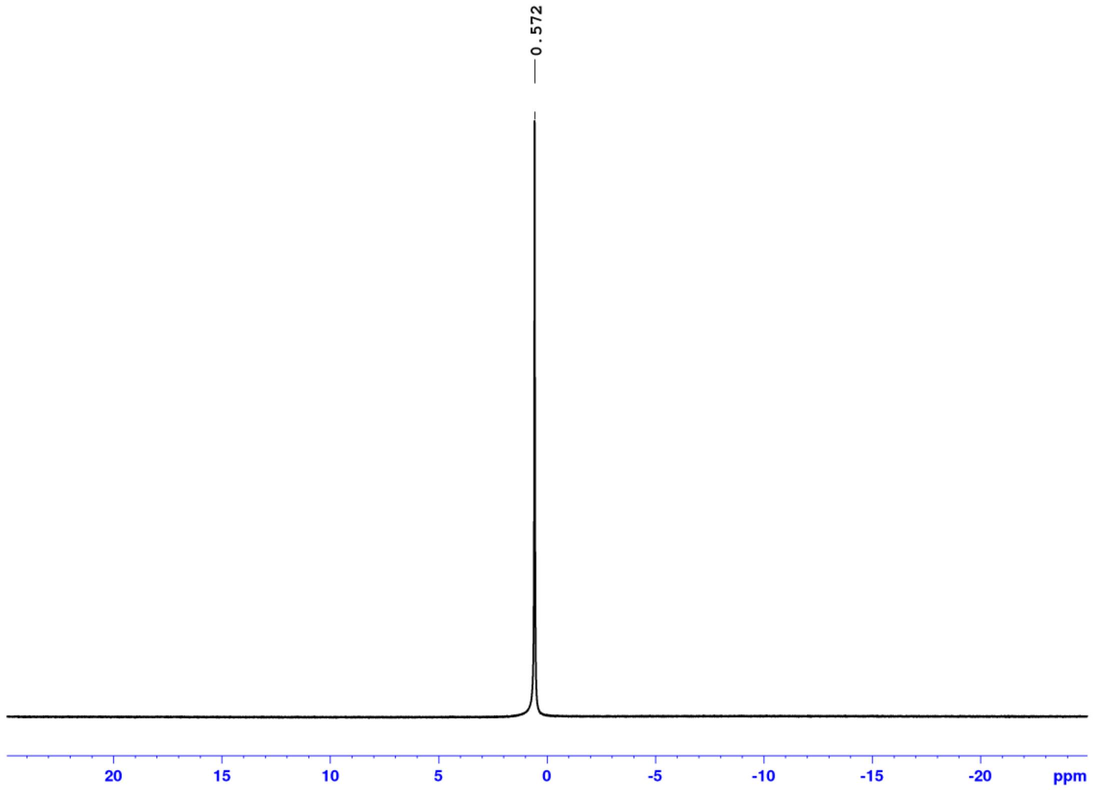

Figure S28. ^{7}Li NMR of $[Li_2(\mathbf{L}^3)_2(CH_3CN)_2]$ (CD_2Cl_2, 300 K)

S4.1.3. Preparation of [Li_2(L^4)$_2$(CH_3CN)$_2$] (6)

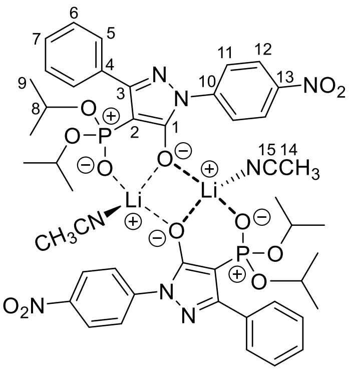

[Li_2(L^4)$_2$(CH_3CN)$_2$] (**6**) was prepared according to the general procedure. H**L**[4] (182.6 mg, 0.4 mmol, 1.0 eq.) was dissolved in 5 ml of CH_2Cl_2 and LiOH·H_2O (18.5 mg, 0.4 mmol, 1.0 eq.) were added. The reaction mixture was stirred at 50 °C for 2 h before an additional 10 ml of CH_2Cl_2 were added. Upon heating for a further 2 h, all solids had dissolved. The drying process was repeated twice with dry CH_2Cl_2 (2 × 5 ml) and twice with dry CH_3CN (2 × 5 ml). The resulting solid was dissolved in a mixture of dry CH_3CN (1 ml) and dry CH_2Cl_2 (0.5 ml), then 20 ml of dry Et_2O were slowly added with stirring to give a yellow precipitate. After stirring for 30 min, the precipitate was filtered off and dried *in vacuo*. Integration of the ^{1}H NMR (Figure. S29), of the crude material indicates the presence of 1.0 eq. of both CH_3CN and Et_2O which is also consistent with elemental analysis $C_{48}H_{59}Li_2N_7O_{13}P_2$ ([Li_2(**L**[4])$_2$(CH_3CN)(Et_2O)]): calculated: C 56.64, H 5.84, N 9.63; found: C 56.61, H 5.47, N 9.52.

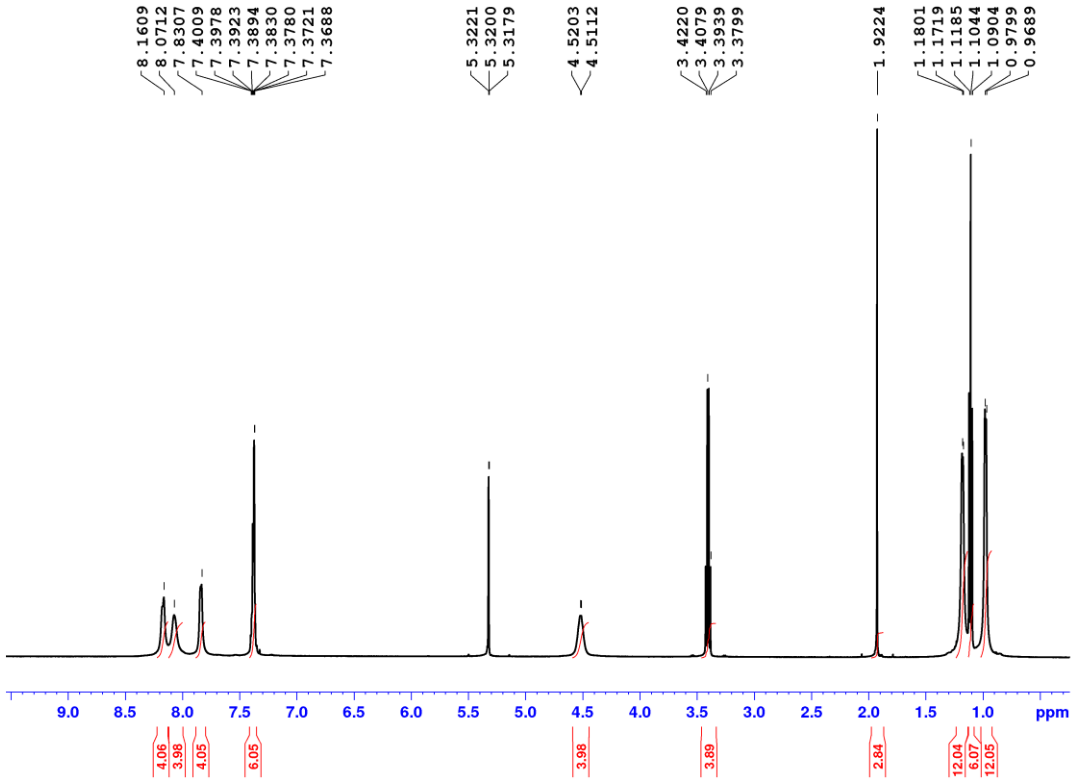

Figure S29. ^{1}H NMR of [Li_2(**L**[4])$_2$(CH_3CN)(Et_2O)] (CD_2Cl_2, 300 K)

Recrystallization of the precipitate obtained resulted in yellow crystals of [Li_2(**L**[4])$_2$(CH_3CN)$_2$] that were suitable for X-ray analysis.

Yield: 90.8 mg (44%); **mp.**: 275 °C; **Raman** (100 mW, 298 K, in cm^{-1}): 1598 (28), 1580 (8), 1510 (7), 1475 (8), 1434 (15), 1330 (100), 1171 (9), 1113 (17), 974 (9); **IR** (ATR, 298 K, in cm^{-1}): 2975 (vw), 2929 (vw), 2305 (vw), 2273 (vw), 1596 (m), 1554 (m), 1511 (m), 1496 (m), 1472 (w), 1402 (w), 1387 (vw), 1374 (vw), 1358 (vw), 1325 (s), 1278 (w), 1210 (m), 1177 (w), 1161 (vw), 1140 (w), 1106 (m), 1080 (w), 1011 (m), 967 (vs), 898 (w), 883 (w), 854 (s), 818 (w), 766 (m), 743 (s), 696 (m), 673 (m), 629 (w), 616 (m), 554 (vw), 537 (w), 512 (w), 498 (vw), 473 (vw), 413 (w); **^{1}H NMR** (CD_2Cl_2, 300K, in ppm): δ = 0.97 (12H, d, $^3J_{HH}$ = 6.1 Hz, H9a), 1.17 (12H, d, $^3J_{HH}$ = 5.9 Hz, H9b), 1.93 (6H, s, H14), 4.48-4.54 (4H, m, H8), 7.34-7.40 (6H, m, H7 and H6), 7.83-7.85 (4H, m, H5), 8.10 (4H, d, $^3J_{HH}$ = 8.3 Hz, H11), 8.18 (4H, d, $^3J_{HH}$ = 8.8 Hz, H12); **^{13}C{^{1}H} NMR** (CD_2Cl_2, 300K, in ppm): δ = 2.1 (2C, s, C14), 23.7 (4C, d, $^3J_{CP}$ = 5 Hz, C9a), 24.0 (4C, d, $^3J_{CP}$ = 4 Hz, C9b), 71.7 (4C, d, $^2J_{CP}$ = 5 Hz, C8), 82.8 (2C, d, $^1J_{CP}$ = 228 Hz, C2), 117.2 (2C, s, C15), 120.9 (4C, s (br), C12), 124.9 (4C, s, C11), 128.2 (4C, s, C6), 128.8 (4C, s, C5), 128.8 (2C, s, C7), 134.5 (2C, s, C4), 144.3 (2C, s, C10), 145.3 (2C, s, C13), 154.1 (2C, d, $^2J_{CP}$ = 12 Hz, C3), 168.5 (2C, d, $^2J_{CP}$ = 24 Hz, C1); **^{31}P{^{1}H} NMR** (CD_2Cl_2, 300K, in ppm): δ = 18.9 (s); **^{31}P NMR** (CD_2Cl_2, 300K,

in ppm): δ = 18.9 (s); **^{7}Li NMR** (CD_2Cl_2, 300K, in ppm): δ = 0.69 (s); **ESI-MS** (m/z, [Da/e]): 444.0 [M-2Li-**L**-2CH$_3$CN]$^-$, 895.3 [M-Li-2CH$_3$CN]$^-$ (ESI$^-$); 446.3 [M-2Li-**L**-2CH$_3$CN+2H]$^+$ (ESI$^+$).

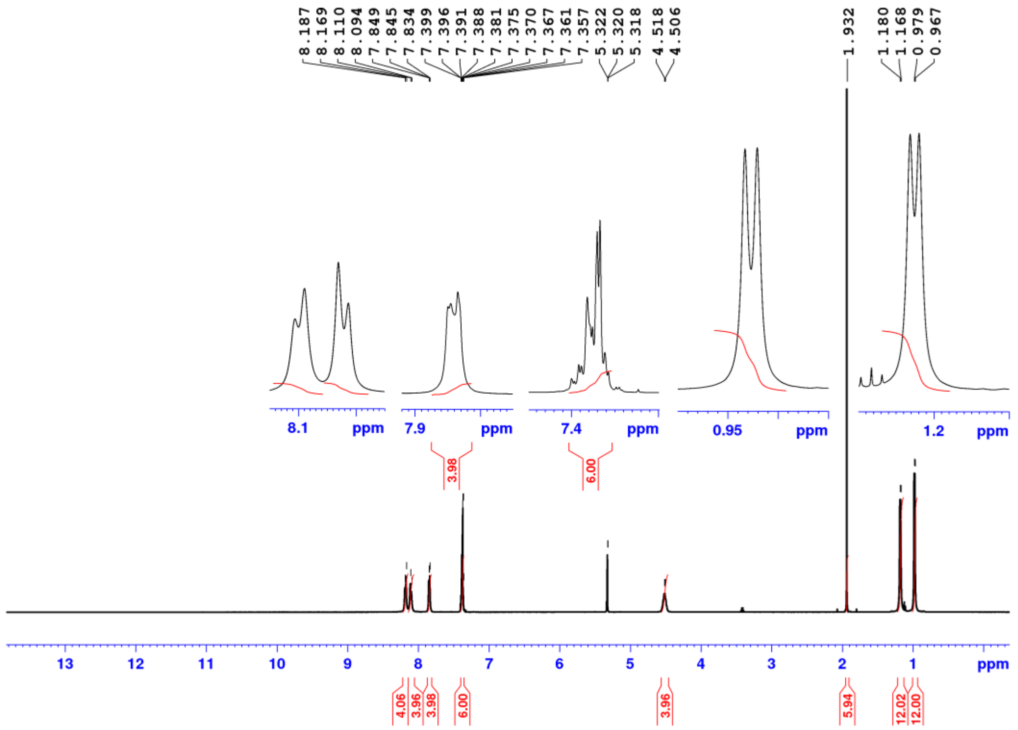

Figure S30. ^{1}H NMR of the dissolved single crystals of [Li$_2$(**L**^{4})$_2$(CH$_3$CN)$_2$] (CD_2Cl_2, 300 K)

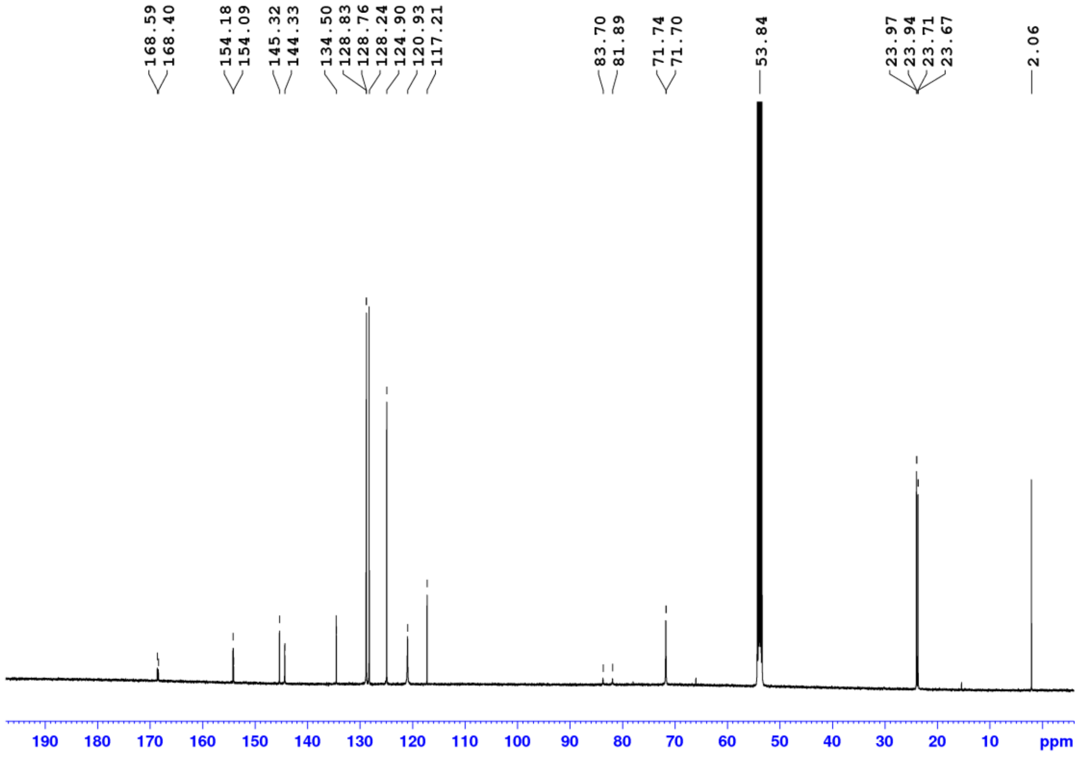

Figure S31. ^{13}C{^{1}H} NMR of the dissolved single crystals of [Li$_2$(**L**^{4})$_2$(CH$_3$CN)$_2$] (CD_2Cl_2, 300 K)

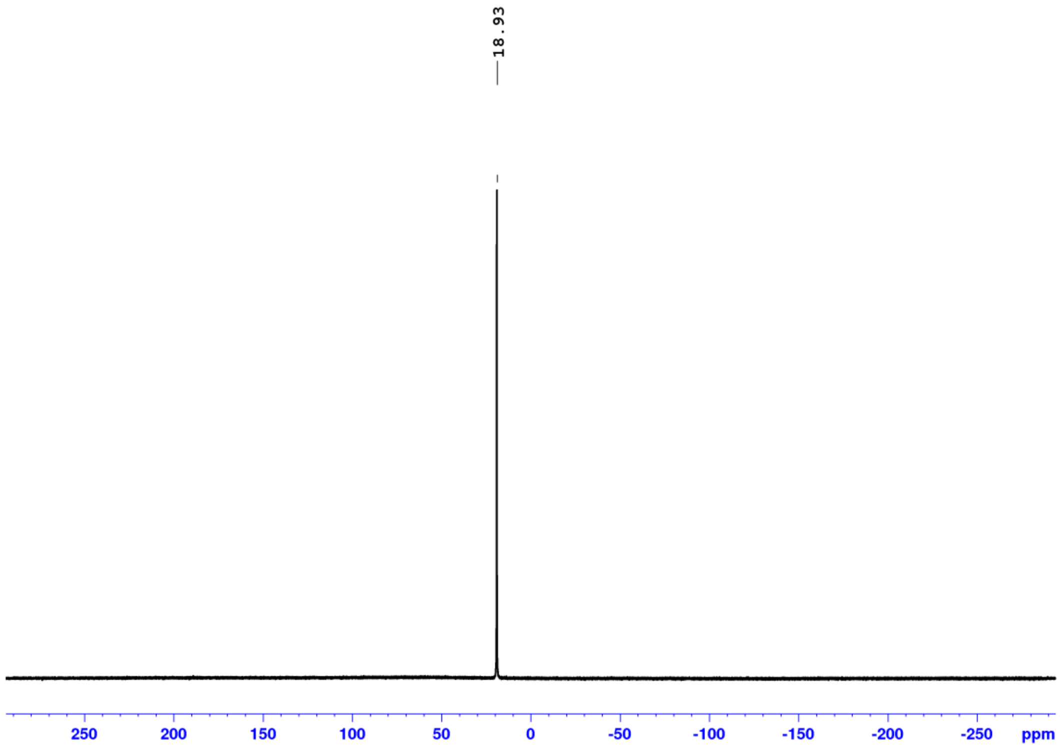

Figure S32. ^{31}P NMR of the dissolved single crystals of $[Li_2(\mathbf{L}^4)_2(CH_3CN)_2]$ (CD_2Cl_2, 300 K)

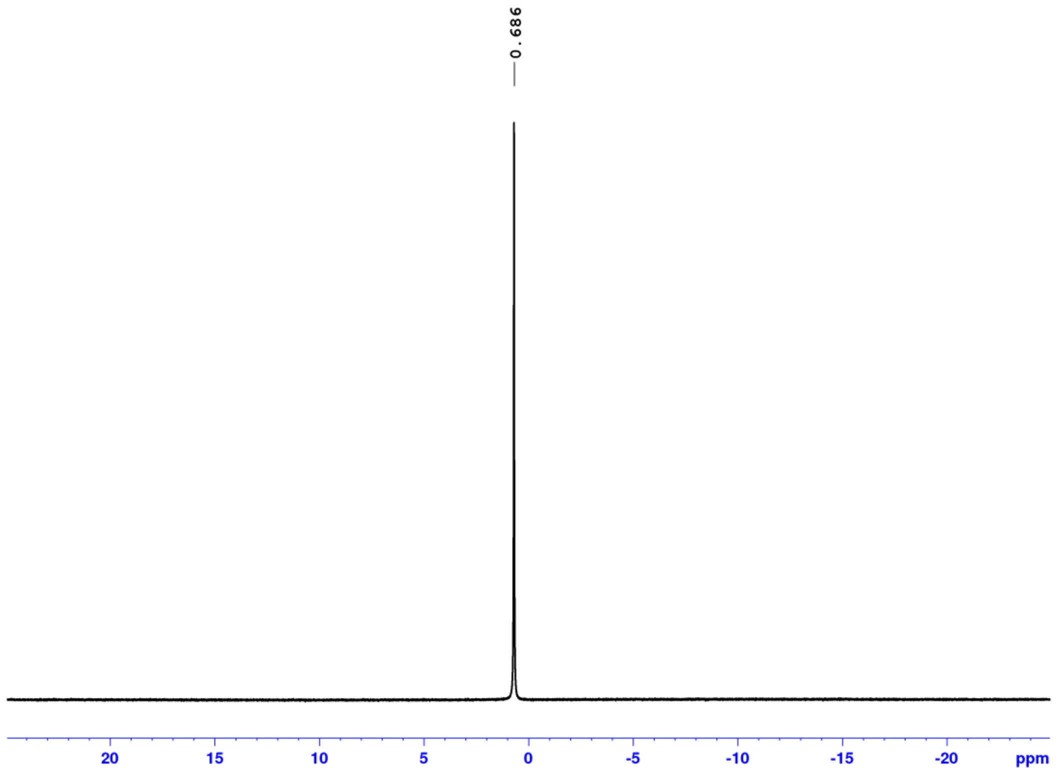

Figure S33. ^{7}Li NMR of the dissolved single crystals of $[Li_2(\mathbf{L}^4)_2(CH_3CN)_2]$ (CD_2Cl_2, 300 K)

S4.2. Preparation of [Li(L[1])(EtOH)$_2$] (7)

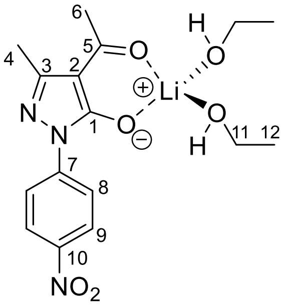

H**L**[1] (206.8 mg, 0.78 mmol, 1.0 eq.) was dissolved in 30 ml of EtOH and LiOH·H_2O (34.3 mg, 0.81 mmol, 1.0 eq.) were added. The reaction mixture was stirred at 75 °C for 4 h and then cooled to room temperature. The resulting red solution was stored at 4 °C for one week. The yellow precipitate was then collected by filtration. Suitable single crystals for X-ray diffraction analysis were obtained by recrystallization of resulting yellow precipitate from EtOH at 4 °C.

Yield: 82.3 mg (32%); **mp.**: 375 °C; **Raman** (100 mW, 298K, in cm^{-1}): 1591 (33), 1545 (9), 1319 (100), 1196 (5), 1176 (6), 1114 (18), 1076 (14); **IR** (ATR, 298 K, in cm^{-1}): 3560 (vw), 3505 (w), 3201 (w), 2975 (vw), 2924 (vw), 1639 (s), 1591 (s), 1497 (vs), 1467 (s), 1446 (m), 1399 (s), 1320 (vs), 1196 (w), 1178 (w), 1133 (vw), 1113 (m), 1097 (m), 1075 (s), 1053 (s), 1019 (m), 958 (m), 886 (vw), 854 (m), 829 (m), 763 (w), 750 (m), 688 (vw), 649 (w), 601 (m), 529 (w), 499 (m), 464 (m), 435 (w); **^{1}H NMR** (CD_3OD, 300K, in ppm): δ = 1.18 (6H, t, $^3J_{HH}$ = 7.1 Hz, H12), 2.37 (3H, s, H4), 2.43 (3H, s, H6), 3.61 (4H, q, $^3J_{HH}$ = 7.0 Hz, H11), 8.21-8.25 (4H, m, H8 and H9); **^{13}C{^{1}H} NMR** (CD_3OD, 300K, in ppm): δ = 16.9 (1C, s, C4), 18.4 (2C, s, C12), 28.0 (1C, s, C6), 58.3 (2C, s, C11), 105.3 (1C, s, C2), 119.7 (2C, s, C8), 125.4 (2C, s, C9), 144.1 (1C, s, C7), 146.7 (1C, s, C10), 154.3 (1C, s, C3), 168.4 (1C, s, C1), 195.0 (1C, s, C5); **^{7}Li NMR** (CD_3OD, 300K, in ppm): δ = -0.15 (s); **Elemental analysis**: Complex **7** is very hygroscopic and readily lose the solvated EtOH, and the results are consistent with Li(**L**[1])(1.5EtOH)(0.5H_2O): calculated: C 52.18, H 5.84, N 12.17; found: C 51.89, H 5.58, N 11.75. **ESI-MS** (m/z, [Da/e]): 260.1 [M-Li-2EtOH]$^-$, 527.0 [M-2EtOH+**L**]$^-$, 794.2 [M-2EtOH+2**L**+Li]$^-$.

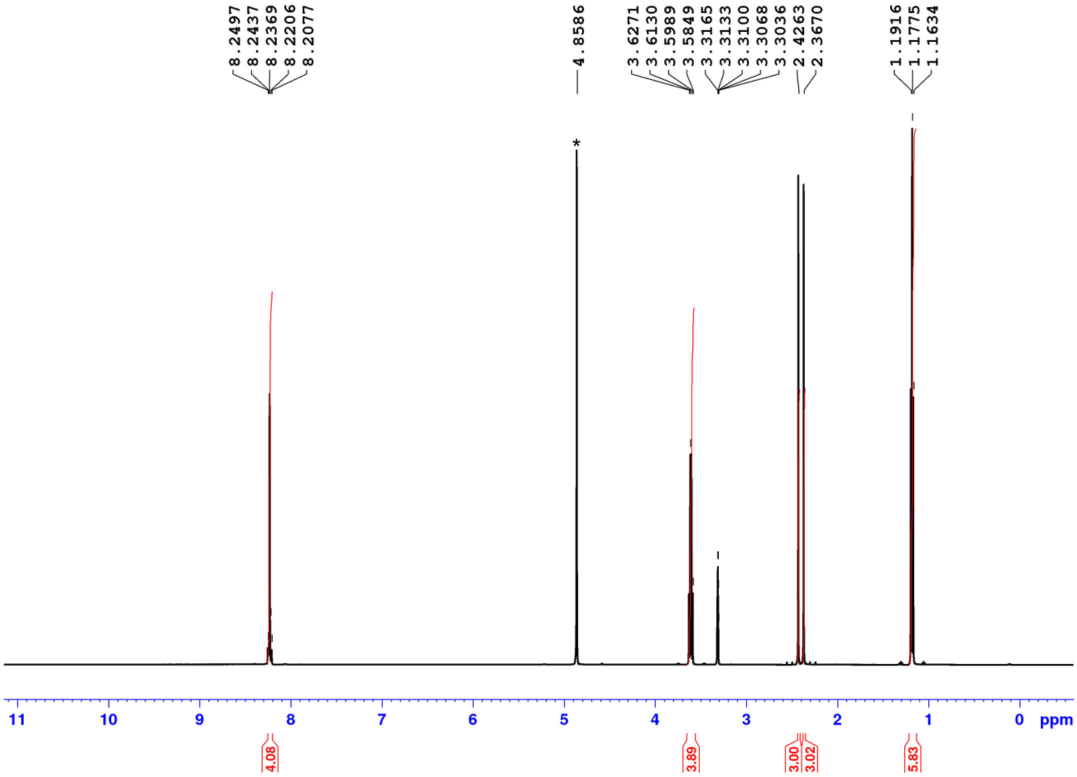

Figure S34. ^{1}H NMR of [Li(**L**[1])(EtOH)$_2$] (CD_3OD, 300 K, * H_2O)

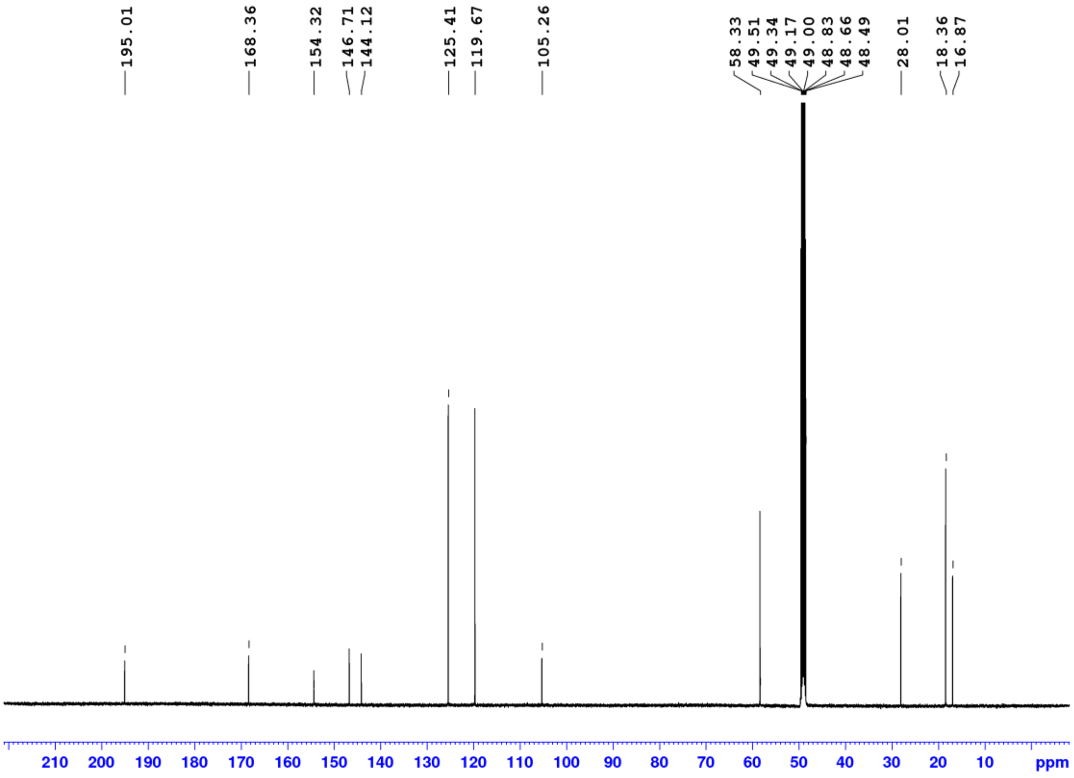

Figure S35. $^{13}C\{^{1}H\}$ NMR of [Li(**L**^{1})(EtOH)$_2$] (CD_3OD, 300 K)

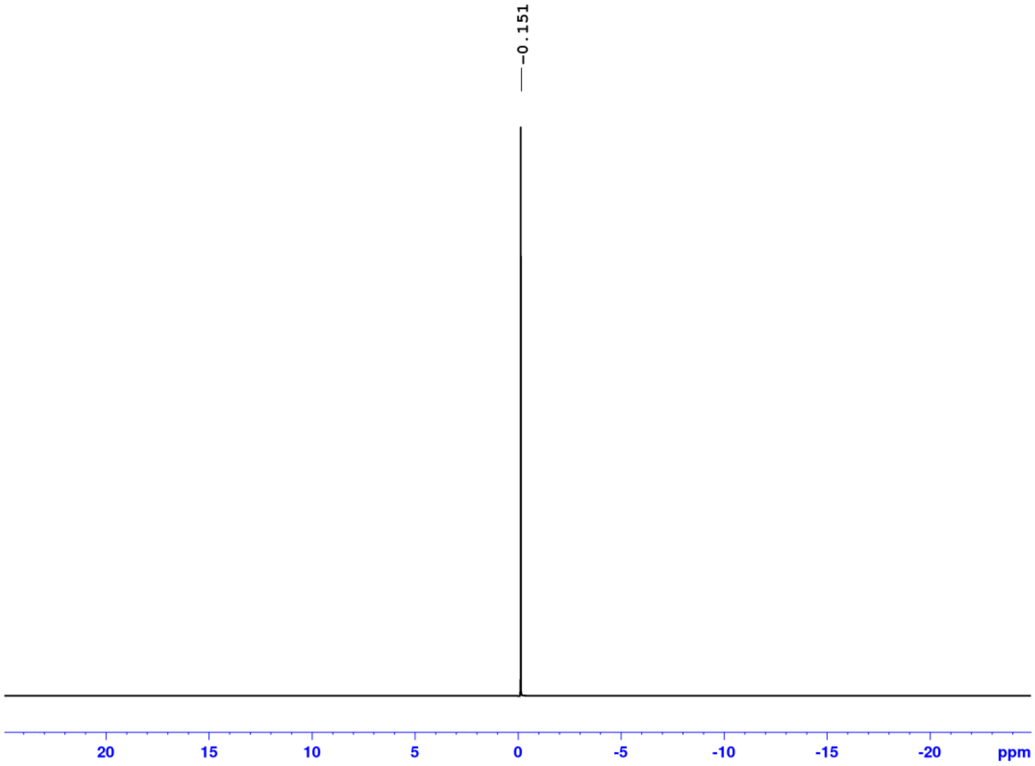

Figure S36. ^{7}Li NMR of [Li(**L**^{1})(EtOH)$_2$] (CD_3OD, 300 K)

S4.3. General procedure for the synthesis of TBPO-coordinated lithium complexes

To prepare the $[Li_2(\mathbf{L}^n)_2(TBPO)_2]$ complexes, H**L**^{n} and tributylphosphine oxide (TBPO, 1.0 eq) were dissolved in CH_2Cl_2 and $LiOH{\cdot}H_2O$ (1.0 eq.) was added. The reaction mixture was stirred at 50 °C for 3 - 4 h and the volume was then reduced to approximately 1 ml. The resulting solution was filtered and 40 - 60 ml pentane were added with stirring. The yellow solution obtained was stored at -18 °C for one week. The yellow precipitate that formed was collected by filtration, washed with pentane, and dried *in vacuo*. Suitable single crystals for X-ray diffraction analysis were obtained by diffusion of pentane into a solution of the complex in CH_2Cl_2 at -18 °C.

S4.3.1. Preparation of $[Li_2(\mathbf{L}^1)_2(TBPO)_2]$ (8)

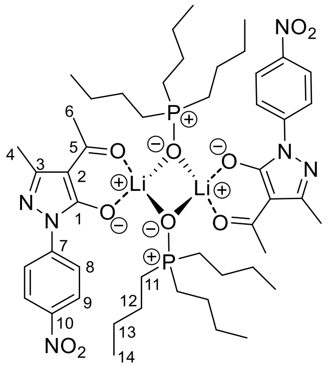

$[Li_2(\mathbf{L}^1)_2(TBPO)_2]$ (**8**) was prepared according to the general procedure. H**L**^{1} (187.8 mg, 0.70 mmol, 1.0 eq.) and tributylphosphine oxide (TBPO) (171.0 mg, 0.74 mmol, 1.0 eq.) were dissolved in 10 ml of CH_2Cl_2 and $LiOH{\cdot}H_2O$ (31.5 mg, 0.74 mmol, 1.0 eq.) were added. Recrystallization of the crude material yielded yellow crystals suitable for X-ray analysis.

Yield: 277.5 mg (82%); **mp.**: 253 °C; **Raman** (100 mW, 298 K, in cm^{-1}): 2930 (6), 2907 (6), 1591 (40), 1548 (12), 1321 (100), 1301 (16), 1194 (6), 1172 (9), 1114 (22), 1074 (13), 1007 (6), 861 (5); **IR** (ATR, 298 K, in cm^{-1}): 2956 (w), 2928 (w), 2871 (vw), 1640 (s), 1589 (s), 1513 (s), 1498 (vs), 1458 (s), 1424 (m), 1397 (m), 1316 (vs), 1223 (w), 1191 (w), 1172 (w), 1132 (s), 1111 (m), 1097 (m), 1072 (vs), 1006 (w), 958 (m), 901 (w), 860 (s), 823 (s), 801 (m), 763 (w), 753 (m), 708 (w), 692 (w), 646 (w), 599 (m), 526 (w), 503 (s), 479 (s), 457 (m), 421 (w); **^{1}H NMR** (CD_2Cl_2, 300K, in ppm): δ = 0.80 (18H, t, $^3J_{HH}$ = 7.2 Hz, H14), 1.26 (12H, sext, $^3J_{HH}$ = 7.2 Hz, H13), 1.38-1.47 (12H, m, H12), 1.60-1.67 (12H, m, H11), 2.33 (6H, s, H6), 2.39 (6H, s, H4), 8.17 (4H, d, $^3J_{HH}$ = 9.1 Hz, H8), 8.36 (4H, d, $^3J_{HH}$ = 9.0 Hz, H9); **^{13}C{^{1}H} NMR** (CD_2Cl_2, 300K, in ppm): δ = 13.7 (6C, s, C14), 17.9 (2C, s, C4), 23.7 (6C, d, $^2J_{CP}$ = 4 Hz, C12), 24.6 (6C, d, $^3J_{CP}$ = 15 Hz, C13), 27.1 (6C, d, $^1J_{CP}$ = 66 Hz, C11), 28.6 (2C, s, C6), 105.4 (2C, s, C2), 118.1 (4C, s, C9), 124.8 (4C, s, C8), 143.0 (2C, s, C7), 146.0 (2C, s, C10), 150.7 (2C, s, C3), 167.2 (2C, s, C1), 192.0 (2C, s, C5); **^{31}P{^{1}H} NMR** (CD_2Cl_2, 300K, in ppm): δ = 59.0 (s); **^{31}P NMR** (CD_2Cl_2, 300K, in ppm): δ = 59.0 (s); **^{7}Li NMR** (CD_2Cl_2, 300K, in ppm): δ = 1.12 (s); **Elemental analysis** for $C_{48}H_{74}Li_2N_6O_{10}P_2$ $[Li_2(\mathbf{L}^1)_2(TBPO)_2]$, calculated: C 59.38, H 7.68, N 8.66; found: C 59.04, H 7.43, N 8.50; **ESI-MS** (m/z, [Da/e]): 260.1 [M-2Li-**L**-2TBPO]$^-$, 527.1 [M-Li-2TBPO]$^-$, 794.1 [M-2TBPO+**L**]$^-$, 1061.3 [M-2TBPO+Li+2**L**]$^-$ (ESI$^-$); 443.5 [M-Li-2**L**]$^-$ (ESI$^+$).

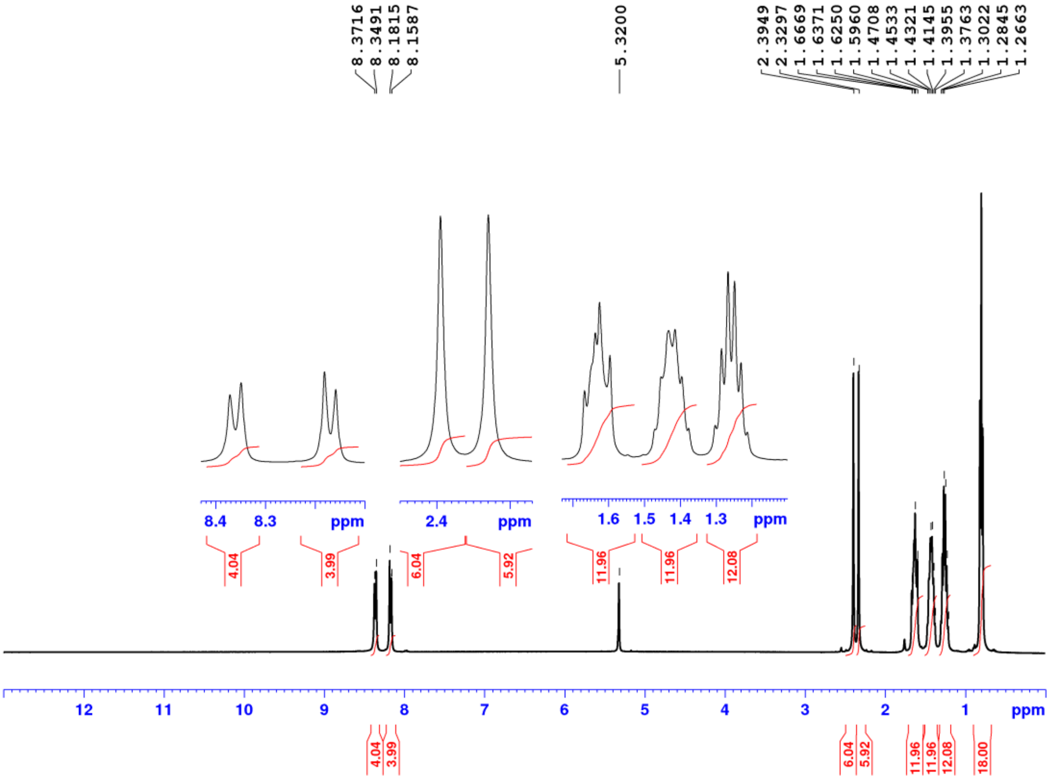

Figure S37. ^{1}H NMR of $[Li_2(\mathbf{L}^1)_2(TBPO)_2]$ (CD_2Cl_2, 300 K)

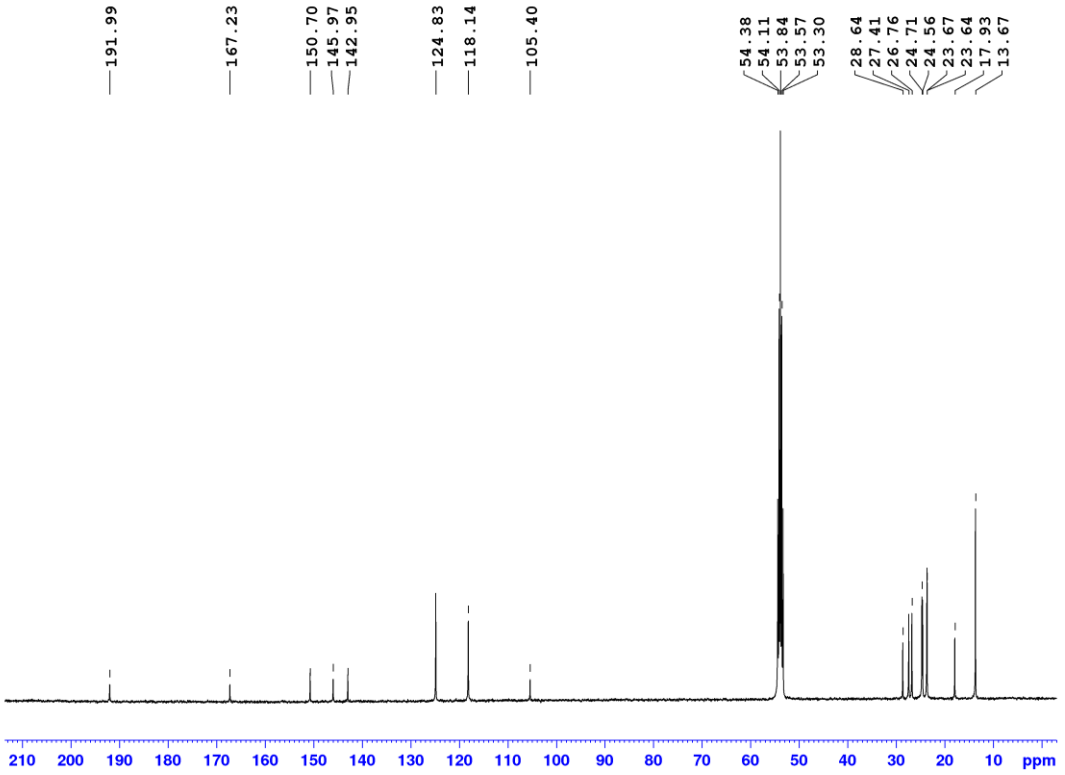

Figure S38. $^{13}C\{^{1}H\}$ NMR of $[Li_2(\mathbf{L}^1)_2(TBPO)_2]$ (CD_2Cl_2, 300 K)

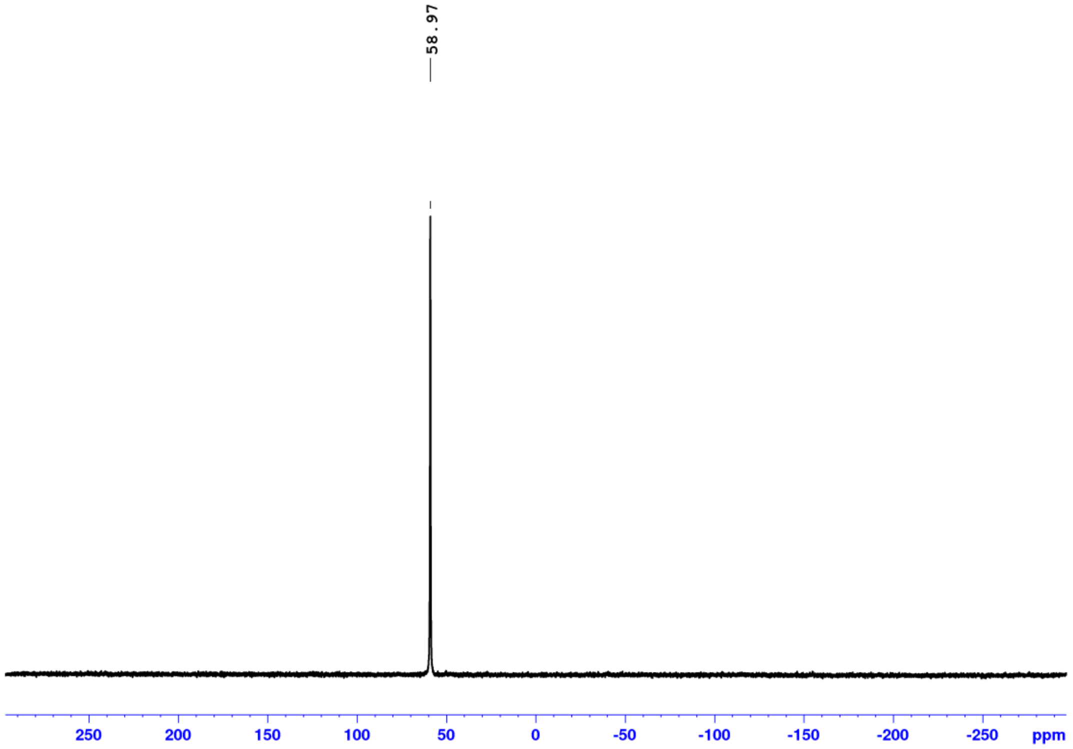

Figure S39. ^{31}P NMR of $[Li_2(\mathbf{L}^1)_2(TBPO)_2]$ (CD_2Cl_2, 300 K)

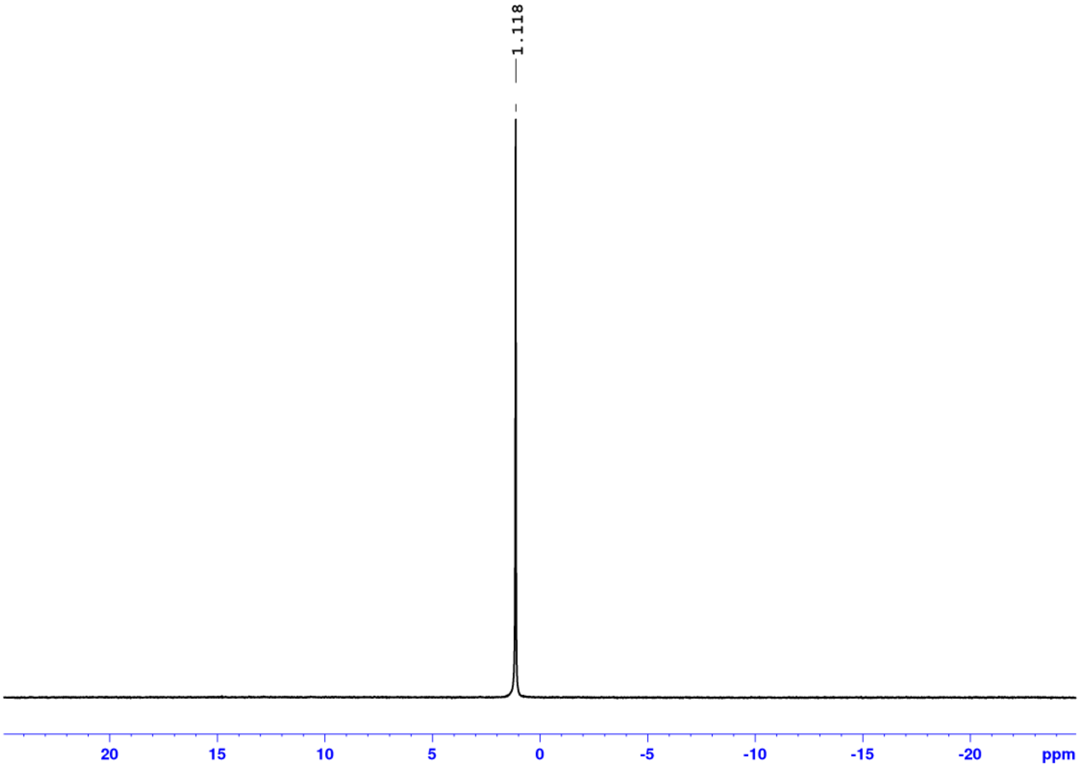

Figure S40. ^{7}Li NMR of $[Li_2(\mathbf{L}^1)_2(TBPO)_2]$ (CD_2Cl_2, 300 K)

S4.3.2. Preparation of [$Li_2(L^3)_2(TBPO)_2$] (9)

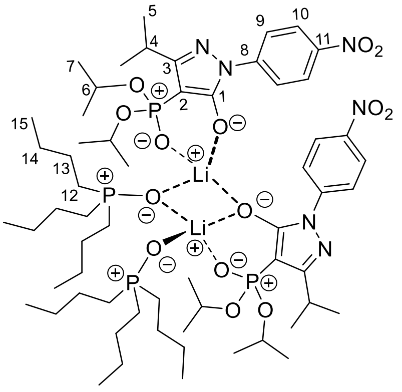

HL[3] (133.6 mg, 0.32 mmol, 1.0 eq.) and of tributylphosphine oxide (TBPO) (75.6 mg, 0.33 mmol, 1.0 eq.) were dissolved in 15 ml of CH_2Cl_2 and $LiOH{\cdot}H_2O$ (17.5 mg, 0.41 mmol, 1.3 eq.) was added. Recrystallization of the crude material that formed yielded yellow crystals suitable for X-ray analysis.

Yield: 98.5 mg (48%); **mp.**: 116 °C; **Raman** (100 mW, 298 K, in cm^{-1}): 2917 (6), 1608 (6), 1597 (10), 1584 (23), 1514 (6), 1317 (100), 1268 (11), 1170 (6), 1137 (8), 1111 (25), 996 (5), 880 (5); **IR** (ATR, 298 K, in cm^{-1}): 2960 (w), 2929 (w), 2870 (vw), 1606 (s), 1580 (m), 1499 (m), 1465 (w), 1426 (w), 1383 (w), 1353 (w), 1314 (vs), 1264 (m), 1199 (m), 1178 (w), 1166 (w), 1124 (m), 1106 (s), 1085 (m), 1033 (vw), 974 (vs), 893 (w), 879 (w), 855 (s), 826 (w), 808 (vw), 776 (m), 754 (s), 694 (w), 670 (m), 588 (s), 538 (w), 488 (w), 453 (w); **^{1}H NMR** (CD_2Cl_2, 300K, in ppm): δ = 0.83 (18H, t, $^3J_{HH}$ = 7.2 Hz, H15), 1.19 (12H, d, $^3J_{HH}$ = 5.8 Hz, H7a), 1.25 (12H, d, $^3J_{HH}$ = 6.9 Hz, H5), 1.27-1.32 (24H, m, H7b and H14), 1.41 (12H, s (br), H13), 1.57-1.63 (12H, m, H12), 2.93 (2H, sept, $^3J_{HH}$ = 6.9 Hz, H4), 4.55 (4H, s (br), H6), 8.09 (4H, d, $^3J_{HH}$ = 6.6 Hz, H9), 8.33(4H, s (br), H10); **^{13}C{^{1}H} NMR** (CD_2Cl_2, 300K, in ppm): δ = 13.7 (6C, s, C15), 22.0 (4C, s, C5), 24.0 (6C, d, $^2J_{CP}$ = 4 Hz, C13), 24.2 (6C, d, $^3J_{CP}$ = 1 Hz, C7a), 24.2 (4C, d, $^3J_{CP}$ = 2 Hz, C7b), 24.6 (4C, d, $^3J_{CP}$ = 15 Hz, C14), 27.3 (6C, d, $^1J_{CP}$ = 66 Hz, C12), 28.4 (2C, s, C4), 70.3 (4C, d, $^2J_{CP}$ = 4 Hz, C6), 80.9 (2C, d, $^1J_{CP}$ = 229 Hz, C2), 118.7 (4C, s, C10), 124.8 (4C, s, C9), 142.8 (2C, s, C8), 146.7 (2C, s, C11), 161.3 (2C, d, $^2J_{CP}$ = 13 Hz, C3), 169.3 (2C, d, $^1J_{CP}$ = 22 Hz, C1); **^{31}P{^{1}H} NMR** (CD_2Cl_2, 300K, in ppm): δ = 20.1 (s), 52.4 (s (br)); **^{31}P NMR** (CD_2Cl_2, 300K, in ppm): δ = 20.1 (s), 52.4 (s (br)); **^{7}Li NMR** (CD_2Cl_2, 300K, in ppm): δ = 0.63 (s); **Elemental analysis** for $C_{60}H_{104}Li_2N_6O_{14}P_4$ [Li_2(**L**^{3})$_2$(TBPO)$_2$], calculated: C 56.69, H 8.25, N 6.61; found: C 56.60, H 8.10, N 6.52; **ESI-MS** (m/z, [Da/e]): 410.0 [M-2Li-**L**-2TBPO]$^-$, 827.3 [M-Li-2TBPO]$^-$, 1244.6 [M-2TBPO+**L**]$^-$, 1662.0 [M-2TBPO+Li+2**L**]$^-$ (ESI$^-$); 443.5 [M-Li-2**L**]$^-$ (ESI$^+$).

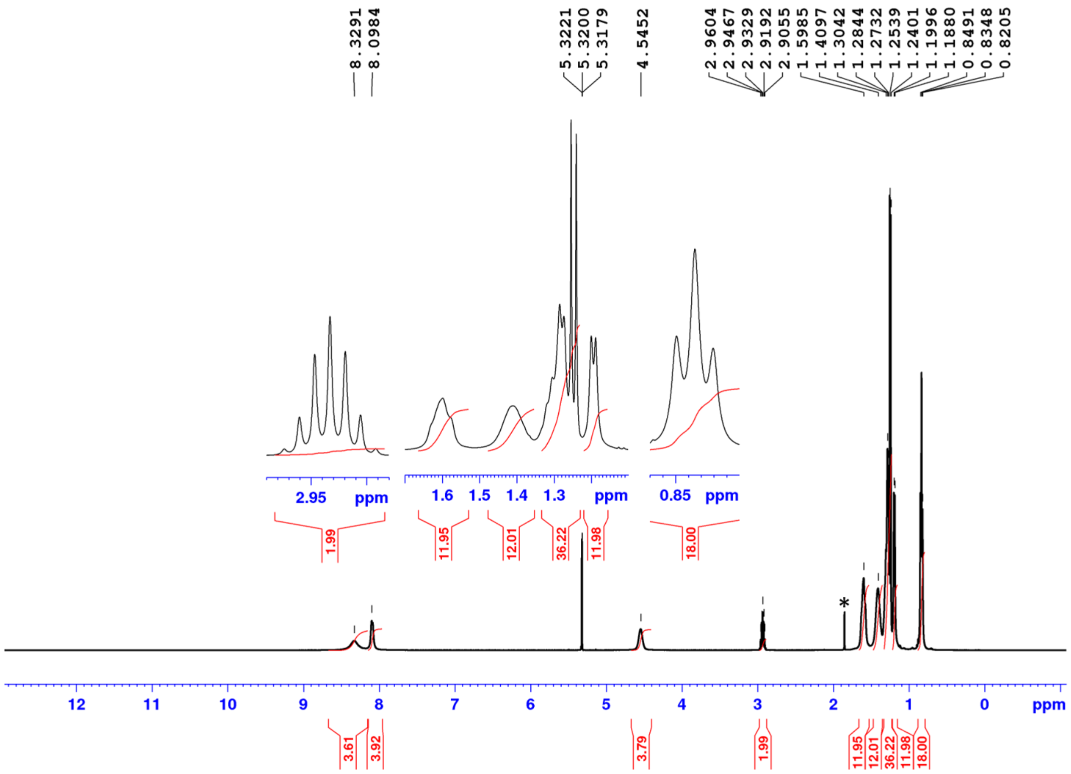

Figure S41. ^{1}H NMR of [Li_2(**L**^{3})$_2$(TBPO)$_2$] (CD_2Cl_2, 300 K, * H_2O)

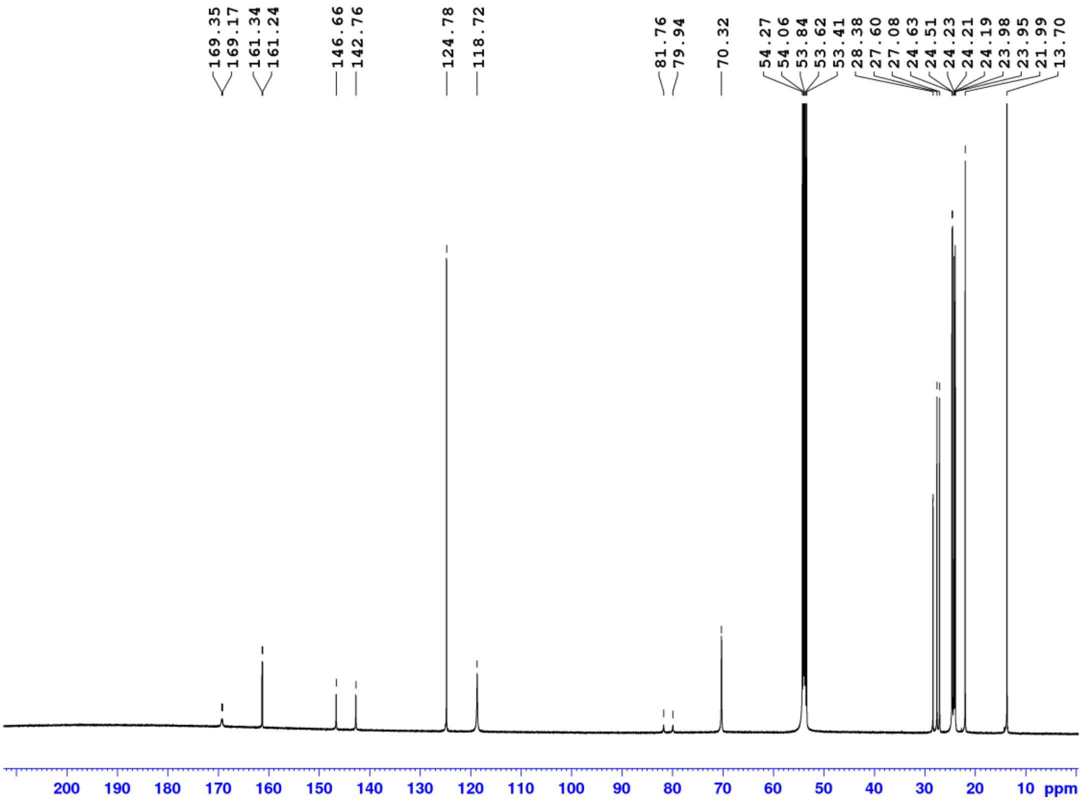

Figure S42. $^{13}C\{^{1}H\}$ NMR of $[Li_2(\mathbf{L}^3)_2(TBPO)_2]$ (CD_2Cl_2, 300 K)

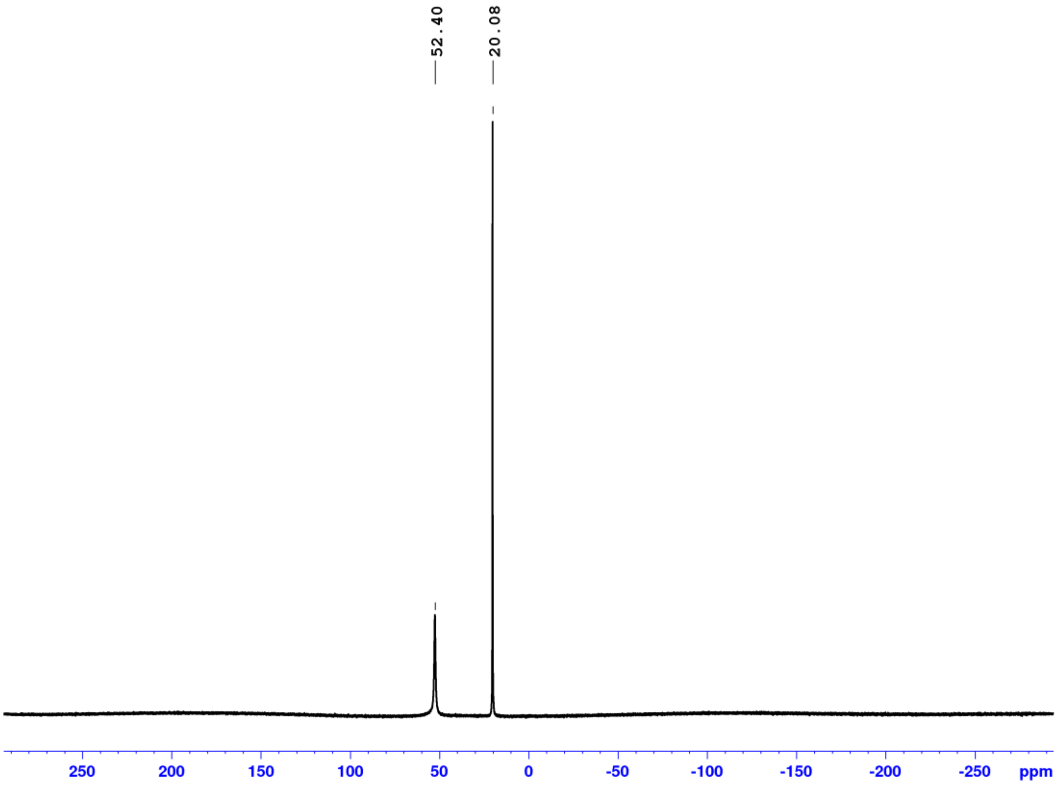

Figure S43. ^{31}P NMR of $[Li_2(\mathbf{L}^3)_2(TBPO)_2]$ (CD_2Cl_2, 300 K)

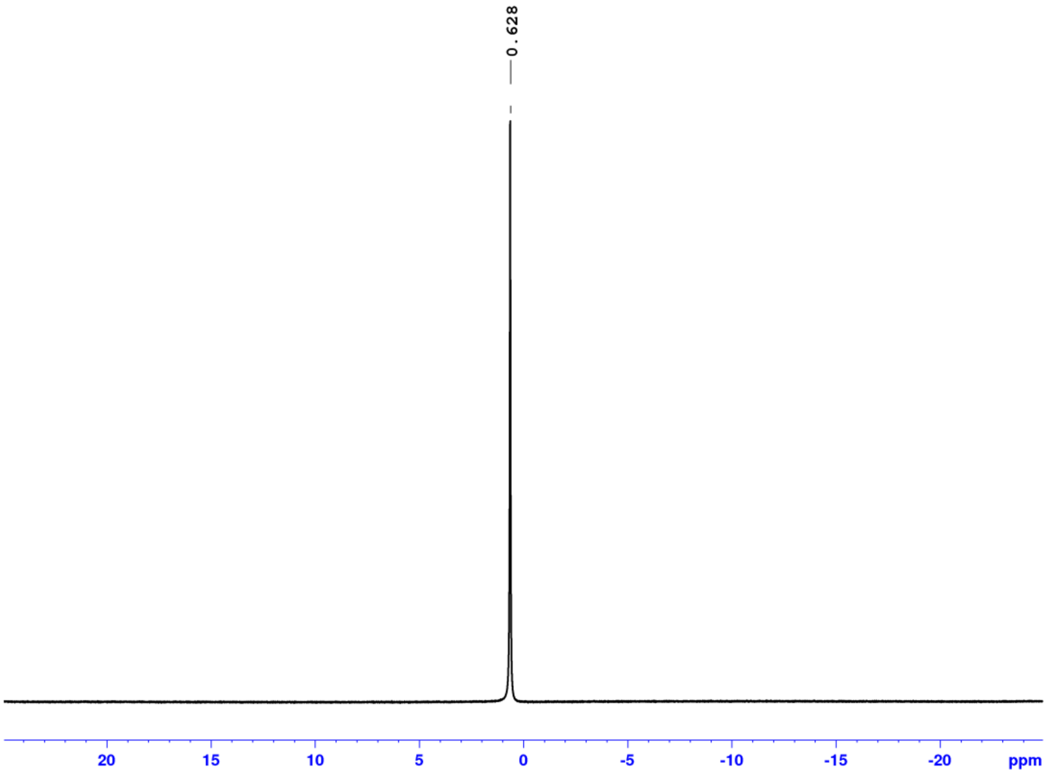

Figure S44. ^{7}Li NMR of [Li_2(**L**^{3})$_2$(TBPO)$_2$] (CD_2Cl_2, 300 K)

S4.3.3 Preparation of [Li_2(L^4)$_2$(TBPO)$_2$]·2CH_2Cl_2 (10)

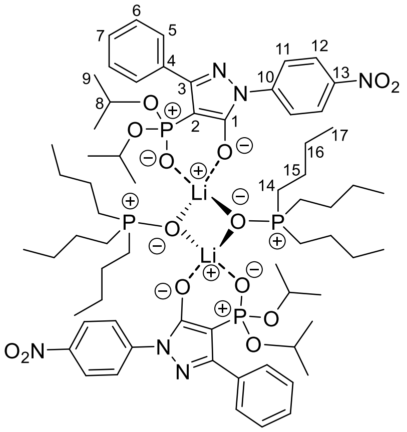

HL[4] (151.5 mg, 0.33 mmol, 1.0 eq.) and tributylphosphine oxide (TBPO) (78.0 mg, 0.34 mmol, 1.0 eq.) were dissolved in 15 ml of CH_2Cl_2 and LiOH·H_2O (15.0 mg, 0.35 mmol, 1.0 eq.) was added. Recrystallization of the crude material that formed yielded orange crystals suitable for X-ray analysis.

Yield: 160.5 mg (72%); **mp.**: 131 °C; **Raman** (100 mW, 298 K, in cm^{-1}): 2938 (7), 2906 (7), 2874 (5), 1606 (20), 1588 (32), 1513 (10), 1479 (10), 1442 (14), 1325 (100), 1284 (8), 1162 (10), 1137 (5), 1112 (28), 970 (13); **IR** (ATR, 298 K, in cm^{-1}): 2958 (w), 2931 (w), 2872 (vw), 2647 (vw), 1605 (s), 1571 (m), 1511 (m), 1495 (m), 1475 (w), 1407 (w), 1383 (w), 1321 (vs), 1280 (m), 1235 (vw), 1205 (m), 1161 (w), 1129 (w), 1108 (s), 1075 (vw), 1004 (m), 980 (vs), 967 (vs), 894 (w), 882 (w), 855 (m), 822 (w), 771 (m), 752 (s), 698 (m), 671 (w), 614 (vs), 552 (w), 533 (w), 509 (w), 491 (w), 446 (w); **^{1}H NMR** (CD_2Cl_2, 300K, in ppm): δ = 0.83 (18H, t, $^3J_{HH}$ = 7.3 Hz, H17), 1.02 (12H, d, $^3J_{HH}$ = 5.5 Hz, H9a), 1.21 (12H, d, $^3J_{HH}$ = 5.8 Hz, H9b), 1.29 (12H, sext, $^3J_{HH}$ = 7.3 Hz, H16), 1.38-1.46 (12H, m, H15), 1.59-1.65 (12H, m, H14), 4.52 (4H, s (br), H8), 7.32-7.38 (6H, m, H7 and H6), 7.85 (4H, d, $^3J_{HH}$ = 6.7 Hz, H5), 8.17 (4H, d, $^3J_{HH}$ = 7.8 Hz, H11), 8.52 (4H, s (br), H12); **^{13}C{^{1}H} NMR** (CD_2Cl_2, 300K, in ppm): δ = 13.7 (6C, s, C17), 23.9 (4C, d, $^3J_{CP}$ = 5 Hz, C9a), 24.0 (6C, d, $^2J_{CP}$ = 4 Hz, C15), 24.1 (4C, d, $^3J_{CP}$ = 4 Hz, C9b), 24.6 (6C, d, $^3J_{CP}$ = 15 Hz, C16), 27.3 (6C, d, $^1J_{CP}$ = 66 Hz, C14), 70.5 (4C, s, C8), 81.2 (2C, d, $^1J_{CP}$ = 225 Hz, C2), 119.0 (4C, s (br), C12), 124.8 (4C, s, C11), 128.0 (4C, s, C6), 128.4 (2C, s, C7), 128.8 (4C, s, C5), 135.3 (2C, s, C4), 143.2 (2C, s, C10), 146.4 (2C, s, C13), 154.2 (2C, d, $^2J_{CP}$ = 12 Hz, C3), 170.2 (2C, s (br), C1); **^{31}P{^{1}H} NMR** (CD_2Cl_2, 300K, in ppm): δ = 19.3 (s), 53.1(s (br)); **^{31}P NMR** (CD_2Cl_2, 300K, in ppm): δ = 19.3 (s), 53.1 (s (br)); **^{7}Li NMR** (CD_2Cl_2, 300K, in ppm): δ = 0.69 (s (br)); **Elemental analysis** for $C_{66}H_{100}Li_2N_6O_{14}P_4$ [Li_2(**L**^{4})$_2$(TBPO)$_2$], calculated: C 59.19, H 7.53, N 6.27; found: C 59.22, H 7.24, N 6.26; **ESI-MS** (m/z, [Da/e]): 444.2 [M-2Li-**L**-2TBPO]$^-$, 895.3 [M-Li-2TBPO]$^-$, 1346.6 [M-2TBPO+**L**]$^-$ (ESI$^-$); 443.5 [M-Li-2**L**]$^-$ (ESI$^+$).

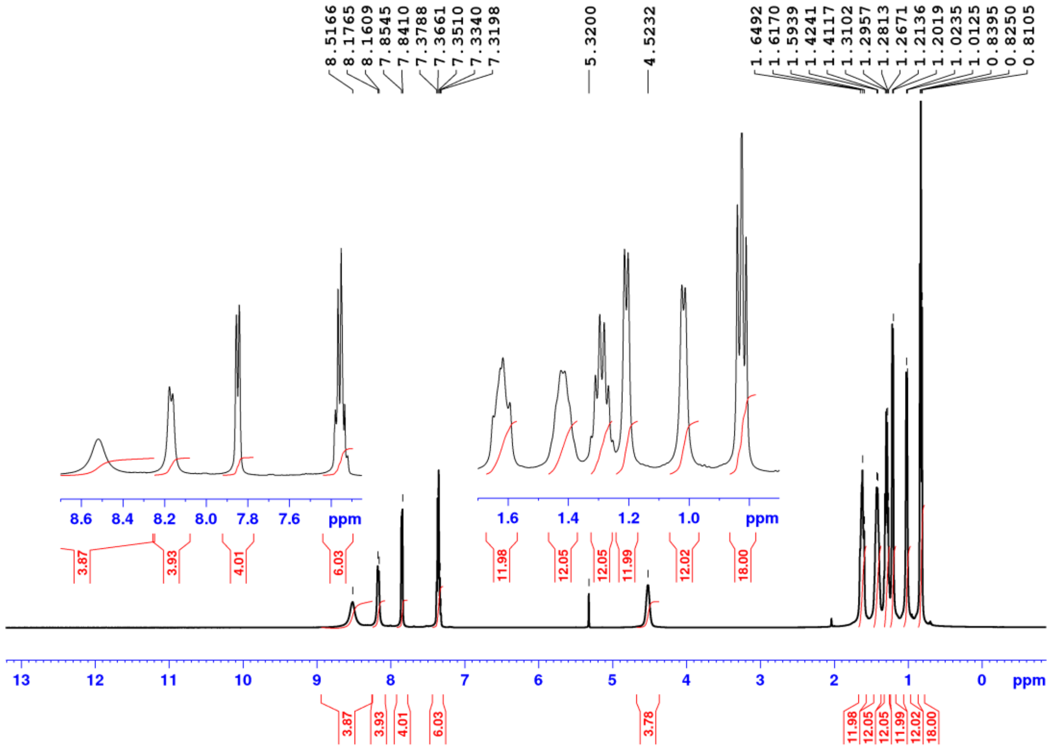

Figure S45. ^{1}H NMR of [Li_2(**L**^{4})$_2$(TBPO)$_2$] (CD_2Cl_2, 300 K)

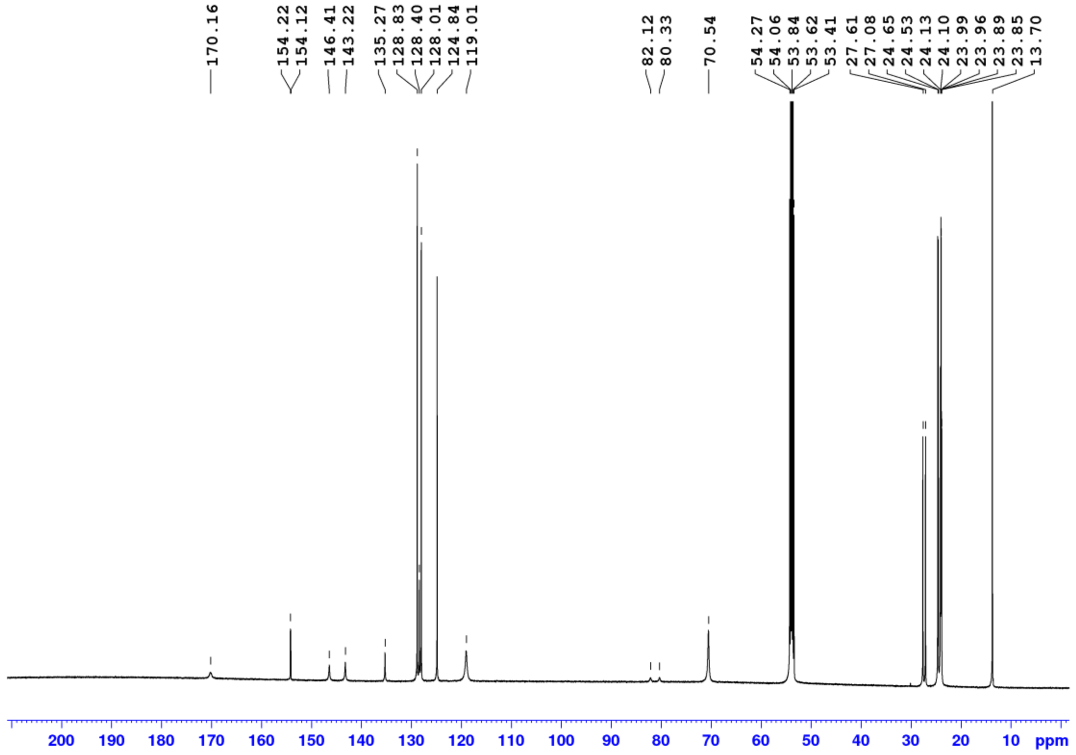

Figure S46. $^{13}C\{^{1}H\}$ NMR of [Li_2(**L**^{4})$_2$(TBPO)$_2$] (CD_2Cl_2, 300 K)

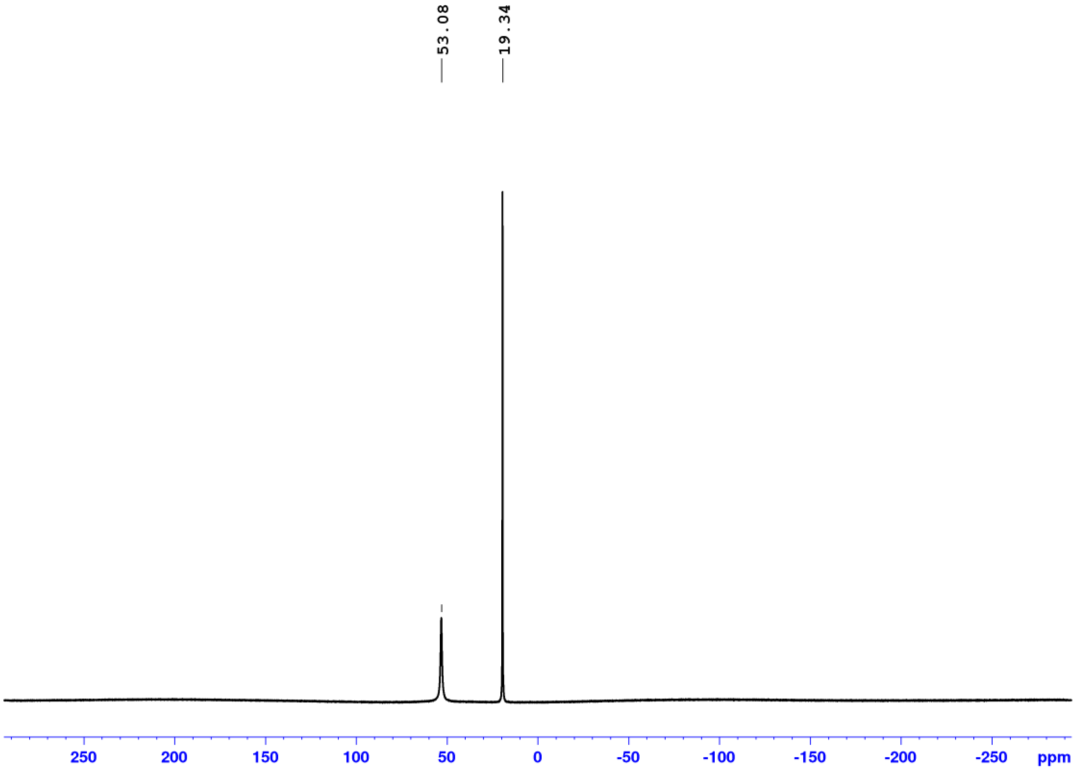

Figure S47. ^{31}P NMR of [Li_2(**L**^{4})$_2$(TBPO)$_2$] (CD_2Cl_2, 300 K)

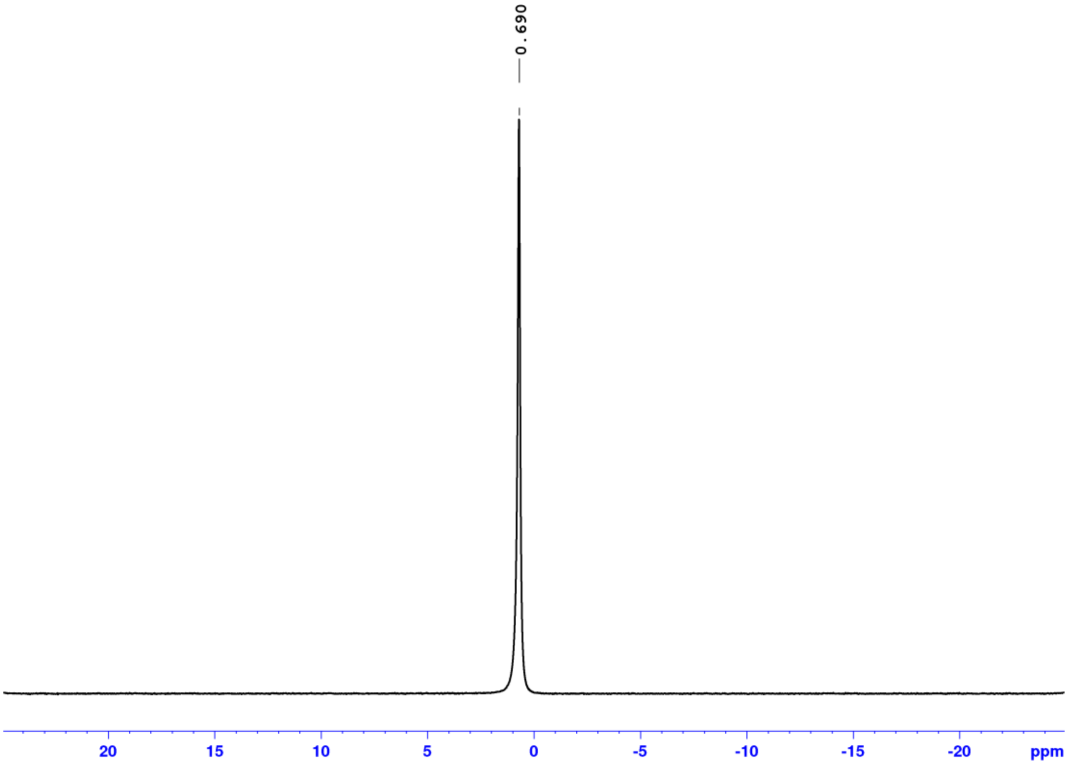

Figure S48. ^{7}Li NMR of [Li_2(**L**^{4})$_2$(TBPO)$_2$] (CD_2Cl_2, 300 K)

S4.4. Preparation of [$Li_2(L^4)_2(TBP)_2$] (11)

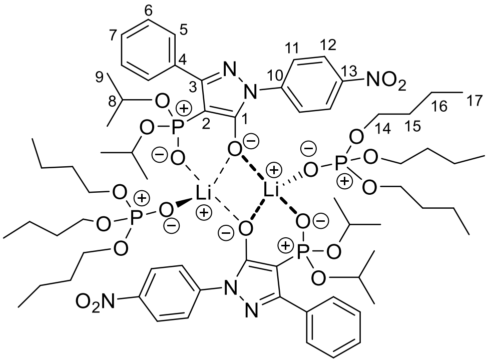

HL[4] (51.7 mg, 0.11 mmol, 1.0 eq.), tributyl phosphate (TBP) (33.0 mg, 0.12 mmol, 1.0 eq.) and $LiOH{\cdot}H_2O$ (5.7 mg, 0.13 mmol, 1.2 eq.) were added to 0.8 ml of CH_2Cl_2. The reaction mixture was stirred at room temperature for 2 h. The resulting yellow solution was filtered and single crystals suitable for X-ray diffraction analysis were obtained by diffusion of pentane into a solution of the dissolved complex in CH_2Cl_2 at -18 °C.

Yield: 42.1 mg (53%); **mp.**: 87 °C; **Raman** (100 mW, 298 K, in cm^{-1}): 2917 (5), 1606 (18), 1585 (19), 1513 (9), 1477 (7), 1441 (11), 1325 (100), 1287 (8), 1166 (8), 1112 (20), 972 (10); **IR** (ATR, 298 K, in cm^{-1}): 2961 (w), 2933 (vw), 2873 (vw), 1598 (m), 1562 (m), 1511 (m), 1495 (w), 1474 (w), 1442 (vw), 1416 (w), 1385 (vw), 1373 (vw), 1325 (vs), 1282 (m), 1207 (m), 1179 (vw), 1164 (vw), 1139 (vw), 1110 (w), 1057 (w), 1013 (s), 999 (s), 971 (vs), 916 (w), 899 (w), 886 (w), 855 (m), 812 (w), 763 (m), 749 (m), 729 (w), 690 (m), 684 (w), 670 (w), 630 (w), 617 (s), 553 (w), 536 (w), 490 (vw), 458 (w); **^{1}H NMR** (CD_2Cl_2, 300K, in ppm): δ = 0.85 (18H and ~ 0.2 eq. C_5H_{12} according to elemental analysis, t, $^3J_{HH}$ = 7.4 Hz, H17), 0.99 (12H, d, $^3J_{HH}$ = 5.5 Hz, H9a), 1.20 (12H, d, $^3J_{HH}$ = 5.3 Hz, H9b), 1.29 (12H and ~ 0.2 eq. C_5H_{12} according to elemental analysis, sext, $^3J_{HH}$ = 7.4 Hz, H16), 1.52 (12H, quint, $^3J_{HH}$ = 6.9 Hz, H15), 3.87 (12H, q, $^3J_{HH}$ = 6.4 Hz, H14), 4.53 (4H, d, $^3J_{HP}$ = 5.5 Hz, H8), 7.34-7.40 (6H, m, H6 and H7), 7.86 (4H, d, $^3J_{HH}$ = 6.3 Hz, H5), 8.16 (4H, s(br), H11), 8.34 (4H, s(br), H12); **^{13}C{^{1}H} NMR** (CD_2Cl_2, 300K, in ppm): δ = 13.7 (6C, s, C17), 19.0 (6C, s, C16), 23.8 (4C, d, $^3J_{CP}$ = 5 Hz, C9a), 24.0 (4C, d, $^3J_{CP}$ = 4 Hz, C9b), 32.6 (6C, d, $^3J_{CP}$ = 7 Hz, C15), 68.0 (6C, d, $^2J_{CP}$ = 6 Hz, C14), 71.2 (4C, d, $^2J_{CP}$ = 5 Hz, C8), 82.2 (2C, d, $^1J_{CP}$ = 227 Hz, C2), 120.0 (4C, s(br), C12), 124.9 (4C, s, C11), 128.1 (4C, s, C6), 128.7 (2C, s, C7), 128.8 (4C, s, C5), 134.8 (2C, s, C4), 143.9 (2C, s, C10), 145.7 (2C, s, C13), 154.1 (2C, d, $^2J_{CP}$ = 12 Hz, C3), 169.2 (2C, d, $^2J_{CP}$ = 22 Hz, C1); **^{31}P{^{1}H} NMR** (CD_2Cl_2, 300K, in ppm): δ = -1.9 (s), 19.2 (s); **^{31}P NMR** (CD_2Cl_2, 300K, in ppm): δ = -1.9 (s), 19.2 (s); **^{7}Li NMR** (CD_2Cl_2, 300K, in ppm): δ = 0.80 (s); **Elemental analysis** for $C_{67}H_{102.4}Li_2N_6O_{20}P_4$ [$Li_2(\mathbf{L}^4)_2(TBP)_2$]$\cdot 0.2C_5H_{12}$, calculated: C 55.51, H 7.12, N 5.80; found: C 55.83, H 7.14, N 5.66; **ESI-MS** (m/z, [Da/e]): 444.0 [M-2Li-**L**-2TBP]$^-$, 895.3 [M-Li-2TBP]$^-$, 1346.6 [M-2TBP+**L**]$^-$ (ESI$^-$).

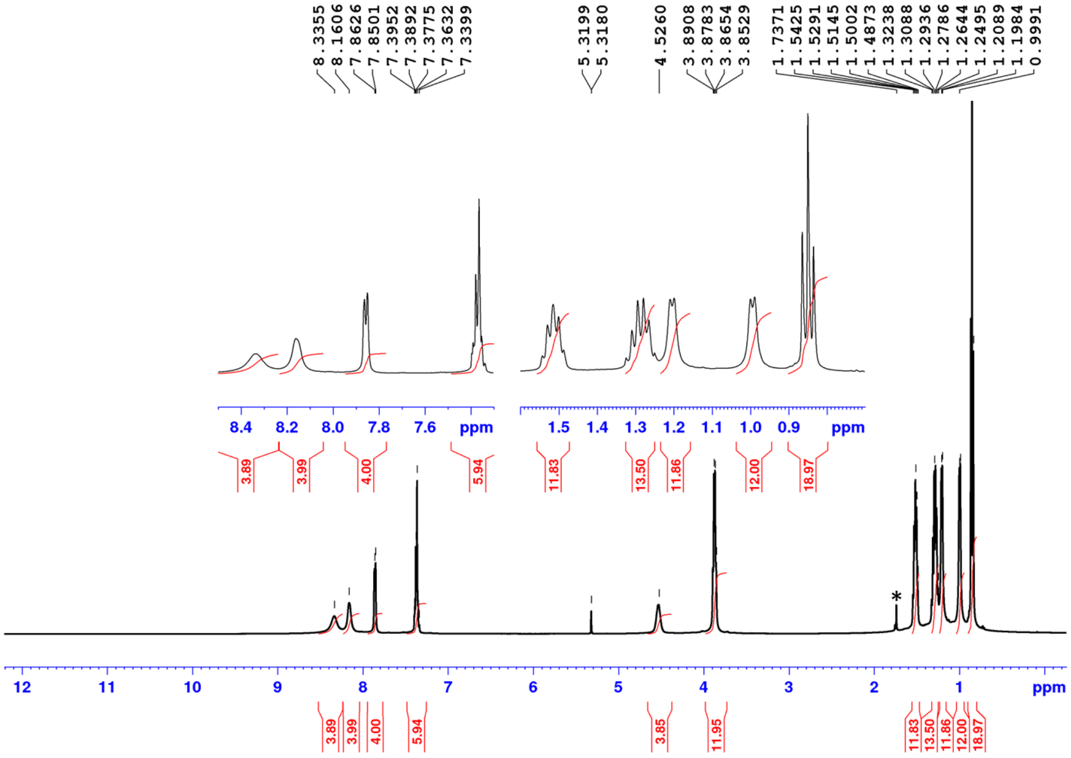

Figure S49. ^{1}H NMR of [$Li_2(\mathbf{L}^4)_2(TBP)_2$]$\cdot 0.2C_5H_{12}$ (CD_2Cl_2, 300 K, * H_2O)

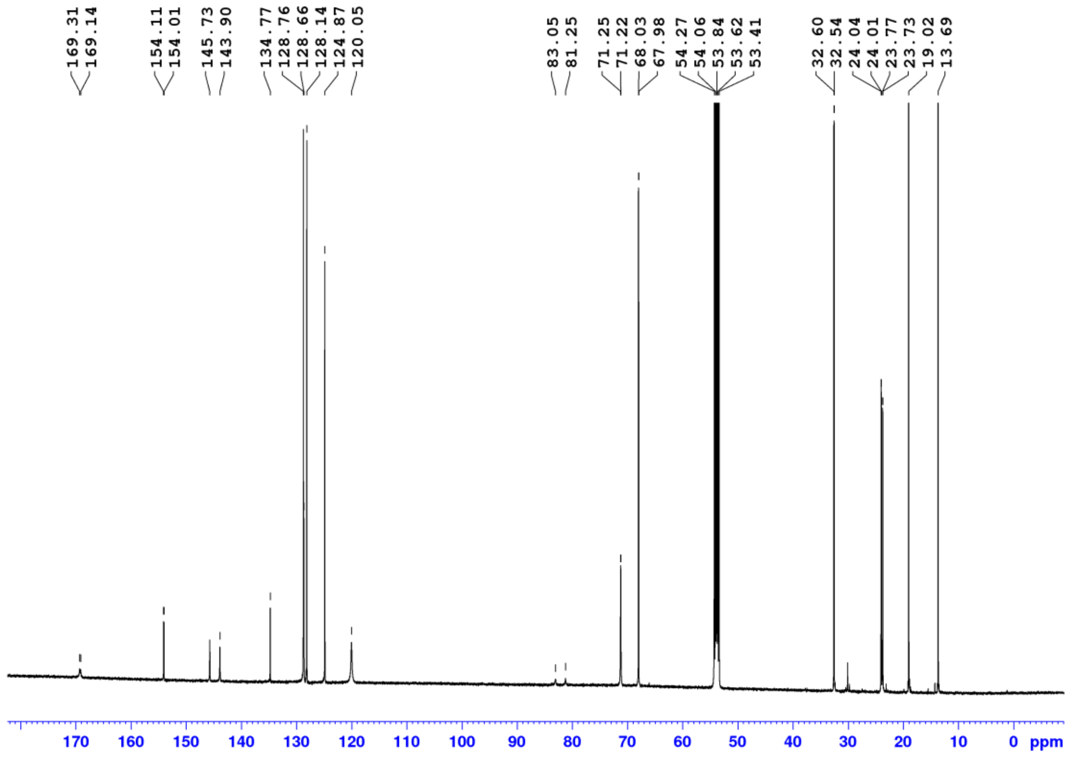

Figure S50. $^{13}C\{^{1}H\}$ NMR of $[Li_2(\mathbf{L}^4)_2(TBP)_2]\cdot 0.2C_5H_{12}$ (CD_2Cl_2, 300 K)

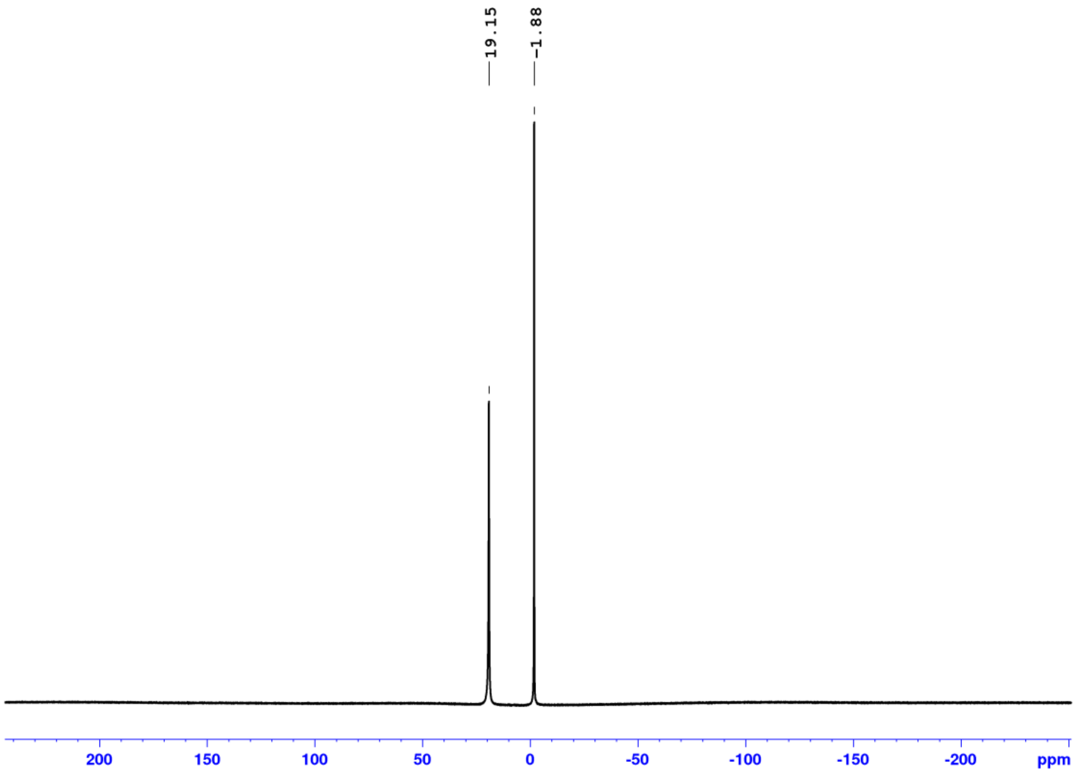

Figure S51. ^{31}P NMR of $[Li_2(\mathbf{L}^4)_2(TBP)_2]\cdot 0.2C_5H_{12}$ (CD_2Cl_2, 300 K)

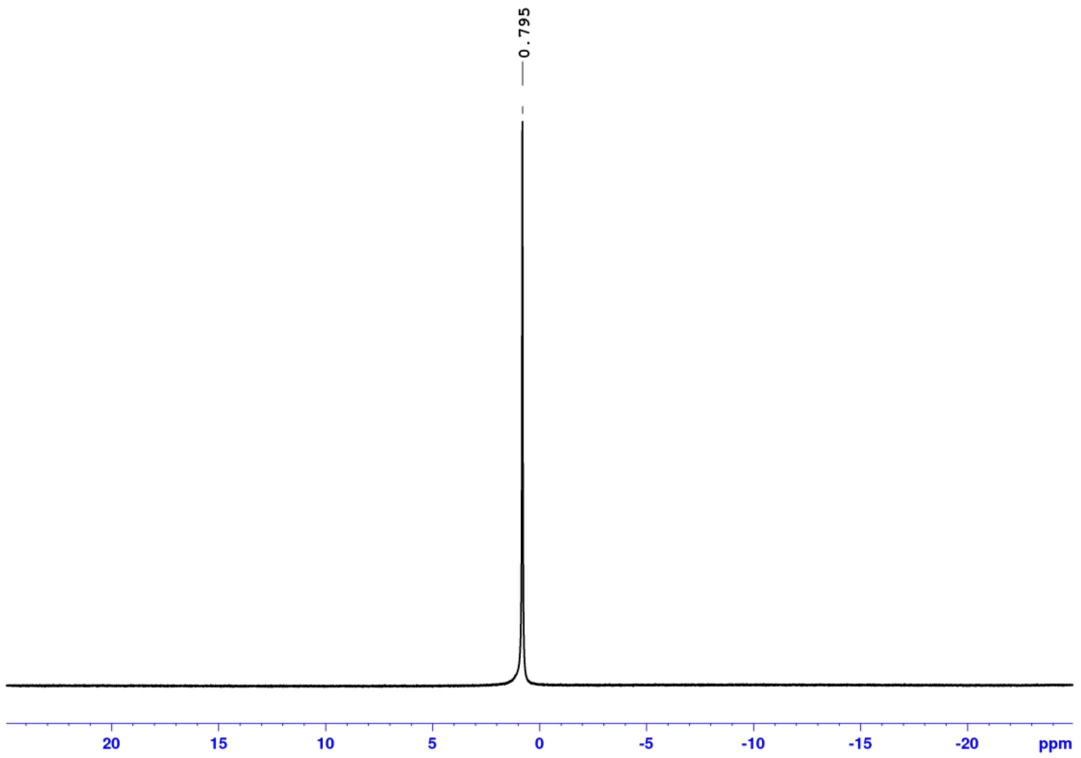

Figure S52. ^{7}Li NMR of [Li_2(**L**^{4})$_2$(TBP)$_2$]·$0.2C_5H_{12}$ (CD_2Cl_2, 300 K)

S4.5. Preparation of [$Li_3(L^3)_3$(TOPO)]·$0.65C_5H_{12}$ (12)

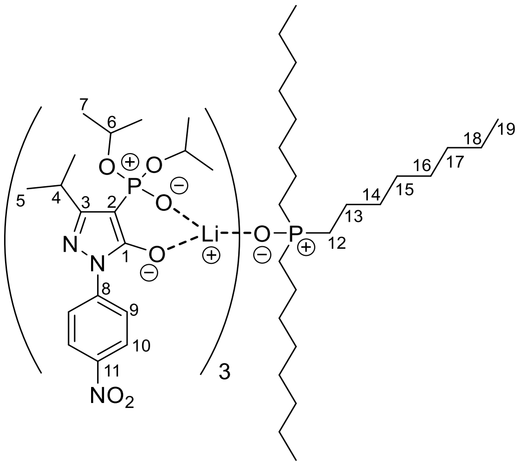

H**L**[3] (172.7 mg, 0.42 mmol, 1.0 eq.), trioctylphosphine oxide (TOPO) (53.1 mg, 0.14 mmol, 0.33 eq.) and LiOH·H_2O (18.4 mg, 0.43 mmol, 1.0 eq.) were reacted in 1.0 ml of CH_2Cl_2. The reaction mixture was stirred at room temperature overnight. The resulting yellow solution was filtered and single crystals suitable for X-ray diffraction analysis were obtained by diffusion of pentane into a solution of the complex in CH_2Cl_2 at -18 °C. Experiments using 0.5 eq. TOPO, 1.0 eq. H**L**[3] and 1.0 eq. LiOH·H_2O employing exactly the same experimental procedure resulted in suitable crystals for X-ray diffraction analysis that have a similar trinuclear structure to that obtained using H**L**[3], TOPO and LiOH·H_2O in a 1 : 0.33 : 1 ratio.

Yield: 91.1 mg (40%); **mp.**: 128 °C; **Raman** (100 mW, 298 K, in cm^{-1}): 2940 (6), 2914 (7), 1600 (13), 1584 (25), 1509 (7), 1323 (100), 1267 (7), 1168 (6), 1144 (5), 1113 (24), 1007 (8); **IR** (ATR, 298 K, in cm^{-1}): 2964 (vw), 2924 (w), 2854 (vw), 1607 (s), 1581 (m), 1505 (s), 1467 (w), 1421 (vw), 1385 (vw), 1377 (vw), 1354 (vw), 1321 (vs), 1295 (w), 1265 (m), 1161 (m), 1144 (vw), 1102 (s), 1089 (m), 1079 (w), 1066 (vw), 1034 (w), 1000 (vs), 986 (vs), 937 (w), 898 (w), 881 (w), 855 (m), 812 (vw), 801 (vw), 777 (m), 758 (m), 752 (m), 724 (vw), 703 (vw), 692 (w), 659 (w), 588 (m), 535 (w), 505 (w), 429 (vw); **^{1}H NMR** (CD_2Cl_2, 300K, in ppm): δ = 0.86 (9H, t, $^3J_{HH}$ = 7.2 Hz, H19), 1.09-1.49 (96H, m, H5, H7 and H12-18), 2.94 (3H, sept, $^3J_{HH}$ = 6.8 Hz, H4), 4.50 (6H, s, H6), 8.05 (6H, s, H9), 8.16 (6H, s, H10); **^{13}C{^{1}H} NMR** (CD_2Cl_2, 300K, in ppm): δ = 14.2 (3C, s, C19), 21.7 (3C, d, $^2J_{CP}$ = 4 Hz, C13), 22.0 (6C, s, C5), 23.0 (3C, s, C18), 24.1 (6C, d, $^3J_{CP}$ = 3 Hz, C7a), 24.1 (6C, d, $^3J_{CP}$ = 4 Hz, C7b), 27.1 (3C, d, $^1J_{CP}$ = 66 Hz, C12), 28.4 (3C, s, C4), 29.3 (3C, s, C16), 29.5 (3C, s, C15), 31.4 (3C, d, $^3J_{CP}$ = 15 Hz, C14), 32.1 (3C, s, C17), 71.0 (6C, d, $^2J_{CP}$ = 5 Hz, C6), 81.6 (3C, d, $^1J_{CP}$ = 232 Hz, C2), 119.7 (6C, s(br), C10), 124.8 (6C, s, C9), 143.4 (3C, s, C8), 146.0 (3C, s, C11), 161.1 (3C, d, $^2J_{CP}$ = 13 Hz, C3), 168.1 (3C, d, $^2J_{CP}$ = 24 Hz, C1); **^{31}P{^{1}H} NMR** (CD_2Cl_2, 300K, in ppm): δ = 20.0 (s), 57.4 (s(br)); **^{31}P NMR** (CD_2Cl_2, 300K, in ppm): δ = 20.0 (s), 57.4 (s(br)); **^{7}Li NMR** (CD_2Cl_2, 300K, in ppm): δ = 0.76 (s); **Elemental analysis** for $C_{78}H_{126}Li_3N_9O_{19}P_4$ [Li_3(**L**[3])$_3$(TOPO)], calculated: C 57.17, H 7.75, N 7.69; found: C 57.55, H 7.79, N 7.70; **ESI-MS** (m/z, [Da/e]): 410.1 [M-3Li-2**L**-TOPO]$^-$, 827.2 [M-2Li-**L**-TOPO]$^-$, 1244.5 [M-Li-TOPO]$^-$.

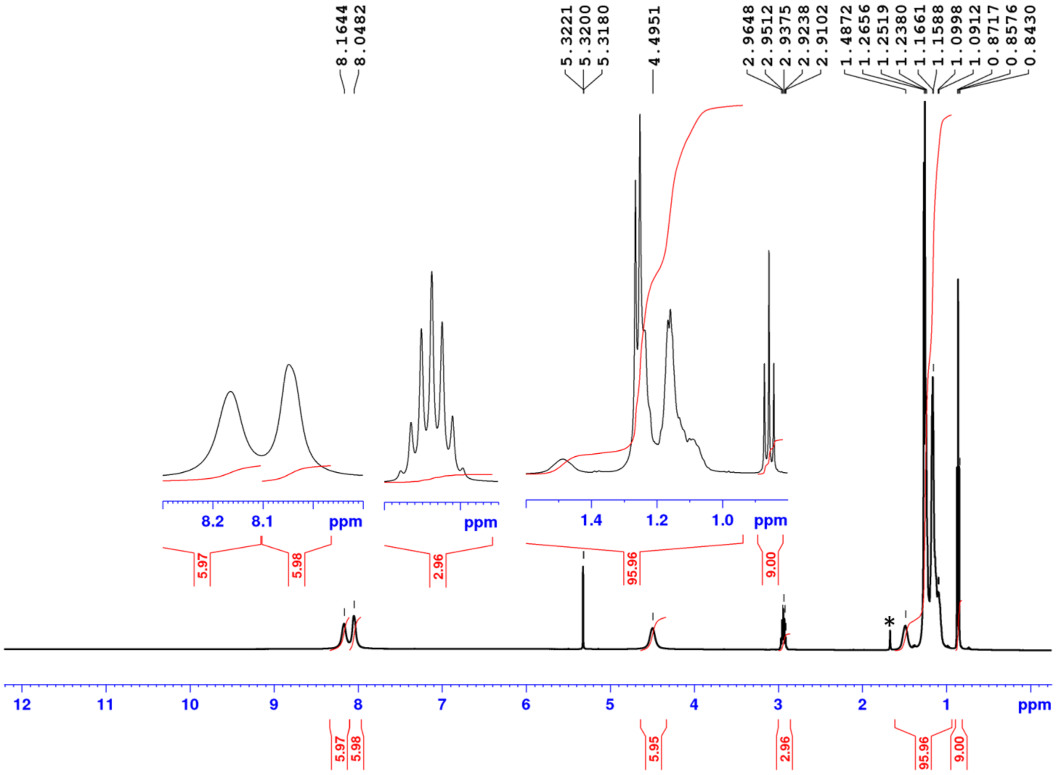

Figure S53. ^{1}H NMR of [Li_3(**L**^{3})$_3$(TOPO)] (CD_2Cl_2, 300 K, * H_2O)

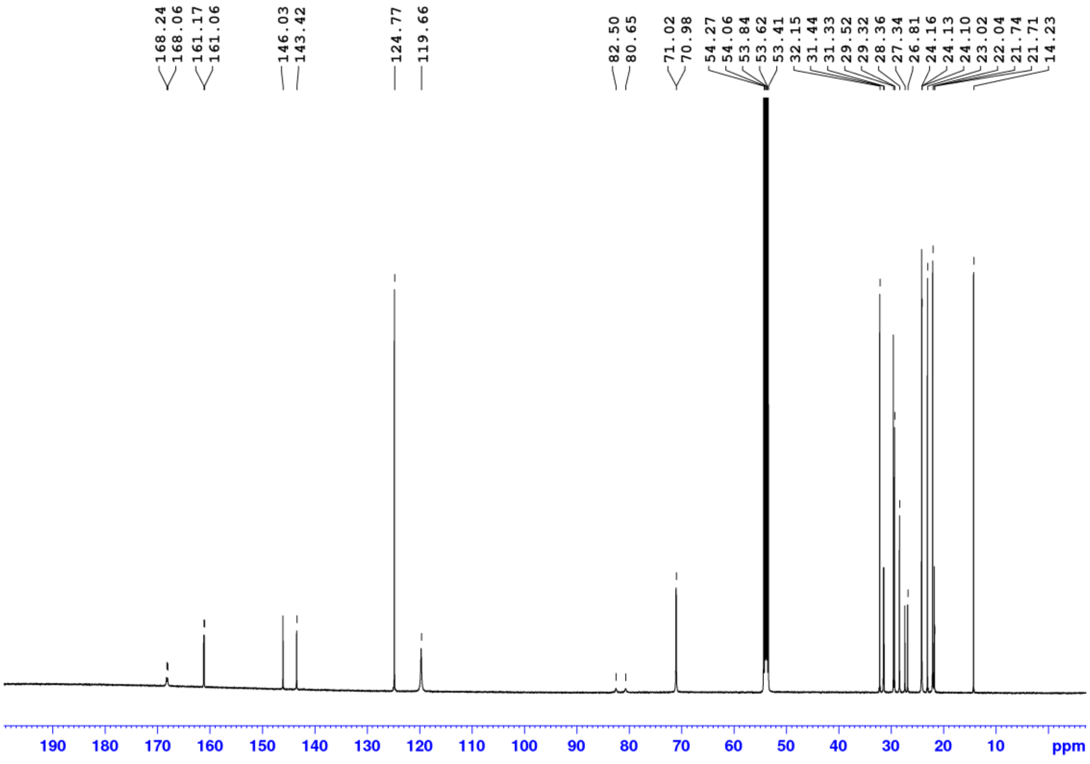

Figure S54. $^{13}C\{^{1}H\}$ NMR of [Li_3(**L**^{3})$_3$(TOPO)] (CD_2Cl_2, 300 K)

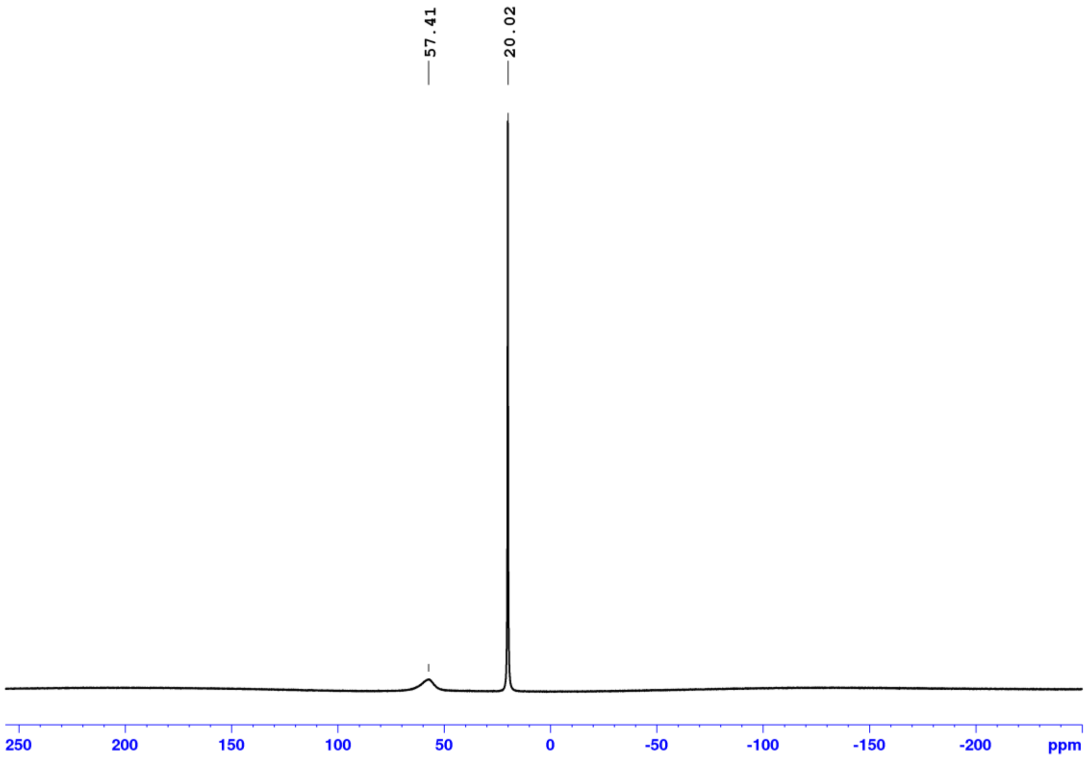

Figure S55. ^{31}P NMR of [Li_3(**L**^{3})$_3$(TOPO)] (CD_2Cl_2, 300 K)

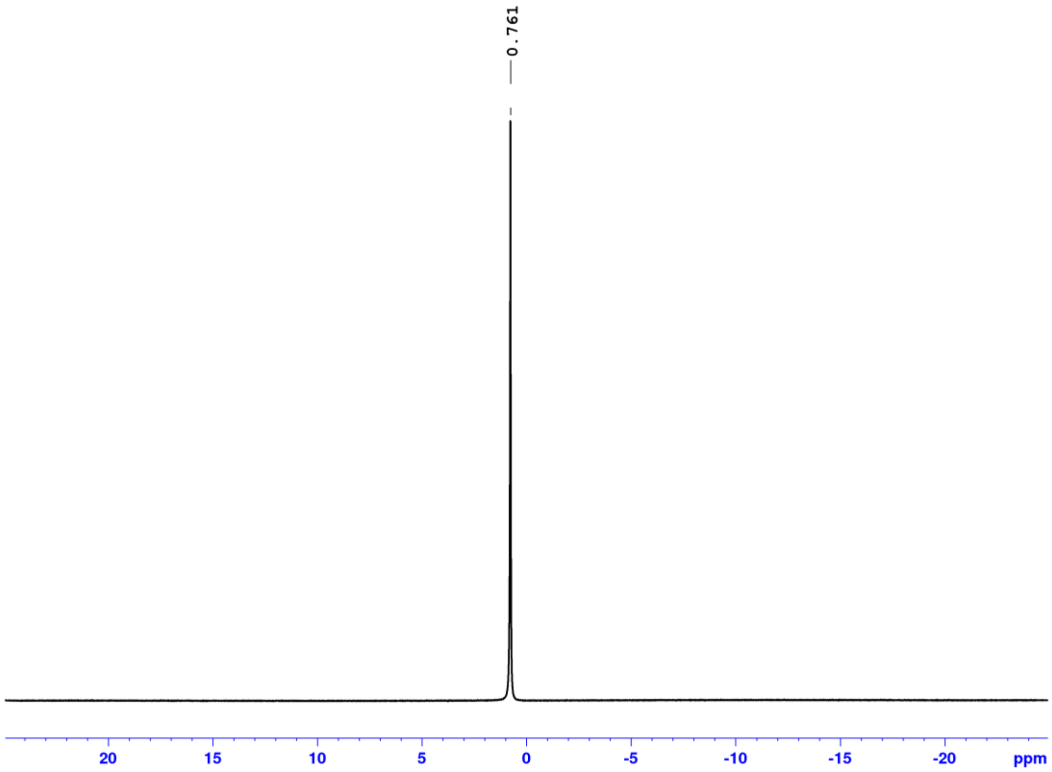

Figure S56. ^7Li NMR of [Li_3(**L**^{3})$_3$(TOPO)] (CD_2Cl_2, 300 K)

S4.6. Preparation of $[Li_3(L^4)_3(TOPO)]\cdot 0.67C_5H_{12}$ (13)

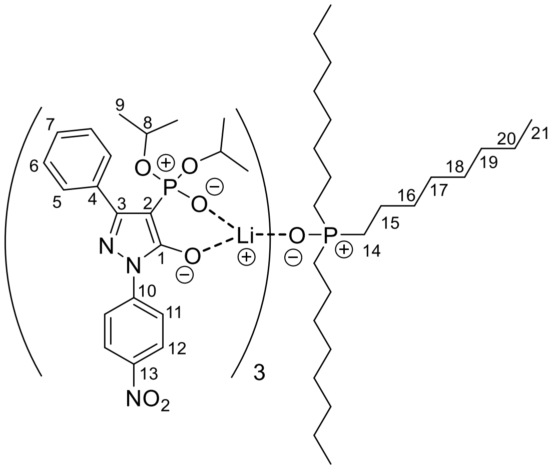

H**L**[4] (72.0 mg, 0.16 mmol, 1.0 eq.), trioctylphosphine oxide (TOPO) (61.2 mg, 0.16 mmol, 1.0 eq.) and $LiOH\cdot H_2O$ (7.2 mg, 0.17 mmol, 1.0 eq.) were added to 0.8 ml CH_2Cl_2. The reaction mixture was stirred for 5 h at room temperature. After filtration, 4 ml of pentane were slowly added to the filtrate with stirring and the resulting solution was stored at -18 °C for one week. The yellow precipitate was collected by filtration and dried *in vacuo*. Single crystals suitable for X-ray diffraction analysis were obtained by diffusion of pentane into a solution of the complex in CH_2Cl_2 at -18 °C. Experiments using H**L**[4], LiOH and TOPO in a ratio of 1 : 1 : 0.5 and 1 : 1 : 2 (H**L**[4] : LiOH : TOPO) were also performed. For the products of both experiments, single crystals were grown by diffusion of pentane into a solution of CH_2Cl_2 or $CHCl_3$, respectively. X-ray diffraction analysis show that a trinuclear structure of the type mentioned above was obtained in each case.

Yield: 41.8 mg (45%); **mp.**: 178 °C; **Raman** (100 mW, 298 K, in cm^{-1}): 1608 (18), 1586 (24), 1513 (11), 1480 (8), 1443 (10), 1322 (100), 1288 (8), 1161 (8), 1139 (5), 1113 (22), 969 (11); **IR** (ATR, 298 K, in cm^{-1}): 2975 (vw), 2957 (vw), 2923 (w), 2853 (vw), 1608 (s), 1583 (m), 1512 (m), 1495 (m), 1478 (w), 1444 (vw), 1402 (w), 1321 (vs), 1285 (m), 1191 (vw), 1165 (w), 1101 (s), 1076 (w), 1020 (vs), 984 (vs), 967 (s), 939 (w), 927 (w), 898 (w), 885 (w), 855 (m), 811 (vw), 800 (vw), 779 (m), 750 (s), 724 (vw), 700 (w), 672 (w), 631 (vw), 611 (s), 559 (w), 531 (w), 511 (vw), 489 (w), 432 (vw); **^{1}H NMR** (CD_2Cl_2, 300K, in ppm): δ = 0.84 (9H and ~ 0.3 eq. C_5H_{12}, t, $^3J_{HH}$ = 7.2 Hz, H21), 0.98-1.63 (78H and ~ 0.3 eq. C_5H_{12}, H9 and H14-H20), 4.45 (6H, s(br), H8), 7.34-7.40 (9H, m, H7 and H6), 7.83-7.85 (6H, m, H5), 8.09 (6H, d, $^3J_{HH}$ = 6.3 Hz, H11), 8.29 (6H, d, $^3J_{HH}$ = 5.9 Hz, H12); **^{13}C{^{1}H} NMR** (CD_2Cl_2, 300K, in ppm): δ = 14.2 (3C, s, C21), 21.8 (3C, d, $^2J_{CP}$ = 4 Hz, C15), 23.0 (3C, s, C20), 23.8 (6C, d, $^3J_{CP}$ = 5 Hz, C9a), 24.0 (6C, d, $^3J_{CP}$ = 4 Hz, C9b), 27.2 (3C, d, $^1J_{CP}$ = 66 Hz, C14), 29.4 (3C, s, C18), 29.6 (3C, s, C17), 31.5 (3C, d, $^3J_{CP}$ = 15 Hz, C16), 32.2 (3C, s, C19), 71.4 (6C, d, $^2J_{CP}$ = 6 Hz, C8), 82.0 (3C, d, $^1J_{CP}$ = 232 Hz, C2), 120.2 (6C, s(br), C12), 124.9 (6C, s, C11), 128.1 (6C, s, C6), 128.7 (3C, s, C7), 128.8 (6C, s, C5), 134.7 (3C, s, C4), 143.9 (3C, s, C10), 145.6 (3C, s, C13), 154.2 (3C, d, $^2J_{CP}$ = 12 Hz, C3), 168.9 (3C, d, $^1J_{CP}$ = 23 Hz, C1); **^{31}P{^{1}H} NMR** (CD_2Cl_2, 300K, in ppm): δ = 19.2 (s), 57.2 (s(br)); **^{31}P NMR** (CD_2Cl_2, 300K, in ppm): δ = 19.2 (s), 57.2 (s(br)); **^{7}Li NMR** (CD_2Cl_2, 300K, in ppm): δ = 0.89 (s); **Elemental analysis** for $C_{87}H_{120}Li_3N_9O_{19}P_4$ [Li_3(**L**^{4})$_3$(TOPO)], calculated: C 60.03, H 6.95, N 7.24; found: C 60.04, H 6.67, N 7.25; **ESI-MS** (m/z, [Da/e]): 444.1 [M-3Li-2**L**-TOPO]$^-$, 895.2 [M-2Li-**L**-TOPO]$^-$, 1346.4 [M-Li-TOPO]$^-$.

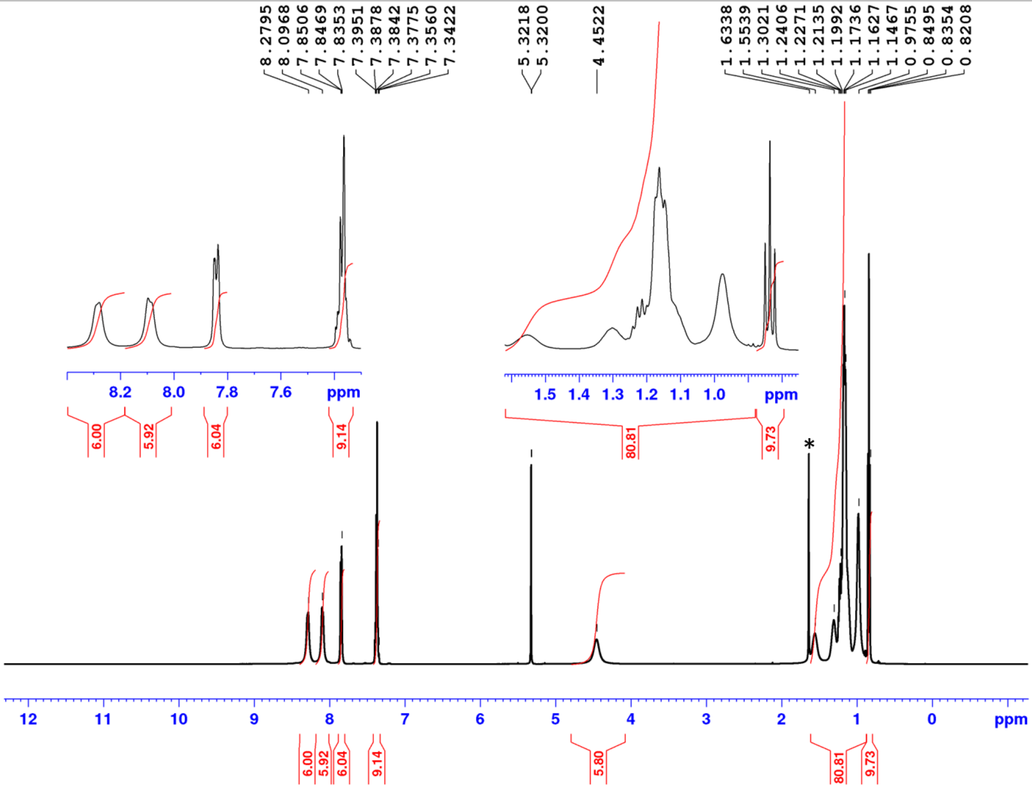

Figure S57. ^{1}H NMR of [Li_3(**L**^{4})$_3$(TOPO)] (CD_2Cl_2, 300 K, * H_2O)

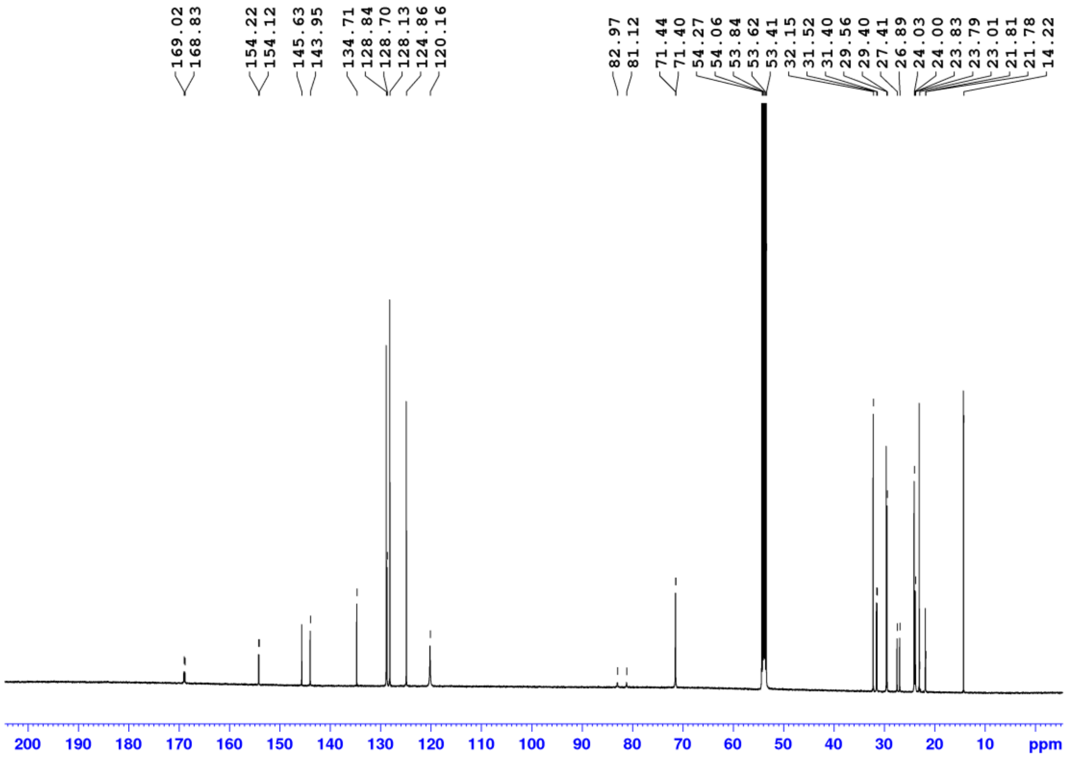

Figure S58. $^{13}C\{^{1}H\}$ NMR of [Li_3(**L**^{4})$_3$(TOPO)] (CD_2Cl_2, 300 K)

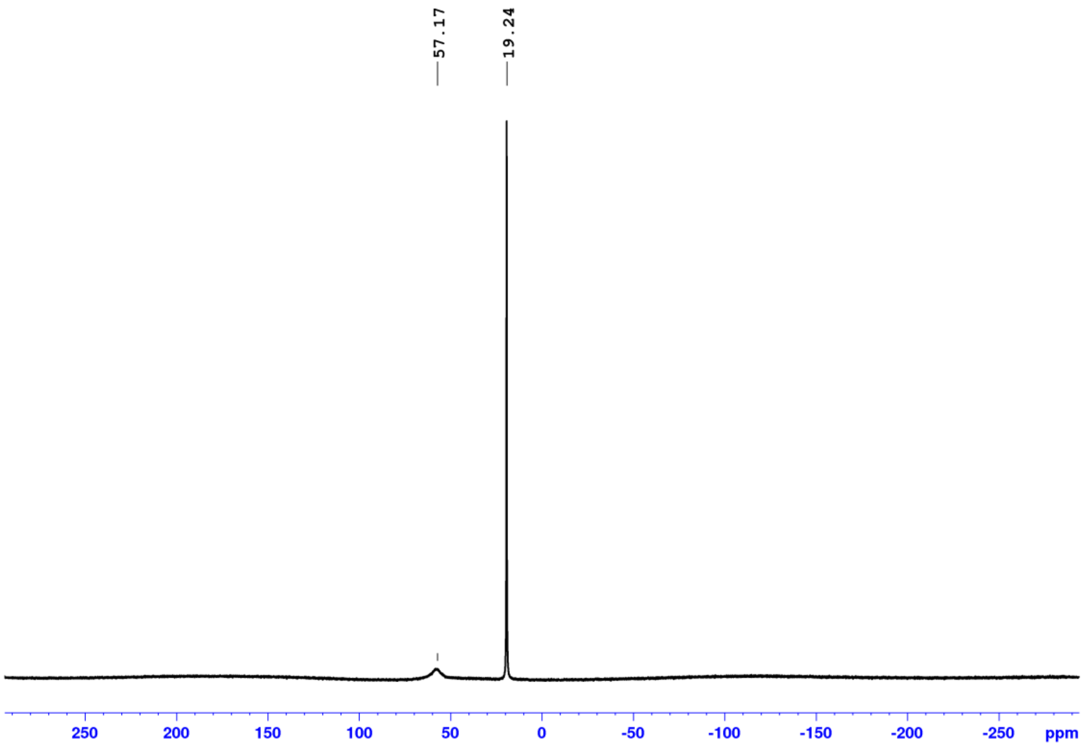

Figure S59. ^{31}P NMR of [Li_3(**L**^{4})$_3$(TOPO)] (CD_2Cl_2, 300 K)

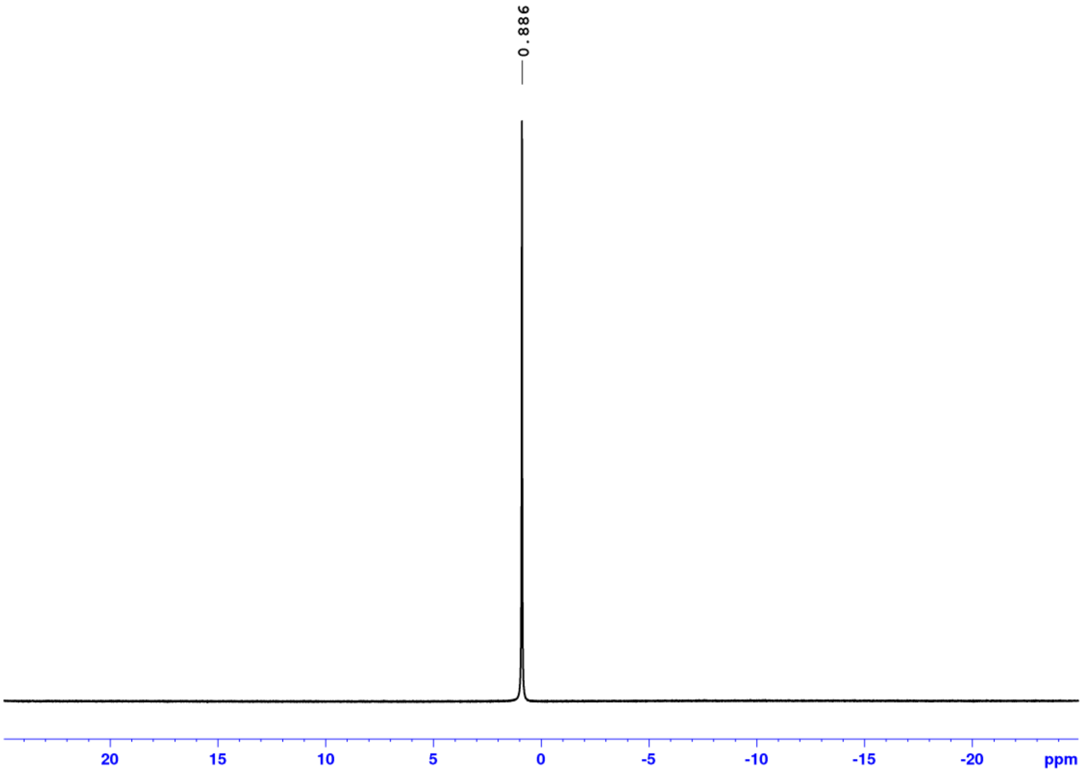

Figure S60. ^{7}Li NMR of [Li_3(**L**^{4})$_3$(TOPO)] (CD_2Cl_2, 300 K)

S5. Crystal structures

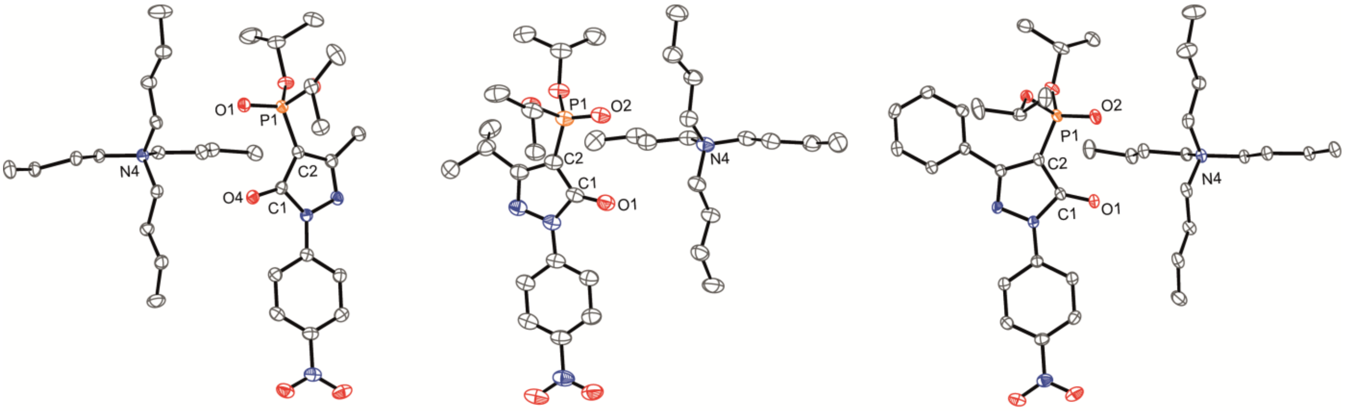

[TBA]**L**[2] [TBA]**L**[3] [TBA]**L**[4]

Figure S61. Molecular structures of [TBA]**L**[2] - [TBA]**L**[4] (hydrogen atoms are omitted for clarity and ellipsoids are drawn at 50% probability level).

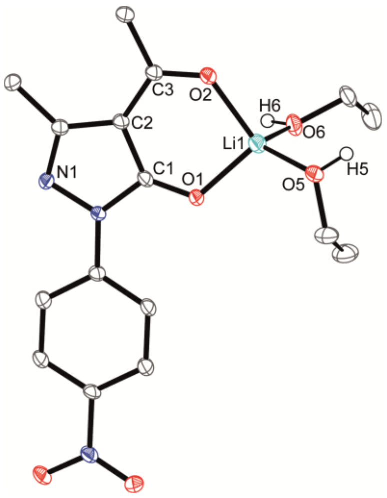

Figure S62. Molecular structures of $[Li(\mathbf{L}^1)(EtOH)_2]$ (**7**) (all carbon hydrogen atoms are omitted for clarity and ellipsoids are drawn at 50% probability level).

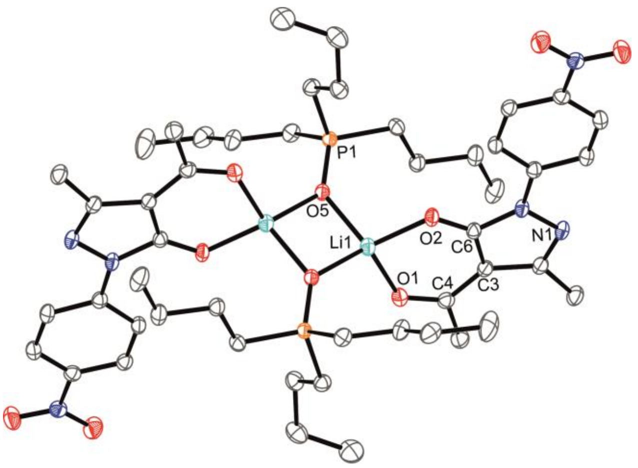

Figure S63. Molecular structures of $[Li_2(\mathbf{L}^1)_2(TBPO)_2]$ (**8**) (all hydrogen atoms are omitted for clarity and ellipsoids are drawn at 50% probability level).

S6. NMR studies

S6.1 Method of Continuous Variation

The method of continuous variation (Job's method) was employed to determine the stoichiometry of the lithium complex species in solution. A 9 mM $LiClO_4$/[TBA]**L**[4] mixture solution was prepared by stirring [TBA]**L**[4] and $LiClO_4$ in CD_2Cl_2 in the glove-box. The yellowish solution was filtered and used without further modification. A 9 mM TOPO solution was prepared in CD_2Cl_2. The mole ratio between $LiClO_4$/[TBA]**L**[4] and TOPO were altered by changing the added volumes (Table S1) while keeping the total concentration of $LiClO_4$/[TBA]**L**[4] and TOPO constant (9 mM). Each solution was stirred for 4 h before undertaking the NMR measurements.

Table S1. Preparation details for achieving different mole ratios

Total V (µl)	$V_{LiClO4/[TBA]L4}$ (9 mM, µl)	V_{TOPO} (9 mM, µl)	$x_{LiClO4/[TBA]L4}$	x_{TOPO}
500	500	0	1	0
500	475	25	0.95	0.05
500	450	50	0.9	0.1
500	425	75	0.85	0.15
500	400	100	0.8	0.2
500	375	125	0.75	0.25
500	350	150	0.7	0.3
500	300	200	0.6	0.4
500	250	250	0.5	0.5
500	200	300	0.4	0.6
500	175	325	0.35	0.65
500	150	350	0.3	0.7
500	100	400	0.2	0.8
500	50	450	0.1	0.9
500	25	475	0.05	0.95

Partial ^{31}P NMR spectra and Job-plot diagram are depicted in Figure S64.

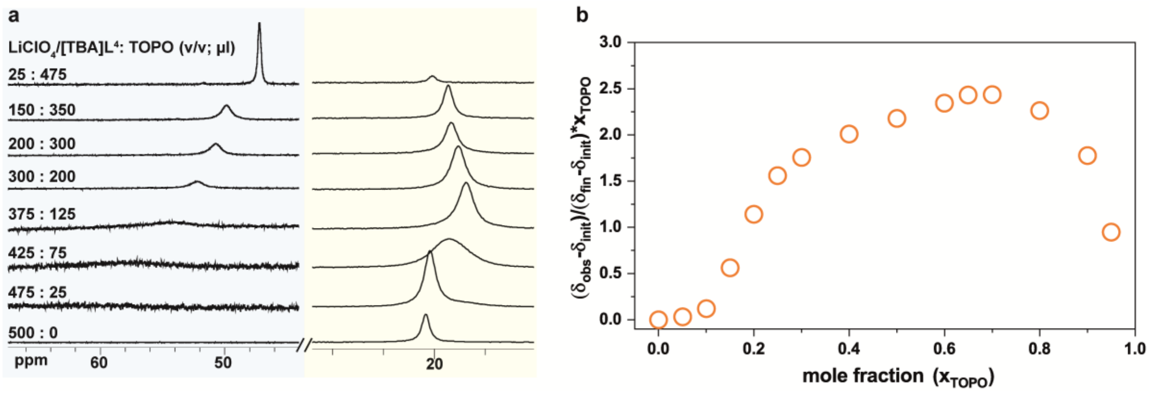

Figure S64. a) Stacked cut-out of the ^{31}P NMR spectra of TOPO and $[L^4]^-$ for different mole ratios of $LiClO_4$/[TBA]**L**[4] and TOPO (CD_2Cl_2, 300 K); b) ^{31}P of $[L^4]^-$ Job-plots, X-axis represents the mole fraction of TOPO while the Y-axis represents $\frac{\delta_{observed}-\delta_{initial}}{\delta_{final}-\delta_{initial}} * x_{TOPO}$.

S6.2 Mole Ratio Method

A 9 mM $LiClO_4$/[TBA]**L**[4] mixture solution was prepared according to the procedure outlined in the above continuous variation method section. A 36 mM TOPO solution was also prepared in CD_2Cl_2. The concentration of $LiClO_4$/[TBA]**L**[4] in each sample was constant (7.2 mM, Table S2) and the TOPO eq. were altered by changing the added volume of TOPO solution and CD_2Cl_2. Each solution was stirred for 4 h before the NMR measurements. The preparation details of each sample are listed in Table S2.

Table S2. Preparation details of different eq. TOPO

C_{TOPO} : $C_{LiClO4/[TBA]L4}$	Total V (µl)	$V_{LiClO4/[TBA]L4}$ (9 mM, µl)	V_{TOPO} (36 mM, µl)	V_{CD2Cl2} (µl)
0.1	500	400	10	90
0.2	500	400	20	80
0.3	500	400	30	70
0.4	500	400	40	60
0.5	500	400	50	50
0.6	500	400	60	40
0.7	500	400	70	30
0.8	500	400	80	20
0.9	500	400	90	10
1.0	500	400	100	0

Table S3. Chemical shifts of ^{31}P and ^{7}Li NMR with increasing number of TOPO eq. and the trinuclear complex **13** and the dinuclear complex **10** (CD_2Cl_2, 300 K)

TOPO eq.	$^{31}P_{Ligand}$ (ppm)	$^{31}P_{TOPO}$ (ppm)	^{7}Li (ppm)
0	20.24	\	0.86
0.1	20.09/19.59*	\	0.87
0.2	19.87*/19.32	\	0.88
0.3	19.32	54.88	0.89
**13**	19.24	57.17	\
0.4	19.36	53.23	0.88
0.5	19.41	52.67	0.86
0.6	19.45	52.66	0.83
0.7	19.49	52.52	0.82
0.8	19.50	52.31	0.79
0.9	19.48	52.19	0.76
1.0	19.53	52.09	0.75
**10**	19.34	\	\

*given by the deconvolution results below.

The deconvolution of the ^{31}P NMR spectra was performed using the software TopSpin3.2 (Bruker). The line shapes are fitted applying Lorentz/Gauss functions of the corresponding signals or spectral areas. Table S4 and S5 summarize the parameters of the deconvolution analyses.

Table S4. Deconvolution results for the ^{31}P NMR of [**L**^{4}]$^-$ in the presence of 0.1 eq. of TOPO.

**Fit**	**Frequency**		**Width**		**Intensity**	**Area**
	**ppm**	**Hz**	**ppm**	**Hz**		
**1**	20.087	4066.78	0.61078	123.657	14.886	**3183.441**
STD	0.001	0.11	0.00169	0.342	0.022	
**2**	19.588	3965.66	0.62829	127.202	6.178	**1359.014**
STD	0.027	5.44	0.15560	31.502	0.364	

Table S5. Deconvolution results for the ^{31}P NMR of [**L**^{4}]$^-$ in the presence of 0.2 eq. TOPO.

**Fit**	**Frequency**		**Width**		**Intensity**	**Area**
	**ppm**	**Hz**	**ppm**	**Hz**		
**1**	19.865	4021.70	0.77885	157.683	7.372	**4020.502**
STD	0.002	0.41	0.00469	0.949	0.042	
**2**	19.323	3912.08	0.73078	147.951	12.571	**6433.144**
STD	0.001	0.22	0.00264	0.535	0.044	

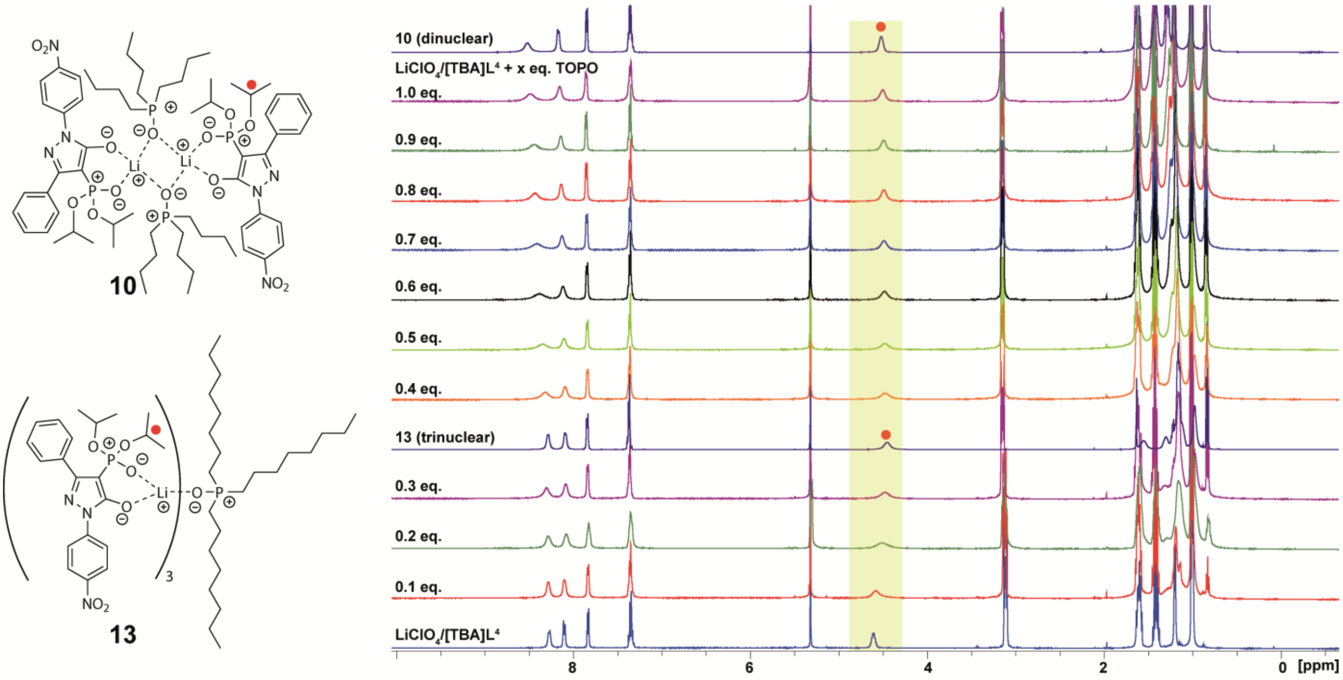

Figure S65. Full ^{1}H NMR spectra of a mixture of $LiClO_4$/[TBA]**L**^{4} and with increasing TOPO eq. and the trinuclear complex **13** and dinuclear complex **10** (CD_2Cl_2, 300 K)

The reversible structural transformation NMR investigation was started employing a 3 : 3 : 1 (Li^+ : [**L**^{4}]$^-$: TOPO) ratio, which was prepared from 375 µl of $LiClO_4$/[TBA]**L**^{4} and 125 µl of TOPO (both solutions were 9 mM in CD_2Cl_2, same as used for the solution data in Table S1). A following 3 : 3 : 3 (Li^+ : [**L**^{4}]$^-$: TOPO) stoichiometry prior the next NMR measurement was achieved by the addition of a further 250 µl TOPO (9 mM in CD_2Cl_2) into the above sample. Then 300 µl mixture solution used above (3 : 3 : 3 (Li^+ : [**L**^{4}]$^-$: TOPO)) was taken out and mixed with another 300 µl $LiClO_4$/[TBA]**L**^{4} (9 mM in CD_2Cl_2) to form a 3 : 3 : 1 (Li^+ : [**L**^{4}]$^-$: TOPO) stoichiometry again. Finally, 300 µl mixture solution used in the last step (3 : 3 : 1 (Li^+ : [**L**^{4}]$^-$: TOPO)) was taken out and mixed with another 150 µl TOPO (9 mM in CD_2Cl_2) and 150 µl blank CD_2Cl_2 to obtain 3 : 3 : 3 (Li^+ : [**L**^{4}]$^-$: TOPO) ratio. The mixture in each step was stirred for 4 h before NMR measurements (Figure 6b).

S7. Mass Spectral studies

The ESI-MS samples were prepared by dissolving the complexes **12** and **13** in methanol. In order to aid ionization and hopefully also gain additional information, 100 µg/ml LiCl solution was added to the complex solution and this resulted in more peaks becoming detectable (Figure S66 for **12**). Furthermore, experiments were carried out variating the TOPO concentration. A methanol solution of **13** with a concentration of 0.025 mM was used and TOPO in approximately 5, 10 and 20 eq. was added. The Y-axis gives the relative values based on the highest peak intensity observed for each sample.

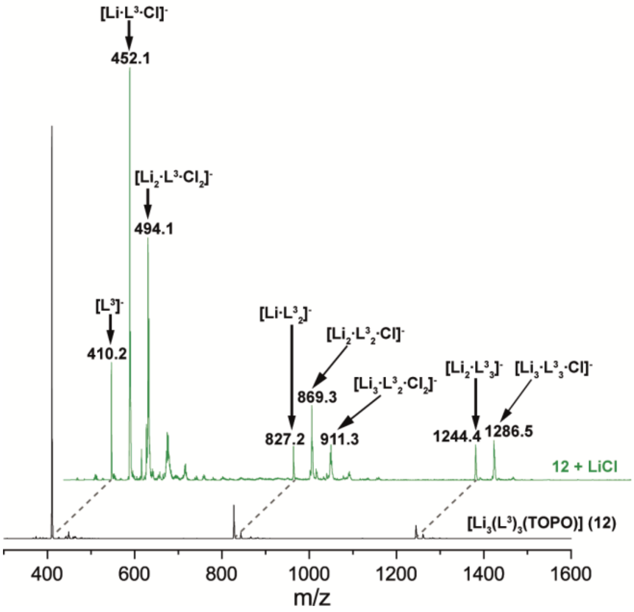

Figure S66. ESI-MS (negative-ion mode) of **12** complex in CH_3OH and after addition of excess LiCl (green).

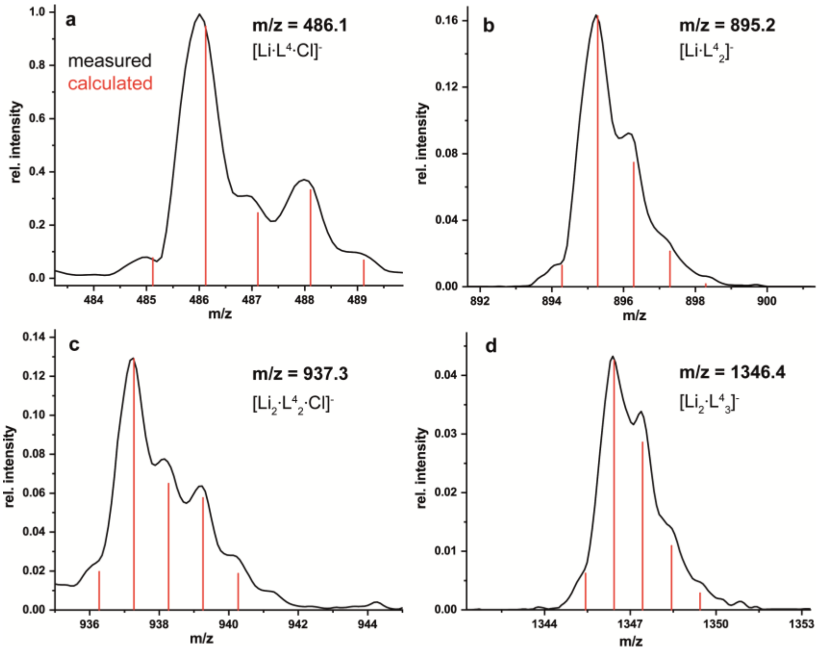

Figure S67. Representative signals for **13** in CH_3OH (in the presence of excess LiCl) and their corresponding isotope patterns.

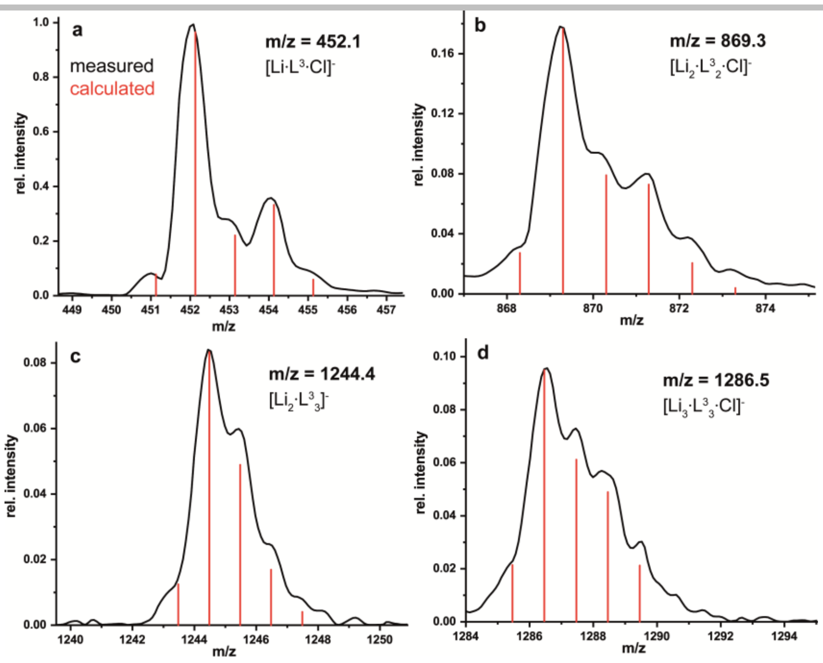

Figure S68. Representative signals for **12** in CH_3OH (in the presence of excess LiCl) and their corresponding isotope patterns.

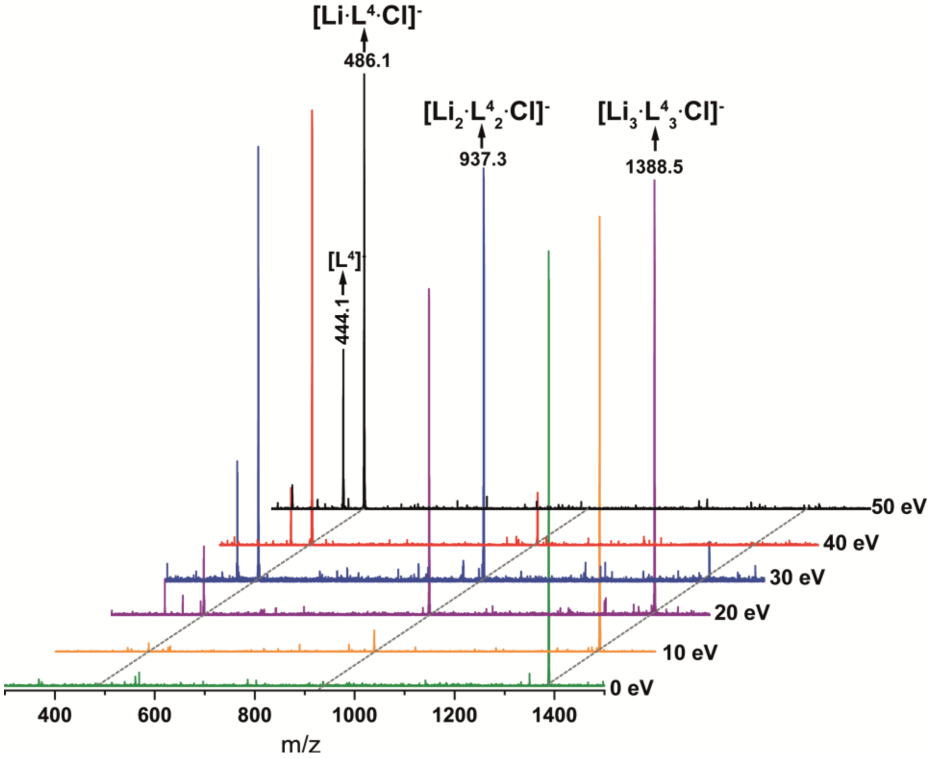

Figure S69. ESI-MS (negative-ion mode) showing daughter species (m/z = 1388.5) for **13** in CH_3OH with excess LiCl corresponding to different collision energies.

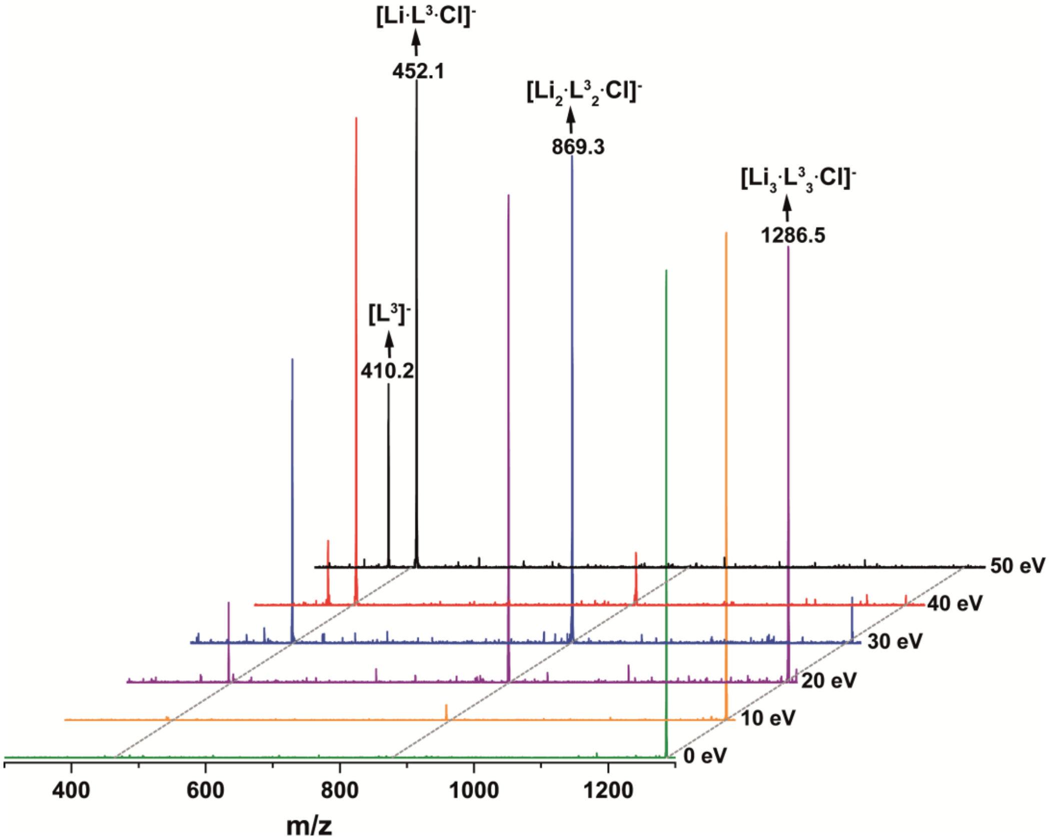

Figure S70. ESI-MS (negative-ion mode) of daughter species (m/z = 1286.5) for **12** in CH_3OH with excess LiCl corresponding to different collision energies.

S8. n-octanol/water distribution

Information about the lipophilicity of ligands H**L**^{3} and H**L**^{4} have been obtained by distribution measurements of the ligands in the n-octanol/water system.[7] A buffer solution (Tris/HCl, pH = 8.3) and n-octanol, saturated with each other have been used. The experiments were performed with a ligand concentration of 1 x 10^{-4} M in n-octanol, a phase ratio of 1 : 1 ($V_{(aq)}$: $V_{(org)}$ = 1 : 1; 800 µl each) and a contact time of 2 h. After separation of the phases, the amount of ligand in n-octanol was monitored by UV/Vis spectroscopy (Lambda 2, PerkinElmer) using the absorption maxima between 300 and 400 nm. The results of the determined n-octanol/water distribution are summarized in Table S6 and show for both ligands an occurrence of approximately 90 % in the organic phase.

Table S6. Distribution of the free ligands between n-octanol and water (Tris/HCl buffer with pH = 8.3); t = 2 h.

Ligand	n-octanol/water content in %
H**L**^{3}	90/10
H**L**^{4}	90/10

S9. Li^+ single element extraction under LLE conditions

The concentration of metals in the organic phase c_{org} was calculated based on the difference of the metal concentration in the aqueous phase before (c_0) and after (c_e) extraction via mass balance. Distribution ratio (*D*) was defined as the ratio of the concentration of metal present in the organic phase to the concentration of metal present in the aqueous phase (Eq. (S1)). The percent extraction (*E* [%]) was calculated from the distribution ratio by Eq. (S2):

$$D = \frac{(c_o - c_e)}{c_e} \tag{S1}$$

$$E[\%] = \frac{D}{(D + \frac{V_{(aq)}}{V_{(org)}})} \cdot 100 \tag{S2}$$

where c_0 and c_e (mg·L^{-1}) are initial and equilibrium concentration of metal in the aqueous phase and $V_{(aq)}$ and $V_{(org)}$ (mL) refer to the volumes of aqueous and organic phases. The phase ratio $V_{(aq)}$: $V_{(org)}$ was 1 : 1 in all experiments. The Tris/HCl buffer was prepared by 0.1 M Tris (tris(hydroxymethyl)aminomethane) and 0.1 M HCl. All liquid-liquid extraction experiments involved the mechanical shaking of the two-phase system. The extraction experiments were performed in duplicate, and both the average value and standard deviation are reported.

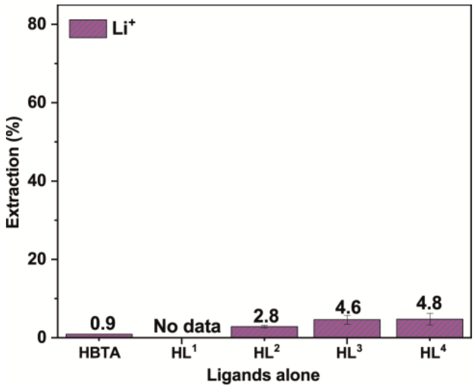

Figure S71. Percent extraction of Li^+ with different ligands in the absence of TOPO. Due to the high hydrophilic nature of H**L**[1], no data is available for this ligand. Experiment conditions: [LiCl] = 0.01 M, [NH_4Cl] = 0.1 M, pH = 8.5 (Tris/HCl buffer), [H**L**] = 0.1 M in $CHCl_3$, 298 K, 1 h.

S10. Slope analysis and loading experiments

The reaction in liquid-liquid extraction can be written by Eq. (S3):

$$Li^+_{(aq)} + m\mathrm{H}\mathbf{L}_{(org)} + n\mathrm{S}_{(org)} = \mathrm{Li}\cdot(\mathrm{H}_{m-1}\mathbf{L}_m)\cdot\mathrm{S}_{n(org)} + H^+_{(aq)} \tag{S3}$$

where H**L** is the ligand and S is the co-ligand (TOPO) with the stoichiometry coefficients *m* and *n*. The index aq and org refer to the aqueous and organic phase.

The extraction constant K_{ex} can be expressed as Eq. (S4).

$$K_{ex} = \frac{[\mathrm{Li}\cdot(\mathrm{H}_{m-1}\mathbf{L}_m)\cdot S_n]_{(org)}\cdot[\mathrm{H}^+]_{(aq)}}{[\mathrm{Li}^+]_{(aq)}\cdot[\mathrm{H}\mathbf{L}]^m_{(org)}\cdot[\mathrm{S}]^n_{(org)}} \tag{S4}$$

With the distribution ratio (D_{Li}) defined as the ratio of the concentration of metal present in the organic phase to the concentration of metal present in the aqueous phase ($\frac{[\mathrm{Li}\cdot(\mathrm{H}_{m-1}\mathbf{L}_m)\cdot S_n]_{(org)}}{[\mathrm{Li}^+]_{(aq)}}$) Eq. (S5) can be accessed:

$$\log D_{Li} = \log K_{ex} + m\log[\mathrm{H}\mathbf{L}] + n\log[\mathrm{S}] + \mathrm{pH} \tag{S5}$$

For a simple reactive extraction, according to Eq. (S5) the distribution ratio depends merely on the pH, the ligand concentration and the concentration of the co-ligand. If the pH value and the concentration of the co-ligand are set constant the coefficient *m* can be

determined from the slope of a logD_{Li}-log[H**L**]$_{(org)}$ plot. Likewise, the coefficient *n* or the amount of protons released through the extraction process can be derived from the slope of a logD_{Li}-log[TOPO]$_{(org)}$ and logD_{Li}-pH plot. Note that the method is only valid if the ligand and co-ligand concentration exceeds the metal concentration, so that the amount of complex species formed can be neglected when calculating log[H**L**]$_{(org)}$ or log[TOPO]$_{(org)}$. In addition, if possible, a constant ionic strength must be used in the aqueous phase in order to minimize the influence of the change in the activity coefficient on the concentration-dependent extraction constant. The counterion is used in large excess for this purpose.

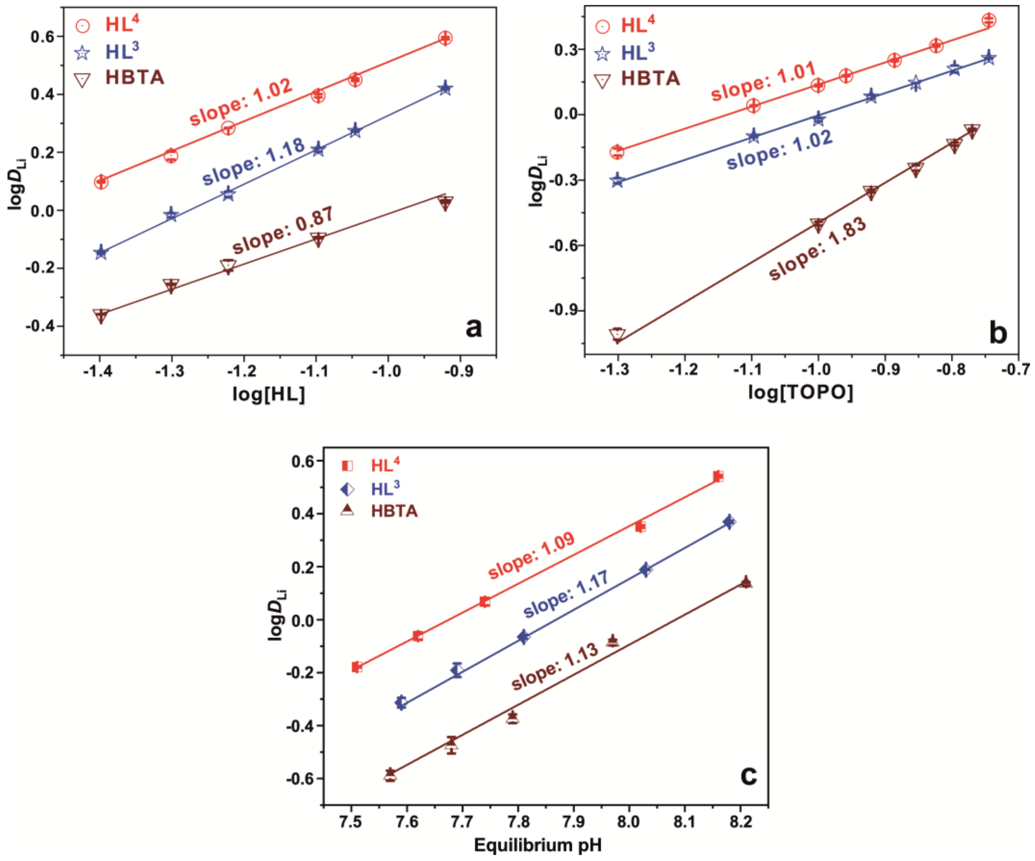

Figure S72. Dependence of the extraction of Li^+ on a) ligand concentration, conditions: [LiCl] = 0.01 M, [NH_4Cl] = 0.1 M, pH = 8.2 (Tris/HCl buffer), [H**L**] = 0.04 - 0.12 M, [TOPO] = 0.2 M in $CHCl_3$, 298 K, 1 h; b) TOPO concentration, conditions: [LiCl] = 0.01 M, [NH_4Cl] = 0.1 M, pH = 8.2 (Tris/HCl buffer), [H**L**] = 0.1 M, [TOPO] = 0.05 - 0.18 M in $CHCl_3$, 298 K, 1 h; c) equilibrium pH, conditions: [LiCl] = 0.01 M, [NH_4Cl] = 0.1 M, pH = 7.5 - 8.2 (Tris/HCl buffer), [H**L**] = 0.1 M, [TOPO] = 0.2 M in $CHCl_3$, 298 K, 1 h.

For H**L**^{3} and H**L**^{4}, the [TOPO]/[metal+ligand] ratio of the extracted species was investigated by varying the TOPO concentration in the organic phase from 0 - 0.1 M while the degree of Li^+ extracted into the chloroform phase was monitored. The inflection in the corresponding plots obtained from these experiments enabled an estimate of the TOPO to Li^+ ratio in the presence of an excess of this metal ion. The conditions employed for these studies are thus somewhat different to those used for the extraction experiments discussed earlier. The extraction data were shown in Fig. S73a and b from which the inflection points in the corresponding plots were obtained. For both ligands an approximate [TOPO] : [metal+ligand] ratio of 1 : 3 was determined, indicating that both ligands can extract Li^+ by the formation of a complex with 0.33 eq. TOPO per metal ion under the conditions employed. In contrast, for HBTA, an inflection at 1.88 was obtained (Fig. S73c), indicating a stoichiometry for the extracted species of 1 : 1 : 2 (Li^+ : $[BTA]^-$: TOPO). Li^+ loading experiments were also undertaken for H**L**^{3} and H**L**^{4} with TBPO, with the plots shown in Fig. S74. Both experiments gave inflections in the corresponding plots at approximately 1.

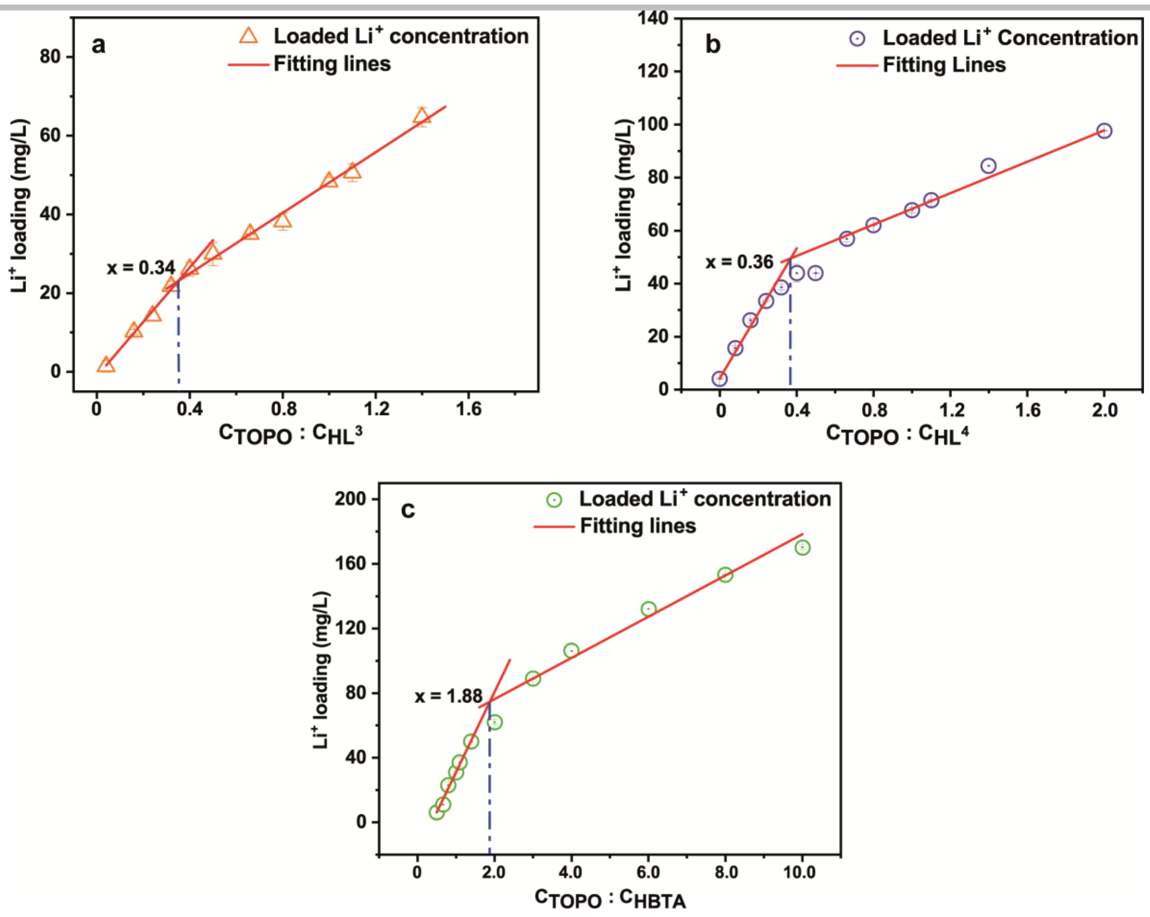

Figure S73. Li^+ loading experiments of a) H**L**^{3} in presence of TOPO, [LiCl] = 0.05 M, [NH_4Cl] = 0.1 M, pH = 8.3 (Tris/HCl buffer), [H**L**^{3}] = 0.05 M, [TOPO] = 0.002-0.07 M in $CHCl_3$, 298 K, 1 h; b) H**L**^{4} in presence of TOPO, [LiCl] = 0.05 M, [NH_4Cl] = 0.1 M, pH = 8.5 (Tris/HCl buffer), [H**L**^{4}] = 0.05 M, [TOPO] = 0-0.1 M in $CHCl_3$, 298 K, 1 h; c) HBTA in presence of TOPO, [LiCl] = 0.05 M, [NH_4Cl] = 0.1 M, pH = 8.3 (Tris/HCl buffer), [HBTA] = 0.05 M, [TOPO] = 0.025-0.5 M in $CHCl_3$, 298 K, 1 h.

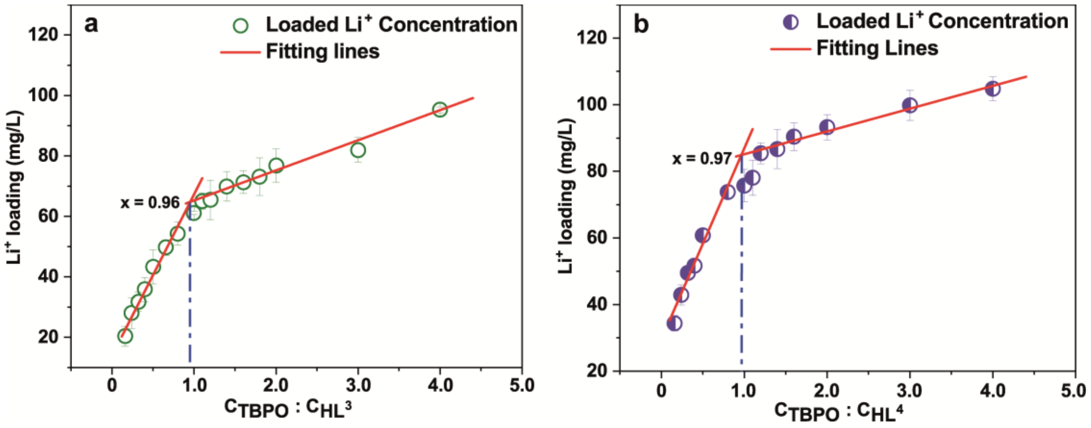

Figure S74. Li^+ loading experiments of a) H**L**^{3} in presence of TBPO, [LiCl] = 0.05 M, [NH_4Cl] = 0.1 M, pH = 8.3 (Tris/HCl buffer), [H**L**^{3}] = 0.05 M, [TBPO] = 0.008-0.2 M in $CHCl_3$, 298 K, 1 h; b) H**L**^{4} in presence of TBPO, [LiCl] = 0.05 M, [NH_4Cl] = 0.1 M, pH = 8.2 (Tris/HCl buffer), [H**L**^{4}] = 0.05 M, [TBPO] = 0.008-0.2 M in $CHCl_3$, 298 K, 1 h.

S11. Selectivity studies

S11.1 Selectivity studies by NMR under LLE conditions

The NMR selectivity studies were also carried out under LLE conditions to confirm the potential application. The organic phase was prepared with 0.1 M H**L**[4] and 0.1 M TOPO in $CHCl_3$ while five types aqueous phase were prepared with 0.1 M LiCl, 0.1 M NaCl, 0.1 M KCl, 0.1 M CsCl respectively as well as no metal ions system in Tris/HCl buffer solution (all five types aqueous solution included 0.5 M NH_4Cl, initial pH = 8.2). The two phases were stirred in centrifuge tube 1 h at room temperature using a 1 : 1 phase ratio (O/A). The organic phases were taken out and NMR measurements were carried out. Partial and full ^{31}P NMR spectra are shown in Figure S75 and S76 respectively.

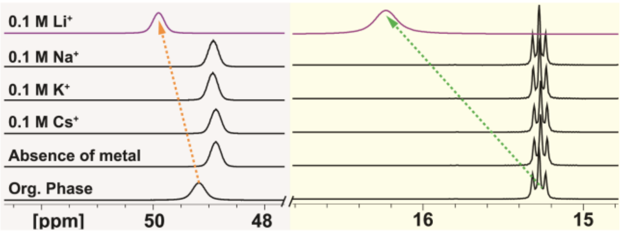

Figure S75. Partial ^{31}P NMR spectra of 0.1 M H**L**[4] in the presence of 0.1 M TOPO in $CHCl_3$ (Org. phase), treated with aqueous phase (Tris/HCl buffer solution, initial pH = 8.2) in absence of metal ions or in presence of 0.1 M Cs^+/0.1 M K^+/0.1 M Na^+/0.1 M Li^+ ($CDCl_3$, 300 K). TOPO (gray shaded, left), H**L**[4] (light yellow shaded, right).

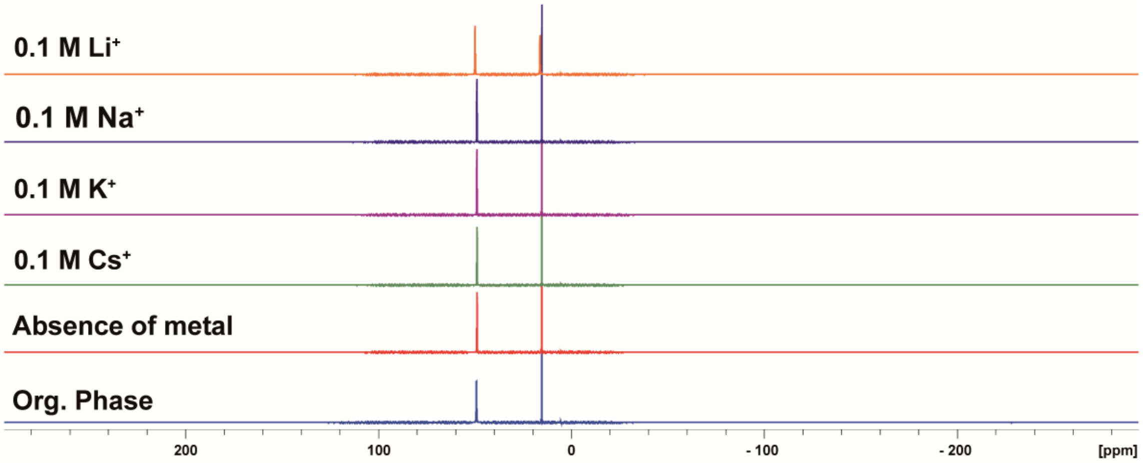

Figure S76. Full ^{31}P NMR spectra of 0.1 M H**L**[4] in the presence of 0.1 M TOPO in $CHCl_3$ (Org. phase), treated with aqueous phase (Tris/HCl buffer solution, initial pH = 8.2) in absence of metal ions or in presence of 0.1 M Cs^+/0.1 M K^+/0.1 M Na^+/0.1 M Li^+ ($CDCl_3$, 300 K).

As shown in Figure S75, more obvious difference is observed for the ^{31}P resonance of H**L**[4] and TOPO. Compared with initial organic phase, only apparent downfield shifts were observed when the organic phase is treated with an aqueous phase containing 0.1 M LiCl. Moreover, the ^{31}P resonance of H**L**[4] broadens and the splitting disappears upon treatment with 0.1 M LiCl. These results confirm the high selectivity for Li^+ over other alkali metal ions which is observed in non-aqueous phase (CD_3CN) and also provide further evidence for the highly selective Li^+ binding in presence of TOPO under LLE conditions.

S11.2 Selectivity studies under SLE conditions

To further investigate the affinity/selectivity of the 4-phosphoryl pyrazolone ligands towards Li^+, solid-liquid extraction (SLE) studies with [TBA]**L**[4] were carried out. [TBA]**L**[4] (5 mM) and a mixture of [TBA]**L**[4] and TOPO (5 mM each) were dissolved in $CHCl_3$ and the alkali chlorides LiCl, NaCl, KCl, CsCl were added in 50-fold excess (250 mM). The mixtures were stirred for 48h at r.t. then filtered. The resulting filtrate was treated with the same volume of 0.2 M H_2SO_4 for 0.5 h and the resulting aqueous phase was diluted before ICP-

OES measurement. The loading percentage was calculated using moles of metal ions loaded divided by the moles of ligands applied. Control experiments in parallel with blank $CHCl_3$ and 5 mM TOPO in $CHCl_3$ showed below 0.2 % loading for Li^+ while Na^+, K^+, and Cs^+ were not detected. The loading reported was corrected accordingly. All experiments were performed in triplicate, and both the average value and standard deviation are reported.

S12. Crystallographic details.

S12.1 Structure solution and refinement

Suitable single crystals were coated with Paratone-N oil or Fomblin Y25 PFPE oil, mounted using a glass fibre and cooled in the cold nitrogen stream. X-ray diffraction data were collected at 100K on a Rigaku Oxford Diffraction SuperNova diffractometer using Cu K_α radiation (λ = 1.54184 Å) generated by a micro-focus source. The data reduction and absorption correction was performed using CrysAlisPro.[8] The structures were solved using Olex2[9] with the SHELXT package[10] and were refined with SHELXS.[11] Hydrogen atoms bonded to carbon atoms were added to the structure models at calculated positions using the riding model. Images of the structures were produced with Olex2[9] software. All structures have been deposited with the Cambridge Crystallographic Data Centre (CCDC) and can be accessed free of charge under the numbers 2101589-2101604.
In order to allow convergent refinement restraints had to be applied on some structures. In the structure of H**L**^{2} and complex **10** were refined applying SIMU and RIGU restraint for disordered functional groups and the solvent molecule. In complexes **12** and **13** the n-pentane solvent molecules are restrained by SADI, SIMU and DFIX. Complexes **10** and **11** were refined only to moderate wR_2 of 26.6% and 20.2%, due to limited crystal quality.

Table S7. Crystallographic data for H**L**^{2} - H**L**^{4}.

	H**L**^{2}	H**L**^{3}	H**L**^{4}
Empirical formula	$C_{16}H_{22}N_3O_6P$	$C_{18}H_{26}N_3O_6P$	$C_{21}H_{24}N_3O_6P$
Formula weight	383.33	411.39	445.40
Color, habit	yellow, block	orange, block	yellow, block
Temperature/K	100.01(10)	100.00(10)	100.01(10)
Crystal system	monoclinic	triclinic	triclinic
Space group	$P2_1/c$	P-1	P-1
a/Å	17.83450(10)	9.3858(3)	8.0663(2)
b/Å	17.32040(10)	10.4703(3)	15.6606(3)
c/Å	12.18950(10)	11.7611(4)	17.6339(2)
α/°	90	71.657(3)	100.3530(10)
β/°	95.1440(10)	78.842(3)	92.8980(10)
γ/°	90	70.319(3)	92.425(2)
Volume/Å^3	3750.18(4)	1028.01(6)	2185.50(7)
Z	8	2	4
ρ_{calc}g/cm^3	1.358	1.329	1.354
μ/mm^{-1}	1.637	1.529	1.487
F(000)	1616.0	436.0	936.0
Crystal size/mm^3	0.284 × 0.167 × 0.132	0.464 × 0.227 × 0.125	0.221 × 0.165 × 0.119
Radiation	CuKα (λ = 1.54184)	CuKα (λ = 1.54184)	CuKα (λ = 1.54184)
2Θ range for data collection/°	4.974 to 153.4	7.958 to 152.924	5.744 to 153.028
Index ranges	-22 ≤ h ≤ 22, -21 ≤ k ≤ 8, -15 ≤ l ≤ 15	-11 ≤ h ≤ 7, -13 ≤ k ≤ 12, -14 ≤ l ≤ 14	-10 ≤ h ≤ 9, -19 ≤ k ≤ 19, -12 ≤ l ≤ 22
Reflections collected	24704	9339	25644
Independent reflections	7807 [R_{int} = 0.0255, R_{sigma} = 0.0234]	4272 [R_{int} = 0.0167, R_{sigma} = 0.0194]	9055 [R_{int} = 0.0212, R_{sigma} = 0.0248]
Data/restraints/parameters	7807/156/575	4272/0/263	9055/0/575
Goodness-of-fit on F^2	1.048	1.024	1.055
Final R indexes [I>=2σ (I)]	R_1 = 0.0475, wR_2 = 0.1279	R_1 = 0.0310, wR_2 = 0.0794	R_1 = 0.0338, wR_2 = 0.0904
Final R indexes [all data]	R_1 = 0.0501, wR_2 = 0.1302	R_1 = 0.0319, wR_2 = 0.0804	R_1 = 0.0377, wR_2 = 0.0933
Largest diff. peak/hole / e Å^{-3}	0.33/-0.41	0.30/-0.43	0.33/-0.51
CCDC	2101594	2101595	2101600

Table S8. Crystallographic data for [TBA]**L**[2] - [TBA]**L**[4].

	[TBA]**L**[2]	[TBA]**L**[3]	[TBA]**L**[4]
Empirical formula	$C_{32}H_{57}N_4O_6P$	$C_{34}H_{61}N_4O_6P$	$C_{37}H_{59}N_4O_6P$
Formula weight	624.78	652.83	686.85
Color, habit	red, block	orange, block	yellow, block
Temperature/K	100.01(10)	100.01(10)	100.01(10)
Crystal system	triclinic	triclinic	monoclinic
Space group	P-1	P-1	$P2_1/n$
a/Å	9.4534(2)	9.4128(4)	14.7485(2)
b/Å	13.6422(3)	13.7217(8)	17.81530(10)
c/Å	14.6689(4)	16.0757(12)	15.0548(2)
α/°	102.960(2)	106.912(6)	90
β/°	91.846(2)	91.464(5)	106.5170(10)
γ/°	107.482(2)	107.581(4)	90
Volume/Å^3	1748.31(8)	1879.0(2)	3792.40(8)
Z	2	2	4
ρ_{calc}g/cm^3	1.187	1.154	1.203
μ/mm^{-1}	1.065	1.010	1.030
F(000)	680.0	712.0	1488.0
Crystal size/mm^3	0.353 × 0.199 × 0.154	0.352 × 0.164 × 0.071	0.307 × 0.235 × 0.188
Radiation	CuKα (λ = 1.54184)	CuKα (λ = 1.54184)	CuKα (λ = 1.54184)
2Θ range for data collection/°	6.218 to 153.424	5.792 to 153.864	7.404 to 153.504
Index ranges	-11 ≤ h ≤ 11, -17 ≤ k ≤ 17, -18 ≤ l ≤ 8	-11 ≤ h ≤ 11, -17 ≤ k ≤ 17, -18 ≤ l ≤ 20	-18 ≤ h ≤ 18, -22 ≤ k ≤ 20, -18 ≤ l ≤ 18
Reflections collected	17305	21095	25754
Independent reflections	7154 [R_{int} = 0.0282, R_{sigma} = 0.0325]	7779 [R_{int} = 0.0606, R_{sigma} = 0.0665]	7916 [R_{int} = 0.0269, R_{sigma} = 0.0232]
Data/restraints/parameters	7154/0/408	7779/0/416	7916/0/441
Goodness-of-fit on F^2	1.032	1.046	1.024
Final R indexes [I>=2σ (I)]	R_1 = 0.0434, wR_2 = 0.1168	R_1 = 0.0623, wR_2 = 0.1526	R_1 = 0.0414, wR_2 = 0.1105
Final R indexes [all data]	R_1 = 0.0459, wR_2 = 0.1200	R_1 = 0.0909, wR_2 = 0.1758	R_1 = 0.0431, wR_2 = 0.1125
Largest diff. peak/hole / e Å^{-3}	0.27/-0.40	0.62/-0.35	0.38/-0.47
CCDC	2101598	2101601	2101604

Table S9. Crystallographic data for $[Li_2(\mathbf{L}^2)_2(CH_3CN)_2]$ (**4**) and $[Li_2(\mathbf{L}^3)_2(CH_3CN)_2]$ (**5**).

	**4**	**5**
Empirical formula	$C_{36}H_{48}Li_2N_8O_{12}P_2$	$C_{40}H_{56}Li_2N_8O_{12}P_2$
Formula weight	860.64	916.74
Color, habit	yellow, block	yellow, block
Temperature/K	100.01(10)	100.01(10)
Crystal system	triclinic	triclinic
Space group	P-1	P-1
a/Å	9.5490(3)	9.2928(10)
b/Å	9.5671(4)	9.9371(8)
c/Å	13.4460(3)	14.0913(8)
α/°	86.808(2)	83.354(5)
β/°	76.561(2)	85.869(7)
γ/°	63.631(4)	63.496(9)
Volume/Å^3	1068.84(7)	1156.35(19)
Z	1	1
ρ_{calc}g/cm^3	1.337	1.316
μ/mm^{-1}	1.504	1.421
F(000)	452.0	484.0
Crystal size/mm^3	0.306 × 0.189 × 0.108	0.249 × 0.055 × 0.046
Radiation	CuKα (λ = 1.54184)	CuKα (λ = 1.54184)
2Θ range for data collection/°	6.77 to 153.56	6.316 to 154.518
Index ranges	-11 ≤ h ≤ 11, -11 ≤ k ≤ 11, -16 ≤ l ≤ 16	-11 ≤ h ≤ 11, -11 ≤ k ≤ 12, -11 ≤ l ≤ 17
Reflections collected	9985	11794
Independent reflections	4438 [R_{int} = 0.0175, R_{sigma} = 0.0212]	4794 [R_{int} = 0.0524, R_{sigma} = 0.0549]
Data/restraints/parameters	4438/0/277	4794/0/317
Goodness-of-fit on F^2	1.040	1.041
Final R indexes [I>=2σ (I)]	R_1 = 0.0314, wR_2 = 0.0807	R_1 = 0.0565, wR_2 = 0.1550
Final R indexes [all data]	R_1 = 0.0326, wR_2 = 0.0819	R_1 = 0.0648, wR_2 = 0.1655
Largest diff. peak/hole / e Å^{-3}	0.28/-0.49	0.38/-0.54
CCDC	2101602	2101589

Table S10. Crystallographic data for [Li_2(**L**[4])$_2$(CH_3CN)$_2$] (**6**) and [Li(**L**[1])(EtOH)$_2$] (**7**).

	**6**	**7**
Empirical formula	$C_{46}H_{52}Li_2N_8O_{12}P_2$	$C_{16}H_{22}LiN_3O_6$
Formula weight	984.77	359.30
Color, habit	yellow, block	yellow, block
Temperature/K	99.97(17)	100.01(10)
Crystal system	triclinic	triclinic
Space group	P-1	P-1
a/Å	10.2728(4)	8.4520(9)
b/Å	10.3803(3)	9.7613(5)
c/Å	12.3915(4)	10.8896(8)
α/°	77.202(3)	89.207(5)
β/°	84.685(3)	89.914(7)
γ/°	75.283(3)	81.679(7)
Volume/Å^3	1245.34(8)	888.88(12)
Z	1	2
ρ_{calc}g/cm^3	1.313	1.342
μ/mm^{-1}	1.363	0.855
F(000)	516.0	380.0
Crystal size/mm^3	0.196 × 0.118 × 0.098	0.371 × 0.118 × 0.049
Radiation	CuKα (λ = 1.54184)	CuKα (λ = 1.54184)
2Θ range for data collection/°	7.322 to 153.076	8.12 to 153.384
Index ranges	-11 ≤ h ≤ 12, -13 ≤ k ≤ 13, -15 ≤ l ≤ 15	-10 ≤ h ≤ 10, -12 ≤ k ≤ 11, -13 ≤ l ≤ 11
Reflections collected	12946	7520
Independent reflections	5143 [R_{int} = 0.0372, R_{sigma} = 0.0376]	3652 [R_{int} = 0.0467, R_{sigma} = 0.0478]
Data/restraints/parameters	5143/0/321	3652/0/247
Goodness-of-fit on F^2	1.061	1.076
Final R indexes [I>=2σ (I)]	R_1 = 0.0685, wR_2 = 0.1730	R_1 = 0.0573, wR_2 = 0.1625
Final R indexes [all data]	R_1 = 0.0727, wR_2 = 0.1818	R_1 = 0.0637, wR_2 = 0.1702
Largest diff. peak/hole / e Å^{-3}	1.13/-0.55	0.43/-0.38
CCDC	2101590	2101591

Table S11. Crystallographic data for [Li_2(**L**[1])$_2$(TBPO)$_2$] (**8**) and [Li_2(**L**[3])$_2$(TBPO)$_2$] (**9**).

	**8**	**9**
Empirical formula	$C_{48}H_{74}Li_2N_6O_{10}P_2$	$C_{60}H_{104}Li_2N_6O_{14}P_4$
Formula weight	970.95	1271.25
Color, habit	yellow, block	yellow, block
Temperature/K	100.00(10)	100.00(10)
Crystal system	triclinic	triclinic
Space group	P-1	P-1
a/Å	9.3184(4)	13.2736(2)
b/Å	11.7463(4)	22.3075(4)
c/Å	12.8352(4)	24.1042(4)
α/°	95.873(3)	81.2330(10)
β/°	108.515(3)	88.8570(10)
γ/°	98.063(3)	89.8260(10)
Volume/Å^3	1302.77(9)	7052.5(2)
Z	1	4
ρ_{calc}g/cm^3	1.238	1.197
μ/mm^{-1}	1.245	1.491
F(000)	520.0	2736.0
Crystal size/mm^3	0.307 × 0.207 × 0.157	0.846 × 0.397 × 0.278
Radiation	CuKα (λ = 1.54184)	CuKα (λ = 1.54184)
2Θ range for data collection/°	7.354 to 153.642	5.03 to 153.742
Index ranges	-9 ≤ h ≤ 11, -14 ≤ k ≤ 14, -16 ≤ l ≤ 14	-16 ≤ h ≤ 16, -22 ≤ k ≤ 28, -30 ≤ l ≤ 30
Reflections collected	13288	61729
Independent reflections	5393 [R_{int} = 0.0186, R_{sigma} = 0.0203]	28912 [R_{int} = 0.0414, R_{sigma} = 0.0480]
Data/restraints/parameters	5393/0/312	28912/0/1585
Goodness-of-fit on F^2	1.035	1.027
Final R indexes [I>=2σ (I)]	R_1 = 0.0391, wR_2 = 0.1047	R_1 = 0.0535, wR_2 = 0.1457
Final R indexes [all data]	R_1 = 0.0398, wR_2 = 0.1056	R_1 = 0.0583, wR_2 = 0.1516
Largest diff. peak/hole / e Å^{-3}	0.38/-0.41	0.84/-0.56
CCDC	2101592	2101603

Table S12. Crystallographic data for $[Li_2(\mathbf{L}^4)_2(TBPO)_2]\cdot 2CH_2Cl_2$ (**10**) and $[Li_2(\mathbf{L}^4)_2(TBP)_2]$ (**11**).

	**10**	**11**
Empirical formula	$C_{68}H_{104}Cl_4Li_2N_6O_{14}P_4$	$C_{66}H_{100}Li_2N_6O_{20}P_4$
Formula weight	1509.13	1435.27
Color, habit	orange, plate	yellow, block
Temperature/K	100.01(10)	100.00(10)
Crystal system	monoclinic	triclinic
Space group	$P2_1/n$	P-1
a/Å	14.9939(9)	9.9987(4)
b/Å	17.6826(6)	13.0950(5)
c/Å	15.6065(9)	14.5751(4)
α/°	90	86.942(3)
β/°	109.669(7)	79.318(3)
γ/°	90	78.995(4)
Volume/Å^3	3896.3(4)	1840.49(12)
Z	2	1
ρ_{calc}g/cm^3	1.286	1.295
μ/mm^{-1}	2.667	1.557
F(000)	1600.0	764.0
Crystal size/mm^3	0.33 × 0.245 × 0.035	0.146 × 0.076 × 0.028
Radiation	CuKα (λ = 1.54184)	CuKα (λ = 1.54184)
2Θ range for data collection/°	7.074 to 156.684	6.172 to 154.96
Index ranges	-18 ≤ h ≤ 18, -22 ≤ k ≤ 15, -19 ≤ l ≤ 19	-12 ≤ h ≤ 12, -16 ≤ k ≤ 16, -12 ≤ l ≤ 18
Reflections collected	42731	21933
Independent reflections	8182 [R_{int} = 0.0868, R_{sigma} = 0.0509]	7651 [R_{int} = 0.0535, R_{sigma} = 0.0497]
Data/restraints/parameters	8182/21/477	7651/0/449
Goodness-of-fit on F^2	1.113	1.040
Final R indexes [I>=2σ (I)]	R_1 = 0.1113, wR_2 = 0.2594	R_1 = 0.0711, wR_2 = 0.1861
Final R indexes [all data]	R_1 = 0.1238, wR_2 = 0.2661	R_1 = 0.0800, wR_2 = 0.2025
Largest diff. peak/hole / e Å^{-3}	0.86/-0.66	0.98/-0.59
CCDC	2101599	2101593

Table S13. Crystallographic data forf $[Li_3(\mathbf{L}^3)_3(TOPO)]\cdot 0.65\ C_5H_{12}$ (**12**) and $[Li_3(\mathbf{L}^4)_3(TOPO)]\cdot 0.67\ C_5H_{12}$ (**13**).

	**12**	**13**
Empirical formula	$C_{81.26}H_{133.81}Li_3N_9O_{19}P_4$	$C_{90.36}H_{128.06}Li_3N_9O_{19}P_4$
Formula weight	1685.55	1789.05
Color, habit	yellow, block	yellow, block
Temperature/K	100.01(10)	100.00(10)
Crystal system	trigonal	trigonal
Space group	P-3	P-3
a/Å	21.0527(6)	21.50690(10)
b/Å	21.0527(6)	21.50690(10)
c/Å	12.5774(3)	12.18670(10)
α/°	90	90
β/°	90	90
γ/°	120	120
Volume/Å^3	4827.7(3)	4881.71(6)
Z	1.99998	1.99998
ρ_{calc}g/cm^3	1.160	1.217
μ/mm^{-1}	1.254	1.273
F(000)	1811.0	1908.0
Crystal size/mm^3	0.224 × 0.119 × 0.076	0.244 × 0.188 × 0.097
Radiation	CuKα (λ = 1.54184)	CuKα (λ = 1.54184)
2Θ range for data collection/°	4.846 to 153.514	4.744 to 153.76
Index ranges	-26 ≤ h ≤ 23, -25 ≤ k ≤ 26, -15 ≤ l ≤ 15	-25 ≤ h ≤ 27, -27 ≤ k ≤ 17, -15 ≤ l ≤ 15
Reflections collected	54280	55945
Independent reflections	6740 [R_{int} = 0.0604, R_{sigma} = 0.0309]	6865 [R_{int} = 0.0245, R_{sigma} = 0.0110]
Data/restraints/parameters	6740/15/428	6865/18/404
Goodness-of-fit on F^2	1.033	1.041
Final R indexes [I>=2σ (I)]	R_1 = 0.0517, wR_2 = 0.1342	R_1 = 0.0419, wR_2 = 0.1136
Final R indexes [all data]	R_1 = 0.0629, wR_2 = 0.1422	R_1 = 0.0425, wR_2 = 0.1143
Largest diff. peak/hole / e Å^{-3}	0.49/-0.45	0.73/-0.44
CCDC	2101596	2101597

References

[1] G. R. Fulmer, A. J. M. Miller, N. H. Sherden, H. E. Gottlieb, A. Nudelman, B. M. Stoltz, J. E. Bercaw, K. I. Goldberg, *Organometallics* **2010**, *29*, 2176-2179.
[2] M. K. Purohit, S. K. Chakka, I. Scovell, A. Neschadim, A. M. Bello, N. Salum, Y. Katsman, M. C. Bareau, D. R. Branch, L. P. Kotra, *Bioorg. Med. Chem.* **2014**, *22*, 2739-2752.
[3] a) B. S. Jensen, *Acta Chem. Scand.* **1959**, *13*, 1668-1670; b) V. B. Kurteva, M. A. Petrova, *J. Chem. Educ.* **2014**, *92*, 382-384.
[4] J. Zhang, M. Wenzel, K. Schnaars, F. Hennersdorf, K. Schwedtmann, J. März, A. Rossberg, P. Kaden, F. Kraus, T. Stumpf, J. J. Weigand, *Dalton Trans.* **2021**, *50*, 3550-3558.
[5] B. Corbel, I. L'Hostis-Kervella, J.-P. Haelters, *Synth. Commun.* **1996**, *26*, 2561-2568.
[6] J. Modranka, R. Jakubowski, M. Rozalski, U. Krajewska, A. Janecka, K. Gach, D. Pomorska, T. Janecki, *Eur. J. Med. Chem.* **2015**, *92*, 565-574; b) V. Zsoldos-Mády, O. Ozohanics, A. Csámpai, V. Kudar, D. Frigyes, P. Sohár, *J. Organomet. Chem* **2009**, *694*, 4185-4195.
[7] a) M. Wenzel, F. Hennersdorf, M. Langer, K. Gloe, B. Antonioli, H.-J. Buschmann, L. F. Lindoy, G. Bernhard, K. Gloe, J. J. Weigand, Sep. Sci. Technol, **2018**, *53*, 1273-1281; b) D. Farrell, K. Gloe, K. Gloe, G. Goretzki, V. McKee, J. Nelson, M. Nieuwenhuyzen, I. Pal, H. Stephan, R. M. Town, K. Wichmann, Dalton Trans., **2003**, 1961–1968.
[8] *CrysAlisPRO, Oxford Diffraction/Agilent Technologies UK Ltd, Yarnton, England.*
[9] O. V. Dolomanov, L. J. Bourhis, R. J. Gildea, J. A. K. Howard, H. Puschmann, *J. Appl. Crystallogr.* **2009**, *42*, 339-341.
[10] G. M. Sheldrick, *Acta Crystallogr. Sect. A Found. Adv.* **2015**, *71*, 3-8.
[11] G. M. Sheldrick, *Acta Crystallogr. Sect. C Struct. Chem.* **2015**, *71*, 3-8.
